# Top-Down and Bottom-Up Identification of Proteins by Liquid Extraction Surface Analysis Mass Spectrometry of Healthy and Diseased Human Liver Tissue

**DOI:** 10.1007/s13361-014-0967-z

**Published:** 2014-09-03

**Authors:** Joscelyn Sarsby, Nicholas J. Martin, Patricia F. Lalor, Josephine Bunch, Helen J. Cooper

**Affiliations:** 1 Physical Sciences of Imaging in the Biomedical Sciences Doctoral Training Centre, University of Birmingham, Edgbaston, Birmingham, B15 2TT UK; 2 School of Chemistry, University of Birmingham, Edgbaston, Birmingham, B15 2TT UK; 3 School of Biosciences, University of Birmingham, Edgbaston, Birmingham, B15 2TT UK; 4 Centre for Liver Research and NIHR BRU, School of Immunity and Infection, University of Birmingham, Edgbaston, Birmingham, B15 2TT UK; 5 School of Pharmacy, Boots Science Building, University Park, University of Nottingham, Nottingham, NG7 2RD UK; 6 Present Address: National Physical Laboratory, Hampton Road, Teddington, Middlesex TW11 0LW UK

**Keywords:** LESA, Liquid microjunction, Ambient, Bottom-up, Top-down, Proteomics

## Abstract

Liquid extraction surface analysis mass spectrometry (LESA MS) has the potential to become a useful tool in the spatially-resolved profiling of proteins in substrates. Here, the approach has been applied to the analysis of thin tissue sections from human liver. The aim was to determine whether LESA MS was a suitable approach for the detection of protein biomarkers of nonalcoholic liver disease (nonalcoholic steatohepatitis, NASH), with a view to the eventual development of LESA MS for imaging NASH pathology. Two approaches were considered. In the first, endogenous proteins were extracted from liver tissue sections by LESA, subjected to automated trypsin digestion, and the resulting peptide mixture was analyzed by liquid chromatography tandem mass spectrometry (LC-MS/MS) (bottom-up approach). In the second (top-down approach), endogenous proteins were extracted by LESA, and analyzed intact. Selected protein ions were subjected to collision-induced dissociation (CID) and/or electron transfer dissociation (ETD) mass spectrometry. The bottom-up approach resulted in the identification of over 500 proteins; however identification of key protein biomarkers, liver fatty acid binding protein (FABP1), and its variant (Thr→Ala, position 94), was unreliable and irreproducible. Top-down LESA MS analysis of healthy and diseased liver tissue revealed peaks corresponding to multiple (~15–25) proteins. MS/MS of four of these proteins identified them as FABP1, its variant, α-hemoglobin, and 10 kDa heat shock protein. The reliable identification of FABP1 and its variant by top-down LESA MS suggests that the approach may be suitable for imaging NASH pathology in sections from liver biopsies.

**Electronic supplementary material:**

The online version of this article (doi:10.1007/s13361-014-0967-z) contains supplementary material, which is available to authorized users.

**Graphical Abstract:**
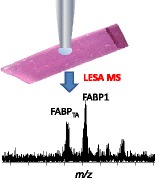
ᅟ

## Introduction

Nonalcoholic steatohepatitis (NASH) is an inflammatory condition of the liver that mimics the damage caused by alcohol without the consumption of large quantities of alcohol. The disease is characterized by the presence of steatosis and fibrosis that can lead to cirrhosis. The lobular destruction and cell loss characteristic of cirrhosis compromise liver function and ultimately lead to liver failure [1]. Causes of the disease remain to be fully characterized but both genetic predisposition and lifestyle choices are important [2]. In common with other liver diseases, serum biomarkers such as elevated amino transferases and a glutamyltransferase are found in patients with NASH, but to date no disease-specific profile of biomarkers has been reported. However, changes in the liver fatty acid binding protein [1, 3–5] show promise. Liver fatty acid binding protein (FABP1 or L-FABP) is a regulator of hepatic lipid metabolism. Recent work by Peng et al. [6] has shown that two single nucleotide polymorphisms in the *FABP1* gene are associated with increased risk of developing non-alcoholic fatty liver disease. The first results in substitution of threonine for alanine at position 94 in FABP1, whereas the second is intronic. Increased risk of disease is linked to the two single nucleotide polymorphisms both individually and cumulatively; however, the mechanisms by which these polymorphisms contribute to the disease process are unknown. Recent work by Huang et al. [7] suggests that the Thr→Ala substitution alters the structure of FABP1 and the conformational changes that accompany binding of long chain fatty acids.

In the present work, we seek to determine whether liquid extraction surface analysis mass spectrometry (LESA MS) of thin tissue sections from human liver could be applied to the study of NASH. Specifically, the aim was to determine whether the approach could be applied for the analysis of protein biomarkers of the disease, with particular emphasis on FABP1 and its Thr→Ala variant. Both bottom-up and top-down approaches have been considered. In the former, endogenous proteins were extracted from the tissue and subjected to automated tryspin digestion [8] before the resulting peptides were analyzed by LC-MS/MS, and in the latter, endogenous proteins were extracted and analyzed intact [9].

LESA, also known as liquid microjunction surface sampling, has the potential to become a useful tool in mass spectrometry imaging of proteins. The technique involves deposition of a solvent droplet from a pipette tip onto a surface [10–12]. The solvent droplet maintains contact with both the surface and the pipette tip for a defined time period (a few seconds) before being re-aspirated. The resulting sample can either be directly electrosprayed or subjected to further manipulation before mass spectrometry analysis. In the present work, automated LESA was performed.

LESA mass spectrometry of intact proteins was first demonstrated by Van Berkel and coworkers [13]. They used a continuous flow liquid microjunction system coupled with an ion trap mass spectrometer to analyze lysozyme protein standard (14.3 kDa) spotted onto a glass surface. Earlier work from our laboratory demonstrated that LESA MS was suitable for the top-down analysis of proteins from dried blood spots. The approach was applied to the screening for known hemoglobin variants [14, 15] and to the diagnosis of unknown hemoglobin variants [16] (~15 kDa) in neonatal samples. A continuous flow version of the LESA technology has been applied to the analysis of hemoglobin in dried blood spots from sheep [13]. More recently, manual LESA was applied to sampling of intact proteins from thin tissue sections of bovine lens, and murine brain and kidney [9].

In order for LESA to be coupled with bottom-up proteomics, a proteolytic digestion step needs to be introduced into the workflow. Two approaches exist; in the first, intact proteins are extracted by LESA prior to digestion [8], and in the second, in-situ digestion is performed prior to liquid microjunction extraction [17, 18]. The first approach was applied to the analysis of dried blood spots, identifying over 100 proteins, and made use of the Advion TriVersa NanoMate (Advion Biosciences, Ithaca, NY, USA) for both the liquid microjunction extraction and the automated digestion procedure. A key challenge for the latter approach is preventing evaporation of the trypsin solution. In-situ digestion was demonstrated for protein arrays (cytochrome *c*, myglobin, and bovine serum albumin) printed on a biomaterial substrate commonly used for cell culture [19]. Quanico et al. [17] applied the in-situ digestion approach, via repeated deposition of trypsin solution, to the analysis of thin tissue sections from frozen rat brain. The approach was subsequently applied to formalin-fixed and paraffin-embedded tissue sections from fallopian tube cancer biopsies [18].

Here, we apply LESA coupled with high resolution MS and MS/MS to survey proteins in sections of human liver. A bottom-up proteomics approach in which intact endogenous proteins are extracted by LESA prior to automated trypsin analysis is compared with a top-down approach in which the proteins are extracted and directly introduced into the mass spectrometer. The bottom-up approach identified more proteins than the top-down approach. Nevertheless, bottom-up identification of FABP1 and its variant is irreproducible, whereas the top-down approach reliably identified this potential biomarker of nonalcoholic liver disease. The results suggest that top-down LESA MS may be a suitable approach for imaging NASH pathology in sections from liver biopsies.

## Methods

### Sample Preparation

The work was approved by the NHS Walsall Local Research Ethics Committee (98/CA5192). All patient liver tissue was collected at The Queen Elizabeth Hospital in Birmingham with written informed patient consent. Healthy liver tissue was collected from donor material surplus to transplantation requirements or from resection margin specimens. Diseased liver specimens were collected upon transplantation surgery. All samples were rapidly processed and snap-frozen in liquid nitrogen prior to storage at –80°C.

Sections of normal human liver tissue of area approximately 1.5 cm^2^ were obtained at a thickness of 10 μm using a CM1810 cryostat (Leica Microsystems, Wetzlar, Germany) and thaw mounted onto glass slides.

### Bottom-Up LESA Mass Spectrometry of Human Liver Tissue Sections

Intact proteins were extracted from healthy human tissue via automated LESA. Samples were mounted onto a 96-well microtiter plate (Thermo Scientific, Loughbrough, UK) and placed in the TriVersa NanoMate nanoelectrospray device. Three of the sample wells contained extraction solvents: one contained 50 mM ammonium bicarbonate, one contained 50% methanol, and one contained 70% methanol. A fourth well contained 0.1 μg/μL trypsin (Trypsin Gold; Promega, Southampton, UK) in 50 mM acetic acid. Surface sampling of the thin tissue section and trypsin digestion was performed using the advanced user interface (AUI) feature of the ChipSoft Manager software, which controls the Triversa Nanomate. Following LESA extraction and digestion, each sample was analyzed in duplicate by LC-MS/MS.

#### Liquid Extraction Surface Analysis

Two μL of extraction solvent was aspirated from the solvent well. The robotic arm relocated to a position above the tissue section and descended to a height of approximately 0.6 mm above the surface. One μL of the solution was dispensed onto the tissue section to form a liquid microjunction. The liquid microjunction was maintained between the probe tip and the surface for 1 s; then 1.3 μL were reaspirated into the pipette tip. The dispense-reaspiration cycle was repeated three times before finally dispensing into a clean well in the microtiter plate. The experiment was conducted using three extraction solutions: (1) 50% methanol, (2) 70% methanol, and (3) 50 mM NH_4_HCO_3_. After extraction, the 50% and 70% methanol solutions were left for 30 min until dry to remove the organic content, and the sample was resuspended in 10 μL 50 mM NH_4_HCO_3_, using one mix cycle (repeat dispense/reaspiration) to promote resuspension. For each of the samples, 4.5 μL of trypsin solution was aspirated from the tryspin-containing well and dispensed into the sample well. The solutions were mixed by a single mix cycle (repeat dispense/reaspiration). The samples were incubated at 37°C for 1 h by using the temperature control unit of the TriVersa NanoMate. At 30 min and 1 h into the incubation, 7 μL of 50 mM NH_4_HCO_3_ was aspirated from the solvent well and added to the sample well in order to account for evaporation. The final volume was approximately 10 μL.

#### LC MS/MS

The microtiter plate was transferred to the HPLC autosampler (Ultimate 3000; Dionex Thermo Fisher Scientific, Loughborough, UK), which is coupled to an Orbitrap Velos ETD mass spectrometer (Thermo Fisher Scientific, Bremen, Germany) via the TriVersa NanoMate. The proteolytic digests were each analyzed in duplicate by LC-MS/MS. Five μL aliquots were injected onto a Pepmap 100, C_18_ 100 μm trap (Thermo Fisher Scientific). The trap was treated with a 5 min wash cycle with 0.1% formic acid prior to injection onto the analytical column (Pepmap 100 reversed phase C18 75 μm, 3 μm, 100 Å; Thermo Fisher Scientific). Peptides were separated using a 30 min 3.2% to 44% ACN (J. T. Baker, Deventer, The Netherlands) gradient at a flow rate of 0.35 μL/min.

Samples eluted into the mass spectrometer via the Triversa Nanomate chip-based nanoelectrospray device. Ionization voltage was 1.4 kV, gas pressure was 0.3 psi, and capillary temperature was 250°C. Mass spectrometry analysis was performed via a ‘top 7’ method in which a survey scan was followed by MS/MS of the seven most abundant precursor ions. Survey scans were acquired in the Orbitrap with a *m/z* range 380–1800, an automatic gain control (AGC) target of 1 × 10^6^ charges, a maximum fill time of 1 s, and a resolution of 60,000 at *m/z* 400. CID was performed in the linear ion trap (AGC target: 30,000 charges) with helium gas and a normalized collision energy of 35%. The width of the precursor isolation window was 2 Th and only multiply charged precursor ions were subjected to CID. The mass exclusion window was *m/z* ±0.05 and the exclusion list was set to 500. Dynamic exclusion was applied for 60 s.

MS/MS data were searched against the SwissProt human database (downloaded November 2012), composed of 20,203 sequences, supplemented with the FABP1 variant, using the Mascot and Sequest algorithms in Proteome Discoverer 1.4. Two sets of parameters were applied. In one set, the parameters were: precursor ion mass accuracy 10 ppm, fragment mass tolerance 0.8 Da, methionine oxidation was allowed as a dynamic modification, up to two missed cleavages in the digestion. In the second set, three missed cleavages were allowed, with all other parameters the same as before. Data were filtered to a false discovery rate of 1% by including only high confidence peptides. The protein grouping algorithm was applied, which grouped all non-unique peptides to the highest scoring protein.

### Top-Down LESA Mass Spectrometry of Human Liver Tissue Sections

Sections of human liver thaw-mounted onto glass slides were washed in a solution of 80% ethanol. The slides were mounted onto the Advion LESA universal plate adapter and an image of the tissue section was acquired using an Epson Perfection V300 photo scanner. The LESA Points software (Advion) was used to select the precise location of the tissue section to be sampled. The universal plate adapter was placed into the TriVersa NanoMate. Extraction/electrospray solution comprising 40% acetonitrile + 1% formic acid was placed in the solvent well.

#### Liquid Extraction Surface Analysis

Two μL of 40% acetonitrile + 1% formic acid was aspirated from the solvent well. The robotic arm relocated to a position above the tissue section and descended to a height of approximately 1 mm above the surface. One μL of the solution was dispensed onto the tissue section to form a liquid microjunction. The liquid microjunction was maintained between the probe tip and the surface for 10 s. Then, 1.3 μL was reaspirated into the pipette tip. The dispense/reaspirate cycle was repeated three times. The sample was introduced into the mass spectrometer via the TriVersa NanoMate, at a flow rate of ~150 nL/min, with gas pressure 1.3 psi, a tip voltage of 1.7 kV, and a capillary temperature of 250°C. All spectra were collected in the Orbitrap at a resolution of 100,000 at *m/z* 400. Full scan mass spectra (*m/z* 500–2000) were collected for 3 min. AGC target was 1 × 10^6^ charges with a maximum injection time of 2 s. CID was performed in the ion trap at a normalized collision energy of 30% and the fragments detected in the Orbitrap (*m/z* 300–2000). The isolation width was 3 Th. AGC target was 1 × 10^6^ charges with a maximum injection time of 2 s. Each CID MS/MS scan comprised of 20 co-added microscans. Data were recorded for 10 min (~10 scans). ETD was performed in the ion trap and the fragments detected in the Orbitrap. The isolation width was 3 Th. AGC target was 1 × 10^6^ charges with a maximum injection time of 1 s. ETD activation time was 20 ms. Each ETD scan comprised of 20 co-added microscans. Data were recorded for 6 min (~9 scans).

All mass spectra were deconvoluted using Xcalibur’s Xtract function, in order to obtain monoisotopic masses*.* Mass spectra were processed with a signal to noise ratio threshold of three. The processed fragment ion list was submitted to Prosight PTM 2.0 (https://prosightptm2.northwestern.edu) using the simple human database, allowing all modifications, and peak accuracy of 10 ppm. Identity was confirmed by manual analysis using Protein Prospector (http://prospector.ucsf.edu/prospector/mshome.htm).

## Results and Discussion

### Bottom-Up LESA Mass Spectrometry of Human Liver Tissue Sections

**Figure 1:**
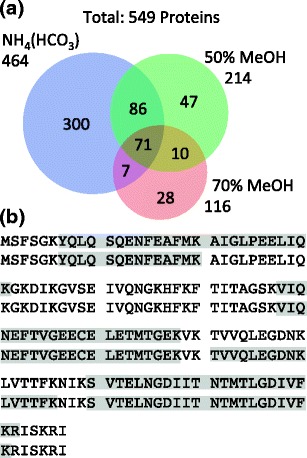
(**a**) Total number of proteins identified from healthy human liver tissue using a bottom-up approach following LESA extraction using solvents comprising 50 mM NH_4_(HCO_3_), 70% methanol, and 50% methanol. (**b**) Sequence coverage obtained for FABP1 following 50 mM NH_4_(HCO_3_) extraction, automated tryptic digestion, and LC MS/MS from two technical replicates

Intact proteins were extracted from healthy human liver tissue via automated direct surface sampling by LESA and subjected to automated trypsin digestion using the method described by Martin et al. [8]. In that work, Martin et al. found that 1 h was the optimum digestion time. Three extraction solvents were considered: 70% (v/v) methanol, 50% (v/v) methanol_,_ and 50 mM ammonium bicarbonate. The three extraction solvents were applied for LESA of three serial sections from a tumor resection margin. For each extraction solvent composition, two technical replicates were performed. Two searches of the data against the protein database were performed in order to determine the effect of the short digestion time. In the first, two missed trypsin cleavages were allowed, and in the second, three missed cleavages were allowed. When two missed cleavages were allowed, a total of 475 non-redundant proteins were detected: 428 proteins were detected following the ammonium bicarbonate extraction; 200 were detected following the 50% methanol extraction, and 99 were detected following the 70% methanol extraction. When three missed cleavages were allowed, 549 non-redundant proteins were detected: 464 proteins were detected following the ammonium bicarbonate extraction; 214 were detected following the 50% methanol extraction, and 116 were detected following the 70% methanol extraction. See Figure 1a. Proteins identified are detailed in Supplementary File 1. Single peptide protein identifications are presented in Supplementary File 2. Increasing the organic solvent content of the extraction solution resulted in fewer identifications, and this is perhaps not surprising. This observation has relevance to the top-down analysis and is discussed further below.

The bottom-up protocol used here was originally developed for the analysis of dried blood spots [8]. The number of proteins identified from the liver tissue is over 4-fold greater than were detected in dried blood spots, despite using a much smaller volume for the liquid microjunction (1 μL versus 6 μL). This observation is perhaps expected given that the liver is a center for metabolism within the body and has an abundance of proteins and enzymes [20]. Blood is a small component of liver tissue and abundant proteins such as alpha and beta hemoglobin chains were also detected. It is possible that the numbers of identified proteins could be increased, either by pooling extracted samples or by performing on-tissue digestion [17]. The latter cannot be achieved, at least in our hands, using the TriVersa NanoMate because of the problem of evaporation of the trypsin solution.

Three known liver disease markers were identified in each of the three extractions: the liver fatty acid binding protein (FABP1) (MW 15.1 kDa) [6], gamma-glutamyltransferase (MW 77.3 kDa) [18], and aspartate amino transferase [3]. There are two forms of aspartate amino transferase, one found in the mitochondria (MW 47.5 kDa) and the other found in the cytoplasm (MW 46.2 kDa). The homology between the two sequences is 68% (as calculated by BLAST http://blast.ncbi.nlm.nih.gov/Blast.cgi). Both proteins were identified by unique peptides in the current dataset. Proteins gamma-glutamyltransferase and the mitochondrial aspartate amino transferase were detected with greatest sequence coverages (32.90% and 48.37% respectively) following the ammonium bicarbonate extraction. The greatest sequence coverage (52.52%) for cytoplasmic aspartate amino transferase was observed following extraction with 50% methanol.

**Figure 2:**
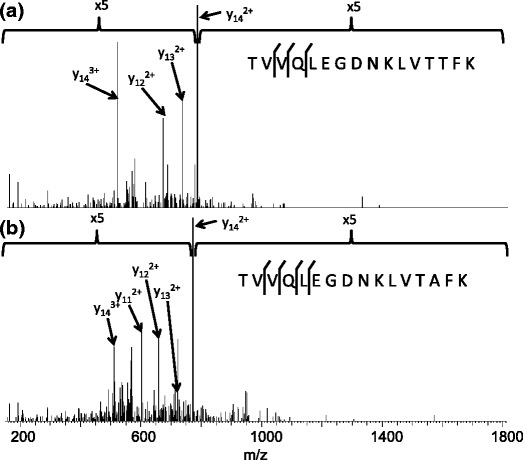
CID mass spectra of +3 ions of tryptic peptides [TVVQLEGDNKLVTTFK] and [TVVQLEGDNKLVTAFK] from FABP1 and FABP1_TA_ identified following LESA extraction with 50 mM NH_4_(HCO_3_) extraction followed by trypsin digestion and LC-MS/MS

FABP1 was detected in each of the six experiments with sequence coverage ranging between 53% and 57% (see Supplementary Figure 1). Figure 1b shows the sequence coverages obtained for the two replicates obtained following extraction with ammonium bicarbonate solution. As described above, FABP1 and its variant implicated in NASH differ by a single amino acid residue (i.e., Thr→Ala at position 94). The tryptic peptide containing residue 94, that is [91-96], was not observed in any of the analyses. (It is possible that an SRM approach may identify the relevant peptide more reproducibly; however, the Orbitrap instrument used in this work is not suited to such an approach). One of the analyses following extraction with ammonium bicarbonate resulted in the identification of the missed-cleavage tryptic peptide [81-96] (*m/z*_meas_ 1791.9931, *m/z*_calc_ 1791.9851, Δ 4.9 ppm) from FABP1. The CID mass spectrum obtained from the [M+3H]^3+^ peptide ions is shown in Figure 2a. The fragmentation sequence coverage for the peptide is poor with no cleavage around the potential substitution site. Clearly, as the variant is the result of a single amino acid substitution and the sequences of FAPB1 and its variant are virtually identical, with the exception of peptide [81-96], the peptides assigned to FABP1 in the database search could also have originated from the variant. The human SwissProt database against which the database was searched was supplemented with the FABP1 variant (hereafter referred to as FABP1_TA_). The peptide [90-96] from FABP1_TA_ was not identified in any of the analyses; however, the missed-cleavage peptide [81-96] was identified in one of the ammonium bicarbonate extraction replicates (*m/z*_meas_ 1761.9831, *m/z*_calc_ 1761.9745, Δ 4.5 ppm). The CID spectrum is shown in Figure 2b. Again, fragmentation sequence coverage is poor and no fragmentation was observed adjacent to the site of substitution. It should be noted that top-down analysis of this tissue sample (see below) revealed the presence of the variant.

### Untargeted Identification of Intact Proteins in Human Liver

Four samples were investigated by top-down LESA mass spectrometry: two NASH samples and two healthy (non-diseased) samples. The non-diseased samples were from (1) a tumor resection margin, and (2) a donor liver not suitable for transplantation.

Liver sections were thaw-mounted onto glass slides and dipped into 80% (v/v) ethanol prior to LESA analysis. This washing step removes lipids and improves protein signal in the mass spectra. The sections were subjected to LESA using extraction solvent comprising 40% (v/v) acetonitrile + 1% formic acid, and the resulting samples introduced directly, via electrospray ionization, into the mass spectrometer. Deconvolution of the resulting mass spectra using the Xtract program suggested that the spectra comprised peaks corresponding to up to 16 proteins in healthy tissue and up to 25 proteins in NASH tissue, with molecular weights ranging between 3000 and 20,000 Da. The results from the bottom-up analyses described above suggest that a greater number of proteins are extracted using ammonium bicarbonate solution; however, the top-down workflow requires that the extraction solution must also be suitable for electrospray ionization. If the extraction and electrospray steps were decoupled, it may be possible to increase the number of top-down identifications.

**Figure 3:**
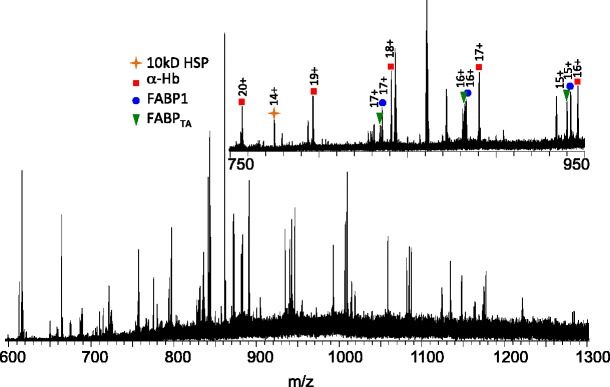
Full scan mass spectrum obtained following LESA MS analysis of healthy human liver tissue. Inset: enlarged region (*m/z* 750–950). Labeled peaks are: FABP1 (blue circle), FABP1_TA_ (green triangle), α-hemoglobin (red square), and 10 kDa heat shock protein (yellow star). Proteins were identified using CID and/or ETD

Figure 3 shows the mass spectrum obtained from a healthy liver sample. Four abundant protein ions were selected for tandem mass spectrometry. Note that the nature of the proteins was not known a priori. Ions at *m/z* 946.38 (+16 charge state) were selected for collision induced dissociation MS/MS. The data were searched against the simple human database in Prosight PTM 2.0, and the protein was identified as the alpha chain of hemoglobin (MW_meas_ 15117.9167, MW_calc_ 15117.8924, Δ 1.6 ppm). Fragments identified are detailed in Supplementary Table 1. The protein sequence coverage following CID was 31%. Ions of the same *m/z* (*m/z* 946.38) observed in the analysis of NASH tissue were selected and subjected to electron transfer dissociation MS/MS. Again, the protein was identified as α-hemoglobin. The ETD protein sequence coverage was 13% and the fragments observed are detailed in Supplementary Table 2. The combined sequence coverage from the two analyses was 42%.

Ions at *m/z* 775.42 (+14 charge state) observed in the mass spectrum from healthy tissue were selected and subjected to both CID MS/MS and ETD MS/MS and the data searched against the simple human database in Prosight PTM 2.0. (A peak at this *m/z* was also observed in NASH tissue however its abundance was insufficient for MS/MS). The protein was identified as mitochondrial 10 kDa heat shock protein (MW_meas_ 10836.8056, MW_calc_ 10836.8485, Δ 3.96 ppm). The protein sequence coverage was 25% (CID) and 21% (ETD), giving an overall coverage of 39%. Details of the fragments observed are given in Supplementary Tables 3 and 4.

**Figure 4:**
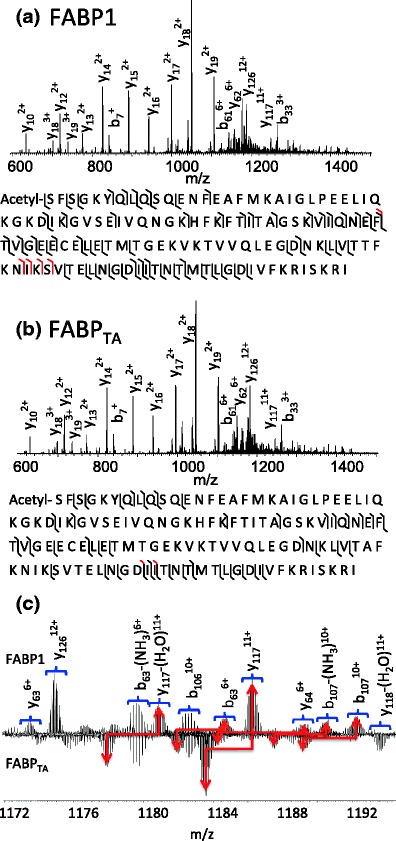
CID mass spectra obtained from +15 ions of (**a**) FABP1 and (**b**) FABP_TA_ following LESA of human liver sections. Insets: observed sequence coverage: b/y ions are shown in black and a ions in red. (**c**) Enlarged region showing *m/z* 1172–1193

The ions at *m/z* 1084.80 and 1087.04 (both +15 charge state) were each selected for CID MS/MS. The two species were identified as FABP1 (MW_meas_ 14111.4161, MW_calc_ 14111.3892, Δ 1.9 ppm) and the variant FABP1_TA_ (MW_meas_ 14081.3600, MW_calc_ 14081.3687, Δ 0.6 ppm). Both proteins were detected with the initiator methionine removed and acetylation of the N-terminus. Therefore, the 92nd and 93rd amino acids in each chain are TT and TA, respectively. The CID sequence coverage obtained was 47% for FABP1 and 33% for the variant FABP1_TA._ Fragments observed are detailed in Supplementary Tables 5 and 6. The site of substitution can be confirmed. The CID MS/MS spectra from both FABP1 and FABP1_TA_ are shown in Figure 4.

**Figure 5:**
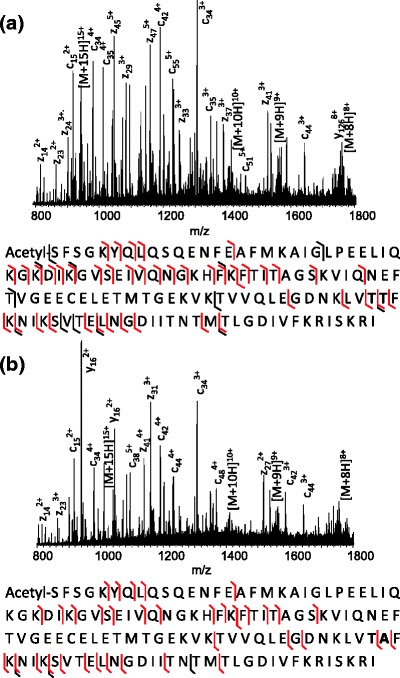
ETD mass spectra obtained from +16 ions of (**a**) FABP1 and (**b**) FABP_TA_ following LESA of NASH human liver sections. Insets: observed sequence coverage: c/z ions are shown in red, a/y ions in black

Ions of *m/z* 942.23 and 940.29 (+16 charge state) (corresponding to FABP1 (MW_meas_ 14111.3943, MW_calc_ 14111.3892, Δ 2.4 ppm) and FABP1_TA_ (MW_meas_ 14081.2845, MW_calc_ 14081.3687, Δ 6.0 ppm) from a NASH sample were subjected to ETD MS/MS. The sequence coverages obtained were 38% and 29%, see Supplementary Tables 7 and 8. The ETD MS/MS spectra of FABP1 and FABP1_TA_ from NASH tissue are shown in Figure 5.

**Figure 6:**
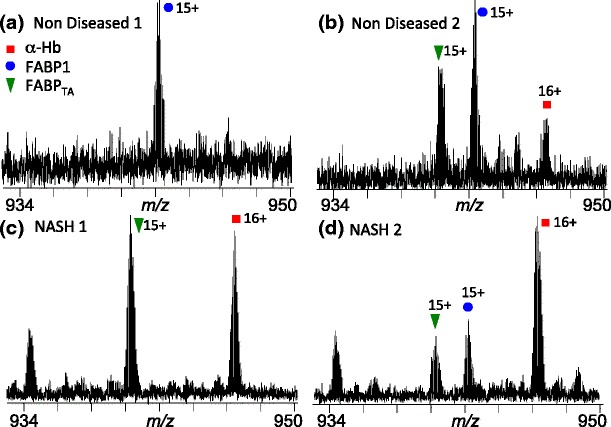
Expanded *m/z* regions from the full scan mass spectra obtained following LESA MS analysis of two healthy human liver sections (**a**) and (**b**), and two NASH human liver sections (**c**) and (**d**). Labeled peaks are FABP1 (+16 charge state) (blue circle), FABP1_TA_ (+16 charge state) (green triangle), and α-Hb (+16 charge state) (red square)

Figure 6 shows expanded *m*/*z* regions containing the +16 charge state of FABP1 and FABP1_TA_ of the mass spectra obtained for the four samples analyzed. Figure 6a and b show the mass spectra from healthy (non-diseased) tissue. In one sample, only FABP1 is observed, whereas in the other both FABP1 and the variant are observed. Figure 6c and d show the mass spectra from NASH tissue. Similarly, one sample contains the variant alone, whereas the other reveals the presence of both FABP1 and FABP1_TA_. As described above, it has been demonstrated recently that individuals with FABP_TA_ have an increased risk of developing NASH [6]. In addition, it was shown that another polymorphism compounds that risk. The observation of the variant in non-diseased tissue could be explained by the risk associated with FABP_TA_ being attenuated by other factors. It is worth noting, however, that the sample containing both FAPB1 and the variant was from a tumor resection margin, whereas the sample containing FAPB1 alone was from a liver rejected for transplant because of the presence of a small lesion. The non-diseased samples are also characterized by reduced signals for α-hemoglobin. In the case of the liver rejected for transplant (Figure 6a), the organ was perfused with buffer (i.e., blood was removed) following removal from the donor. In the case of the tumor resection margin (Figure 6b), it is likely that the surgical procedure resulted in local hemolysis and reduced blood supply.

## Conclusion

We have shown that LESA mass spectrometry is a suitable tool for the investigation of proteins in healthy and diseased human liver tissue. Bottom-up analysis results in identification of far greater numbers of proteins than does top-down analysis. It may be possible to increase the number of top-down identifications by introducing a liquid chromatography step or by decoupling the extraction step from the electrospray ionization step thereby enabling the use of different solvent systems in each case.

Given that the protein FABP1 and its variant FABP_TA_ are implicated in nonalcoholic liver disease, it is particularly important to be able to distinguish between the two forms. The bottom-up approach, although confidently identifying the FABP1 protein in all cases, does not reliably and reproducibly detect the peptide containing the site of amino acid substitution. The results suggest that the bottom-up approach is not suitable for the spatially resolved profiling of NASH tissue sections. (It should be noted, however, that the timescale of the trypsin proteolysis step best-suited for efficient, integrated LESA-based analysis is 1 h. An overnight digestion may yield better sequence coverage, as might alternative proteases). Conversely, the top-down approach easily distinguishes FABP1 and FABP_TA_. Peaks corresponding to the two species are clearly discerned in the mass spectra and are, in fact, some of the most abundant. MS/MS of the intact proteins (either CID or ETD) allows the nature of the proteins to be confirmed, including the site of substitution. Further work will focus on validation of the approach on larger numbers of samples, together with development of LESA-based top-down mass spectrometry for spatially-resolved profiling of NASH tissue.

## Electronic supplementary material

Below is the link to the electronic supplementary material.

Supplementary File 1 (XLSX 189 kb)

Supplementary File 2 (ZIP 20872 kb)

ESM 1 (DOCX 60 kb)

ESM 2 (ZIP 7870 kb)

ESM 3 (ZIP 6136 kb)

ESM 4 (ZIP 3005 kb)

ESM 5 (ZIP 2096 kb)

ESM 6 (ZIP 1626 kb)

ESM 7 (ZIP 1716 kb)
