# Supplementary material for: Top-Down and Bottom-Up Identification of Proteins by Liquid Extraction Surface Analysis Mass Spectrometry of Healthy and Diseased Human Liver Tissue
Source: J Am Soc Mass Spectrom. 2014 Sep 3;25(11):1953–61. doi: 10.1007/s13361-014-0967-z (PMC4197381; doi:10.1007/s13361-014-0967-z)
Supplement: Supplementary file 3 — (DOCX 60 kb) [file 13361_2014_967_MOESM3_ESM.docx]

**Top-down and bottom-up protein identification by liquid extraction surface analysis mass spectrometry of healthy and diseased human liver tissue**

Joscelyn Sarsby^a,b,c^, Nicholas J. Martin^c^, Patricia F. Lalor^d^, Josephine Bunch^a‡^ and Helen J. Cooper^c*^

*^a^Physical Sciences of Imaging in the Biomedical Sciences Doctoral Training Centre, University of Birmingham, Edgbaston, Birmingham, B15 2TT, UK*

*^b^School of Chemistry, University of Birmingham, Edgbaston, Birmingham, B15 2TT, UK*

*^c^School of Biosciences, University of Birmingham, Edgbaston, Birmingham, B15 2TT, UK*

^d^C*entre for Liver Research and NIHR BRU, School of Immunity and Infection, University of Birmingham, Edgbaston, Birmingham, B15 2TT, UK*

*^‡^Current address: National Physical Laboratory, Hampton Road, Teddington, Middlesex, TW11 0LW, UK*

*To whom correspondence should be addressed. Tel: +44 (0)121 414 7527. Email: [h.j.cooper@bham.ac.uk](mailto:h.j.cooper@bham.ac.uk)

Table of Contents

S.I. file 1: All proteins identified using LESA extraction followed by tryptic digestion 3

S.I. file 2: Single Peptide identification 4

S.I. Figure 1: Coverage of FABP in All Extractions and Replicates 5

S.I. Table 1: Fragments observed following CID of +16 ions with *m/z* 946.40 from healthy tissue (identified as α-hemoglobin). 6

S.I. Table 2: Fragments observed following ETD of 16+ ions with *m/z* 946.40 from NASH tissue (identified as α- haemoglobin). 8

S.I. Table 3: Fragments observed following CID of +14 ions with *m/z* 775.42 from healthy tissue (identified as 10kDa heat shock protein (mitochondrial)). 9

S.I. Table 4: Fragments observed following ETD of +14 ions with *m/z* 775.42 from healthy tissue (identified as 10kDa Heat shock protein (mitochondrial)). 10

S.I. Table 5: Fragments observed following CID of +15 ions with *m/z* 1087.04 from healthy tissue (identified as FABP1). 11

S.I. Table 6: Fragments observed following CID of +15 ions with *m/z* 1084.80 from healthy tissue (identified as FABP_TA_). 14

S.I. Table 7: Fragments observed following ETD of +16 ions with *m/z* 942.24 from NASH tissue (identified as FABP1). 16

S.I. Table 8: Fragments observed following ETD of +16 ions with *m/z* 940.24 from NASH tissue (identified as FABP_TA_). 18

# S.I. file 1: All proteins identified using LESA extraction followed by tryptic digestion

See file Supplementary file 1.

# S.I. file 2: Single Peptide identification

See file Supplementary file 2.

# S.I. Figure 1: Coverage of FABP in All Extractions and Replicates

**Ammonium bicarbonate extraction, replicate1** Coverage 54.33%

MSFSGKYQLQ SQENFEAFMK AIGLPEELIQ KGKDIKGVSE IVQNGKHFKF TITAGSKVIQ NEFTVGEECE LETMTGEKVK TVVQLEGDNK LVTTFKNIKS VTELNGDIIT NTMTLGDIVF KRISKRI

**Ammonium bicarbonate extraction, replicate 2** Coverage 57.48%

MSFSGKYQLQ SQENFEAFMK AIGLPEELIQ KGKDIKGVSE IVQNGKHFKF TITAGSKVIQ NEFTVGEECE LETMTGEKVK TVVQLEGDNK LVTTFKNIKS VTELNGDIIT NTMTLGDIVF KRISKRI

**50% Methanol extraction, replicate 1** Coverage 54.33%

MSFSGKYQLQ SQENFEAFMK AIGLPEELIQ KGKDIKGVSE IVQNGKHFKF TITAGSKVIQ NEFTVGEECE LETMTGEKVK TVVQLEGDNK LVTTFKNIKS VTELNGDIIT NTMTLGDIVF KRISKRI

**50% Methanol extraction, replicate 2** Coverage 53.54%

MSFSGKYQLQ SQENFEAFMK AIGLPEELIQ KGKDIKGVSE IVQNGKHFKF TITAGSKVIQ NEFTVGEECE LETMTGEKVK TVVQLEGDNK LVTTFKNIKS VTELNGDIIT NTMTLGDIVF KRISKRI

**70% Methanol extraction, replicate 1** Coverage 53.54%

MSFSGKYQLQ SQENFEAFMK AIGLPEELIQ KGKDIKGVSE IVQNGKHFKF TITAGSKVIQ NEFTVGEECE LETMTGEKVK TVVQLEGDNK LVTTFKNIKS VTELNGDIIT NTMTLGDIVF KRISKRI

**70% Methanol extraction, replicate 2**  Coverage 54.33%

MSFSGKYQLQ SQENFEAFMK AIGLPEELIQ KGKDIKGVSE IVQNGKHFKF TITAGSKVIQ NEFTVGEECE LETMTGEKVK TVVQLEGDNK LVTTFKNIKS VTELNGDIIT NTMTLGDIVF KRISKRI

# S.I. Table 1: Fragments observed following CID of +16 ions with *m/z* 946.40 from healthy tissue (identified as α-hemoglobin).

| Measured (*m/z*) | Theoretical (*m/z*) | Δppm | Assignment |
| --- | --- | --- | --- |
| 527.7966 | 527.7982 | 3.03 | y_9_^2+^ |
| 553.3101 | 553.3093 | -1.45 | y_4_^1+^ |
| 620.8496 | 620.8484 | -1.93 | y_11_^2+^ |
| 623.3494 | 623.3483 | -1.76 | y_23_^4+^ |
| 654.3581 | 654.3570 | -1.68 | y_5_^1+^ |
| 656.3680 | 656.3670 | -1.52 | y_12_^2+^ |
| 672.3766 | 672.3772 | 0.89 | y_18_^3+^ |
| 677.7105 | 677.7093 | -1.77 | b_20_^3+^ |
| 693.3874 | 693.3930 | 8.08 | b_7_-H_2_O^1+^ |
| 696.0576 | 696.0563 | -1.87 | y_19_^3+^ |
| 701.3896 | 701.3884 | -1.71 | b_21_^3+^ |
| 712.9102 | 712.9090 | -1.68 | y_13_^2+^ |
| 741.7438 | 741.7426 | -1.62 | y_20_^3+^ |
| 755.1591 | 755.1578 | -1.72 | y_28_-H_2_O^4+^ |
| 759.6616 | 759.6605 | -1.45 | y_28_^4+^ |
| 767.4425 | 767.4410 | -1.95 | y_6_^1+^ |
| 774.7673 | 774.7654 | -2.45 | y_21_^3+^ |
| 786.4440 | 786.4432 | -1.02 | y_14_^2+^ |
| 787.9330 | 787.9315 | -1.90 | y_29_^4+^ |
| 824.7938 | 824.7918 | -2.42 | y_23_-H_2_O^3+^ |
| 830.7966 | 830.7953 | -1.56 | y_23_^3+^ |
| 836.7724 | 836.7713 | -1.31 | b_25_^3+^ |
| 850.4922 | 850.4907 | -1.76 | y_15_^2+^ |
| 864.1694 | 864.1696 | 0.23 | b_113_^14+^ |
| 864.4794 | 864.4779 | -1.74 | y_24_^3+^ |
| 866.5108 | 866.5094 | -1.62 | y_7_^1+^ |
| 903.4657 | 903.4645 | -1.33 | b_27_^3+^ |
| 905.7042 | 905.7067 | 2.76 | b_100_^13+^ |
| 910.2216 | 910.2201 | -1.65 | b_34_-H_2_O^4+^ |
| 911.2490 | 911.2477 | -1.43 | y_34_^4+^ |
| 911.9266 | 911.9312 | 5.04 | y_58_^7+^ |
| 913.5023 | 913.5007 | -1.75 | y_25_^3+^ |
| 927.2371 | 927.2386 | 1.62 | y_138_^16+^ |
| 929.6957 | 929.6940 | -1.83 | b_79_^9+^ |
| 931.6800 | 931.6774 | -2.79 | y_139_-H_2_O^16+^ |
| 931.6800 | 931.6764 | -3.86 | y_139_-NH_3_^16+^ |
| 932.6817 | 932.6781 | -3.86 | y_139_^16+^ |
| 936.0169 | 936.0148 | -2.24 | y_35_^4+^ |
| 938.2323 | 938.2342 | 2.03 | y_60_^7+^ |
| 956.5168 | 956.5149 | -1.99 | y_26_^3+^ |
| 980.1953 | 980.1939 | -1.43 | y_27_^3+^ |
| 993.8845 | 993.8832 | -1.31 | b_75_^8+^ |
| 1010.3902 | 1010.3882 | -1.98 | b_76_^8+^ |
| 1012.5463 | 1012.5449 | -1.38 | y_28_^3+^ |
| 1018.5608 | 1018.5591 | -1.67 | y_38_^4+^ |
| 1021.9672 | 1021.9661 | -1.08 | y_47_^5+^ |
| 1036.7795 | 1036.7752 | -4.15 | b_78_^8+^ |
| 1043.5821 | 1043.5808 | -1.25 | y_19_^2+^ |
| 1045.7826 | 1045.7798 | -2.68 | b_79_^8+^ |
| 1049.4150 | 1049.4132 | -1.72 | y_57_^6+^ |
| 1050.2403 | 1050.2396 | -0.67 | y_29_^3+^ |
| 1054.5902 | 1054.5891 | -1.04 | y_9_^1+^ |
| 1058.8553 | 1058.8546 | -0.66 | y_68_^7+^ |
| 1063.7533 | 1063.7516 | -1.60 | y_58_^6+^ |
| 1082.7680 | 1082.7659 | -1.94 | y_59_^6+^ |
| 1094.6065 | 1094.6053 | -1.10 | y_60_^6+^ |
| 1095.9266 | 1095.9259 | -0.64 | y_30_^4+^ |
| 1108.9402 | 1108.9441 | 3.52 | y_61_^6+^ |
| 1112.1114 | 1112.1104 | -0.90 | y_20_^2+^ |
| 1135.8672 | 1135.8654 | -1.58 | b_75_^7+^ |
| 1154.4374 | 1154.4426 | 4.50 | b_76_^7+^ |
| 1213.2925 | 1213.2982 | 4.70 | b_102_^9+^ |
| 1245.6904 | 1245.6894 | -0.80 | y_23_^2+^ |
| 1268.3189 | 1268.3173 | -1.26 | b_36_^3+^ |
| 1277.7085 | 1277.7058 | -2.11 | y_47_^4+^ |
| 1296.2147 | 1296.2132 | -1.16 | y_24_^2+^ |
| 1311.7273 | 1311.7267 | -0.46 | y_12_^1+^ |
| 1369.7483 | 1369.7474 | -0.66 | y_25_^2+^ |
| 1518.3146 | 1518.3137 | -0.59 | y_28_^2+^ |

# S.I. Table 2: Fragments observed following ETD of 16+ ions with *m/z* 946.40 from NASH tissue (identified as α- haemoglobin).

| Measured (*m/z*) | Calculated (*m/z*) | Δppm | Assignment |
| --- | --- | --- | --- |
| 521.7976 | 521.7982 | -1.15 | c_10_^2+^ |
| 585.8450 | 585.8457 | -1.19 | c_11_^2+^ |
| 600.3344 | 600.3352 | -1.33 | c_6_^1+^ |
| 621.3635 | 621.3642 | -1.13 | c_12_^2+^ |
| 656.8821 | 656.8828 | -1.07 | c_13_^2+^ |
| 728.4296 | 728.4301 | -0.69 | c_7_^1+^ |
| 749.9214 | 749.9225 | -1.47 | c_14_^2+^ |
| 778.4325 | 778.4332 | -0.90 | c_15_^2+^ |
| 823.4394 | 823.4397 | -0.36 | c_24_^3+^ |
| 829.4771 | 829.4778 | -0.84 | c_8_^1+^ |
| 842.4797 | 842.4807 | -1.19 | c_16_^2+^ |
| 908.4953 | 908.4971 | -1.98 | z_25_^3+.^ |
| 918.9791 | 918.9793 | -0.22 | c_35_^4+.^ |
| 1000.0522 | 1000.0529 | -0.70 | z_18_^2+^ |
| 1042.5890 | 1042.5891 | -0.10 | c_10_^1+^ |
| 1044.8998 | 1044.9000 | -0.19 | z_29_^3+^ |
| 1127.1196 | 1127.1212 | -1.42 | z_42_^4+.^ |
| 1128.8341 | 1128.8350 | -0.80 | c_42_^4+^ |
| 1153.1239 | 1153.1242 | -0.26 | c_23_^2+^ |
| 1159.1432 | 1159.1449 | -1.47 | z_43_^4+.^ |
| 1224.6322 | 1224.6341 | -1.55 | c_35_^3+.^ |
| 1234.6555 | 1234.6559 | -0.32 | c_24_^2+^ |
| 1545.1897 | 1545.1908 | -0.71 | z_43_^3+.^ |

# S.I. Table 3: Fragments observed following CID of +14 ions with *m/z* 775.42 from healthy tissue (identified as 10kDa heat shock protein (mitochondrial)).

| Measured (*m/z*) | Theoretical (*m/z*) | Δppm | Assignment |
| --- | --- | --- | --- |
| 581.2924 | 581.2930 | 1.03 | y_5_^1+^ |
| 659.1627 | 659.1622 | -0.76 | b_60_-NH_3_^9+^ |
| 661.8642 | 661.8644 | 0.30 | b_23_^4+^ |
| 668.3382 | 668.3384 | 0.30 | y_29_^5+^ |
| 679.0526 | 679.0540 | 2.06 | b^609+^ |
| 685.8093 | 685.8081 | -1.75 | b_44_-NH_3_^7+^ |
| 699.8188 | 699.8201 | 1.86 | b_45_-H_2_O^7+^ |
| 702.3912 | 702.3931 | 2.71 | b_45_^7+^ |
| 709.7940 | 709.7950 | 1.41 | b_32_^5+^ |
| 727.0272 | 727.0284 | 1.65 | y_18_^3+^ |
| 739.7415 | 739.7377 | -5.14 | y_63_^9+^ |
| 755.8973 | 755.8986 | 1.72 | y_13_^2+^ |
| 762.9104 | 762.9107 | 0.39 | y_41_^6+^ |
| 799.7760 | 799.7775 | 1.88 | b_44_-H_2_O^6+^ |
| 809.1444 | 809.1454 | 1.24 | y_53_^7+^ |
| 816.2874 | 816.2889 | 1.84 | b_45_-H_2_O^6+^ |
| 819.2888 | 819.2907 | 2.32 | b_45_^6+^ |
| 823.2969 | 823.2980 | 1.34 | y_54_^7+^ |
| 832.0836 | 832.0790 | -5.53 | y_63_-NH_3_^8+^ |
| 833.4447 | 833.4462 | 1.80 | y_55_^7+^ |
| 835.1697 | 835.1711 | 1.68 | y_29_^6+^ |
| 844.6394 | 844.6398 | 0.47 | b_47_-H_2_O^6+^ |
| 847.5953 | 847.5988 | 4.13 | y_56_^7+^ |
| 863.4402 | 863.4421 | 2.20 | y_30_^4+^ |
| 876.0657 | 876.0671 | 1.60 | y_39_^5+^ |
| 880.4868 | 880.4882 | 1.59 | b_40_^5+^ |
| 886.9909 | 886.9920 | 1.24 | b_32_^4+^ |
| 910.9621 | 910.9645 | 2.63 | y_14_^2+^ |
| 915.2896 | 915.2913 | 1.86 | y_41_^5+^ |
| 948.8537 | 948.8555 | 1.90 | b_25_^3+^ |
| 968.4762 | 968.4780 | 1.86 | y_16_^2+^ |
| 1037.8581 | 1037.8606 | 2.41 | y_27_^3+^ |
| 1061.5872 | 1061.5891 | 1.79 | b_9_^1+^ |
| 1094.8294 | 1094.8321 | 2.47 | y_39_^4+^ |
| 1113.2240 | 1113.2258 | 1.62 | y_29_^3+^ |
| 1188.6136 | 1188.6151 | 1.26 | y_31_^3+^ |

# S.I. Table 4: Fragments observed following ETD of +14 ions with *m/z* 775.42 from healthy tissue (identified as 10kDa Heat shock protein (mitochondrial)).

| Measured (*m/z*) | Theoretical (*m/z*) | Δppm | Assignment |
| --- | --- | --- | --- |
| 666.1198 | 666.1210 | 1.80 | c_23_^4+^ |
| 668.0371 | 668.0386 | 2.25 | c_36_^6+^ |
| 690.3671 | 690.3682 | 1.59 | c_6_^1+^ |
| 690.5825 | 690.5835 | 1.45 | c_31_^5+^ |
| 698.8852 | 698.8852 | 0.00 | c_38_^6+^ |
| 716.1491 | 716.1501 | 1.40 | c_25_^4+^ |
| 716.4074 | 716.4090 | 2.23 | c_18_^3+^ |
| 718.4129 | 718.4141 | 1.67 | c_12_^2+^ |
| 736.7445 | 736.7458 | 1.76 | c_40_^6+^ |
| 741.4122 | 741.4120 | -0.27 | c_26_^4+^ |
| 758.4180 | 758.4194 | 1.85 | y_34_^5+^ |
| 771.629 | 771.6288 | -0.26 | a_35_^5+^ |
| 775.9272 | 775.9275 | 0.39 | c_13_^2+^ |
| 801.4445 | 801.4448 | 0.37 | c_36_^5+^ |
| 821.1307 | 821.1324 | 2.07 | c_21_^3+^ |
| 830.2134 | 830.2175 | 4.94 | c_30_^4+^ |
| 838.4611 | 838.4608 | -0.36 | c_38_^5+^ |
| 887.8237 | 887.8256 | 2.14 | c_23_^3+^ |
| 915.2896 | 915.2913 | 1.86 | y_41_^5+^ |
| 964.2852 | 964.2842 | -1.04 | a_35_-NH_3_^4+^ |
| 973.2548 | 973.2561 | 1.34 | z_34_^4+^ |
| 978.1647 | 978.1666 | 1.94 | z_26_^3+^ |
| 1012.2776 | 1012.2785 | 0.89 | z_36_^4+^ |
| 1016.2817 | 1016.2832 | 1.48 | y_36_^4+^ |
| 1044.0497 | 1044.0503 | 0.57 | y_37_-NH_3_^4+^ |
| 1047.8200 | 1047.8242 | 4.01 | c_38_^4+^ |
| 1061.5872 | 1061.5891 | 1.79 | b_9_^1+^ |
| 1100.3869 | 1100.3862 | -0.64 | z_51_^5+^ |
| 1113.2240 | 1113.2258 | 1.62 | y_29_^3+^ |
| 1171.8651 | 1171.8723 | 6.14 | z_42_^4+^ |
| 1297.3378 | 1297.3390 | 0.92 | z_34_^3+^ |
| 1307.1917 | 1307.1938 | 1.61 | z_48_^4+^ |
| 1349.3694 | 1349.3690 | -0.30 | z_36_^3+^ |
| 1392.0636 | 1392.0630 | -0.43 | z_37_^3+^ |
| 1562.1624 | 1562.1607 | -1.09 | z_42_^3+^ |

# S.I. Table 5: Fragments observed following CID of +15 ions with *m/z* 1087.04 from healthy tissue (identified as FABP1).

| Measured (*m/z*) | Theoretical (*m/z*) | Δppm | Assignment |
| --- | --- | --- | --- |
| 620.6970 | 620.7011 | -6.61 | y_16_-H_2_O^3+^ |
| 630.4153 | 630.4192 | -6.19 | y_10_^2+^ |
| 664.7148 | 664.7189 | -6.17 | y_17_^3+^ |
| 687.9283 | 687.9326 | -6.25 | y_11_^2+^ |
| 692.3941 | 692.3980 | -5.63 | y_18_-H_2_O^3+^ |
| 698.3972 | 698.4015 | -6.16 | y_18_^3+^ |
| 712.3259 | 712.3301 | -5.90 | b_6_^1+^ |
| 716.4390 | 716.4434 | -6.14 | y_12_^2+^ |
| 730.0882 | 730.0927 | -6.16 | y_19_-H_2_O^3+^ |
| 736.0916 | 736.0962 | -6.25 | y_19_^3+^ |
| 772.9807 | 772.9854 | -6.08 | y_13_^2+^ |
| 823.5043 | 823.5096 | -6.44 | y_14_^2+^ |
| 831.1353 | 831.1403 | -6.02 | y_22_^3+^ |
| 840.3837 | 840.3886 | -5.83 | b_7_^1+^ |
| 843.8865 | 843.8916 | -6.04 | b_14_^2+^ |
| 869.1495 | 869.1547 | -5.98 | y_23_^3+^ |
| 880.0189 | 880.0242 | -6.02 | y_15_-H_2_O^2+^ |
| 885.0999 | 885.1049 | -5.65 | y_39_^5+^ |
| 889.0242 | 889.0295 | -5.96 | y_15_^2+^ |
| 906.8436 | 906.8493 | -6.29 | y_24_^3+^ |
| 919.7096 | 919.7146 | -5.44 | y_41_^5+^ |
| 930.5424 | 930.5480 | -6.02 | y_16_-H_2_O^2+^ |
| 935.4568 | 935.4621 | -5.67 | b_8_-H_2_O^1+^ |
| 939.5477 | 939.5533 | -5.96 | y_16_^2+^ |
| 983.5407 | 983.5461 | -5.49 | y_26_^3+^ |
| 985.2162 | 985.2200 | -3.82 | y_53_^6+^ |
| 987.5637 | 987.5695 | -5.87 | y_17_-H_2_O^2+^ |
| 992.8021 | 992.8067 | -4.63 | y_35_^4+^ |
| 996.5689 | 996.5748 | -5.92 | y_17_^2+^ |
| 1007.2662 | 1007.2725 | -6.25 | b_35_^4+^ |
| 1010.8091 | 1010.8126 | -3.46 | b_63_-NH_3_^7+^ |
| 1017.3183 | 1017.3238 | -5.41 | y_36_^4+^ |
| 1023.7342 | 1023.7347 | -0.49 | y_55_^6+^ |
| 1033.9520 | 1033.9573 | -5.13 | y_93_^10+^ |
| 1038.0872 | 1038.0933 | -5.88 | y_18_-H_2_O^2+^ |
| 1042.5673 | 1042.5733 | -5.79 | y_56_-H_2_O^6+^ |
| 1045.4028 | 1045.4085 | -5.42 | y_56_^6+^ |
| 1045.5881 | 1045.5948 | -6.41 | y_37_^4+^ |
| 1047.0925 | 1047.0986 | -5.83 | y_18_^2+^ |
| 1060.0547 | 1060.0603 | -5.25 | b_57_^6+^ |
| 1063.5144 | 1063.5207 | -5.92 | b_9_-H_2_O^1+^ |
| 1064.2476 | 1064.2558 | -7.67 | y_57_^6+^ |
| 1090.7665 | 1090.7709 | -4.03 | b_48_^6+^ |
| 1094.6290 | 1094.6354 | -5.85 | y_19_-H_2_O^2+^ |
| 1100.2465 | 1100.2511 | -4.15 | b_59_^6+^ |
| 1100.5572 | 1100.5636 | -5.82 | b_39_^4+^ |
| 1103.6342 | 1103.6406 | -5.80 | y_19_^2+^ |
| 1116.5825 | 1116.5871 | -4.15 | b_60_-NH_3_^6+^ |
| 1119.2524 | 1119.2582 | -5.15 | b_60_^6+^ |
| 1121.0315 | 1121.0355 | -3.59 | b_111_-NH_3_^11+^ |
| 1122.5780 | 1122.5834 | -4.83 | b_111_^11+^ |
| 1124.4329 | 1124.4382 | -4.68 | y_60_^6+^ |
| 1128.5775 | 1128.5846 | -6.29 | b_40_^4+^ |
| 1131.5600 | 1131.5663 | -5.60 | b_106_^9+^ |
| 1137.9231 | 1137.9276 | -3.93 | b_61_-NH_3_^6+^ |
| 1140.5886 | 1140.5941 | -4.82 | b_50_^5+^ |
| 1140.7582 | 1140.7653 | -6.19 | b_61_^6+^ |
| 1145.6049 | 1145.6096 | -4.10 | y_123_-H_2_O^12+^ |
| 1146.1067 | 1146.1119 | -4.57 | y_61_^6+^ |
| 1146.9393 | 1146.9438 | -3.95 | y_123_^12+^ |
| 1152.6089 | 1152.6137 | -4.19 | y_62_-H_2_O^6+^ |
| 1154.1892 | 1154.1965 | -6.35 | y_124_^12+^ |
| 1155.4439 | 1155.4489 | -4.30 | y_62_^6+^ |
| 1157.7714 | 1157.7731 | -1.44 | a_62_^6+^ |
| 1160.1757 | 1160.1827 | -6.03 | y_20_^2+^ |
| 1163.2052 | 1163.2109 | -4.90 | b_51_^5+^ |
| 1165.4368 | 1165.4433 | -5.61 | b_62_^6+^ |
| 1166.3439 | 1166.3497 | -5.00 | y_115_^11+^ |
| 1172.1232 | 1172.1269 | -3.19 | y_63_^6+^ |
| 1174.5311 | 1174.5383 | -6.10 | y_126_^12+^ |
| 1179.2763 | 1179.2802 | -3.28 | b_63_-NH_3_^6+^ |
| 1180.4031 | 1180.4104 | -6.18 | b_106_^10+^ |
| 1182.1116 | 1182.1180 | -5.39 | b_63_^6+^ |
| 1184.3505 | 1184.3570 | -5.53 | y_117_-H_2_O^11+^ |
| 1185.7123 | 1185.7216 | -7.86 | y_117_^11+^ |
| 1188.9624 | 1188.9682 | -4.91 | y_64_^6+^ |
| 1191.4998 | 1191.5063 | -5.44 | a_96_^9+^ |
| 1191.6113 | 1191.6188 | -6.29 | b_107_^10+^ |
| 1195.6208 | 1195.6249 | -3.43 | b_64_^6+^ |
| 1197.6344 | 1197.6360 | -1.37 | y_118_^11+^ |
| 1198.0222 | 1198.0297 | -6.26 | b_53_^5+^ |
| 1201.5233 | 1201.5246 | -1.08 | b_108_-NH_3_^10+^ |
| 1203.9539 | 1203.9601 | -5.12 | a_97_^9+^ |
| 1208.2932 | 1208.2996 | -5.32 | b_65_^6+^ |
| 1212.6122 | 1212.6190 | -5.61 | b_87_^8+^ |
| 1213.6416 | 1213.6463 | -3.87 | y_65_^6+^ |
| 1216.4073 | 1216.4121 | -3.93 | a_98_-NH_3_^9+^ |
| 1217.6884 | 1217.6962 | -6.41 | y_21_^2+^ |
| 1225.8583 | 1225.8602 | -1.51 | a_99_-NH_3_^9+^ |
| 1232.9330 | 1232.9384 | -4.38 | b_111_-NH_3_^10+^ |
| 1234.8348 | 1234.8410 | -5.02 | b_111_^10+^ |
| 1246.1991 | 1246.2069 | -6.26 | y_22_^2+^ |
| 1251.3071 | 1251.3138 | -5.38 | b_67_^3+^ |
| 1252.0500 | 1252.0576 | -6.07 | b_56_^5+^ |
| 1254.2829 | 1254.2887 | -4.62 | y_56_^5+^ |
| 1260.3986 | 1260.4062 | -6.03 | b_45_^4+^ |
| 1262.2935 | 1262.3012 | -6.10 | b_33_^3+^ |
| 1271.8636 | 1271.8712 | -5.98 | b_57_^5+^ |
| 1289.8163 | 1289.8225 | -4.78 | b_69_^6+^ |
| 1294.4824 | 1294.4881 | -4.40 | b_58_^5+^ |
| 1303.2203 | 1303.2283 | -6.14 | y_23_^2+^ |
| 1320.0918 | 1320.0998 | -6.06 | b_59_^5+^ |
| 1340.1905 | 1340.1927 | -1.64 | a_96_^8+^ |
| 1343.1000 | 1343.1084 | -6.25 | b_60_^5+^ |
| 1354.4481 | 1354.4542 | -4.50 | a_97_^8+^ |
| 1359.7623 | 1359.7704 | -5.96 | y_24_^2+^ |
| 1368.7094 | 1368.7169 | -5.48 | b_61_^5+^ |
| 1382.9269 | 1382.9351 | -5.93 | y_62_-H_2_O^5+^ |
| 1386.3293 | 1386.3372 | -5.70 | y_62_^5+^ |
| 1398.1219 | 1398.1306 | -6.22 | b_62_^5+^ |
| 1414.7300 | 1414.7348 | -3.39 | b_63_-NH_3_^5+^ |
| 1418.3321 | 1418.3401 | -5.64 | b_63_^5+^ |
| 1434.5449 | 1434.5485 | -2.51 | b_64_-NH_3_^5+^ |
| 1438.1508 | 1438.1538 | -2.09 | b_64_^5+^ |

# S.I. Table 6: Fragments observed following CID of +15 ions with *m/z* 1084.80 from healthy tissue (identified as FABP_TA_).

| Measured (*m/z*) | Theoretical (*m/z*) | Δppm | Assignment |
| --- | --- | --- | --- |
| 477.2368 | 477.2400 | -6.71 | b_8_^2+^ |
| 573.8734 | 573.8771 | -6.45 | y_9_^2+^ |
| 584.7817 | 584.7853 | -6.16 | b_10_^2+^ |
| 620.6969 | 620.7011 | -6.77 | y_16_-H_2_O^3+^ |
| 630.4150 | 630.4192 | -6.66 | y_10_^2+^ |
| 687.9281 | 687.9326 | -6.54 | y_11_^2+^ |
| 692.3934 | 692.3980 | -6.64 | y_18_-H_2_O^3+^ |
| 698.3968 | 698.4015 | -6.73 | y_18_^3+^ |
| 712.3254 | 712.3301 | -6.60 | b_6_^1+^ |
| 716.4387 | 716.4434 | -6.56 | y_12_^2+^ |
| 730.0879 | 730.0927 | -6.57 | y_19_^3+^ |
| 736.0911 | 736.0962 | -6.93 | y_19_^3+^ |
| 772.9803 | 772.9854 | -6.60 | y_13_^2+^ |
| 773.7861 | 773.7909 | -6.20 | y_20_^3+^ |
| 823.5039 | 823.5092 | -6.44 | y_14_^2+^ |
| 831.1341 | 831.1403 | -7.46 | y_22_^2+^ |
| 840.3834 | 840.3886 | -6.19 | b_7_^1+^ |
| 869.1488 | 869.1547 | -6.79 | y_23_^3+^ |
| 879.0963 | 879.1028 | -7.39 | y_38_^5+^ |
| 889.0237 | 889.0295 | -6.52 | y_15_^2+^ |
| 930.5419 | 930.5480 | -6.56 | y_16_-H_2_O^2+^ |
| 939.5471 | 939.5533 | -6.60 | y_16_^2+^ |
| 953.4668 | 953.4727 | -6.19 | y_8_^1+^ |
| 985.2983 | 985.3040 | -5.79 | y_35_^4+^ |
| 987.5631 | 987.5695 | -6.48 | y_17_-H_2_O^2+^ |
| 996.5684 | 996.5748 | -6.42 | y_17_^2+^ |
| 1007.2651 | 1007.2725 | -7.35 | b_35_^4+^ |
| 1009.8155 | 1009.8211 | -5.55 | y_36_^4+^ |
| 1010.9526 | 1010.9555 | -2.83 | b_63_-NH_3_^7+^ |
| 1018.8932 | 1018.8996 | -6.25 | y_55_^6+^ |
| 1038.0865 | 1038.0933 | -6.55 | y_18_-H_2_O^2+^ |
| 1040.4000 | 1040.4067 | -6.41 | y_56_^6+^ |
| 1045.5732 | 1045.5796 | -6.12 | y_28_^3+^ |
| 1047.0918 | 1047.0986 | -6.49 | y_18_^2+^ |
| 1059.2478 | 1059.2541 | -5.92 | y_57_^6+^ |
| 1091.1647 | 1091.1709 | -5.68 | b_48_^5+^ |
| 1094.6284 | 1094.6354 | -6.39 | y_19_-H_2_O^2+^ |
| 1103.6335 | 1103.6406 | -6.43 | y_19_^2+^ |
| 1118.3942 | 1118.3982 | -3.61 | b_111_-NH_3_^11+^ |
| 1119.4195 | 1119.4248 | -4.76 | b_60_^6+^ |
| 1119.5984 | 1119.6030 | -4.14 | y_60_^6+^ |
| 1119.9413 | 1119.9461 | -4.32 | b_111_^11+^ |
| 1140.7582 | 1140.7653 | -6.19 | b_61_^6+^ |
| 1143.1029 | 1143.1088 | -5.16 | y_123_-H_2_O^12+^ |
| 1144.5203 | 1144.5263 | -5.21 | y_123_^12+^ |
| 1150.2724 | 1150.2804 | -6.95 | y_62_^6+^ |
| 1151.7724 | 1151.7790 | -5.70 | y_124_^12+^ |
| 1160.1750 | 1160.1827 | -6.64 | y_20_^2+^ |
| 1163.6046 | 1163.6072 | -2.23 | a_105_^5+^ |
| 1165.6043 | 1165.6100 | -4.89 | b_62_^6+^ |
| 1166.9515 | 1166.9585 | -5.97 | y_63_^6+^ |
| 1177.4013 | 1177.4093 | -6.79 | b_106_^10+^ |
| 1179.1082 | 1179.1135 | -4.49 | b_63_-NH_3_^6+^ |
| 1181.5303 | 1181.5379 | -6.46 | y_117_-H_2_O^11+^ |
| 1182.1109 | 1182.1180 | -5.98 | b_63_^6+^ |
| 1183.1681 | 1183.1752 | -6.03 | y_117_^11+^ |
| 1183.9601 | 1183.9664 | -5.35 | y_64_^6+^ |
| 1186.2120 | 1186.2183 | -5.31 | a_107_^10+^ |
| 1187.1088 | 1187.1151 | -5.31 | b_107_-NH_3_^10+^ |
| 1188.9096 | 1188.9177 | -6.81 | b_107_^10+^ |
| 1193.2638 | 1193.2704 | -5.57 | y_118_-H_2_O^11+^ |
| 1194.9012 | 1194.9078 | -5.56 | y_118_^11+^ |
| 1195.7870 | 1195.7916 | -3.82 | b_64_^6+^ |
| 1197.6204 | 1197.6279 | -6.26 | b_53_^5+^ |
| 1212.6108 | 1212.6190 | -6.76 | b_87_^8+^ |
| 1231.8308 | 1231.8400 | -7.47 | b_111_^10+^ |
| 1260.3979 | 1260.4062 | -6.59 | b_45_^4+^ |
| 1262.2927 | 1262.3012 | -6.73 | b_33_^3+^ |
| 1279.7738 | 1279.7830 | -7.19 | y_115_^10+^ |
| 1289.9810 | 1289.9891 | -6.30 | b_69_^6+^ |
| 1294.6811 | 1294.6881 | -5.41 | b_58_^5+^ |
| 1303.2194 | 1303.2283 | -6.83 | y_23_^2+^ |
| 1308.3406 | 1308.3429 | -1.79 | b_106_^9+^ |
| 1320.2931 | 1320.2998 | -5.07 | b_59_^5+^ |
| 1368.7149 | 1368.7169 | -1.46 | b_61_^5+^ |
| 1380.3269 | 1380.3351 | -5.94 | y_62_^5+^ |
| 1398.3225 | 1398.3306 | -5.79 | b_62_^5+^ |
| 1414.9289 | 1414.9348 | -4.17 | b_63_-NH_3_^5+^ |
| 1418.5317 | 1418.5401 | -5.92 | b_63_^5+^ |
| 1438.3462 | 1438.3538 | -5.28 | b_64_^5+^ |

# S.I. Table 7: Fragments observed following ETD of +16 ions with *m/z* 942.24 from NASH tissue (identified as FABP1).

| Measured (*m/z*) | Calcuated (*m/z*) | Δppm | Assignment |
| --- | --- | --- | --- |
| 566.2926 | 566.2933 | -1.24 | c_5_^1+^ |
| 729.3557 | 729.3566 | -1.23 | c_6_^1+^ |
| 806.7926 | 806.7936 | -1.24 | z_21_^3+^ |
| 815.4995 | 815.4999 | -0.49 | z_14_^2+^ |
| 823.5086 | 823.5092 | -0.73 | y_14_^2+^ |
| 825.8002 | 825.8008 | -0.73 | z_22_^3+^ |
| 857.4146 | 857.4152 | -0.70 | c_7_^1+^ |
| 863.8144 | 863.8151 | -0.81 | z_23_^3+^ |
| 881.0195 | 881.0201 | -0.68 | z_15_^2+^ |
| 901.2601 | 901.2611 | -1.11 | z_32_^4+^ |
| 901.8437 | 901.8457 | -2.22 | z_24_^3+.^ |
| 911.9820 | 911.9814 | 0.66 | c_48_^6+^ |
| 916.3101 | 916.3108 | -0.76 | z_41_^5+^ |
| 916.9255 | 916.9261 | -0.65 | c_15_^2+^ |
| 922.4772 | 922.4776 | -0.43 | c_32_^4+^ |
| 926.4851 | 926.4881 | -3.24 | c_41_^5+^ |
| 936.6598 | 936.6593 | 0.57 | c_49_^6+^ |
| 938.0276 | 938.0282 | -0.64 | z_33_^6+^ |
| 944.5235 | 944.5240 | -0.53 | z_25_^3+^ |
| 949.8633 | 949.8635 | -0.21 | y_25_^3+^ |
| 951.4841 | 951.4844 | -0.32 | c_33_^4+^ |
| 952.0999 | 952.0999 | 0.00 | c_52_^5+^ |
| 963.2893 | 963.2901 | -0.83 | z_34_^4+^ |
| 967.2933 | 967.2948 | -1.55 | y_34_^4+^ |
| 970.4984 | 970.4993 | -0.93 | c_8_^1+^ |
| 974.9085 | 974.9085 | 0.00 | c_43_^4+^ |
| 979.5049 | 979.5054 | -0.51 | c_34_^4+^ |
| 988.5514 | 988.5520 | -0.61 | z_35_^4+^ |
| 1000.5260 | 1000.5238 | 2.20 | a_35_^4+^ |
| 1011.5287 | 1011.5291 | -0.40 | c_35_^4+^ |
| 1016.5686 | 1016.5689 | -0.30 | y_27_^3+^ |
| 1025.0371 | 1025.0378 | -0.65 | c_55_^6+^ |
| 1039.1435 | 1039.1435 | 0.00 | c_46_^5+^ |
| 1040.2395 | 1040.2400 | -0.48 | z_28_^3+^ |
| 1041.5887 | 1041.5901 | -1.34 | z_37_^4+^ |
| 1045.5789 | 1045.5796 | -0.67 | y_28_^3+^ |
| 1050.5510 | 1050.5516 | -0.57 | c_37_^4+^ |
| 1053.3880 | 1053.3885 | -0.47 | y_47_^5+^ |
| 1068.5582 | 1068.5572 | 0.94 | c_47_^5+^ |
| 1072.3084 | 1072.3096 | -1.12 | c_38_^4+^ |
| 1082.9377 | 1082.9383 | -0.55 | z_29_^3+^ |
| 1094.1748 | 1094.1762 | -1.28 | c_48_^5+^ |
| 1123.7902 | 1123.7899 | 0.27 | c_49_^5+^ |
| 1132.8388 | 1132.8393 | -0.44 | c_40_^4+^ |
| 1145.1362 | 1145.1367 | -0.44 | z_41_^4+^ |
| 1158.6471 | 1158.6473 | -0.17 | z_31_^3+^ |
| 1159.6084 | 1159.6084 | 0.00 | c_41_^4+^ |
| 1164.3221 | 1164.3202 | 1.60 | y_31_^3+^ |
| 1166.6154 | 1166.6162 | -0.69 | c_51_^5+^ |
| 1167.9287 | 1167.9289 | -0.17 | c_30_^3+^ |
| 1187.2701 | 1187.2694 | 0.56 | c_31_^3+^ |
| 1189.6223 | 1189.6230 | -0.59 | c_42_^4+^ |
| 1201.6800 | 1201.6816 | -1.33 | z_32_^3+.^ |
| 1214.9638 | 1214.9606 | 2.61 | a_32_^3+^ |
| 1218.1333 | 1218.1318 | 1.23 | c_41_^4+.^ |
| 1229.8444 | 1229.8439 | 0.41 | c_55_^5+^ |
| 1229.9686 | 1229.9677 | 0.70 | c_32_^2+^ |
| 1232.3884 | 1232.3891 | -0.57 | c_44_^4+^ |
| 1250.7035 | 1250.7018 | 1.33 | z_33_^3+^ |
| 1254.1108 | 1254.1072 | 2.87 | a_22_^2+^ |
| 1284.3857 | 1284.3870 | -1.01 | z_34_^3+.^ |
| 1298.9276 | 1298.9276 | 0.00 | c_46_^4+^ |
| 1305.6717 | 1305.6714 | 0.23 | c_34_^3+^ |
| 1316.7358 | 1316.7338 | 1.52 | y_47_^4+^ |
| 1323.7074 | 1323.7051 | 1.74 | c_59_^5+^ |
| 1324.9443 | 1324.9393 | 3.77 | a_47_^4+^ |
| 1333.6969 | 1333.6959 | 0.72 | a_35_^3+^ |
| 1335.6949 | 1335.6947 | 0.15 | c_47_^4+^ |
| 1348.3696 | 1348.3697 | -0.07 | c_35_^3+^ |
| 1352.2643 | 1352.2649 | -0.44 | z_24_^2+.^ |
| 1367.4701 | 1367.4684 | 1.24 | c_48_^4+^ |
| 1388.7870 | 1388.7870 | 0.00 | z_37_^3+^ |
| 1404.7368 | 1404.7355 | 0.93 | c_49_^4+^ |
| 1408.0442 | 1408.0444 | -0.14 | y_126_^10+^ |
| 1412.5408 | 1412.5411 | -0.21 | a_63_^5+^ |
| 1416.7877 | 1416.7864 | 0.92 | z_25_^2+^ |
| 1429.7476 | 1429.7437 | 2.70 | c_38_^3+^ |
| 1442.6015 | 1442.5994 | 1.46 | z_78_^6+.^ |
| 1458.2708 | 1458.2685 | 1.58 | c_51_^4+^ |
| 1526.8494 | 1526.8491 | 0.20 | z_41_^3+.^ |
| 1537.0566 | 1537.0530 | 2.34 | c_55_^4+^ |
| 1558.8639 | 1558.8605 | 2.18 | z_28_^2+^ |
| 1564.4942 | 1564.4930 | 0.75 | y_126_^9+^ |
| 1586.1657 | 1586.1616 | 2.56 | c_42_^4+^ |
| 1624.1791 | 1624.1759 | 1.95 | c_43_^3+^ |
| 1643.1858 | 1643.1830 | 1.70 | c_44_^3+^ |
| 1760.1848 | 1760.1787 | 3.47 | y_126_^8+^ |

# S.I. Table 8: Fragments observed following ETD of +16 ions with *m/z* 940.24 from NASH tissue (identified as FABP_TA_).

| Measured (*m/z*) | Calculated (*m/z*) | Δppm | Assignment |
| --- | --- | --- | --- |
| 566.2923 | 566.2933 | -1.77 | c_5_^1+^ |
| 729.3552 | 729.3566 | -1.92 | c_6_^1+^ |
| 815.4987 | 815.4999 | -1.47 | z_14_^2+^ |
| 826.1347 | 826.1367 | -2.42 | z_22_^3+.^ |
| 857.4141 | 857.4152 | -1.28 | c_7_^1+^ |
| 863.8139 | 863.8151 | -1.39 | z_23_^3+^ |
| 869.2355 | 869.2373 | -2.07 | z_31_^4+^ |
| 901.2595 | 901.2611 | -1.78 | z^324+^ |
| 901.8432 | 901.8457 | -2.77 | z_24_^3+.^ |
| 910.3072 | 910.3087 | -1.65 | z_41_^5+^ |
| 912.1470 | 912.1481 | -1.17 | c_48_^6+^ |
| 916.9252 | 916.9261 | -0.98 | c_15_^2+^ |
| 922.7268 | 922.7276 | -0.87 | c_32_^4+^ |
| 939.5525 | 939.5533 | -0.85 | y_16_^2+^ |
| 944.5225 | 944.5240 | -1.59 | z_25_^3+^ |
| 952.0989 | 952.0999 | -1.05 | c_52_^5+^ |
| 956.0359 | 956.0394 | -3.66 | z_34_^4+.^ |
| 970.4984 | 970.4993 | -0.93 | c_8_^1+^ |
| 979.5044 | 979.5054 | -1.02 | c_34_^4+^ |
| 1011.5285 | 1011.5291 | -0.59 | c_35_^4+^ |
| 1025.0362 | 1025.0378 | -1.53 | c_55_^6+^ |
| 1039.3439 | 1039.3435 | 0.38 | c_46_^5+^ |
| 1040.2394 | 1040.2400 | -0.58 | z_28_^3+^ |
| 1044.3813 | 1044.3842 | -2.78 | z_47_^5+.^ |
| 1045.5785 | 1045.5796 | -1.05 | y_28_^3+^ |
| 1050.5537 | 1050.5516 | 2.00 | c_37_^4+^ |
| 1068.5621 | 1068.5572 | 4.59 | c_47_^5+^ |
| 1072.3072 | 1072.3096 | -2.24 | c_38_^4+^ |
| 1082.9376 | 1082.9383 | -0.65 | z_29_^3+^ |
| 1094.3759 | 1094.3762 | -0.27 | c_48_^5+^ |
| 1123.6280 | 1123.6306 | -2.31 | z_40_^4+.^ |
| 1123.7895 | 1123.7899 | -0.36 | c_49_^5+^ |
| 1135.9464 | 1135.9447 | 1.50 | c_47_^4+^ |
| 1137.6325 | 1137.6340 | -1.32 | z_41_^4+^ |
| 1158.6464 | 1158.6473 | -0.78 | z_31_^3+^ |
| 1164.3209 | 1164.3202 | 0.57 | y_31_^3+^ |
| 1166.6172 | 1166.6162 | 0.86 | c_51_^5+^ |
| 1189.6225 | 1189.6230 | -0.42 | c_42_^4+^ |
| 1201.6801 | 1201.6816 | -1.25 | z_32_^3+.^ |
| 1218.1369 | 1218.1318 | 4.19 | c_41_^4+.^ |
| 1229.8437 | 1229.8439 | -0.16 | c_55_^5+^ |
| 1229.9682 | 1229.9677 | 0.38 | c_32_^2+^ |
| 1232.6393 | 1232.6391 | 0.16 | c_44_^4+^ |
| 1250.7030 | 1250.7018 | 0.93 | z_33_^3+^ |
| 1274.3844 | 1274.3834 | 0.78 | z_34_^3+.^ |
| 1305.6709 | 1305.6714 | -0.38 | c_34_^3+^ |
| 1348.3695 | 1348.3697 | -0.15 | c_35_^3+^ |
| 1367.7199 | 1367.7184 | 1.10 | c_48_^4+^ |
| 1516.8444 | 1516.8443 | 0.07 | z_27_^3+.^ |
| 1537.0569 | 1537.0530 | 2.54 | c_55_^4+^ |
| 1586.1619 | 1586.1616 | 0.19 | c_42_^3+^ |
| 1643.1868 | 1643.1830 | 2.31 | c_44_^3+^ |
| 1725.3199 | 1725.3158 | 2.38 | z_78_^5+.^ |
| 1737.9724 | 1737.9674 | 2.88 | z_31_^2+^ |
