# Supplementary material for: Top-Down and Bottom-Up Identification of Proteins by Liquid Extraction Surface Analysis Mass Spectrometry of Healthy and Diseased Human Liver Tissue
Source: J Am Soc Mass Spectrom. 2014 Sep 3;25(11):1953–61. doi: 10.1007/s13361-014-0967-z (PMC4197381; doi:10.1007/s13361-014-0967-z)
Supplement: Supplementary file 4 — (ZIP 7870 kb) [file 13361_2014_967_MOESM4_ESM.zip › index.html]

Annotated spectra


## Annotated spectra of Water\_1\_MC3.msf

go to Peptides  
go to Search Summary  

### Peptides

|  |  |  |  |  |  |  |  |  |  |  |  |  |  |  |  |  |  |  |  |  |  |  |  |  |  |  |  |  |  |  |  |  |
| --- | --- | --- | --- | --- | --- | --- | --- | --- | --- | --- | --- | --- | --- | --- | --- | --- | --- | --- | --- | --- | --- | --- | --- | --- | --- | --- | --- | --- | --- | --- | --- | --- |
|  | | | | | | | | | | | | | | | | | | | | | | | | | | | | | | | | |
| Confidence Sequence Activation Type Modifications IonScore XCorr ΔScore Rank Search Engine Charge Precursor m/z [Da] ΔM [ppm] First Scan Last Scan Annotated Spectrum Peak List  | | | | | | | | | | | | | | | | | | | | | | | | | | | | | | | | |
|  | | | | | | | | | | | | | | | | | | | | | | | | | | | | | | | | |
|  | **O14773 - Tripeptidyl-peptidase 1 OS=Homo sapiens GN=TPP1 PE=1 SV=2 - [TPP1\_HUMAN]** | | | | | | | | | | | | | | | | | | | | | | | | | | | | | | |  |
|  | | | | | | | | | | | | | | | | | | | | | | | | | | | | | | | | |
|  | High |  | VPIPWVSGTSASTPVFGGILSLINEHR |  | CID |  |  |  | 78.57 |  |  |  | 1.00 |  | 1 |  | Mascot (2) |  | 3 |  | 945.51709 |  | 5.87 |  | 4088 |  | 4088 |  | Image |  | Peak List |  |
|  | | | | | | | | | | | | | | | | | | | | | | | | | | | | | | | | |
|  | High |  | VPIPWVSGTSASTPVFGGILSLINEHR |  | CID |  |  |  |  |  | 6.25 |  | 1.00 |  | 1 |  | SEQUEST (4) |  | 3 |  | 945.51709 |  | 5.87 |  | 4088 |  | 4088 |  | Image |  | Peak List |  |
|  | | | | | | | | | | | | | | | | | | | | | | | | | | | | | | | | |
|  | **O43708 - Maleylacetoacetate isomerase OS=Homo sapiens GN=GSTZ1 PE=1 SV=3 - [MAAI\_HUMAN]** | | | | | | | | | | | | | | | | | | | | | | | | | | | | | | |  |
|  | | | | | | | | | | | | | | | | | | | | | | | | | | | | | | | | |
|  | High |  | IDGITIHQSLAIIEYLEEMRPTPR |  | CID |  |  |  | 25.94 |  |  |  | 1.00 |  | 1 |  | Mascot (2) |  | 4 |  | 699.62677 |  | 3.25 |  | 3676 |  | 3676 |  | Image |  | Peak List |  |
|  | | | | | | | | | | | | | | | | | | | | | | | | | | | | | | | | |
|  | High |  | IDGITIHQSLAIIEYLEEMRPTPR |  | CID |  |  |  |  |  | 2.40 |  | 1.00 |  | 1 |  | SEQUEST (4) |  | 4 |  | 699.62677 |  | 3.25 |  | 3676 |  | 3676 |  | Image |  | Peak List |  |
|  | | | | | | | | | | | | | | | | | | | | | | | | | | | | | | | | |
|  | **O43795 - Unconventional myosin-Ib OS=Homo sapiens GN=MYO1B PE=1 SV=3 - [MYO1B\_HUMAN]** | | | | | | | | | | | | | | | | | | | | | | | | | | | | | | |  |
|  | | | | | | | | | | | | | | | | | | | | | | | | | | | | | | | | |
|  | High |  | NAMQIVGFMDHEAESVLAVVAAVLK |  | CID |  |  |  | 44.58 |  |  |  | 1.00 |  | 1 |  | Mascot (2) |  | 3 |  | 881.46576 |  | 5.49 |  | 5745 |  | 5745 |  | Image |  | Peak List |  |
|  | | | | | | | | | | | | | | | | | | | | | | | | | | | | | | | | |
|  | High |  | NAMQIVGFMDHEAESVLAVVAAVLK |  | CID |  |  |  |  |  | 4.07 |  | 1.00 |  | 1 |  | SEQUEST (4) |  | 3 |  | 881.46576 |  | 5.49 |  | 5745 |  | 5745 |  | Image |  | Peak List |  |
|  | | | | | | | | | | | | | | | | | | | | | | | | | | | | | | | | |
|  | **O60256 - Phosphoribosyl pyrophosphate synthase-associated protein 2 OS=Homo sapiens GN=PRPSAP2 PE=1 SV=1 - [KPRB\_HUMAN]** | | | | | | | | | | | | | | | | | | | | | | | | | | | | | | |  |
|  | | | | | | | | | | | | | | | | | | | | | | | | | | | | | | | | |
|  | High |  | IAIIVDDIIDDVDSFLAAAETLK |  | CID |  |  |  | 57.00 |  |  |  | 1.00 |  | 1 |  | Mascot (2) |  | 3 |  | 820.78064 |  | 6.20 |  | 6241 |  | 6241 |  | Image |  | Peak List |  |
|  | | | | | | | | | | | | | | | | | | | | | | | | | | | | | | | | |
|  | High |  | IAIIVDDIIDDVDSFLAAAETLK |  | CID |  |  |  |  |  | 5.56 |  | 1.00 |  | 1 |  | SEQUEST (4) |  | 3 |  | 820.78064 |  | 6.20 |  | 6241 |  | 6241 |  | Image |  | Peak List |  |
|  | | | | | | | | | | | | | | | | | | | | | | | | | | | | | | | | |
|  | **O60763 - General vesicular transport factor p115 OS=Homo sapiens GN=USO1 PE=1 SV=2 - [USO1\_HUMAN]** | | | | | | | | | | | | | | | | | | | | | | | | | | | | | | |  |
|  | | | | | | | | | | | | | | | | | | | | | | | | | | | | | | | | |
|  | High |  | LLDIISEEGNSDGGIVVEDCLILLQNLLK |  | CID |  |  |  | 54.23 |  |  |  | 1.00 |  | 1 |  | Mascot (2) |  | 3 |  | 1042.56812 |  | 7.88 |  | 6410 |  | 6410 |  | Image |  | Peak List |  |
|  | | | | | | | | | | | | | | | | | | | | | | | | | | | | | | | | |
|  | High |  | LLDIISEEGNSDGGIVVEDCLILLQNLLK |  | CID |  |  |  |  |  | 5.14 |  | 1.00 |  | 1 |  | SEQUEST (4) |  | 3 |  | 1042.56763 |  | 7.41 |  | 6427 |  | 6427 |  | Image |  | Peak List |  |
|  | | | | | | | | | | | | | | | | | | | | | | | | | | | | | | | | |
|  | **O75191 - Xylulose kinase OS=Homo sapiens GN=XYLB PE=1 SV=3 - [XYLB\_HUMAN]** | | | | | | | | | | | | | | | | | | | | | | | | | | | | | | |  |
|  | | | | | | | | | | | | | | | | | | | | | | | | | | | | | | | | |
|  | High |  | DGLTVTSPVLMWVQALDIILEK |  | CID |  |  |  | 23.40 |  |  |  | 1.00 |  | 1 |  | Mascot (2) |  | 2 |  | 1221.18054 |  | 7.24 |  | 6267 |  | 6267 |  | Image |  | Peak List |  |
|  | | | | | | | | | | | | | | | | | | | | | | | | | | | | | | | | |
|  | High |  | DGLTVTSPVLMWVQALDIILEK |  | CID |  |  |  |  |  | 3.97 |  | 1.00 |  | 1 |  | SEQUEST (4) |  | 2 |  | 1221.18054 |  | 7.24 |  | 6267 |  | 6267 |  | Image |  | Peak List |  |
|  | | | | | | | | | | | | | | | | | | | | | | | | | | | | | | | | |
|  | **O75356 - Ectonucleoside triphosphate diphosphohydrolase 5 OS=Homo sapiens GN=ENTPD5 PE=1 SV=1 - [ENTP5\_HUMAN]** | | | | | | | | | | | | | | | | | | | | | | | | | | | | | | |  |
|  | | | | | | | | | | | | | | | | | | | | | | | | | | | | | | | | |
|  | High |  | MPGQLPILEGEVFDSVKPGLSAFVDQPK |  | CID |  |  |  | 47.00 |  |  |  | 1.00 |  | 1 |  | Mascot (2) |  | 3 |  | 1000.19434 |  | 2.96 |  | 3701 |  | 3701 |  | Image |  | Peak List |  |
|  | | | | | | | | | | | | | | | | | | | | | | | | | | | | | | | | |
|  | High |  | MPGQLPILEGEVFDSVKPGLSAFVDQPK |  | CID |  |  |  |  |  | 4.48 |  | 1.00 |  | 1 |  | SEQUEST (4) |  | 3 |  | 1000.19434 |  | 2.96 |  | 3701 |  | 3701 |  | Image |  | Peak List |  |
|  | | | | | | | | | | | | | | | | | | | | | | | | | | | | | | | | |
|  | **P00441 - Superoxide dismutase [Cu-Zn] OS=Homo sapiens GN=SOD1 PE=1 SV=2 - [SODC\_HUMAN]** | | | | | | | | | | | | | | | | | | | | | | | | | | | | | | |  |
|  | | | | | | | | | | | | | | | | | | | | | | | | | | | | | | | | |
|  | High |  | HVGDLGNVTADKDGVADVSIEDSVISLSGDHCIIGR |  | CID |  |  |  | 54.72 |  |  |  | 1.00 |  | 1 |  | Mascot (2) |  | 4 |  | 916.70660 |  | 3.41 |  | 2752 |  | 2752 |  | Image |  | Peak List |  |
|  | | | | | | | | | | | | | | | | | | | | | | | | | | | | | | | | |
|  | High |  | HVGDLGNVTADKDGVADVSIEDSVISLSGDHCIIGR |  | CID |  |  |  |  |  | 4.80 |  | 1.00 |  | 1 |  | SEQUEST (4) |  | 4 |  | 916.71094 |  | 8.14 |  | 2748 |  | 2748 |  | Image |  | Peak List |  |
|  | | | | | | | | | | | | | | | | | | | | | | | | | | | | | | | | |
|  | **P00915 - Carbonic anhydrase 1 OS=Homo sapiens GN=CA1 PE=1 SV=2 - [CAH1\_HUMAN]** | | | | | | | | | | | | | | | | | | | | | | | | | | | | | | |  |
|  | | | | | | | | | | | | | | | | | | | | | | | | | | | | | | | | |
|  | High |  | LYPIANGNNQSPVDIK |  | CID |  |  |  | 25.93 |  |  |  | 1.00 |  | 1 |  | Mascot (2) |  | 2 |  | 871.95850 |  | 1.42 |  | 1782 |  | 1782 |  | Image |  | Peak List |  |
|  | | | | | | | | | | | | | | | | | | | | | | | | | | | | | | | | |
|  | High |  | LYPIANGNNQSPVDIK |  | CID |  |  |  |  |  | 3.83 |  | 1.00 |  | 1 |  | SEQUEST (4) |  | 2 |  | 871.95850 |  | 1.42 |  | 1782 |  | 1782 |  | Image |  | Peak List |  |
|  | | | | | | | | | | | | | | | | | | | | | | | | | | | | | | | | |
|  | **P00918 - Carbonic anhydrase 2 OS=Homo sapiens GN=CA2 PE=1 SV=2 - [CAH2\_HUMAN]** | | | | | | | | | | | | | | | | | | | | | | | | | | | | | | |  |
|  | | | | | | | | | | | | | | | | | | | | | | | | | | | | | | | | |
|  | High |  | AVQQPDGLAVLGIFLK |  | CID |  |  |  | 56.87 |  |  |  | 1.00 |  | 1 |  | Mascot (2) |  | 2 |  | 834.99170 |  | 4.62 |  | 3947 |  | 3947 |  | Image |  | Peak List |  |
|  | | | | | | | | | | | | | | | | | | | | | | | | | | | | | | | | |
|  | High |  | AVQQPDGLAVLGIFLK |  | CID |  |  |  |  |  | 4.04 |  | 1.00 |  | 1 |  | SEQUEST (4) |  | 2 |  | 834.99170 |  | 4.62 |  | 3947 |  | 3947 |  | Image |  | Peak List |  |
|  | | | | | | | | | | | | | | | | | | | | | | | | | | | | | | | | |
|  | **P02763 - Alpha-1-acid glycoprotein 1 OS=Homo sapiens GN=ORM1 PE=1 SV=1 - [A1AG1\_HUMAN]** | | | | | | | | | | | | | | | | | | | | | | | | | | | | | | |  |
|  | | | | | | | | | | | | | | | | | | | | | | | | | | | | | | | | |
|  | High |  | TYMLAFDVNDEKNWGLSVYADKPETTK |  | CID |  |  |  | 68.92 |  |  |  | 1.00 |  | 1 |  | Mascot (2) |  | 3 |  | 1045.84290 |  | 5.12 |  | 2709 |  | 2709 |  | Image |  | Peak List |  |
|  | | | | | | | | | | | | | | | | | | | | | | | | | | | | | | | | |
|  | High |  | TYMLAFDVNDEKNWGLSVYADKPETTK |  | CID |  |  |  |  |  | 5.66 |  | 1.00 |  | 1 |  | SEQUEST (4) |  | 3 |  | 1045.84290 |  | 5.12 |  | 2709 |  | 2709 |  | Image |  | Peak List |  |
|  | | | | | | | | | | | | | | | | | | | | | | | | | | | | | | | | |
|  | **P06133 - UDP-glucuronosyltransferase 2B4 OS=Homo sapiens GN=UGT2B4 PE=1 SV=2 - [UD2B4\_HUMAN]** | | | | | | | | | | | | | | | | | | | | | | | | | | | | | | |  |
|  | | | | | | | | | | | | | | | | | | | | | | | | | | | | | | | | |
|  | High |  | FDVVLADAVFPFGELLAELLK |  | CID |  |  |  | 21.59 |  |  |  | 1.00 |  | 1 |  | Mascot (2) |  | 3 |  | 769.43030 |  | 3.45 |  | 6258 |  | 6258 |  | Image |  | Peak List |  |
|  | | | | | | | | | | | | | | | | | | | | | | | | | | | | | | | | |
|  | High |  | FDVVLADAVFPFGELLAELLK |  | CID |  |  |  |  |  | 2.91 |  | 1.00 |  | 1 |  | SEQUEST (4) |  | 3 |  | 769.43030 |  | 3.45 |  | 6258 |  | 6258 |  | Image |  | Peak List |  |
|  | | | | | | | | | | | | | | | | | | | | | | | | | | | | | | | | |
|  | **P07108 - Acyl-CoA-binding protein OS=Homo sapiens GN=DBI PE=1 SV=2 - [ACBP\_HUMAN]** | | | | | | | | | | | | | | | | | | | | | | | | | | | | | | |  |
|  | | | | | | | | | | | | | | | | | | | | | | | | | | | | | | | | |
|  | High |  | QATVGDINTERPGMLDFTGK |  | CID |  |  |  | 48.57 |  |  |  | 1.00 |  | 1 |  | Mascot (2) |  | 3 |  | 717.35889 |  | 3.42 |  | 1907 |  | 1907 |  | Image |  | Peak List |  |
|  | | | | | | | | | | | | | | | | | | | | | | | | | | | | | | | | |
|  | High |  | QATVGDINTERPGMLDFTGK |  | CID |  |  |  |  |  | 1.69 |  | 1.00 |  | 1 |  | SEQUEST (4) |  | 3 |  | 717.35889 |  | 3.42 |  | 1907 |  | 1907 |  | Image |  | Peak List |  |
|  | | | | | | | | | | | | | | | | | | | | | | | | | | | | | | | | |
|  | **P08684 - Cytochrome P450 3A4 OS=Homo sapiens GN=CYP3A4 PE=1 SV=4 - [CP3A4\_HUMAN]** | | | | | | | | | | | | | | | | | | | | | | | | | | | | | | |  |
|  | | | | | | | | | | | | | | | | | | | | | | | | | | | | | | | | |
|  | High |  | ALIPDLAMETWLLLAVSLVLLYLYGTHSHGLFK |  | CID |  |  |  | 75.56 |  |  |  | 1.00 |  | 1 |  | Mascot (2) |  | 4 |  | 925.27350 |  | 7.84 |  | 6600 |  | 6600 |  | Image |  | Peak List |  |
|  | | | | | | | | | | | | | | | | | | | | | | | | | | | | | | | | |
|  | **P09467 - Fructose-1,6-bisphosphatase 1 OS=Homo sapiens GN=FBP1 PE=1 SV=5 - [F16P1\_HUMAN]** | | | | | | | | | | | | | | | | | | | | | | | | | | | | | | |  |
|  | | | | | | | | | | | | | | | | | | | | | | | | | | | | | | | | |
|  | High |  | APVILGSPDDVLEFLK |  | CID |  |  |  | 72.63 |  |  |  | 1.00 |  | 1 |  | Mascot (2) |  | 2 |  | 856.98016 |  | 3.53 |  | 3834 |  | 3834 |  | Image |  | Peak List |  |
|  | | | | | | | | | | | | | | | | | | | | | | | | | | | | | | | | |
|  | High |  | APVILGSPDDVLEFLK |  | CID |  |  |  |  |  | 4.32 |  | 1.00 |  | 1 |  | SEQUEST (4) |  | 2 |  | 856.98016 |  | 3.53 |  | 3834 |  | 3834 |  | Image |  | Peak List |  |
|  | | | | | | | | | | | | | | | | | | | | | | | | | | | | | | | | |
|  | **P0C0L5;Q6U2E9 - Complement C4-B OS=Homo sapiens GN=C4B PE=1 SV=2 - [CO4B\_HUMAN]** | | | | | | | | | | | | | | | | | | | | | | | | | | | | | | |  |
|  | | | | | | | | | | | | | | | | | | | | | | | | | | | | | | | | |
|  | High |  | NPSDPMPQAPALWIETTAYALLHLLLHEGK |  | CID |  |  |  | 31.99 |  |  |  | 1.00 |  | 1 |  | Mascot (2) |  | 4 |  | 832.44214 |  | 6.73 |  | 5369 |  | 5369 |  | Image |  | Peak List |  |
|  | | | | | | | | | | | | | | | | | | | | | | | | | | | | | | | | |
|  | High |  | NPSDPMPQAPALWIETTAYALLHLLLHEGK |  | CID |  |  |  |  |  | 3.05 |  | 1.00 |  | 1 |  | SEQUEST (4) |  | 4 |  | 832.44214 |  | 6.73 |  | 5369 |  | 5369 |  | Image |  | Peak List |  |
|  | | | | | | | | | | | | | | | | | | | | | | | | | | | | | | | | |
|  | **P10620 - Microsomal glutathione S-transferase 1 OS=Homo sapiens GN=MGST1 PE=1 SV=1 - [MGST1\_HUMAN]** | | | | | | | | | | | | | | | | | | | | | | | | | | | | | | |  |
|  | | | | | | | | | | | | | | | | | | | | | | | | | | | | | | | | |
|  | High |  | VDLTQVMDDEVFMAFASYATIILSK |  | CID |  |  |  | 57.32 |  |  |  | 1.00 |  | 1 |  | Mascot (2) |  | 3 |  | 936.47394 |  | 6.75 |  | 6232 |  | 6232 |  | Image |  | Peak List |  |
|  | | | | | | | | | | | | | | | | | | | | | | | | | | | | | | | | |
|  | **P10632 - Cytochrome P450 2C8 OS=Homo sapiens GN=CYP2C8 PE=1 SV=2 - [CP2C8\_HUMAN]** | | | | | | | | | | | | | | | | | | | | | | | | | | | | | | |  |
|  | | | | | | | | | | | | | | | | | | | | | | | | | | | | | | | | |
|  | High |  | SEFNIENLVGTVADLFVAGTETTSTTLR |  | CID |  |  |  | 42.37 |  |  |  | 1.00 |  | 1 |  | Mascot (2) |  | 3 |  | 995.84656 |  | 6.65 |  | 5657 |  | 5657 |  | Image |  | Peak List |  |
|  | | | | | | | | | | | | | | | | | | | | | | | | | | | | | | | | |
|  | High |  | SEFNIENLVGTVADLFVAGTETTSTTLR |  | CID |  |  |  |  |  | 4.65 |  | 1.00 |  | 1 |  | SEQUEST (4) |  | 3 |  | 995.84656 |  | 6.65 |  | 5657 |  | 5657 |  | Image |  | Peak List |  |
|  | | | | | | | | | | | | | | | | | | | | | | | | | | | | | | | | |
|  | **P11182 - Lipoamide acyltransferase component of branched-chain alpha-keto acid dehydrogenase complex, mitochondrial OS=Homo sapiens GN=DBT PE=1 SV=3 - [ODB2\_HUMAN]** | | | | | | | | | | | | | | | | | | | | | | | | | | | | | | |  |
|  | | | | | | | | | | | | | | | | | | | | | | | | | | | | | | | | |
|  | High |  | SYLENPAFMLLDLK |  | CID |  |  |  | 66.06 |  |  |  | 1.00 |  | 1 |  | Mascot (2) |  | 2 |  | 827.43579 |  | 5.18 |  | 3875 |  | 3875 |  | Image |  | Peak List |  |
|  | | | | | | | | | | | | | | | | | | | | | | | | | | | | | | | | |
|  | High |  | SYLENPAFMLLDLK |  | CID |  |  |  |  |  | 4.18 |  | 1.00 |  | 1 |  | SEQUEST (4) |  | 2 |  | 827.43579 |  | 5.18 |  | 3875 |  | 3875 |  | Image |  | Peak List |  |
|  | | | | | | | | | | | | | | | | | | | | | | | | | | | | | | | | |
|  | **P13010 - X-ray repair cross-complementing protein 5 OS=Homo sapiens GN=XRCC5 PE=1 SV=3 - [XRCC5\_HUMAN]** | | | | | | | | | | | | | | | | | | | | | | | | | | | | | | |  |
|  | | | | | | | | | | | | | | | | | | | | | | | | | | | | | | | | |
|  | High |  | DDEAAAVALSSLIHALDDLDMVAIVR |  | CID |  |  |  | 82.52 |  |  |  | 1.00 |  | 1 |  | Mascot (2) |  | 3 |  | 908.47321 |  | 4.73 |  | 5561 |  | 5561 |  | Image |  | Peak List |  |
|  | | | | | | | | | | | | | | | | | | | | | | | | | | | | | | | | |
|  | High |  | DDEAAAVALSSLIHALDDLDMVAIVR |  | CID |  |  |  |  |  | 5.85 |  | 1.00 |  | 1 |  | SEQUEST (4) |  | 3 |  | 908.47321 |  | 4.73 |  | 5561 |  | 5561 |  | Image |  | Peak List |  |
|  | | | | | | | | | | | | | | | | | | | | | | | | | | | | | | | | |
|  | **P14866 - Heterogeneous nuclear ribonucleoprotein L OS=Homo sapiens GN=HNRNPL PE=1 SV=2 - [HNRPL\_HUMAN]** | | | | | | | | | | | | | | | | | | | | | | | | | | | | | | |  |
|  | | | | | | | | | | | | | | | | | | | | | | | | | | | | | | | | |
|  | High |  | GLIDGVVEADLVEALQEFGPISYVVVMPK |  | CID |  |  |  | 29.34 |  |  |  | 1.00 |  | 1 |  | Mascot (2) |  | 3 |  | 1029.89124 |  | 8.66 |  | 5945 |  | 5945 |  | Image |  | Peak List |  |
|  | | | | | | | | | | | | | | | | | | | | | | | | | | | | | | | | |
|  | High |  | GLIDGVVEADLVEALQEFGPISYVVVMPK |  | CID |  |  |  |  |  | 6.00 |  | 1.00 |  | 1 |  | SEQUEST (4) |  | 3 |  | 1029.89124 |  | 8.66 |  | 5945 |  | 5945 |  | Image |  | Peak List |  |
|  | | | | | | | | | | | | | | | | | | | | | | | | | | | | | | | | |
|  | **P15428 - 15-hydroxyprostaglandin dehydrogenase [NAD(+)] OS=Homo sapiens GN=HPGD PE=1 SV=1 - [PGDH\_HUMAN]** | | | | | | | | | | | | | | | | | | | | | | | | | | | | | | |  |
|  | | | | | | | | | | | | | | | | | | | | | | | | | | | | | | | | |
|  | High |  | YYGILDPPLIANGLITLIEDDALNGAIMK |  | CID |  |  |  | 50.12 |  |  |  | 1.00 |  | 1 |  | Mascot (2) |  | 3 |  | 1039.56494 |  | 6.84 |  | 5721 |  | 5721 |  | Image |  | Peak List |  |
|  | | | | | | | | | | | | | | | | | | | | | | | | | | | | | | | | |
|  | High |  | YYGILDPPLIANGLITLIEDDALNGAIMK |  | CID |  |  |  |  |  | 5.34 |  | 1.00 |  | 1 |  | SEQUEST (4) |  | 3 |  | 1039.56421 |  | 6.14 |  | 5707 |  | 5707 |  | Image |  | Peak List |  |
|  | | | | | | | | | | | | | | | | | | | | | | | | | | | | | | | | |
|  | **P18124 - 60S ribosomal protein L7 OS=Homo sapiens GN=RPL7 PE=1 SV=1 - [RL7\_HUMAN]** | | | | | | | | | | | | | | | | | | | | | | | | | | | | | | |  |
|  | | | | | | | | | | | | | | | | | | | | | | | | | | | | | | | | |
|  | High |  | YGIICMEDLIHEIYTVGK |  | CID |  |  |  | 33.15 |  |  |  | 1.00 |  | 1 |  | Mascot (2) |  | 3 |  | 699.68719 |  | 3.55 |  | 3964 |  | 3964 |  | Image |  | Peak List |  |
|  | | | | | | | | | | | | | | | | | | | | | | | | | | | | | | | | |
|  | High |  | YGIICMEDLIHEIYTVGK |  | CID |  |  |  |  |  | 2.77 |  | 1.00 |  | 1 |  | SEQUEST (4) |  | 3 |  | 699.68719 |  | 3.55 |  | 3964 |  | 3964 |  | Image |  | Peak List |  |
|  | | | | | | | | | | | | | | | | | | | | | | | | | | | | | | | | |
|  | **P21399 - Cytoplasmic aconitate hydratase OS=Homo sapiens GN=ACO1 PE=1 SV=3 - [ACOC\_HUMAN]** | | | | | | | | | | | | | | | | | | | | | | | | | | | | | | |  |
|  | | | | | | | | | | | | | | | | | | | | | | | | | | | | | | | | |
|  | High |  | ANYLASPPLVIAYAIAGTIR |  | CID |  |  |  | 74.89 |  |  |  | 1.00 |  | 1 |  | Mascot (2) |  | 2 |  | 1037.59534 |  | 6.64 |  | 4494 |  | 4494 |  | Image |  | Peak List |  |
|  | | | | | | | | | | | | | | | | | | | | | | | | | | | | | | | | |
|  | High |  | ANYLASPPLVIAYAIAGTIR |  | CID |  |  |  |  |  | 4.47 |  | 1.00 |  | 1 |  | SEQUEST (4) |  | 2 |  | 1037.59534 |  | 6.64 |  | 4494 |  | 4494 |  | Image |  | Peak List |  |
|  | | | | | | | | | | | | | | | | | | | | | | | | | | | | | | | | |
|  | **P21695 - Glycerol-3-phosphate dehydrogenase [NAD(+)], cytoplasmic OS=Homo sapiens GN=GPD1 PE=1 SV=4 - [GPDA\_HUMAN]** | | | | | | | | | | | | | | | | | | | | | | | | | | | | | | |  |
|  | | | | | | | | | | | | | | | | | | | | | | | | | | | | | | | | |
|  | High |  | LPPNVVAVPDVVQAAEDADILIFVVPHQFIGK |  | CID |  |  |  | 47.06 |  |  |  | 1.00 |  | 1 |  | Mascot (2) |  | 3 |  | 1137.63721 |  | 7.23 |  | 4889 |  | 4889 |  | Image |  | Peak List |  |
|  | | | | | | | | | | | | | | | | | | | | | | | | | | | | | | | | |
|  | High |  | LPPNVVAVPDVVQAAEDADILIFVVPHQFIGK |  | CID |  |  |  |  |  | 4.75 |  | 1.00 |  | 1 |  | SEQUEST (4) |  | 3 |  | 1137.63721 |  | 7.23 |  | 4889 |  | 4889 |  | Image |  | Peak List |  |
|  | | | | | | | | | | | | | | | | | | | | | | | | | | | | | | | | |
|  | **P22033 - Methylmalonyl-CoA mutase, mitochondrial OS=Homo sapiens GN=MUT PE=1 SV=4 - [MUTA\_HUMAN]** | | | | | | | | | | | | | | | | | | | | | | | | | | | | | | |  |
|  | | | | | | | | | | | | | | | | | | | | | | | | | | | | | | | | |
|  | High |  | FNSISISGYHMQEAGADAILELAYTLADGLEYSR |  | CID |  |  |  | 40.72 |  |  |  | 1.00 |  | 1 |  | Mascot (2) |  | 3 |  | 1235.93616 |  | 5.30 |  | 4966 |  | 4966 |  | Image |  | Peak List |  |
|  | | | | | | | | | | | | | | | | | | | | | | | | | | | | | | | | |
|  | High |  | FNSISISGYHMQEAGADAILELAYTLADGLEYSR |  | CID |  |  |  |  |  | 4.94 |  | 1.00 |  | 1 |  | SEQUEST (4) |  | 3 |  | 1235.93616 |  | 5.30 |  | 4966 |  | 4966 |  | Image |  | Peak List |  |
|  | | | | | | | | | | | | | | | | | | | | | | | | | | | | | | | | |
|  | **P22314 - Ubiquitin-like modifier-activating enzyme 1 OS=Homo sapiens GN=UBA1 PE=1 SV=3 - [UBA1\_HUMAN]** | | | | | | | | | | | | | | | | | | | | | | | | | | | | | | |  |
|  | | | | | | | | | | | | | | | | | | | | | | | | | | | | | | | | |
|  | High |  | IIPAIATTTAAVVGLVCLELYK |  | CID |  |  |  | 29.70 |  |  |  | 1.00 |  | 1 |  | Mascot (2) |  | 3 |  | 753.77692 |  | 5.68 |  | 5522 |  | 5522 |  | Image |  | Peak List |  |
|  | | | | | | | | | | | | | | | | | | | | | | | | | | | | | | | | |
|  | High |  | IIPAIATTTAAVVGLVCLELYK |  | CID |  |  |  |  |  | 3.74 |  | 1.00 |  | 1 |  | SEQUEST (4) |  | 3 |  | 753.77716 |  | 6.01 |  | 5502 |  | 5502 |  | Image |  | Peak List |  |
|  | | | | | | | | | | | | | | | | | | | | | | | | | | | | | | | | |
|  | **P30085 - UMP-CMP kinase OS=Homo sapiens GN=CMPK1 PE=1 SV=3 - [KCY\_HUMAN]** | | | | | | | | | | | | | | | | | | | | | | | | | | | | | | |  |
|  | | | | | | | | | | | | | | | | | | | | | | | | | | | | | | | | |
|  | High |  | FLIDGFPR |  | CID |  |  |  | 28.66 |  |  |  | 1.00 |  | 1 |  | Mascot (2) |  | 2 |  | 482.76733 |  | 2.34 |  | 2614 |  | 2614 |  | Image |  | Peak List |  |
|  | | | | | | | | | | | | | | | | | | | | | | | | | | | | | | | | |
|  | High |  | FLIDGFPR |  | CID |  |  |  |  |  | 2.65 |  | 1.00 |  | 1 |  | SEQUEST (4) |  | 2 |  | 482.76733 |  | 2.34 |  | 2614 |  | 2614 |  | Image |  | Peak List |  |
|  | | | | | | | | | | | | | | | | | | | | | | | | | | | | | | | | |
|  | **P30711 - Glutathione S-transferase theta-1 OS=Homo sapiens GN=GSTT1 PE=1 SV=4 - [GSTT1\_HUMAN]** | | | | | | | | | | | | | | | | | | | | | | | | | | | | | | |  |
|  | | | | | | | | | | | | | | | | | | | | | | | | | | | | | | | | |
|  | High |  | VMFPVFLGEPVSPQTLAATLAELDVTLQLLEDK |  | CID |  |  |  | 32.36 |  |  |  | 1.00 |  | 1 |  | Mascot (2) |  | 3 |  | 1195.65320 |  | 7.71 |  | 6032 |  | 6032 |  | Image |  | Peak List |  |
|  | | | | | | | | | | | | | | | | | | | | | | | | | | | | | | | | |
|  | High |  | VMFPVFLGEPVSPQTLAATLAELDVTLQLLEDK |  | CID |  |  |  |  |  | 4.87 |  | 1.00 |  | 1 |  | SEQUEST (4) |  | 3 |  | 1195.65320 |  | 7.71 |  | 6032 |  | 6032 |  | Image |  | Peak List |  |
|  | | | | | | | | | | | | | | | | | | | | | | | | | | | | | | | | |
|  | **P35270 - Sepiapterin reductase OS=Homo sapiens GN=SPR PE=1 SV=1 - [SPRE\_HUMAN]** | | | | | | | | | | | | | | | | | | | | | | | | | | | | | | |  |
|  | | | | | | | | | | | | | | | | | | | | | | | | | | | | | | | | |
|  | High |  | VPADLGAEAGLQQLLGALR |  | CID |  |  |  | 25.79 |  |  |  | 1.00 |  | 1 |  | Mascot (2) |  | 3 |  | 631.36005 |  | 2.90 |  | 3966 |  | 3966 |  | Image |  | Peak List |  |
|  | | | | | | | | | | | | | | | | | | | | | | | | | | | | | | | | |
|  | High |  | VPADLGAEAGLQQLLGALR |  | CID |  |  |  |  |  | 3.33 |  | 1.00 |  | 1 |  | SEQUEST (4) |  | 3 |  | 631.36005 |  | 2.90 |  | 3966 |  | 3966 |  | Image |  | Peak List |  |
|  | | | | | | | | | | | | | | | | | | | | | | | | | | | | | | | | |
|  | **P35754 - Glutaredoxin-1 OS=Homo sapiens GN=GLRX PE=1 SV=2 - [GLRX1\_HUMAN]** | | | | | | | | | | | | | | | | | | | | | | | | | | | | | | |  |
|  | | | | | | | | | | | | | | | | | | | | | | | | | | | | | | | | |
|  | High |  | QGLLEFVDITATNHTNEIQDYLQQLTGAR |  | CID |  |  |  | 89.04 |  |  |  | 1.00 |  | 1 |  | Mascot (2) |  | 3 |  | 1096.89404 |  | 5.46 |  | 4169 |  | 4169 |  | Image |  | Peak List |  |
|  | | | | | | | | | | | | | | | | | | | | | | | | | | | | | | | | |
|  | High |  | QGLLEFVDITATNHTNEIQDYLQQLTGAR |  | CID |  |  |  |  |  | 5.43 |  | 1.00 |  | 1 |  | SEQUEST (4) |  | 3 |  | 1096.89404 |  | 5.46 |  | 4169 |  | 4169 |  | Image |  | Peak List |  |
|  | | | | | | | | | | | | | | | | | | | | | | | | | | | | | | | | |
|  | **P40939 - Trifunctional enzyme subunit alpha, mitochondrial OS=Homo sapiens GN=HADHA PE=1 SV=2 - [ECHA\_HUMAN]** | | | | | | | | | | | | | | | | | | | | | | | | | | | | | | |  |
|  | | | | | | | | | | | | | | | | | | | | | | | | | | | | | | | | |
|  | High |  | MGLVDQLVEPLGPGLKPPEER |  | CID |  |  |  | 43.38 |  |  |  | 1.00 |  | 1 |  | Mascot (2) |  | 3 |  | 758.74634 |  | 3.56 |  | 2875 |  | 2875 |  | Image |  | Peak List |  |
|  | | | | | | | | | | | | | | | | | | | | | | | | | | | | | | | | |
|  | High |  | MGLVDQLVEPLGPGLKPPEER |  | CID |  |  |  |  |  | 2.67 |  | 1.00 |  | 1 |  | SEQUEST (4) |  | 3 |  | 758.74634 |  | 3.56 |  | 2875 |  | 2875 |  | Image |  | Peak List |  |
|  | | | | | | | | | | | | | | | | | | | | | | | | | | | | | | | | |
|  | **P46952 - 3-hydroxyanthranilate 3,4-dioxygenase OS=Homo sapiens GN=HAAO PE=1 SV=2 - [3HAO\_HUMAN]** | | | | | | | | | | | | | | | | | | | | | | | | | | | | | | |  |
|  | | | | | | | | | | | | | | | | | | | | | | | | | | | | | | | | |
|  | High |  | DLGTQLAPIIQEFFSSEQYR |  | CID |  |  |  | 39.10 |  |  |  | 1.00 |  | 1 |  | Mascot (2) |  | 3 |  | 781.39984 |  | 7.93 |  | 5040 |  | 5040 |  | Image |  | Peak List |  |
|  | | | | | | | | | | | | | | | | | | | | | | | | | | | | | | | | |
|  | High |  | DLGTQLAPIIQEFFSSEQYR |  | CID |  |  |  |  |  | 4.69 |  | 1.00 |  | 1 |  | SEQUEST (4) |  | 3 |  | 781.39984 |  | 7.93 |  | 5040 |  | 5040 |  | Image |  | Peak List |  |
|  | | | | | | | | | | | | | | | | | | | | | | | | | | | | | | | | |
|  | **P47756 - F-actin-capping protein subunit beta OS=Homo sapiens GN=CAPZB PE=1 SV=4 - [CAPZB\_HUMAN]** | | | | | | | | | | | | | | | | | | | | | | | | | | | | | | |  |
|  | | | | | | | | | | | | | | | | | | | | | | | | | | | | | | | | |
|  | High |  | NLSDLIDLVPSLCEDLLSSVDQPLK |  | CID |  |  |  | 31.28 |  |  |  | 1.00 |  | 1 |  | Mascot (2) |  | 3 |  | 909.48364 |  | 7.12 |  | 6457 |  | 6457 |  | Image |  | Peak List |  |
|  | | | | | | | | | | | | | | | | | | | | | | | | | | | | | | | | |
|  | High |  | NLSDLIDLVPSLCEDLLSSVDQPLK |  | CID |  |  |  |  |  | 3.76 |  | 1.00 |  | 1 |  | SEQUEST (4) |  | 3 |  | 909.48364 |  | 7.12 |  | 6457 |  | 6457 |  | Image |  | Peak List |  |
|  | | | | | | | | | | | | | | | | | | | | | | | | | | | | | | | | |
|  | **P48163 - NADP-dependent malic enzyme OS=Homo sapiens GN=ME1 PE=1 SV=1 - [MAOX\_HUMAN]** | | | | | | | | | | | | | | | | | | | | | | | | | | | | | | |  |
|  | | | | | | | | | | | | | | | | | | | | | | | | | | | | | | | | |
|  | High |  | LSDQTILFQGAGEAALGIAHLIVMALEK |  | CID |  |  |  | 69.05 |  |  |  | 1.00 |  | 1 |  | Mascot (2) |  | 3 |  | 970.53674 |  | 5.16 |  | 5656 |  | 5656 |  | Image |  | Peak List |  |
|  | | | | | | | | | | | | | | | | | | | | | | | | | | | | | | | | |
|  | High |  | LSDQTILFQGAGEAALGIAHLIVMALEK |  | CID |  |  |  |  |  | 4.25 |  | 1.00 |  | 1 |  | SEQUEST (4) |  | 3 |  | 970.53674 |  | 5.16 |  | 5656 |  | 5656 |  | Image |  | Peak List |  |
|  | | | | | | | | | | | | | | | | | | | | | | | | | | | | | | | | |
|  | **P48643 - T-complex protein 1 subunit epsilon OS=Homo sapiens GN=CCT5 PE=1 SV=1 - [TCPE\_HUMAN]** | | | | | | | | | | | | | | | | | | | | | | | | | | | | | | |  |
|  | | | | | | | | | | | | | | | | | | | | | | | | | | | | | | | | |
|  | High |  | SQDDEIGDGTTGVVVLAGALLEEAEQLLDR |  | CID |  |  |  | 36.66 |  |  |  | 1.00 |  | 1 |  | Mascot (2) |  | 3 |  | 1038.52832 |  | 6.99 |  | 6465 |  | 6465 |  | Image |  | Peak List |  |
|  | | | | | | | | | | | | | | | | | | | | | | | | | | | | | | | | |
|  | High |  | SQDDEIGDGTTGVVVLAGALLEEAEQLLDR |  | CID |  |  |  |  |  | 4.66 |  | 1.00 |  | 1 |  | SEQUEST (4) |  | 3 |  | 1038.52832 |  | 6.99 |  | 6465 |  | 6465 |  | Image |  | Peak List |  |
|  | | | | | | | | | | | | | | | | | | | | | | | | | | | | | | | | |
|  | **P49189 - 4-trimethylaminobutyraldehyde dehydrogenase OS=Homo sapiens GN=ALDH9A1 PE=1 SV=3 - [AL9A1\_HUMAN]** | | | | | | | | | | | | | | | | | | | | | | | | | | | | | | |  |
|  | | | | | | | | | | | | | | | | | | | | | | | | | | | | | | | | |
|  | High |  | SPLIIFSDCDMNNAVK |  | CID |  |  |  | 67.11 |  |  |  | 1.00 |  | 1 |  | Mascot (2) |  | 2 |  | 883.92865 |  | 2.70 |  | 2971 |  | 2971 |  | Image |  | Peak List |  |
|  | | | | | | | | | | | | | | | | | | | | | | | | | | | | | | | | |
|  | High |  | SPLIIFSDCDMNNAVK |  | CID |  |  |  |  |  | 4.54 |  | 1.00 |  | 1 |  | SEQUEST (4) |  | 2 |  | 883.92865 |  | 2.70 |  | 2971 |  | 2971 |  | Image |  | Peak List |  |
|  | | | | | | | | | | | | | | | | | | | | | | | | | | | | | | | | |
|  | **P49368 - T-complex protein 1 subunit gamma OS=Homo sapiens GN=CCT3 PE=1 SV=4 - [TCPG\_HUMAN]** | | | | | | | | | | | | | | | | | | | | | | | | | | | | | | |  |
|  | | | | | | | | | | | | | | | | | | | | | | | | | | | | | | | | |
|  | High |  | TQDEEVGDGTTSVIILAGEMLSVAEHFLEQQMHPTVVISAYR |  | CID |  |  |  | 56.97 |  |  |  | 1.00 |  | 1 |  | Mascot (2) |  | 4 |  | 1151.07739 |  | 7.33 |  | 5720 |  | 5720 |  | Image |  | Peak List |  |
|  | | | | | | | | | | | | | | | | | | | | | | | | | | | | | | | | |
|  | High |  | TQDEEVGDGTTSVIILAGEMLSVAEHFLEQQMHPTVVISAYR |  | CID |  |  |  |  |  | 4.59 |  | 1.00 |  | 1 |  | SEQUEST (4) |  | 4 |  | 1151.07739 |  | 7.33 |  | 5720 |  | 5720 |  | Image |  | Peak List |  |
|  | | | | | | | | | | | | | | | | | | | | | | | | | | | | | | | | |
|  | **P49588 - Alanine--tRNA ligase, cytoplasmic OS=Homo sapiens GN=AARS PE=1 SV=2 - [SYAC\_HUMAN]** | | | | | | | | | | | | | | | | | | | | | | | | | | | | | | |  |
|  | | | | | | | | | | | | | | | | | | | | | | | | | | | | | | | | |
|  | High |  | GFFATLVDVVVQSLGDAFPELK |  | CID |  |  |  | 25.25 |  |  |  | 1.00 |  | 1 |  | Mascot (2) |  | 3 |  | 784.75922 |  | 6.12 |  | 6496 |  | 6496 |  | Image |  | Peak List |  |
|  | | | | | | | | | | | | | | | | | | | | | | | | | | | | | | | | |
|  | High |  | GFFATLVDVVVQSLGDAFPELK |  | CID |  |  |  |  |  | 4.48 |  | 1.00 |  | 1 |  | SEQUEST (4) |  | 3 |  | 784.75922 |  | 6.12 |  | 6496 |  | 6496 |  | Image |  | Peak List |  |
|  | | | | | | | | | | | | | | | | | | | | | | | | | | | | | | | | |
|  | **P50213 - Isocitrate dehydrogenase [NAD] subunit alpha, mitochondrial OS=Homo sapiens GN=IDH3A PE=1 SV=1 - [IDH3A\_HUMAN]** | | | | | | | | | | | | | | | | | | | | | | | | | | | | | | |  |
|  | | | | | | | | | | | | | | | | | | | | | | | | | | | | | | | | |
|  | High |  | DMANPTALLLSAVMMLR |  | CID |  |  |  | 61.37 |  |  |  | 1.00 |  | 1 |  | Mascot (2) |  | 3 |  | 616.32788 |  | 5.53 |  | 5499 |  | 5499 |  | Image |  | Peak List |  |
|  | | | | | | | | | | | | | | | | | | | | | | | | | | | | | | | | |
|  | High |  | DMANPTALLLSAVMMLR |  | CID |  |  |  |  |  | 4.90 |  | 1.00 |  | 1 |  | SEQUEST (4) |  | 3 |  | 616.32733 |  | 4.63 |  | 5526 |  | 5526 |  | Image |  | Peak List |  |
|  | | | | | | | | | | | | | | | | | | | | | | | | | | | | | | | | |
|  | **P51570 - Galactokinase OS=Homo sapiens GN=GALK1 PE=1 SV=1 - [GALK1\_HUMAN]** | | | | | | | | | | | | | | | | | | | | | | | | | | | | | | |  |
|  | | | | | | | | | | | | | | | | | | | | | | | | | | | | | | | | |
|  | High |  | VNLIGEHTDYNQGLVLPMALELMTVLVGSPR |  | CID |  |  |  | 38.39 |  |  |  | 1.00 |  | 1 |  | Mascot (2) |  | 3 |  | 1127.27222 |  | 7.90 |  | 5001 |  | 5001 |  | Image |  | Peak List |  |
|  | | | | | | | | | | | | | | | | | | | | | | | | | | | | | | | | |
|  | High |  | VNLIGEHTDYNQGLVLPMALELMTVLVGSPR |  | CID |  |  |  |  |  | 4.13 |  | 1.00 |  | 1 |  | SEQUEST (4) |  | 3 |  | 1127.27222 |  | 7.90 |  | 5001 |  | 5001 |  | Image |  | Peak List |  |
|  | | | | | | | | | | | | | | | | | | | | | | | | | | | | | | | | |
|  | **P51649 - Succinate-semialdehyde dehydrogenase, mitochondrial OS=Homo sapiens GN=ALDH5A1 PE=1 SV=2 - [SSDH\_HUMAN]** | | | | | | | | | | | | | | | | | | | | | | | | | | | | | | |  |
|  | | | | | | | | | | | | | | | | | | | | | | | | | | | | | | | | |
|  | High |  | VSMELGGLAPFIVFDSANVDQAVAGAMASK |  | CID |  |  |  | 24.61 |  |  |  | 1.00 |  | 1 |  | Mascot (2) |  | 3 |  | 999.17407 |  | 5.72 |  | 4483 |  | 4483 |  | Image |  | Peak List |  |
|  | | | | | | | | | | | | | | | | | | | | | | | | | | | | | | | | |
|  | High |  | VSMELGGLAPFIVFDSANVDQAVAGAMASK |  | CID |  |  |  |  |  | 3.97 |  | 1.00 |  | 1 |  | SEQUEST (4) |  | 3 |  | 999.17407 |  | 5.72 |  | 4483 |  | 4483 |  | Image |  | Peak List |  |
|  | | | | | | | | | | | | | | | | | | | | | | | | | | | | | | | | |
|  | **P54819 - Adenylate kinase 2, mitochondrial OS=Homo sapiens GN=AK2 PE=1 SV=2 - [KAD2\_HUMAN]** | | | | | | | | | | | | | | | | | | | | | | | | | | | | | | |  |
|  | | | | | | | | | | | | | | | | | | | | | | | | | | | | | | | | |
|  | High |  | NGFLLDGFPR |  | CID |  |  |  | 66.51 |  |  |  | 1.00 |  | 1 |  | Mascot (2) |  | 2 |  | 568.29926 |  | 1.50 |  | 2731 |  | 2731 |  | Image |  | Peak List |  |
|  | | | | | | | | | | | | | | | | | | | | | | | | | | | | | | | | |
|  | High |  | NGFLLDGFPR |  | CID |  |  |  |  |  | 2.79 |  | 1.00 |  | 1 |  | SEQUEST (4) |  | 2 |  | 568.29926 |  | 1.50 |  | 2731 |  | 2731 |  | Image |  | Peak List |  |
|  | | | | | | | | | | | | | | | | | | | | | | | | | | | | | | | | |
|  | **P55263 - Adenosine kinase OS=Homo sapiens GN=ADK PE=1 SV=2 - [ADK\_HUMAN]** | | | | | | | | | | | | | | | | | | | | | | | | | | | | | | |  |
|  | | | | | | | | | | | | | | | | | | | | | | | | | | | | | | | | |
|  | High |  | ENILFGMGNPLLDISAVVDKDFLDK |  | CID |  |  |  | 26.57 |  |  |  | 1.00 |  | 1 |  | Mascot (2) |  | 3 |  | 921.81927 |  | 5.71 |  | 4786 |  | 4786 |  | Image |  | Peak List |  |
|  | | | | | | | | | | | | | | | | | | | | | | | | | | | | | | | | |
|  | High |  | ENILFGMGNPLLDISAVVDKDFLDK |  | CID |  |  |  |  |  | 2.77 |  | 1.00 |  | 1 |  | SEQUEST (4) |  | 3 |  | 921.81842 |  | 4.78 |  | 4803 |  | 4803 |  | Image |  | Peak List |  |
|  | | | | | | | | | | | | | | | | | | | | | | | | | | | | | | | | |
|  | **P61158 - Actin-related protein 3 OS=Homo sapiens GN=ACTR3 PE=1 SV=3 - [ARP3\_HUMAN]** | | | | | | | | | | | | | | | | | | | | | | | | | | | | | | |  |
|  | | | | | | | | | | | | | | | | | | | | | | | | | | | | | | | | |
|  | High |  | FLGPEIFFHPEFANPDFTQPISEVVDEVIQNCPIDVR |  | CID |  |  |  | 27.10 |  |  |  | 1.00 |  | 1 |  | Mascot (2) |  | 3 |  | 1420.37622 |  | 8.10 |  | 5401 |  | 5401 |  | Image |  | Peak List |  |
|  | | | | | | | | | | | | | | | | | | | | | | | | | | | | | | | | |
|  | High |  | FLGPEIFFHPEFANPDFTQPISEVVDEVIQNCPIDVR |  | CID |  |  |  |  |  | 4.06 |  | 1.00 |  | 1 |  | SEQUEST (4) |  | 3 |  | 1420.37622 |  | 8.10 |  | 5401 |  | 5401 |  | Image |  | Peak List |  |
|  | | | | | | | | | | | | | | | | | | | | | | | | | | | | | | | | |
|  | **P61457 - Pterin-4-alpha-carbinolamine dehydratase OS=Homo sapiens GN=PCBD1 PE=1 SV=2 - [PHS\_HUMAN]** | | | | | | | | | | | | | | | | | | | | | | | | | | | | | | |  |
|  | | | | | | | | | | | | | | | | | | | | | | | | | | | | | | | | |
|  | High |  | DINLASFIEQVAVSMT |  | CID |  |  |  | 21.24 |  |  |  | 1.00 |  | 1 |  | Mascot (2) |  | 2 |  | 869.44543 |  | 6.18 |  | 6641 |  | 6641 |  | Image |  | Peak List |  |
|  | | | | | | | | | | | | | | | | | | | | | | | | | | | | | | | | |
|  | High |  | DINLASFIEQVAVSMT |  | CID |  |  |  |  |  | 2.41 |  | 1.00 |  | 1 |  | SEQUEST (4) |  | 2 |  | 869.44543 |  | 6.18 |  | 6641 |  | 6641 |  | Image |  | Peak List |  |
|  | | | | | | | | | | | | | | | | | | | | | | | | | | | | | | | | |
|  | **P62841 - 40S ribosomal protein S15 OS=Homo sapiens GN=RPS15 PE=1 SV=2 - [RS15\_HUMAN]** | | | | | | | | | | | | | | | | | | | | | | | | | | | | | | |  |
|  | | | | | | | | | | | | | | | | | | | | | | | | | | | | | | | | |
|  | High |  | GVDLDQLLDMSYEQLMQLYSAR |  | CID |  |  |  | 54.06 |  |  |  | 1.00 |  | 1 |  | Mascot (2) |  | 3 |  | 863.42175 |  | 5.21 |  | 5122 |  | 5122 |  | Image |  | Peak List |  |
|  | | | | | | | | | | | | | | | | | | | | | | | | | | | | | | | | |
|  | High |  | GVDLDQLLDMSYEQLMQLYSAR |  | CID |  |  |  |  |  | 5.40 |  | 1.00 |  | 1 |  | SEQUEST (4) |  | 3 |  | 863.42175 |  | 5.21 |  | 5122 |  | 5122 |  | Image |  | Peak List |  |
|  | | | | | | | | | | | | | | | | | | | | | | | | | | | | | | | | |
|  | **P62906 - 60S ribosomal protein L10a OS=Homo sapiens GN=RPL10A PE=1 SV=2 - [RL10A\_HUMAN]** | | | | | | | | | | | | | | | | | | | | | | | | | | | | | | |  |
|  | | | | | | | | | | | | | | | | | | | | | | | | | | | | | | | | |
|  | High |  | MTDDELVYNIHLAVNFLVSLLK |  | CID |  |  |  | 35.14 |  |  |  | 1.00 |  | 1 |  | Mascot (2) |  | 3 |  | 849.79462 |  | 6.46 |  | 5965 |  | 5965 |  | Image |  | Peak List |  |
|  | | | | | | | | | | | | | | | | | | | | | | | | | | | | | | | | |
|  | High |  | MTDDELVYNIHLAVNFLVSLLK |  | CID |  |  |  |  |  | 3.03 |  | 1.00 |  | 1 |  | SEQUEST (4) |  | 3 |  | 849.79462 |  | 6.46 |  | 5965 |  | 5965 |  | Image |  | Peak List |  |
|  | | | | | | | | | | | | | | | | | | | | | | | | | | | | | | | | |
|  | **P78417 - Glutathione S-transferase omega-1 OS=Homo sapiens GN=GSTO1 PE=1 SV=2 - [GSTO1\_HUMAN]** | | | | | | | | | | | | | | | | | | | | | | | | | | | | | | |  |
|  | | | | | | | | | | | | | | | | | | | | | | | | | | | | | | | | |
|  | High |  | MILELFSK |  | CID |  |  |  | 27.45 |  |  |  | 1.00 |  | 1 |  | Mascot (2) |  | 2 |  | 490.77908 |  | 2.36 |  | 2796 |  | 2796 |  | Image |  | Peak List |  |
|  | | | | | | | | | | | | | | | | | | | | | | | | | | | | | | | | |
|  | High |  | MILELFSK |  | CID |  |  |  |  |  | 2.07 |  | 1.00 |  | 1 |  | SEQUEST (4) |  | 2 |  | 490.77908 |  | 2.36 |  | 2796 |  | 2796 |  | Image |  | Peak List |  |
|  | | | | | | | | | | | | | | | | | | | | | | | | | | | | | | | | |
|  | **P84243 - Histone H3.3 OS=Homo sapiens GN=H3F3A PE=1 SV=2 - [H33\_HUMAN]** | | | | | | | | | | | | | | | | | | | | | | | | | | | | | | |  |
|  | | | | | | | | | | | | | | | | | | | | | | | | | | | | | | | | |
|  | High |  | FQSAAIGALQEASEAYLVGLFEDTNLCAIHAK |  | CID |  |  |  | 40.68 |  |  |  | 1.00 |  | 1 |  | Mascot (2) |  | 3 |  | 1127.57605 |  | 8.97 |  | 4672 |  | 4672 |  | Image |  | Peak List |  |
|  | | | | | | | | | | | | | | | | | | | | | | | | | | | | | | | | |
|  | High |  | FQSAAIGALQEASEAYLVGLFEDTNLCAIHAK |  | CID |  |  |  |  |  | 5.10 |  | 1.00 |  | 1 |  | SEQUEST (4) |  | 3 |  | 1127.57605 |  | 8.97 |  | 4672 |  | 4672 |  | Image |  | Peak List |  |
|  | | | | | | | | | | | | | | | | | | | | | | | | | | | | | | | | |
|  | **Q04446 - 1,4-alpha-glucan-branching enzyme OS=Homo sapiens GN=GBE1 PE=1 SV=3 - [GLGB\_HUMAN]** | | | | | | | | | | | | | | | | | | | | | | | | | | | | | | |  |
|  | | | | | | | | | | | | | | | | | | | | | | | | | | | | | | | | |
|  | High |  | YGTPEELQELVDTAHSMGIIVLLDVVHSHASK |  | CID |  |  |  | 37.56 |  |  |  | 1.00 |  | 1 |  | Mascot (2) |  | 5 |  | 698.56372 |  | 4.69 |  | 4291 |  | 4291 |  | Image |  | Peak List |  |
|  | | | | | | | | | | | | | | | | | | | | | | | | | | | | | | | | |
|  | High |  | YGTPEELQELVDTAHSMGIIVLLDVVHSHASK |  | CID |  |  |  |  |  | 3.39 |  | 1.00 |  | 1 |  | SEQUEST (4) |  | 5 |  | 698.56372 |  | 4.69 |  | 4291 |  | 4291 |  | Image |  | Peak List |  |
|  | | | | | | | | | | | | | | | | | | | | | | | | | | | | | | | | |
|  | **Q06323 - Proteasome activator complex subunit 1 OS=Homo sapiens GN=PSME1 PE=1 SV=1 - [PSME1\_HUMAN]** | | | | | | | | | | | | | | | | | | | | | | | | | | | | | | |  |
|  | | | | | | | | | | | | | | | | | | | | | | | | | | | | | | | | |
|  | High |  | DVIEQLNLVTTWLQLQIPR |  | CID |  |  |  | 55.74 |  |  |  | 1.00 |  | 1 |  | Mascot (2) |  | 3 |  | 760.43384 |  | 4.86 |  | 5420 |  | 5420 |  | Image |  | Peak List |  |
|  | | | | | | | | | | | | | | | | | | | | | | | | | | | | | | | | |
|  | High |  | DVIEQLNLVTTWLQLQIPR |  | CID |  |  |  |  |  | 4.60 |  | 1.00 |  | 1 |  | SEQUEST (4) |  | 3 |  | 760.43384 |  | 4.86 |  | 5420 |  | 5420 |  | Image |  | Peak List |  |
|  | | | | | | | | | | | | | | | | | | | | | | | | | | | | | | | | |
|  | **Q10713 - Mitochondrial-processing peptidase subunit alpha OS=Homo sapiens GN=PMPCA PE=1 SV=2 - [MPPA\_HUMAN]** | | | | | | | | | | | | | | | | | | | | | | | | | | | | | | |  |
|  | | | | | | | | | | | | | | | | | | | | | | | | | | | | | | | | |
|  | High |  | GLDTVVALLADVVLQPR |  | CID |  |  |  | 45.10 |  |  |  | 1.00 |  | 1 |  | Mascot (2) |  | 3 |  | 593.68689 |  | 4.81 |  | 5381 |  | 5381 |  | Image |  | Peak List |  |
|  | | | | | | | | | | | | | | | | | | | | | | | | | | | | | | | | |
|  | High |  | GLDTVVALLADVVLQPR |  | CID |  |  |  |  |  | 4.43 |  | 1.00 |  | 1 |  | SEQUEST (4) |  | 3 |  | 593.68689 |  | 4.81 |  | 5381 |  | 5381 |  | Image |  | Peak List |  |
|  | | | | | | | | | | | | | | | | | | | | | | | | | | | | | | | | |
|  | **Q13200 - 26S proteasome non-ATPase regulatory subunit 2 OS=Homo sapiens GN=PSMD2 PE=1 SV=3 - [PSMD2\_HUMAN]** | | | | | | | | | | | | | | | | | | | | | | | | | | | | | | |  |
|  | | | | | | | | | | | | | | | | | | | | | | | | | | | | | | | | |
|  | High |  | GEAIEAILAALEVVSEPFR |  | CID |  |  |  | 32.67 |  |  |  | 1.00 |  | 1 |  | Mascot (2) |  | 3 |  | 672.03748 |  | 6.07 |  | 5469 |  | 5469 |  | Image |  | Peak List |  |
|  | | | | | | | | | | | | | | | | | | | | | | | | | | | | | | | | |
|  | High |  | GEAIEAILAALEVVSEPFR |  | CID |  |  |  |  |  | 4.53 |  | 1.00 |  | 1 |  | SEQUEST (4) |  | 3 |  | 672.03748 |  | 6.07 |  | 5469 |  | 5469 |  | Image |  | Peak List |  |
|  | | | | | | | | | | | | | | | | | | | | | | | | | | | | | | | | |
|  | **Q13451 - Peptidyl-prolyl cis-trans isomerase FKBP5 OS=Homo sapiens GN=FKBP5 PE=1 SV=2 - [FKBP5\_HUMAN]** | | | | | | | | | | | | | | | | | | | | | | | | | | | | | | |  |
|  | | | | | | | | | | | | | | | | | | | | | | | | | | | | | | | | |
|  | High |  | IPSNATLFFEIELLDFKGEDLFEDGGIIR |  | CID |  |  |  | 24.93 |  |  |  | 1.00 |  | 1 |  | Mascot (2) |  | 3 |  | 1100.24268 |  | 7.61 |  | 4461 |  | 4461 |  | Image |  | Peak List |  |
|  | | | | | | | | | | | | | | | | | | | | | | | | | | | | | | | | |
|  | High |  | IPSNATLFFEIELLDFKGEDLFEDGGIIR |  | CID |  |  |  |  |  | 2.97 |  | 1.00 |  | 1 |  | SEQUEST (4) |  | 3 |  | 1100.24268 |  | 7.61 |  | 4461 |  | 4461 |  | Image |  | Peak List |  |
|  | | | | | | | | | | | | | | | | | | | | | | | | | | | | | | | | |
|  | **Q14558 - Phosphoribosyl pyrophosphate synthase-associated protein 1 OS=Homo sapiens GN=PRPSAP1 PE=1 SV=2 - [KPRA\_HUMAN]** | | | | | | | | | | | | | | | | | | | | | | | | | | | | | | |  |
|  | | | | | | | | | | | | | | | | | | | | | | | | | | | | | | | | |
|  | High |  | IAIIVDDIIDDVESFVAAAEILK |  | CID |  |  |  | 56.16 |  |  |  | 1.00 |  | 1 |  | Mascot (2) |  | 3 |  | 824.79163 |  | 4.79 |  | 6428 |  | 6428 |  | Image |  | Peak List |  |
|  | | | | | | | | | | | | | | | | | | | | | | | | | | | | | | | | |
|  | High |  | IAIIVDDIIDDVESFVAAAEILK |  | CID |  |  |  |  |  | 6.14 |  | 1.00 |  | 1 |  | SEQUEST (4) |  | 3 |  | 824.79163 |  | 4.79 |  | 6428 |  | 6428 |  | Image |  | Peak List |  |
|  | | | | | | | | | | | | | | | | | | | | | | | | | | | | | | | | |
|  | **Q14974 - Importin subunit beta-1 OS=Homo sapiens GN=KPNB1 PE=1 SV=2 - [IMB1\_HUMAN]** | | | | | | | | | | | | | | | | | | | | | | | | | | | | | | |  |
|  | | | | | | | | | | | | | | | | | | | | | | | | | | | | | | | | |
|  | High |  | MFQSTAGSGGVQEDALMAVSTLVEVLGGEFLK |  | CID |  |  |  | 33.20 |  |  |  | 1.00 |  | 1 |  | Mascot (2) |  | 3 |  | 1091.22302 |  | 9.73 |  | 6107 |  | 6107 |  | Image |  | Peak List |  |
|  | | | | | | | | | | | | | | | | | | | | | | | | | | | | | | | | |
|  | High |  | MFQSTAGSGGVQEDALMAVSTLVEVLGGEFLK |  | CID |  |  |  |  |  | 4.07 |  | 1.00 |  | 1 |  | SEQUEST (4) |  | 3 |  | 1091.22302 |  | 9.73 |  | 6107 |  | 6107 |  | Image |  | Peak List |  |
|  | | | | | | | | | | | | | | | | | | | | | | | | | | | | | | | | |
|  | **Q15181 - Inorganic pyrophosphatase OS=Homo sapiens GN=PPA1 PE=1 SV=2 - [IPYR\_HUMAN]** | | | | | | | | | | | | | | | | | | | | | | | | | | | | | | |  |
|  | | | | | | | | | | | | | | | | | | | | | | | | | | | | | | | | |
|  | High |  | VLGILAMIDEGETDWK |  | CID |  |  |  | 82.46 |  |  |  | 1.00 |  | 1 |  | Mascot (2) |  | 2 |  | 895.45923 |  | 3.92 |  | 3950 |  | 3950 |  | Image |  | Peak List |  |
|  | | | | | | | | | | | | | | | | | | | | | | | | | | | | | | | | |
|  | High |  | VLGILAMIDEGETDWK |  | CID |  |  |  |  |  | 4.67 |  | 1.00 |  | 1 |  | SEQUEST (4) |  | 2 |  | 895.45923 |  | 3.92 |  | 3950 |  | 3950 |  | Image |  | Peak List |  |
|  | | | | | | | | | | | | | | | | | | | | | | | | | | | | | | | | |
|  | **Q3LXA3 - Bifunctional ATP-dependent dihydroxyacetone kinase/FAD-AMP lyase (cyclizing) OS=Homo sapiens GN=DAK PE=1 SV=2 - [DHAK\_HUMAN]** | | | | | | | | | | | | | | | | | | | | | | | | | | | | | | |  |
|  | | | | | | | | | | | | | | | | | | | | | | | | | | | | | | | | |
|  | High |  | EGPPPASPAQLLSK |  | CID |  |  |  | 30.87 |  |  |  | 1.00 |  | 1 |  | Mascot (2) |  | 2 |  | 696.38153 |  | 2.01 |  | 1702 |  | 1702 |  | Image |  | Peak List |  |
|  | | | | | | | | | | | | | | | | | | | | | | | | | | | | | | | | |
|  | High |  | EGPPPASPAQLLSK |  | CID |  |  |  |  |  | 2.72 |  | 1.00 |  | 1 |  | SEQUEST (4) |  | 2 |  | 696.38153 |  | 2.01 |  | 1702 |  | 1702 |  | Image |  | Peak List |  |
|  | | | | | | | | | | | | | | | | | | | | | | | | | | | | | | | | |
|  | **Q4G0N4 - NAD kinase 2, mitochondrial OS=Homo sapiens GN=NADK2 PE=1 SV=2 - [NAKD2\_HUMAN]** | | | | | | | | | | | | | | | | | | | | | | | | | | | | | | |  |
|  | | | | | | | | | | | | | | | | | | | | | | | | | | | | | | | | |
|  | High |  | VATQAVEDVLNIAK |  | CID |  |  |  | 76.25 |  |  |  | 1.00 |  | 1 |  | Mascot (2) |  | 2 |  | 735.91437 |  | 3.50 |  | 2530 |  | 2530 |  | Image |  | Peak List |  |
|  | | | | | | | | | | | | | | | | | | | | | | | | | | | | | | | | |
|  | High |  | VATQAVEDVLNIAK |  | CID |  |  |  |  |  | 3.57 |  | 1.00 |  | 1 |  | SEQUEST (4) |  | 2 |  | 735.91437 |  | 3.50 |  | 2530 |  | 2530 |  | Image |  | Peak List |  |
|  | | | | | | | | | | | | | | | | | | | | | | | | | | | | | | | | |
|  | **Q6YN16 - Hydroxysteroid dehydrogenase-like protein 2 OS=Homo sapiens GN=HSDL2 PE=1 SV=1 - [HSDL2\_HUMAN]** | | | | | | | | | | | | | | | | | | | | | | | | | | | | | | |  |
|  | | | | | | | | | | | | | | | | | | | | | | | | | | | | | | | | |
|  | High |  | LLGTIYTAAEEIEAVGGK |  | CID |  |  |  | 47.42 |  |  |  | 1.00 |  | 1 |  | Mascot (2) |  | 2 |  | 917.99609 |  | 2.81 |  | 3353 |  | 3353 |  | Image |  | Peak List |  |
|  | | | | | | | | | | | | | | | | | | | | | | | | | | | | | | | | |
|  | High |  | LLGTIYTAAEEIEAVGGK |  | CID |  |  |  |  |  | 3.36 |  | 1.00 |  | 1 |  | SEQUEST (4) |  | 2 |  | 917.99609 |  | 2.81 |  | 3353 |  | 3353 |  | Image |  | Peak List |  |
|  | | | | | | | | | | | | | | | | | | | | | | | | | | | | | | | | |
|  | **Q709F0 - Acyl-CoA dehydrogenase family member 11 OS=Homo sapiens GN=ACAD11 PE=1 SV=2 - [ACD11\_HUMAN]** | | | | | | | | | | | | | | | | | | | | | | | | | | | | | | |  |
|  | | | | | | | | | | | | | | | | | | | | | | | | | | | | | | | | |
|  | High |  | VEGLWNLFLPAVSGLSHVDYALIAEETGK |  | CID |  |  |  | 37.76 |  |  |  | 1.00 |  | 1 |  | Mascot (2) |  | 3 |  | 1043.55481 |  | 6.22 |  | 4634 |  | 4634 |  | Image |  | Peak List |  |
|  | | | | | | | | | | | | | | | | | | | | | | | | | | | | | | | | |
|  | High |  | VEGLWNLFLPAVSGLSHVDYALIAEETGK |  | CID |  |  |  |  |  | 4.25 |  | 1.00 |  | 1 |  | SEQUEST (4) |  | 3 |  | 1043.55481 |  | 6.22 |  | 4634 |  | 4634 |  | Image |  | Peak List |  |
|  | | | | | | | | | | | | | | | | | | | | | | | | | | | | | | | | |
|  | **Q96IU4 - Alpha/beta hydrolase domain-containing protein 14B OS=Homo sapiens GN=ABHD14B PE=1 SV=1 - [ABHEB\_HUMAN]** | | | | | | | | | | | | | | | | | | | | | | | | | | | | | | |  |
|  | | | | | | | | | | | | | | | | | | | | | | | | | | | | | | | | |
|  | High |  | TPALIVYGDQDPMGQTSFEHLK |  | CID |  |  |  | 47.03 |  |  |  | 1.00 |  | 1 |  | Mascot (2) |  | 3 |  | 816.40405 |  | 2.60 |  | 2345 |  | 2345 |  | Image |  | Peak List |  |
|  | | | | | | | | | | | | | | | | | | | | | | | | | | | | | | | | |
|  | High |  | TPALIVYGDQDPMGQTSFEHLK |  | CID |  |  |  |  |  | 4.23 |  | 1.00 |  | 1 |  | SEQUEST (4) |  | 3 |  | 816.40405 |  | 2.60 |  | 2345 |  | 2345 |  | Image |  | Peak List |  |
|  | | | | | | | | | | | | | | | | | | | | | | | | | | | | | | | | |
|  | **Q96NU7 - Probable imidazolonepropionase OS=Homo sapiens GN=AMDHD1 PE=2 SV=2 - [HUTI\_HUMAN]** | | | | | | | | | | | | | | | | | | | | | | | | | | | | | | |  |
|  | | | | | | | | | | | | | | | | | | | | | | | | | | | | | | | | |
|  | High |  | MSMPEALAAATINAAYALGK |  | CID |  |  |  | 55.30 |  |  |  | 1.00 |  | 1 |  | Mascot (2) |  | 2 |  | 997.51373 |  | 5.78 |  | 4178 |  | 4178 |  | Image |  | Peak List |  |
|  | | | | | | | | | | | | | | | | | | | | | | | | | | | | | | | | |
|  | High |  | MSMPEALAAATINAAYALGK |  | CID |  |  |  |  |  | 3.96 |  | 1.00 |  | 1 |  | SEQUEST (4) |  | 2 |  | 997.51373 |  | 5.78 |  | 4178 |  | 4178 |  | Image |  | Peak List |  |
|  | | | | | | | | | | | | | | | | | | | | | | | | | | | | | | | | |
|  | **Q9BWD1 - Acetyl-CoA acetyltransferase, cytosolic OS=Homo sapiens GN=ACAT2 PE=1 SV=2 - [THIC\_HUMAN]** | | | | | | | | | | | | | | | | | | | | | | | | | | | | | | |  |
|  | | | | | | | | | | | | | | | | | | | | | | | | | | | | | | | | |
|  | High |  | AGWSLEDVDIFEINEAFAAVSAAIVK |  | CID |  |  |  | 84.96 |  |  |  | 1.00 |  | 1 |  | Mascot (2) |  | 3 |  | 922.47754 |  | 5.31 |  | 5411 |  | 5411 |  | Image |  | Peak List |  |
|  | | | | | | | | | | | | | | | | | | | | | | | | | | | | | | | | |
|  | High |  | AGWSLEDVDIFEINEAFAAVSAAIVK |  | CID |  |  |  |  |  | 6.53 |  | 1.00 |  | 1 |  | SEQUEST (4) |  | 3 |  | 922.47754 |  | 5.31 |  | 5411 |  | 5411 |  | Image |  | Peak List |  |
|  | | | | | | | | | | | | | | | | | | | | | | | | | | | | | | | | |
|  | **Q9UBR2 - Cathepsin Z OS=Homo sapiens GN=CTSZ PE=1 SV=1 - [CATZ\_HUMAN]** | | | | | | | | | | | | | | | | | | | | | | | | | | | | | | |  |
|  | | | | | | | | | | | | | | | | | | | | | | | | | | | | | | | | |
|  | High |  | NVDGVNYASITR |  | CID |  |  |  | 47.38 |  |  |  | 1.00 |  | 1 |  | Mascot (2) |  | 2 |  | 654.83197 |  | 1.81 |  | 1373 |  | 1373 |  | Image |  | Peak List |  |
|  | | | | | | | | | | | | | | | | | | | | | | | | | | | | | | | | |
|  | High |  | NVDGVNYASITR |  | CID |  |  |  |  |  | 2.51 |  | 1.00 |  | 1 |  | SEQUEST (4) |  | 2 |  | 654.83197 |  | 1.81 |  | 1373 |  | 1373 |  | Image |  | Peak List |  |
|  | | | | | | | | | | | | | | | | | | | | | | | | | | | | | | | | |
|  | **Q9UDR5 - Alpha-aminoadipic semialdehyde synthase, mitochondrial OS=Homo sapiens GN=AASS PE=1 SV=1 - [AASS\_HUMAN]** | | | | | | | | | | | | | | | | | | | | | | | | | | | | | | |  |
|  | | | | | | | | | | | | | | | | | | | | | | | | | | | | | | | | |
|  | High |  | QDLVISLLPYVLHPLVAK |  | CID |  |  |  | 39.55 |  |  |  | 1.00 |  | 1 |  | Mascot (2) |  | 3 |  | 673.40881 |  | 3.44 |  | 4038 |  | 4038 |  | Image |  | Peak List |  |
|  | | | | | | | | | | | | | | | | | | | | | | | | | | | | | | | | |
|  | High |  | QDLVISLLPYVLHPLVAK |  | CID |  |  |  |  |  | 2.49 |  | 1.00 |  | 1 |  | SEQUEST (4) |  | 3 |  | 673.40881 |  | 3.44 |  | 4038 |  | 4038 |  | Image |  | Peak List |  |
|  | | | | | | | | | | | | | | | | | | | | | | | | | | | | | | | | |
|  | **Q9Y4L1 - Hypoxia up-regulated protein 1 OS=Homo sapiens GN=HYOU1 PE=1 SV=1 - [HYOU1\_HUMAN]** | | | | | | | | | | | | | | | | | | | | | | | | | | | | | | |  |
|  | | | | | | | | | | | | | | | | | | | | | | | | | | | | | | | | |
|  | High |  | VPGPVQQALQSAEMSLDEIEQVILVGGATR |  | CID |  |  |  | 40.18 |  |  |  | 1.00 |  | 1 |  | Mascot (2) |  | 3 |  | 1045.89026 |  | 6.57 |  | 5572 |  | 5572 |  | Image |  | Peak List |  |
|  | | | | | | | | | | | | | | | | | | | | | | | | | | | | | | | | |
|  | High |  | VPGPVQQALQSAEMSLDEIEQVILVGGATR |  | CID |  |  |  |  |  | 3.83 |  | 1.00 |  | 1 |  | SEQUEST (4) |  | 3 |  | 1045.89026 |  | 6.57 |  | 5572 |  | 5572 |  | Image |  | Peak List |  |
|  | | | | | | | | | | | | | | | | | | | | | | | | | | | | | | | | |
|  | **B5MCP9 - 40S ribosomal protein S7 OS=Homo sapiens GN=RPS7 PE=4 SV=1 - [B5MCP9\_HUMAN]** | | | | | | | | | | | | | | | | | | | | | | | | | | | | | | |  |
|  | | | | | | | | | | | | | | | | | | | | | | | | | | | | | | | | |
|  | High |  | IVKPNGEKPDEFESGISQALLELEMNSDLK |  | CID |  |  |  |  |  | 2.87 |  | 1.00 |  | 1 |  | SEQUEST (4) |  | 4 |  | 833.42603 |  | 1.42 |  | 3285 |  | 3285 |  | Image |  | Peak List |  |
|  | | | | | | | | | | | | | | | | | | | | | | | | | | | | | | | | |
|  | **B5MD38 - 3-ketoacyl-CoA thiolase OS=Homo sapiens GN=HADHB PE=3 SV=1 - [B5MD38\_HUMAN]** | | | | | | | | | | | | | | | | | | | | | | | | | | | | | | |  |
|  | | | | | | | | | | | | | | | | | | | | | | | | | | | | | | | | |
|  | High |  | VGLPPLEK |  | CID |  |  |  |  |  | 1.17 |  | 1.00 |  | 1 |  | SEQUEST (4) |  | 2 |  | 426.76334 |  | 0.49 |  | 1454 |  | 1454 |  | Image |  | Peak List |  |
|  | | | | | | | | | | | | | | | | | | | | | | | | | | | | | | | | |
|  | **B7Z2F4 - T-complex protein 1 subunit delta OS=Homo sapiens GN=CCT4 PE=2 SV=1 - [B7Z2F4\_HUMAN]** | | | | | | | | | | | | | | | | | | | | | | | | | | | | | | |  |
|  | | | | | | | | | | | | | | | | | | | | | | | | | | | | | | | | |
|  | High |  | KGGISNILEELVVQPLLVSVSALTLATETVR |  | CID |  |  |  |  |  | 1.94 |  | 1.00 |  | 1 |  | SEQUEST (4) |  | 3 |  | 1083.96655 |  | 5.58 |  | 5932 |  | 5932 |  | Image |  | Peak List |  |
|  | | | | | | | | | | | | | | | | | | | | | | | | | | | | | | | | |
|  | **C9J8U2 - Nicotinate phosphoribosyltransferase OS=Homo sapiens GN=NAPRT1 PE=4 SV=1 - [C9J8U2\_HUMAN]** | | | | | | | | | | | | | | | | | | | | | | | | | | | | | | |  |
|  | | | | | | | | | | | | | | | | | | | | | | | | | | | | | | | | |
|  | High |  | LLGSDGSPLMDMLQLAEEPVPQAGQELR |  | CID |  |  |  |  |  | 3.05 |  | 1.00 |  | 1 |  | SEQUEST (4) |  | 3 |  | 998.83978 |  | 4.52 |  | 4474 |  | 4474 |  | Image |  | Peak List |  |
|  | | | | | | | | | | | | | | | | | | | | | | | | | | | | | | | | |
|  | **C9JEY0 - 3-ketoacyl-CoA thiolase (Fragment) OS=Homo sapiens GN=HADHB PE=4 SV=1 - [C9JEY0\_HUMAN]** | | | | | | | | | | | | | | | | | | | | | | | | | | | | | | |  |
|  | | | | | | | | | | | | | | | | | | | | | | | | | | | | | | | | |
|  | High |  | TPAHTVTmACISANQAMTTGVG |  | CID |  | M8(Oxidation) |  |  |  | 1.82 |  | 1.00 |  | 1 |  | SEQUEST (4) |  | 2 |  | 1089.51306 |  | 9.16 |  | 5914 |  | 5914 |  | Image |  | Peak List |  |
|  | | | | | | | | | | | | | | | | | | | | | | | | | | | | | | | | |
|  | **E5RK99 - Collagen triple helix repeat-containing protein 1 OS=Homo sapiens GN=CTHRC1 PE=4 SV=1 - [E5RK99\_HUMAN]** | | | | | | | | | | | | | | | | | | | | | | | | | | | | | | |  |
|  | | | | | | | | | | | | | | | | | | | | | | | | | | | | | | | | |
|  | High |  | IIIEELPK |  | CID |  |  |  |  |  | 1.53 |  | 1.00 |  | 1 |  | SEQUEST (4) |  | 2 |  | 477.79816 |  | 2.07 |  | 2054 |  | 2054 |  | Image |  | Peak List |  |
|  | | | | | | | | | | | | | | | | | | | | | | | | | | | | | | | | |
|  | **E7ERX2 - Zinc finger SWIM domain-containing protein 4 OS=Homo sapiens GN=ZSWIM4 PE=4 SV=1 - [E7ERX2\_HUMAN]** | | | | | | | | | | | | | | | | | | | | | | | | | | | | | | |  |
|  | | | | | | | | | | | | | | | | | | | | | | | | | | | | | | | | |
|  | High |  | QLWDELGALWVCVVLSPHCKPEER |  | CID |  |  |  |  |  | 2.13 |  | 1.00 |  | 1 |  | SEQUEST (4) |  | 3 |  | 936.47363 |  | 1.94 |  | 6213 |  | 6213 |  | Image |  | Peak List |  |
|  | | | | | | | | | | | | | | | | | | | | | | | | | | | | | | | | |
|  | **E9PNW1 - Harmonin (Fragment) OS=Homo sapiens GN=USH1C PE=4 SV=1 - [E9PNW1\_HUMAN]** | | | | | | | | | | | | | | | | | | | | | | | | | | | | | | |  |
|  | | | | | | | | | | | | | | | | | | | | | | | | | | | | | | | | |
|  | High |  | LPLFDAIRPLIPLKHQVEYDQLTPR |  | CID |  |  |  |  |  | 2.87 |  | 1.00 |  | 1 |  | SEQUEST (4) |  | 3 |  | 991.56586 |  | 3.66 |  | 5899 |  | 5899 |  | Image |  | Peak List |  |
|  | | | | | | | | | | | | | | | | | | | | | | | | | | | | | | | | |
|  | **F2Z3B8 - Receptor-type tyrosine-protein phosphatase F OS=Homo sapiens GN=PTPRF PE=4 SV=1 - [F2Z3B8\_HUMAN]** | | | | | | | | | | | | | | | | | | | | | | | | | | | | | | |  |
|  | | | | | | | | | | | | | | | | | | | | | | | | | | | | | | | | |
|  | High |  | VTFDPTSSYTLEDLKPDTLYR |  | CID |  |  |  |  |  | 1.67 |  | 1.00 |  | 1 |  | SEQUEST (4) |  | 2 |  | 1231.11316 |  | 2.30 |  | 4925 |  | 4925 |  | Image |  | Peak List |  |
|  | | | | | | | | | | | | | | | | | | | | | | | | | | | | | | | | |
|  | **F5H0Q4 - Diablo homolog, mitochondrial (Fragment) OS=Homo sapiens GN=DIABLO PE=4 SV=1 - [F5H0Q4\_HUMAN]** | | | | | | | | | | | | | | | | | | | | | | | | | | | | | | |  |
|  | | | | | | | | | | | | | | | | | | | | | | | | | | | | | | | | |
|  | High |  | AVSLVTDSTSTFLSQTTYALIEAITEYTK |  | CID |  |  |  |  |  | 3.28 |  | 1.00 |  | 1 |  | SEQUEST (4) |  | 3 |  | 1051.87927 |  | 4.51 |  | 5693 |  | 5693 |  | Image |  | Peak List |  |
|  | | | | | | | | | | | | | | | | | | | | | | | | | | | | | | | | |
|  | **F5H2F2 - Branched-chain-amino-acid aminotransferase, cytosolic (Fragment) OS=Homo sapiens GN=BCAT1 PE=4 SV=1 - [F5H2F2\_HUMAN]** | | | | | | | | | | | | | | | | | | | | | | | | | | | | | | |  |
|  | | | | | | | | | | | | | | | | | | | | | | | | | | | | | | | | |
|  | High |  | ATLPVFDKEELLECIQQLVK |  | CID |  |  |  |  |  | 2.65 |  | 1.00 |  | 1 |  | SEQUEST (4) |  | 3 |  | 772.75574 |  | 0.26 |  | 5464 |  | 5464 |  | Image |  | Peak List |  |
|  | | | | | | | | | | | | | | | | | | | | | | | | | | | | | | | | |
|  | **F8VRD9 - Sodium-coupled neutral amino acid transporter 4 (Fragment) OS=Homo sapiens GN=SLC38A4 PE=4 SV=1 - [F8VRD9\_HUMAN]** | | | | | | | | | | | | | | | | | | | | | | | | | | | | | | |  |
|  | | | | | | | | | | | | | | | | | | | | | | | | | | | | | | | | |
|  | High |  | IGAFVSITmQNIGAMSSYLFIIK |  | CID |  | M9(Oxidation) |  |  |  | 2.03 |  | 1.00 |  | 1 |  | SEQUEST (4) |  | 3 |  | 840.78516 |  | 6.64 |  | 6182 |  | 6182 |  | Image |  | Peak List |  |
|  | | | | | | | | | | | | | | | | | | | | | | | | | | | | | | | | |
|  | **F8WEF0 - Phosphoribosylaminoimidazolecarboxamide formyltransferase OS=Homo sapiens GN=ATIC PE=4 SV=1 - [F8WEF0\_HUMAN]** | | | | | | | | | | | | | | | | | | | | | | | | | | | | | | |  |
|  | | | | | | | | | | | | | | | | | | | | | | | | | | | | | | | | |
|  | High |  | MAPGQLALFSVSDK |  | CID |  |  |  |  |  | 1.51 |  | 1.00 |  | 1 |  | SEQUEST (4) |  | 2 |  | 732.38708 |  | 7.20 |  | 7658 |  | 7658 |  | Image |  | Peak List |  |
|  | | | | | | | | | | | | | | | | | | | | | | | | | | | | | | | | |
|  | **H0YCQ8 - Eukaryotic translation initiation factor 3 subunit M (Fragment) OS=Homo sapiens GN=EIF3M PE=4 SV=1 - [H0YCQ8\_HUMAN]** | | | | | | | | | | | | | | | | | | | | | | | | | | | | | | |  |
|  | | | | | | | | | | | | | | | | | | | | | | | | | | | | | | | | |
|  | High |  | EISFDTMQQELQIGADDVEAFVIDAVR |  | CID |  |  |  |  |  | 3.27 |  | 1.00 |  | 1 |  | SEQUEST (4) |  | 3 |  | 1013.82788 |  | 2.42 |  | 5734 |  | 5734 |  | Image |  | Peak List |  |
|  | | | | | | | | | | | | | | | | | | | | | | | | | | | | | | | | |
|  | **H0YEP9 - Protein C11orf30 (Fragment) OS=Homo sapiens GN=C11orf30 PE=4 SV=1 - [H0YEP9\_HUMAN]** | | | | | | | | | | | | | | | | | | | | | | | | | | | | | | |  |
|  | | | | | | | | | | | | | | | | | | | | | | | | | | | | | | | | |
|  | High |  | TAVSDILKmSLmEAQIDTNVEHMIVDPPK |  | CID |  | M9(Oxidation); M12(Oxidation) |  |  |  | 1.90 |  | 1.00 |  | 1 |  | SEQUEST (4) |  | 4 |  | 815.16479 |  | 8.28 |  | 5755 |  | 5755 |  | Image |  | Peak List |  |
|  | | | | | | | | | | | | | | | | | | | | | | | | | | | | | | | | |
|  | **H3BU24 - S phase cyclin A-associated protein in the endoplasmic reticulum (Fragment) OS=Homo sapiens GN=SCAPER PE=4 SV=1 - [H3BU24\_HUMAN]** | | | | | | | | | | | | | | | | | | | | | | | | | | | | | | |  |
|  | | | | | | | | | | | | | | | | | | | | | | | | | | | | | | | | |
|  | High |  | VKAHHTGSTASSEITPAQSCPPmTVQK |  | CID |  | M23(Oxidation) |  |  |  | 0.58 |  | 1.00 |  | 1 |  | SEQUEST (4) |  | 2 |  | 1405.18384 |  | -0.16 |  | 6005 |  | 6005 |  | Image |  | Peak List |  |
|  | | | | | | | | | | | | | | | | | | | | | | | | | | | | | | | | |
|  | **O60667-2 - Isoform 2 of Fas apoptotic inhibitory molecule 3 OS=Homo sapiens GN=FAIM3 - [FAIM3\_HUMAN]** | | | | | | | | | | | | | | | | | | | | | | | | | | | | | | |  |
|  | | | | | | | | | | | | | | | | | | | | | | | | | | | | | | | | |
|  | High |  | MDFWLWPLYFLPEYEPSWEEQPmPETPK |  | CID |  | M23(Oxidation) |  |  |  | 1.66 |  | 1.00 |  | 1 |  | SEQUEST (4) |  | 3 |  | 1201.22229 |  | 4.19 |  | 5336 |  | 5336 |  | Image |  | Peak List |  |
|  | | | | | | | | | | | | | | | | | | | | | | | | | | | | | | | | |
|  | **C9JPQ8 - Propionyl-CoA carboxylase alpha chain, mitochondrial OS=Homo sapiens GN=PCCA PE=4 SV=2 - [C9JPQ8\_HUMAN]** | | | | | | | | | | | | | | | | | | | | | | | | | | | | | | |  |
|  | | | | | | | | | | | | | | | | | | | | | | | | | | | | | | | | |
|  | High |  | MADEAVCVGPAPTSK |  | CID |  |  |  |  |  | 1.97 |  | 1.00 |  | 1 |  | SEQUEST (4) |  | 2 |  | 738.35162 |  | 6.10 |  | 1236 |  | 1236 |  | Image |  | Peak List |  |
|  | | | | | | | | | | | | | | | | | | | | | | | | | | | | | | | | |
|  | **P07814 - Bifunctional glutamate/proline--tRNA ligase OS=Homo sapiens GN=EPRS PE=1 SV=5 - [SYEP\_HUMAN]** | | | | | | | | | | | | | | | | | | | | | | | | | | | | | | |  |
|  | | | | | | | | | | | | | | | | | | | | | | | | | | | | | | | | |
|  | High |  | EFLWQEGHSAFATMEEAAEEVLQILDLYAQVYEELLAIPVVK |  | CID |  |  |  |  |  | 4.02 |  | 1.00 |  | 1 |  | SEQUEST (4) |  | 4 |  | 1206.36829 |  | 6.20 |  | 6792 |  | 6792 |  | Image |  | Peak List |  |
|  | | | | | | | | | | | | | | | | | | | | | | | | | | | | | | | | |
|  | **P09884 - DNA polymerase alpha catalytic subunit OS=Homo sapiens GN=POLA1 PE=1 SV=2 - [DPOLA\_HUMAN]** | | | | | | | | | | | | | | | | | | | | | | | | | | | | | | |  |
|  | | | | | | | | | | | | | | | | | | | | | | | | | | | | | | | | |
|  | High |  | mNLEVIYGDTDSImINTNSTNLEEVFK |  | CID |  | M1(Oxidation); M14(Oxidation) |  |  |  | 1.97 |  | 1.00 |  | 1 |  | SEQUEST (4) |  | 3 |  | 1041.49890 |  | 8.84 |  | 5462 |  | 5462 |  | Image |  | Peak List |  |
|  | | | | | | | | | | | | | | | | | | | | | | | | | | | | | | | | |
|  | **A6NKK6 - UDP-glucuronosyltransferase 1-6 OS=Homo sapiens GN=UGT1A6 PE=3 SV=1 - [A6NKK6\_HUMAN]** | | | | | | | | | | | | | | | | | | | | | | | | | | | | | | |  |
|  | | | | | | | | | | | | | | | | | | | | | | | | | | | | | | | | |
|  | High |  | DRPVEPLDLAVFWVEFVMR |  | CID |  |  |  |  |  | 1.64 |  | 1.00 |  | 1 |  | SEQUEST (4) |  | 3 |  | 773.40887 |  | 5.07 |  | 5605 |  | 5605 |  | Image |  | Peak List |  |
|  | | | | | | | | | | | | | | | | | | | | | | | | | | | | | | | | |
|  | **P35573-2 - Isoform 5 of Glycogen debranching enzyme OS=Homo sapiens GN=AGL - [GDE\_HUMAN]** | | | | | | | | | | | | | | | | | | | | | | | | | | | | | | |  |
|  | | | | | | | | | | | | | | | | | | | | | | | | | | | | | | | | |
|  | High |  | NLQPNLYVVAELFTGSEDLDNVFVTR |  | CID |  |  |  |  |  | 2.58 |  | 1.00 |  | 1 |  | SEQUEST (4) |  | 3 |  | 985.17395 |  | 4.40 |  | 4843 |  | 4843 |  | Image |  | Peak List |  |
|  | | | | | | | | | | | | | | | | | | | | | | | | | | | | | | | | |
|  | **P49591 - Serine--tRNA ligase, cytoplasmic OS=Homo sapiens GN=SARS PE=1 SV=3 - [SYSC\_HUMAN]** | | | | | | | | | | | | | | | | | | | | | | | | | | | | | | |  |
|  | | | | | | | | | | | | | | | | | | | | | | | | | | | | | | | | |
|  | High |  | SWEMFEEMITTAEEFYQSLGIPYHIVNIVSGSLNHAASK |  | CID |  |  |  |  |  | 2.71 |  | 1.00 |  | 1 |  | SEQUEST (4) |  | 4 |  | 1108.04297 |  | 7.75 |  | 5689 |  | 5689 |  | Image |  | Peak List |  |
|  | | | | | | | | | | | | | | | | | | | | | | | | | | | | | | | | |
|  | **Q14117 - Dihydropyrimidinase OS=Homo sapiens GN=DPYS PE=1 SV=1 - [DPYS\_HUMAN]** | | | | | | | | | | | | | | | | | | | | | | | | | | | | | | |  |
|  | | | | | | | | | | | | | | | | | | | | | | | | | | | | | | | | |
|  | High |  | VVYGEPIAASLGTDGTHYWNK |  | CID |  |  |  |  |  | 1.77 |  | 1.00 |  | 1 |  | SEQUEST (4) |  | 3 |  | 760.04425 |  | 1.86 |  | 2155 |  | 2155 |  | Image |  | Peak List |  |
|  | | | | | | | | | | | | | | | | | | | | | | | | | | | | | | | | |
|  | **Q14353 - Guanidinoacetate N-methyltransferase OS=Homo sapiens GN=GAMT PE=1 SV=1 - [GAMT\_HUMAN]** | | | | | | | | | | | | | | | | | | | | | | | | | | | | | | |  |
|  | | | | | | | | | | | | | | | | | | | | | | | | | | | | | | | | |
|  | High |  | GLWEDVAPTLPDGHFDGILYDTYPLSEETWHTHQFNFIK |  | CID |  |  |  |  |  | 2.50 |  | 1.00 |  | 1 |  | SEQUEST (4) |  | 5 |  | 918.64850 |  | 8.88 |  | 3606 |  | 3606 |  | Image |  | Peak List |  |
|  | | | | | | | | | | | | | | | | | | | | | | | | | | | | | | | | |
|  | **Q5TCU6 - Talin 1 OS=Homo sapiens GN=TLN1 PE=2 SV=1 - [Q5TCU6\_HUMAN]** | | | | | | | | | | | | | | | | | | | | | | | | | | | | | | |  |
|  | | | | | | | | | | | | | | | | | | | | | | | | | | | | | | | | |
|  | High |  | GTEWVDPEDPTVIAENELLGAAAAIEAAAK |  | CID |  |  |  |  |  | 3.44 |  | 1.00 |  | 1 |  | SEQUEST (4) |  | 3 |  | 1017.84784 |  | 4.29 |  | 5096 |  | 5096 |  | Image |  | Peak List |  |
|  | | | | | | | | | | | | | | | | | | | | | | | | | | | | | | | | |
|  | **Q6PKG0-3 - Isoform 2 of La-related protein 1 OS=Homo sapiens GN=LARP1 - [LARP1\_HUMAN]** | | | | | | | | | | | | | | | | | | | | | | | | | | | | | | |  |
|  | | | | | | | | | | | | | | | | | | | | | | | | | | | | | | | | |
|  | High |  | SVQPQSHKPQPTRK |  | CID |  |  |  |  |  | 1.69 |  | 1.00 |  | 1 |  | SEQUEST (4) |  | 2 |  | 809.44464 |  | -0.01 |  | 5604 |  | 5604 |  | Image |  | Peak List |  |
|  | | | | | | | | | | | | | | | | | | | | | | | | | | | | | | | | |
|  | **Q6ZW49-2 - Isoform 3 of PAX-interacting protein 1 OS=Homo sapiens GN=PAXIP1 - [PAXI1\_HUMAN]** | | | | | | | | | | | | | | | | | | | | | | | | | | | | | | |  |
|  | | | | | | | | | | | | | | | | | | | | | | | | | | | | | | | | |
|  | High |  | QNEVANVQPSSKR |  | CID |  |  |  |  |  | 1.98 |  | 1.00 |  | 1 |  | SEQUEST (4) |  | 3 |  | 486.25787 |  | 5.99 |  | 1017 |  | 1017 |  | Image |  | Peak List |  |
|  | | | | | | | | | | | | | | | | | | | | | | | | | | | | | | | | |
|  | **Q86UK7-2 - Isoform 2 of Zinc finger protein 598 OS=Homo sapiens GN=ZNF598 - [ZN598\_HUMAN]** | | | | | | | | | | | | | | | | | | | | | | | | | | | | | | |  |
|  | | | | | | | | | | | | | | | | | | | | | | | | | | | | | | | | |
|  | High |  | TQGEGPGPKETSTNGPVSQEAFSVTGPAAPGALPPPSPK |  | CID |  |  |  |  |  | 1.82 |  | 1.00 |  | 1 |  | SEQUEST (4) |  | 5 |  | 749.38385 |  | 9.10 |  | 2088 |  | 2088 |  | Image |  | Peak List |  |
|  | | | | | | | | | | | | | | | | | | | | | | | | | | | | | | | | |
|  | **Q8IWZ3-5 - Isoform 5 of Ankyrin repeat and KH domain-containing protein 1 OS=Homo sapiens GN=ANKHD1 - [ANKH1\_HUMAN]** | | | | | | | | | | | | | | | | | | | | | | | | | | | | | | |  |
|  | | | | | | | | | | | | | | | | | | | | | | | | | | | | | | | | |
|  | High |  | GHLDmVRFLLEAGADQEHKTDEMHTALMEACmDGHVEVAR |  | CID |  | M5(Oxidation); M32(Oxidation) |  |  |  | 1.96 |  | 1.00 |  | 1 |  | SEQUEST (4) |  | 7 |  | 647.29370 |  | -7.55 |  | 7896 |  | 7896 |  | Image |  | Peak List |  |
|  | | | | | | | | | | | | | | | | | | | | | | | | | | | | | | | | |
|  | **Q8IXK2 - Polypeptide N-acetylgalactosaminyltransferase 12 OS=Homo sapiens GN=GALNT12 PE=1 SV=3 - [GLT12\_HUMAN]** | | | | | | | | | | | | | | | | | | | | | | | | | | | | | | |  |
|  | | | | | | | | | | | | | | | | | | | | | | | | | | | | | | | | |
|  | High |  | TVYSVLETSPDILLEEVILVDDYSDREHLK |  | CID |  |  |  |  |  | 2.77 |  | 1.00 |  | 1 |  | SEQUEST (4) |  | 5 |  | 698.96625 |  | 5.17 |  | 4390 |  | 4390 |  | Image |  | Peak List |  |
|  | | | | | | | | | | | | | | | | | | | | | | | | | | | | | | | | |
|  | **Q8N165 - Serine/threonine-protein kinase PDIK1L OS=Homo sapiens GN=PDIK1L PE=2 SV=1 - [PDK1L\_HUMAN]** | | | | | | | | | | | | | | | | | | | | | | | | | | | | | | |  |
|  | | | | | | | | | | | | | | | | | | | | | | | | | | | | | | | | |
|  | High |  | DGMVQKMSHGSNSSLYLQLVETSLK |  | CID |  |  |  |  |  | 2.16 |  | 1.00 |  | 1 |  | SEQUEST (4) |  | 3 |  | 918.13538 |  | 9.82 |  | 6604 |  | 6604 |  | Image |  | Peak List |  |
|  | | | | | | | | | | | | | | | | | | | | | | | | | | | | | | | | |
|  | **Q8NFC6 - Biorientation of chromosomes in cell division protein 1-like 1 OS=Homo sapiens GN=BOD1L1 PE=1 SV=2 - [BD1L1\_HUMAN]** | | | | | | | | | | | | | | | | | | | | | | | | | | | | | | |  |
|  | | | | | | | | | | | | | | | | | | | | | | | | | | | | | | | | |
|  | High |  | VTTEEFEAPMPSAVSGDDSQLTASRSEEK |  | CID |  |  |  |  |  | 0.65 |  | 1.00 |  | 1 |  | SEQUEST (4) |  | 2 |  | 1549.71973 |  | 6.90 |  | 5827 |  | 5827 |  | Image |  | Peak List |  |
|  | | | | | | | | | | | | | | | | | | | | | | | | | | | | | | | | |
|  | **Q96HY7 - Probable 2-oxoglutarate dehydrogenase E1 component DHKTD1, mitochondrial OS=Homo sapiens GN=DHTKD1 PE=2 SV=2 - [DHTK1\_HUMAN]** | | | | | | | | | | | | | | | | | | | | | | | | | | | | | | |  |
|  | | | | | | | | | | | | | | | | | | | | | | | | | | | | | | | | |
|  | High |  | LLPLWEAQFGDFFNGAQIIFDTFISGGEAK |  | CID |  |  |  |  |  | 4.01 |  | 1.00 |  | 1 |  | SEQUEST (4) |  | 3 |  | 1111.23792 |  | 9.49 |  | 6221 |  | 6221 |  | Image |  | Peak List |  |
|  | | | | | | | | | | | | | | | | | | | | | | | | | | | | | | | | |
|  | **Q9BPW9-2 - Isoform 2 of Dehydrogenase/reductase SDR family member 9 OS=Homo sapiens GN=DHRS9 - [DHRS9\_HUMAN]** | | | | | | | | | | | | | | | | | | | | | | | | | | | | | | |  |
|  | | | | | | | | | | | | | | | | | | | | | | | | | | | | | | | | |
|  | High |  | IFWIPLSHMPAALQDFLLLK |  | CID |  |  |  |  |  | 1.80 |  | 1.00 |  | 1 |  | SEQUEST (4) |  | 3 |  | 785.11194 |  | 3.00 |  | 6476 |  | 6476 |  | Image |  | Peak List |  |
|  | | | | | | | | | | | | | | | | | | | | | | | | | | | | | | | | |
|  | **Q9BY49-2 - Isoform 2 of Peroxisomal trans-2-enoyl-CoA reductase OS=Homo sapiens GN=PECR - [PECR\_HUMAN]** | | | | | | | | | | | | | | | | | | | | | | | | | | | | | | |  |
|  | | | | | | | | | | | | | | | | | | | | | | | | | | | | | | | | |
|  | High |  | IGVPEEVSSVVCFLLSPAASFITGQSVDVDGGR |  | CID |  |  |  |  |  | 2.97 |  | 1.00 |  | 1 |  | SEQUEST (4) |  | 3 |  | 1112.57153 |  | 5.12 |  | 6008 |  | 6008 |  | Image |  | Peak List |  |
|  | | | | | | | | | | | | | | | | | | | | | | | | | | | | | | | | |
|  | **Q9HAT2-2 - Isoform 2 of Sialate O-acetylesterase OS=Homo sapiens GN=SIAE - [SIAE\_HUMAN]** | | | | | | | | | | | | | | | | | | | | | | | | | | | | | | |  |
|  | | | | | | | | | | | | | | | | | | | | | | | | | | | | | | | | |
|  | High |  | FFPFGLVQLSSDLSK |  | CID |  |  |  |  |  | 1.83 |  | 1.00 |  | 1 |  | SEQUEST (4) |  | 2 |  | 842.95862 |  | 9.14 |  | 4009 |  | 4009 |  | Image |  | Peak List |  |
|  | | | | | | | | | | | | | | | | | | | | | | | | | | | | | | | | |
|  | **Q9NT99 - Leucine-rich repeat-containing protein 4B OS=Homo sapiens GN=LRRC4B PE=1 SV=3 - [LRC4B\_HUMAN]** | | | | | | | | | | | | | | | | | | | | | | | | | | | | | | |  |
|  | | | | | | | | | | | | | | | | | | | | | | | | | | | | | | | | |
|  | High |  | EPPGPTTDGVWGGGRPGDAAGPASSSTTAPAPR |  | CID |  |  |  |  |  | 2.18 |  | 1.00 |  | 1 |  | SEQUEST (4) |  | 3 |  | 1025.49133 |  | 0.99 |  | 5018 |  | 5018 |  | Image |  | Peak List |  |
|  | | | | | | | | | | | | | | | | | | | | | | | | | | | | | | | | |
|  | **Q9NWW6-2 - Isoform 2 of Nicotinamide riboside kinase 1 OS=Homo sapiens GN=NMRK1 - [NRK1\_HUMAN]** | | | | | | | | | | | | | | | | | | | | | | | | | | | | | | |  |
|  | | | | | | | | | | | | | | | | | | | | | | | | | | | | | | | | |
|  | High |  | SEEDLFLQVYEDLIQELAK |  | CID |  |  |  |  |  | 2.71 |  | 1.00 |  | 1 |  | SEQUEST (4) |  | 2 |  | 1141.58020 |  | 4.03 |  | 5516 |  | 5516 |  | Image |  | Peak List |  |
|  | | | | | | | | | | | | | | | | | | | | | | | | | | | | | | | | |
|  | **Q9UKG4 - Solute carrier family 13 member 4 OS=Homo sapiens GN=SLC13A4 PE=2 SV=2 - [S13A4\_HUMAN]** | | | | | | | | | | | | | | | | | | | | | | | | | | | | | | |  |
|  | | | | | | | | | | | | | | | | | | | | | | | | | | | | | | | | |
|  | High |  | IQEEYEKLGDISYPEmVTGFFFILMTVLWFTR |  | CID |  | M16(Oxidation) |  |  |  | 2.27 |  | 1.00 |  | 1 |  | SEQUEST (4) |  | 4 |  | 980.49304 |  | 3.29 |  | 5517 |  | 5517 |  | Image |  | Peak List |  |
|  | | | | | | | | | | | | | | | | | | | | | | | | | | | | | | | | |
|  | **B3KX72 - Heterogeneous nuclear ribonucleoprotein U OS=Homo sapiens GN=HNRNPU PE=2 SV=1 - [B3KX72\_HUMAN]** | | | | | | | | | | | | | | | | | | | | | | | | | | | | | | |  |
|  | | | | | | | | | | | | | | | | | | | | | | | | | | | | | | | | |
|  | High |  | EKPYFPIPEEYTFIQNVPLEDR |  | CID |  |  |  |  |  | 2.70 |  | 1.00 |  | 1 |  | SEQUEST (4) |  | 3 |  | 908.79358 |  | 3.86 |  | 3238 |  | 3238 |  | Image |  | Peak List |  |
|  | | | | | | | | | | | | | | | | | | | | | | | | | | | | | | | | |
|  | **B4DW08 - Uncharacterized protein OS=Homo sapiens GN=ACO2 PE=2 SV=1 - [B4DW08\_HUMAN]** | | | | | | | | | | | | | | | | | | | | | | | | | | | | | | |  |
|  | | | | | | | | | | | | | | | | | | | | | | | | | | | | | | | | |
|  | High |  | SQFTITPGSEQIR |  | CID |  |  |  |  |  | 1.58 |  | 1.00 |  | 1 |  | SEQUEST (4) |  | 2 |  | 732.38062 |  | 3.42 |  | 1477 |  | 1477 |  | Image |  | Peak List |  |
|  | | | | | | | | | | | | | | | | | | | | | | | | | | | | | | | | |
|  | **F5H3L8 - Stress-70 protein, mitochondrial OS=Homo sapiens GN=HSPA9 PE=3 SV=1 - [F5H3L8\_HUMAN]** | | | | | | | | | | | | | | | | | | | | | | | | | | | | | | |  |
|  | | | | | | | | | | | | | | | | | | | | | | | | | | | | | | | | |
|  | High |  | VEAVNMAEGIIHDTETK |  | CID |  |  |  |  |  | 1.68 |  | 1.00 |  | 1 |  | SEQUEST (4) |  | 3 |  | 619.64142 |  | 2.02 |  | 2174 |  | 2174 |  | Image |  | Peak List |  |
|  | | | | | | | | | | | | | | | | | | | | | | | | | | | | | | | | |
|  | **H0Y3D8 - Alpha-N-acetylglucosaminidase 77 kDa form OS=Homo sapiens GN=NAGLU PE=4 SV=1 - [H0Y3D8\_HUMAN]** | | | | | | | | | | | | | | | | | | | | | | | | | | | | | | |  |
|  | | | | | | | | | | | | | | | | | | | | | | | | | | | | | | | | |
|  | High |  | LFPNSTMVGTGMAPEGISQNEVVYSLmAELGWR |  | CID |  | M27(Oxidation) |  |  |  | 2.21 |  | 1.00 |  | 1 |  | SEQUEST (4) |  | 3 |  | 1200.91870 |  | 6.73 |  | 5017 |  | 5017 |  | Image |  | Peak List |  |
|  | | | | | | | | | | | | | | | | | | | | | | | | | | | | | | | | |
|  | **Q70L72 - Antigen KI-67 (Fragment) OS=Homo sapiens GN=MKI67 PE=2 SV=1 - [Q70L72\_HUMAN]** | | | | | | | | | | | | | | | | | | | | | | | | | | | | | | |  |
|  | | | | | | | | | | | | | | | | | | | | | | | | | | | | | | | | |
|  | High |  | SGVDGPHFPLSLSTCLFGR |  | CID |  |  |  |  |  | 1.80 |  | 1.00 |  | 1 |  | SEQUEST (4) |  | 3 |  | 664.00275 |  | 4.26 |  | 3226 |  | 3226 |  | Image |  | Peak List |  |
|  | | | | | | | | | | | | | | | | | | | | | | | | | | | | | | | | |

  
Top
  

### Search Summary

Workflow created with Discoverer version: 1.4.0.288 (DBVersion:79)
  
  
================================================================================
  
  
Search name: Water\_1\_MC3
  
Search description: -
  
Search date: 06/25/2014 16:31:56
  
  
================================================================================
  
  
The pipeline tree:
  
------------------
  
  
    |-(0) Spectrum Files
  
        |-(1) Spectrum Selector
  
            |-(2) Mascot
  
                |-(3) Percolator
  
            |-(4) SEQUEST
  
                |-(3) Percolator
  
  
================================================================================
  
  
Search name: Water\_1\_MC3
  
Search description: -
  
Search date: 06/25/2014 16:31:56
  
  
================================================================================
  
  
The pipeline tree:
  
------------------
  
  
    |-(0) Spectrum Files
  
        |-(1) Spectrum Selector
  
            |-(2) Mascot
  
                |-(3) Percolator
  
            |-(4) SEQUEST
  
                |-(3) Percolator
  
  
------------------------------------------------------------------------------
  
Processing node 0: Spectrum Files
  
------------------------------------------------------------------------------
  
  
Input Data:
  
-----------------------------
  
File Name(s): E:\Jos\Water\_1.raw
  
  
------------------------------------------------------------------------------
  
Processing node 1: Spectrum Selector
  
------------------------------------------------------------------------------
  
  
1. General Settings:
  
-----------------------------
  
Precursor Selection: Use MS1 Precursor
  
Use New Precursor Reevaluation: True
  
  
2. Spectrum Properties Filter:
  
-----------------------------
  
Lower RT Limit: 0
  
Upper RT Limit: 0
  
First Scan: 0
  
Last Scan: 0
  
Lowest Charge State: 0
  
Highest Charge State: 0
  
Min. Precursor Mass: 350 Da
  
Max. Precursor Mass: 5000 Da
  
Total Intensity Threshold: 0
  
Minimum Peak Count: 1
  
  
3. Scan Event Filters:
  
-----------------------------
  
MS Order: Is MS2
  
Activation Type: Is CID
  
Min. Collision Energy: 0
  
Max. Collision Energy: 1000
  
Scan Type: Is Full
  
Ionization Source: Is Nanospray
  
  
4. Peak Filters:
  
-----------------------------
  
S/N Threshold (FT-only): 1.5
  
  
5. Replacements for Unrecognized Properties:
  
-----------------------------
  
Unrecognized Charge Replacements: Automatic
  
Unrecognized Mass Analyzer Replacements: ITMS
  
Unrecognized MS Order Replacements: MS2
  
Unrecognized Activation Type Replacements: CID
  
Unrecognized Polarity Replacements: +
  
  
6. Just for Testing:
  
-----------------------------
  
Precursor Clipping Range Before: 2.5 Da
  
Precursor Clipping Range After: 5.5 Da
  
  
------------------------------------------------------------------------------
  
Processing node 2: Mascot
  
------------------------------------------------------------------------------
  
  
1. Input Data:
  
-----------------------------
  
Protein Database: SwissProt
  
Enzyme Name: Trypsin
  
Maximum Missed Cleavage Sites: 3
  
Instrument: Default
  
Taxonomy: . . . . . . . . . . . . . . . . Homo sapiens (human)
  
  
1.1 Peptide Scoring Options:
  
-----------------------------
  
Peptide Cut Off Score: 10
  
Peptide Without Protein Cut Off Score: 5
  
  
1.2 Protein Scoring Options:
  
-----------------------------
  
Use MudPIT Scoring: Automatic
  
Protein Relevance Threshold: 20
  
Protein Relevance Factor: 1
  
  
2. Tolerances:
  
-----------------------------
  
Precursor Mass Tolerance: 10 ppm
  
Fragment Mass Tolerance: 0.8 Da
  
Use Average Precursor Mass: False
  
  
4. Dynamic Modifications:
  
-----------------------------
  
1. Dynamic Modification: Oxidation (M)
  
  
------------------------------------------------------------------------------
  
Processing node 3: Percolator
  
------------------------------------------------------------------------------
  
  
1. Input Data:
  
-----------------------------
  
Maximum Delta Cn: 0.05
  
  
2. Decoy Database Search:
  
-----------------------------
  
Target FDR (Strict): 0.01
  
Target FDR (Relaxed): 0.05
  
Validation based on: q-Value
  
  
------------------------------------------------------------------------------
  
Processing node 4: SEQUEST
  
------------------------------------------------------------------------------
  
  
1. Input Data:
  
-----------------------------
  
Protein Database: HUMAN\_swiss\_Jos.fasta
  
Enzyme Name: Trypsin (Full)
  
Maximum Missed Cleavage Sites: 3
  
  
1.1 Peptide Scoring Options:
  
-----------------------------
  
Maximum Peptides Considered: 500
  
Maximum Peptides Output: 10
  
Calculate Probability Scores: False
  
Absolute XCorr Threshold: 0.4
  
Fragment Ion Cutoff Percentage: 0.1
  
Peptide Without Protein XCorr Threshold: 1.5
  
  
1.2 Protein Scoring Options:
  
-----------------------------
  
Maximum Protein References Per Peptide: 100
  
Protein Relevance Threshold: 1.5
  
Peptide Relevance Factor: 0.4
  
  
2. Tolerances:
  
-----------------------------
  
Precursor Mass Tolerance: 10 ppm
  
Fragment Mass Tolerance: 0.8 Da
  
Use Average Precursor Mass: False
  
Use Average Fragment Masses: False
  
  
3. Ion Series:
  
-----------------------------
  
Use Neutral Loss a Ions: True
  
Use Neutral Loss b Ions: True
  
Use Neutral Loss y Ions: True
  
Weight of a Ions: 0
  
Weight of b Ions: 1
  
Weight of c Ions: 0
  
Weight of x Ions: 0
  
Weight of y Ions: 1
  
Weight of z Ions: 0
  
  
4. Dynamic Modifications:
  
-----------------------------
  
Max. Modifications Per Peptide: 4
  
1. Dynamic Modification: Oxidation / +15.995 Da (M)
  
  
================================================================================
  
  
Processing details:
  
  
06/25/2014 05:05 PM (4):SEQUEST: Total search time was 2 min 44 s.
  
06/25/2014 05:05 PM (3):Percolator: Performing percolator for SEQUEST (4) took 1 min 11 s.
  
06/25/2014 05:05 PM (4):SEQUEST: Search completed
  
06/25/2014 05:05 PM (4):SEQUEST: 14349 protein(s) + 13225 decoy proteins scored and inserted into result file in 8.1 s.
  
06/25/2014 05:05 PM (4):SEQUEST: 14349 protein(s) scored
  
06/25/2014 05:05 PM (4):SEQUEST: Search result finalization started.
  
06/25/2014 05:05 PM (3):Percolator: Start reading Percolator results
  
06/25/2014 05:05 PM (3):Percolator: PSMId score q-value posterior\_error\_prob peptide proteinIds
  
06/25/2014 05:05 PM (3):Percolator: Processing took 6.848 cpu seconds or 7 seconds wall time
  
06/25/2014 05:05 PM (3):Percolator: Calibrating statistics - calculating Posterior error probabilities (PEPs)
  
06/25/2014 05:05 PM (3):Percolator: New pi\_0 estimate on merged list gives 1449 peptides over q=0.0100
  
06/25/2014 05:05 PM (3):Percolator: Calibrating statistics - calculating q values
  
06/25/2014 05:05 PM (3):Percolator: Selecting pi\_0=0.6482
  
06/25/2014 05:05 PM (3):Percolator: Tossing out "redundant" PSMs keeping only the best scoring PSM for each unique peptide.
  
06/25/2014 05:05 PM (3):Percolator: Merging results from 3 datasets
  
06/25/2014 05:05 PM (3):Percolator: Found 2576 target PSMs scoring over 1.0000% FDR level on testset
  
06/25/2014 05:05 PM (3):Percolator: 0.7522 -0.0007 -0.0243 0.0293 0.0112 0.0007 44.1120 0.0686 58.7067 -0.3634 -0.0979 0.0000 -0.0632 0.1928 -0.1432 -0.0229 -1.3538 -1.0082 0.0000 -0.5910 0.0174 -0.0174 0.0071 -0.2317 0.2215 0.1415 0.2519 -2.3542 0.0006 -8.0346 0.0016 -0.8963 -0.0011 -0.1884
  
06/25/2014 05:05 PM (3):Percolator: 0.817 -0.2750 -0.0067 1.3873 0.2877 0.6644 0.6680 0.3746 0.5247 -0.9978 -0.8145 0.0000 -0.0305 0.0962 -0.0475 -0.0037 -0.1515 -1.1245 0.0000 -0.5000 0.2513 -0.3236 0.1417 -0.7611 0.7375 0.4128 0.8724 -0.4206 0.1878 -0.7341 0.2852 -0.0750 -0.2296 -2.4348
  
06/25/2014 05:05 PM (3):Percolator: XCorr SpScore Delta Cn From Second PSM Binomial Score Isolation Interference [%] MH+ [Da] Delta Mass [Da] Delta Mass [ppm] Absolute Delta Mass [Da] Absolute Delta Mass [ppm] Peptide Length Is z=1 Is z=2 Is z=3 Is z=4 Is z=5 Is z>5 # Missed Cleavages Log Peptides Matched Log Total Intensity Fraction Matched Intensity [%] Fragment Coverage Series A, B, C [%] Fragment Coverage Series X, Y, Z [%] Log Matched Fragment Series Intensities A, B, C Log Matched Fragment Series Intensities X, Y, Z Longest Sequence Series A, B, C Longest Sequence Series X, Y, Z IQR Fragment Delta Mass [Da] IQR Fragment Delta Mass [ppm] Mean Fragment Delta Mass [Da] Mean Fragment Delta Mass [ppm] Mean Absolute Fragment Delta Mass [Da] Mean Absolute Fragment Delta Mass [ppm] m0
  
06/25/2014 05:05 PM (3):Percolator: # first line contains normalized weights, second line the raw weights
  
06/25/2014 05:05 PM (3):Percolator: Obtained weights (only showing weights of first cross validation set)
  
06/25/2014 05:05 PM (3):Percolator: Iteration 10 : After the iteration step, 2637 target PSMs with q<0.01 were estimated by cross validation
  
06/25/2014 05:05 PM (3):Percolator: Iteration 9 : After the iteration step, 2638 target PSMs with q<0.01 were estimated by cross validation
  
06/25/2014 05:05 PM (3):Percolator: Iteration 8 : After the iteration step, 2639 target PSMs with q<0.01 were estimated by cross validation
  
06/25/2014 05:05 PM (3):Percolator: Iteration 7 : After the iteration step, 2638 target PSMs with q<0.01 were estimated by cross validation
  
06/25/2014 05:05 PM (3):Percolator: Iteration 6 : After the iteration step, 2635 target PSMs with q<0.01 were estimated by cross validation
  
06/25/2014 05:05 PM (3):Percolator: Iteration 5 : After the iteration step, 2630 target PSMs with q<0.01 were estimated by cross validation
  
06/25/2014 05:05 PM (3):Percolator: Iteration 4 : After the iteration step, 2622 target PSMs with q<0.01 were estimated by cross validation
  
06/25/2014 05:05 PM (3):Percolator: Iteration 3 : After the iteration step, 2613 target PSMs with q<0.01 were estimated by cross validation
  
06/25/2014 05:05 PM (3):Percolator: Iteration 2 : After the iteration step, 2600 target PSMs with q<0.01 were estimated by cross validation
  
06/25/2014 05:05 PM (3):Percolator: Iteration 1 : After the iteration step, 2540 target PSMs with q<0.01 were estimated by cross validation
  
06/25/2014 05:05 PM (3):Percolator: ---Training with Cpos selected by cross validation, Cneg selected by cross validation, fdr=0.01
  
06/25/2014 05:05 PM (3):Percolator: Reading in data and feature calculation took 9.781 cpu seconds or 10 seconds wall time
  
06/25/2014 05:05 PM (3):Percolator: Estimating 1917 over q=0.01 in initial direction
  
06/25/2014 05:05 PM (3):Percolator: Selected feature number 4 as initial search direction, could separate 1248 positives in that direction
  
06/25/2014 05:05 PM (3):Percolator: Selected feature number 4 as initial search direction, could separate 1252 positives in that direction
  
06/25/2014 05:05 PM (3):Percolator: Selected feature number 4 as initial search direction, could separate 1334 positives in that direction
  
06/25/2014 05:05 PM (3):Percolator: selecting cneg by cross validation
  
06/25/2014 05:05 PM (3):Percolator: selecting cpos by cross validation
  
06/25/2014 05:05 PM (3):Percolator: Train/test set contains 5919 positives and 6935 negatives, size ratio=0.853497 and pi0=1
  
06/25/2014 05:05 PM (3):Percolator: 31e77142-29e9-402c-9ec2-8468a9513af0 e39a792e-622c-452d-b49b-59809cad79d0 Delta Cn From Second PSM Binomial Score b8754504-e95e-476b-b9a4-454d4bb53aeb 1d91a87b-953a-4887-9f22-f75a497a3538 Delta Mass [Da] Delta Mass [ppm] Absolute Delta Mass [Da] Absolute Delta Mass [ppm] Peptide Length Is z=1 Is z=2 Is z=3 Is z=4 Is z=5 Is z>5 041eb6d5-e486-44a0-9bc1-19e25811c686 Log Peptides Matched Log Total Intensity Fraction Matched Intensity [%] Fragment Coverage Series A, B, C [%] Fragment Coverage Series X, Y, Z [%] Log Matched Fragment Series Intensities A, B, C Log Matched Fragment Series Intensities X, Y, Z Longest Sequence Series A, B, C Longest Sequence Series X, Y, Z IQR Fragment Delta Mass [Da] IQR Fragment Delta Mass [ppm] Mean Fragment Delta Mass [Da] Mean Fragment Delta Mass [ppm] Mean Absolute Fragment Delta Mass [Da] Mean Absolute Fragment Delta Mass [ppm]
  
06/25/2014 05:05 PM (3):Percolator: Features:
  
06/25/2014 05:05 PM (3):Percolator: enzyme=Trypsin
  
06/25/2014 05:05 PM (3):Percolator: Hyperparameters fdr=0.01, Cpos=0, Cneg=0, maxNiter=10
  
06/25/2014 05:05 PM (3):Percolator: Started Wed Jun 25 17:05:15 2014
  
06/25/2014 05:05 PM (3):Percolator: C:\Program Files\Thermo\Discoverer 1.4\Tools\Percolator\percolator.exe -X C:\ProgramData\Thermo\Discoverer 1.4\Scratch\a15b5cd1-64d2-4738-974b-4a52ac1565bc\output.xml -Z C:\ProgramData\Thermo\Discoverer 1.4\Scratch\a15b5cd1-64d2-4738-974b-4a52ac1565bc\input.xml
  
06/25/2014 05:05 PM (3):Percolator: Issued command:
  
06/25/2014 05:05 PM (3):Percolator: Department of Genome Sciences at the University of Washington.
  
06/25/2014 05:05 PM (3):Percolator: Written by Lukas K+�ll (lukall@u.washington.edu) in the
  
06/25/2014 05:05 PM (3):Percolator: Copyright (c) 2006-9 University of Washington. All rights reserved.
  
06/25/2014 05:05 PM (3):Percolator: Percolator version 2.04, Build Date Feb 1 2012 03:35:34
  
06/25/2014 05:05 PM (3):Percolator: Starting Percolator
  
06/25/2014 05:05 PM (3):Percolator: The input file contains 5919 peptides, 6935 decoy peptides and 33 features.
  
06/25/2014 05:05 PM (3):Percolator: Creating input file for SEQUEST (4) took 52.2 s.
  
06/25/2014 05:04 PM (3):Percolator: Start calculating features for peptides of SEQUEST (4)
  
06/25/2014 05:04 PM (2):Mascot: Total search time was 4 min 8 s.
  
06/25/2014 05:04 PM (3):Percolator: Performing percolator for Mascot (2) took 59.2 s.
  
06/25/2014 05:04 PM (2):Mascot: Search completed
  
06/25/2014 05:04 PM (2):Mascot: 405 protein(s) + 56 decoy proteins scored and inserted into result file in 0.9 s.
  
06/25/2014 05:04 PM (2):Mascot: 405 protein(s) scored
  
06/25/2014 05:04 PM (2):Mascot: Search result finalization started.
  
06/25/2014 05:04 PM (3):Percolator: Start reading Percolator results
  
06/25/2014 05:04 PM (3):Percolator: Processing took 6.988 cpu seconds or 6 seconds wall time
  
06/25/2014 05:04 PM (3):Percolator: PSMId score q-value posterior\_error\_prob peptide proteinIds
  
06/25/2014 05:04 PM (3):Percolator: Calibrating statistics - calculating Posterior error probabilities (PEPs)
  
06/25/2014 05:04 PM (3):Percolator: New pi\_0 estimate on merged list gives 1435 peptides over q=0.0100
  
06/25/2014 05:04 PM (3):Percolator: Calibrating statistics - calculating q values
  
06/25/2014 05:04 PM (3):Percolator: Selecting pi\_0=0.5787
  
06/25/2014 05:04 PM (3):Percolator: Tossing out "redundant" PSMs keeping only the best scoring PSM for each unique peptide.
  
06/25/2014 05:04 PM (3):Percolator: Merging results from 3 datasets
  
06/25/2014 05:04 PM (3):Percolator: Found 2485 target PSMs scoring over 1.0000% FDR level on testset
  
06/25/2014 05:04 PM (3):Percolator: 0.0437 -0.6048 0.0044 0.0022 0.0005 36.3895 0.0137 39.1496 -0.2530 -0.0369 0.0000 -0.2088 0.5450 -0.3008 -1.0713 -1.5233 -0.9154 0.0000 -1.1887 -0.0010 0.0142 0.0201 -0.1013 0.4246 0.1481 0.3837 -0.1779 -0.0018 -7.2592 0.0005 -3.1631 0.0017 0.5209
  
06/25/2014 05:04 PM (3):Percolator: 0.951 -0.1718 0.2166 0.0547 0.5299 0.5683 0.0732 0.3690 -0.6812 -0.3476 0.0000 -0.0965 0.2725 -0.1077 -0.1880 -0.1747 -1.0251 0.0000 -1.0906 -0.0150 0.2858 0.4622 -0.6748 1.4594 0.4721 1.5049 -0.0358 -0.6234 -0.6896 0.0894 -0.2841 0.3861 -1.9889
  
06/25/2014 05:04 PM (3):Percolator: IonScore Delta Cn From Second PSM Binomial Score Isolation Interference [%] MH+ [Da] Delta Mass [Da] Delta Mass [ppm] Absolute Delta Mass [Da] Absolute Delta Mass [ppm] Peptide Length Is z=1 Is z=2 Is z=3 Is z=4 Is z=5 Is z>5 # Missed Cleavages Log Peptides Matched Log Total Intensity Fraction Matched Intensity [%] Fragment Coverage Series A, B, C [%] Fragment Coverage Series X, Y, Z [%] Log Matched Fragment Series Intensities A, B, C Log Matched Fragment Series Intensities X, Y, Z Longest Sequence Series A, B, C Longest Sequence Series X, Y, Z IQR Fragment Delta Mass [Da] IQR Fragment Delta Mass [ppm] Mean Fragment Delta Mass [Da] Mean Fragment Delta Mass [ppm] Mean Absolute Fragment Delta Mass [Da] Mean Absolute Fragment Delta Mass [ppm] m0
  
06/25/2014 05:04 PM (3):Percolator: # first line contains normalized weights, second line the raw weights
  
06/25/2014 05:04 PM (3):Percolator: Obtained weights (only showing weights of first cross validation set)
  
06/25/2014 05:04 PM (3):Percolator: Iteration 10 : After the iteration step, 2548 target PSMs with q<0.01 were estimated by cross validation
  
06/25/2014 05:04 PM (3):Percolator: Iteration 9 : After the iteration step, 2548 target PSMs with q<0.01 were estimated by cross validation
  
06/25/2014 05:04 PM (3):Percolator: Iteration 8 : After the iteration step, 2549 target PSMs with q<0.01 were estimated by cross validation
  
06/25/2014 05:04 PM (3):Percolator: Iteration 7 : After the iteration step, 2548 target PSMs with q<0.01 were estimated by cross validation
  
06/25/2014 05:04 PM (3):Percolator: Iteration 6 : After the iteration step, 2546 target PSMs with q<0.01 were estimated by cross validation
  
06/25/2014 05:04 PM (3):Percolator: Iteration 5 : After the iteration step, 2545 target PSMs with q<0.01 were estimated by cross validation
  
06/25/2014 05:04 PM (3):Percolator: Iteration 4 : After the iteration step, 2542 target PSMs with q<0.01 were estimated by cross validation
  
06/25/2014 05:04 PM (3):Percolator: Iteration 3 : After the iteration step, 2537 target PSMs with q<0.01 were estimated by cross validation
  
06/25/2014 05:04 PM (3):Percolator: Iteration 2 : After the iteration step, 2525 target PSMs with q<0.01 were estimated by cross validation
  
06/25/2014 05:04 PM (3):Percolator: Iteration 1 : After the iteration step, 2487 target PSMs with q<0.01 were estimated by cross validation
  
06/25/2014 05:04 PM (3):Percolator: ---Training with Cpos selected by cross validation, Cneg selected by cross validation, fdr=0.01
  
06/25/2014 05:04 PM (3):Percolator: Reading in data and feature calculation took 7.098 cpu seconds or 8 seconds wall time
  
06/25/2014 05:04 PM (3):Percolator: Estimating 1990 over q=0.01 in initial direction
  
06/25/2014 05:04 PM (3):Percolator: Selected feature number 1 as initial search direction, could separate 1343 positives in that direction
  
06/25/2014 05:04 PM (3):Percolator: Selected feature number 1 as initial search direction, could separate 1286 positives in that direction
  
06/25/2014 05:04 PM (3):Percolator: Selected feature number 1 as initial search direction, could separate 1352 positives in that direction
  
06/25/2014 05:04 PM (3):Percolator: selecting cneg by cross validation
  
06/25/2014 05:04 PM (3):Percolator: selecting cpos by cross validation
  
06/25/2014 05:04 PM (3):Percolator: Train/test set contains 4918 positives and 4613 negatives, size ratio=1.06612 and pi0=1
  
06/25/2014 05:04 PM (3):Percolator: e6e22773-e9a6-4a26-9694-1ca77a797099 Delta Cn From Second PSM Binomial Score b8754504-e95e-476b-b9a4-454d4bb53aeb 1d91a87b-953a-4887-9f22-f75a497a3538 Delta Mass [Da] Delta Mass [ppm] Absolute Delta Mass [Da] Absolute Delta Mass [ppm] Peptide Length Is z=1 Is z=2 Is z=3 Is z=4 Is z=5 Is z>5 041eb6d5-e486-44a0-9bc1-19e25811c686 Log Peptides Matched Log Total Intensity Fraction Matched Intensity [%] Fragment Coverage Series A, B, C [%] Fragment Coverage Series X, Y, Z [%] Log Matched Fragment Series Intensities A, B, C Log Matched Fragment Series Intensities X, Y, Z Longest Sequence Series A, B, C Longest Sequence Series X, Y, Z IQR Fragment Delta Mass [Da] IQR Fragment Delta Mass [ppm] Mean Fragment Delta Mass [Da] Mean Fragment Delta Mass [ppm] Mean Absolute Fragment Delta Mass [Da] Mean Absolute Fragment Delta Mass [ppm]
  
06/25/2014 05:04 PM (3):Percolator: Features:
  
06/25/2014 05:04 PM (3):Percolator: enzyme=Trypsin
  
06/25/2014 05:04 PM (3):Percolator: Hyperparameters fdr=0.01, Cpos=0, Cneg=0, maxNiter=10
  
06/25/2014 05:04 PM (3):Percolator: Started Wed Jun 25 17:04:02 2014
  
06/25/2014 05:04 PM (3):Percolator: C:\Program Files\Thermo\Discoverer 1.4\Tools\Percolator\percolator.exe -X C:\ProgramData\Thermo\Discoverer 1.4\Scratch\77c6e45e-0e91-4cb7-8d09-40c0c299b617\output.xml -Z C:\ProgramData\Thermo\Discoverer 1.4\Scratch\77c6e45e-0e91-4cb7-8d09-40c0c299b617\input.xml
  
06/25/2014 05:04 PM (3):Percolator: Issued command:
  
06/25/2014 05:04 PM (3):Percolator: Department of Genome Sciences at the University of Washington.
  
06/25/2014 05:04 PM (3):Percolator: Written by Lukas K+�ll (lukall@u.washington.edu) in the
  
06/25/2014 05:04 PM (3):Percolator: Copyright (c) 2006-9 University of Washington. All rights reserved.
  
06/25/2014 05:04 PM (3):Percolator: Percolator version 2.04, Build Date Feb 1 2012 03:35:34
  
06/25/2014 05:04 PM (3):Percolator: Starting Percolator
  
06/25/2014 05:04 PM (3):Percolator: The input file contains 4918 peptides, 4613 decoy peptides and 32 features.
  
06/25/2014 05:04 PM (3):Percolator: Creating input file for Mascot (2) took 43.3 s.
  
06/25/2014 05:03 PM (3):Percolator: Start calculating features for peptides of Mascot (2)
  
06/25/2014 05:03 PM (2):Mascot: Used mascot server http://fenn.bham.ac.uk/mascot/ with Mascot version 2.4.1
  
06/25/2014 05:03 PM (2):Mascot: Sending 4427 peptide hits (18458 peptides) to result file
  
06/25/2014 05:03 PM (2):Mascot: Sending 3845 decoy peptide hits (15768 peptides) to result file
  
06/25/2014 05:03 PM (2):Mascot: Reading decoy results
  
06/25/2014 05:03 PM (2):Mascot: Start translating results
  
06/25/2014 05:03 PM (2):Mascot: Start mapping modifications
  
06/25/2014 05:03 PM (2):Mascot: Received 426 proteins from Mascot server
  
06/25/2014 05:03 PM (2):Mascot: Start mapping 426 proteins
  
06/25/2014 05:02 PM (2):Mascot: Start parsing results
  
06/25/2014 05:02 PM (2):Mascot: Received Mascot result file (filename=../data/20140625/F004280.dat)
  
06/25/2014 05:02 PM (2):Mascot: Mascot Server completed
  
06/25/2014 05:00 PM (2):Mascot: Mascot result on server (filename=../data/20140625/F004280.dat)
  
06/25/2014 04:59 PM (2):Mascot: Start searching 5365 spectra
  
06/25/2014 04:59 PM (4):SEQUEST: Sending 365 decoy peptide hits (2936 peptides) to result file
  
06/25/2014 04:59 PM (4):SEQUEST: Starting SEQUEST decoy search
  
06/25/2014 04:59 PM (4):SEQUEST: Sending 365 peptide hits (2919 peptides) to result file
  
06/25/2014 04:59 PM (4):SEQUEST: Starting SEQUEST (search spectra 5000 - 5365)
  
06/25/2014 04:59 PM (4):SEQUEST: Sending 1000 decoy peptide hits (7549 peptides) to result file
  
06/25/2014 04:58 PM (4):SEQUEST: Starting SEQUEST decoy search
  
06/25/2014 04:58 PM (4):SEQUEST: Sending 1000 peptide hits (7566 peptides) to result file
  
06/25/2014 04:58 PM (4):SEQUEST: Starting SEQUEST (search spectra 4000 - 5000)
  
06/25/2014 04:58 PM (4):SEQUEST: Sending 1000 decoy peptide hits (8243 peptides) to result file
  
06/25/2014 04:58 PM (4):SEQUEST: Starting SEQUEST decoy search
  
06/25/2014 04:58 PM (4):SEQUEST: Sending 1000 peptide hits (8292 peptides) to result file
  
06/25/2014 04:58 PM (4):SEQUEST: Starting SEQUEST (search spectra 3000 - 4000)
  
06/25/2014 04:57 PM (4):SEQUEST: Sending 1000 decoy peptide hits (9387 peptides) to result file
  
06/25/2014 04:57 PM (4):SEQUEST: Starting SEQUEST decoy search
  
06/25/2014 04:57 PM (4):SEQUEST: Sending 1000 peptide hits (9445 peptides) to result file
  
06/25/2014 04:57 PM (4):SEQUEST: Starting SEQUEST (search spectra 2000 - 3000)
  
06/25/2014 04:57 PM (4):SEQUEST: Sending 1000 decoy peptide hits (9918 peptides) to result file
  
06/25/2014 04:56 PM (4):SEQUEST: Starting SEQUEST decoy search
  
06/25/2014 04:56 PM (4):SEQUEST: Sending 1000 peptide hits (9885 peptides) to result file
  
06/25/2014 04:56 PM (4):SEQUEST: Starting SEQUEST (search spectra 1000 - 2000)
  
06/25/2014 04:56 PM (4):SEQUEST: Sending 1000 decoy peptide hits (9925 peptides) to result file
  
06/25/2014 04:56 PM (4):SEQUEST: Starting SEQUEST decoy search
  
06/25/2014 04:56 PM (4):SEQUEST: Sending 1000 peptide hits (9929 peptides) to result file
  
06/25/2014 04:55 PM (4):SEQUEST: Starting SEQUEST (search spectra 0 - 1000)
  
06/25/2014 04:55 PM (4):SEQUEST: There is already an adequate decoy FASTA index.
  
06/25/2014 04:55 PM (4):SEQUEST: Looking for existing decoy FASTA index.
  
06/25/2014 04:55 PM (4):SEQUEST: There is already an adequate target FASTA index.
  
06/25/2014 04:55 PM (4):SEQUEST: Looking for existing target FASTA index.
  
06/25/2014 04:55 PM (2):Mascot: Use mascot server http://fenn.bham.ac.uk/mascot/ with Mascot version 2.4.1
  
06/25/2014 04:55 PM (1):Spectrum Selector: Reading from File 1 of 1:E:\Jos\Water\_1.raw (8338 spectra total)
  
  
  
Top
