# Supplementary material for: Top-Down and Bottom-Up Identification of Proteins by Liquid Extraction Surface Analysis Mass Spectrometry of Healthy and Diseased Human Liver Tissue
Source: J Am Soc Mass Spectrom. 2014 Sep 3;25(11):1953–61. doi: 10.1007/s13361-014-0967-z (PMC4197381; doi:10.1007/s13361-014-0967-z)
Supplement: Supplementary file 6 — (ZIP 3005 kb) [file 13361_2014_967_MOESM6_ESM.zip › index.html]

Annotated spectra


## Annotated spectra of Methanol\_50\_1\_MC3.msf

go to Peptides  
go to Search Summary  

### Peptides

|  |  |  |  |  |  |  |  |  |  |  |  |  |  |  |  |  |  |  |  |  |  |  |  |  |  |  |  |  |  |  |  |  |
| --- | --- | --- | --- | --- | --- | --- | --- | --- | --- | --- | --- | --- | --- | --- | --- | --- | --- | --- | --- | --- | --- | --- | --- | --- | --- | --- | --- | --- | --- | --- | --- | --- |
|  | | | | | | | | | | | | | | | | | | | | | | | | | | | | | | | | |
| Confidence Sequence Activation Type Modifications IonScore XCorr ΔScore Rank Search Engine Charge Precursor m/z [Da] ΔM [ppm] First Scan Last Scan Annotated Spectrum Peak List  | | | | | | | | | | | | | | | | | | | | | | | | | | | | | | | | |
|  | | | | | | | | | | | | | | | | | | | | | | | | | | | | | | | | |
|  | **O00757 - Fructose-1,6-bisphosphatase isozyme 2 OS=Homo sapiens GN=FBP2 PE=1 SV=2 - [F16P2\_HUMAN]** | | | | | | | | | | | | | | | | | | | | | | | | | | | | | | |  |
|  | | | | | | | | | | | | | | | | | | | | | | | | | | | | | | | | |
|  | High |  | IYSLNEGYAK |  | CID |  |  |  | 32.02 |  |  |  | 1.00 |  | 1 |  | Mascot (2) |  | 2 |  | 579.29608 |  | 0.99 |  | 1101 |  | 1101 |  | Image |  | Peak List |  |
|  | | | | | | | | | | | | | | | | | | | | | | | | | | | | | | | | |
|  | High |  | IYSLNEGYAK |  | CID |  |  |  |  |  | 2.53 |  | 1.00 |  | 1 |  | SEQUEST (4) |  | 2 |  | 579.29657 |  | 1.83 |  | 1091 |  | 1091 |  | Image |  | Peak List |  |
|  | | | | | | | | | | | | | | | | | | | | | | | | | | | | | | | | |
|  | **P00505 - Aspartate aminotransferase, mitochondrial OS=Homo sapiens GN=GOT2 PE=1 SV=3 - [AATM\_HUMAN]** | | | | | | | | | | | | | | | | | | | | | | | | | | | | | | |  |
|  | | | | | | | | | | | | | | | | | | | | | | | | | | | | | | | | |
|  | High |  | NLFAFFDMAYQGFASGDGDKDAWAVR |  | CID |  |  |  | 52.57 |  |  |  | 1.00 |  | 1 |  | Mascot (2) |  | 3 |  | 967.11932 |  | 9.94 |  | 4030 |  | 4030 |  | Image |  | Peak List |  |
|  | | | | | | | | | | | | | | | | | | | | | | | | | | | | | | | | |
|  | High |  | NLFAFFDMAYQGFASGDGDKDAWAVR |  | CID |  |  |  |  |  | 2.95 |  | 1.00 |  | 1 |  | SEQUEST (4) |  | 3 |  | 967.11932 |  | 9.94 |  | 4030 |  | 4030 |  | Image |  | Peak List |  |
|  | | | | | | | | | | | | | | | | | | | | | | | | | | | | | | | | |
|  | **P04075 - Fructose-bisphosphate aldolase A OS=Homo sapiens GN=ALDOA PE=1 SV=2 - [ALDOA\_HUMAN]** | | | | | | | | | | | | | | | | | | | | | | | | | | | | | | |  |
|  | | | | | | | | | | | | | | | | | | | | | | | | | | | | | | | | |
|  | High |  | IGEHTPSALAIMENANVLAR |  | CID |  |  |  | 31.99 |  |  |  | 1.00 |  | 1 |  | Mascot (2) |  | 3 |  | 703.03870 |  | 2.36 |  | 2396 |  | 2396 |  | Image |  | Peak List |  |
|  | | | | | | | | | | | | | | | | | | | | | | | | | | | | | | | | |
|  | High |  | IGEHTPSALAIMENANVLAR |  | CID |  |  |  |  |  | 3.04 |  | 1.00 |  | 1 |  | SEQUEST (4) |  | 3 |  | 703.03870 |  | 2.36 |  | 2396 |  | 2396 |  | Image |  | Peak List |  |
|  | | | | | | | | | | | | | | | | | | | | | | | | | | | | | | | | |
|  | **P06576 - ATP synthase subunit beta, mitochondrial OS=Homo sapiens GN=ATP5B PE=1 SV=3 - [ATPB\_HUMAN]** | | | | | | | | | | | | | | | | | | | | | | | | | | | | | | |  |
|  | | | | | | | | | | | | | | | | | | | | | | | | | | | | | | | | |
|  | High |  | SLQDIIAILGMDELSEEDKLTVSR |  | CID |  |  |  | 62.51 |  |  |  | 1.00 |  | 1 |  | Mascot (2) |  | 3 |  | 892.47278 |  | 8.54 |  | 3988 |  | 3988 |  | Image |  | Peak List |  |
|  | | | | | | | | | | | | | | | | | | | | | | | | | | | | | | | | |
|  | High |  | SLQDIIAILGMDELSEEDKLTVSR |  | CID |  |  |  |  |  | 3.92 |  | 1.00 |  | 1 |  | SEQUEST (4) |  | 3 |  | 892.47278 |  | 8.54 |  | 3988 |  | 3988 |  | Image |  | Peak List |  |
|  | | | | | | | | | | | | | | | | | | | | | | | | | | | | | | | | |
|  | **P06744 - Glucose-6-phosphate isomerase OS=Homo sapiens GN=GPI PE=1 SV=4 - [G6PI\_HUMAN]** | | | | | | | | | | | | | | | | | | | | | | | | | | | | | | |  |
|  | | | | | | | | | | | | | | | | | | | | | | | | | | | | | | | | |
|  | High |  | IFVQGIIWDINSFDQWGVELGK |  | CID |  |  |  | 31.72 |  |  |  | 1.00 |  | 1 |  | Mascot (2) |  | 3 |  | 855.44672 |  | 2.76 |  | 4400 |  | 4400 |  | Image |  | Peak List |  |
|  | | | | | | | | | | | | | | | | | | | | | | | | | | | | | | | | |
|  | High |  | IFVQGIIWDINSFDQWGVELGK |  | CID |  |  |  |  |  | 5.09 |  | 1.00 |  | 1 |  | SEQUEST (4) |  | 3 |  | 855.44672 |  | 2.76 |  | 4400 |  | 4400 |  | Image |  | Peak List |  |
|  | | | | | | | | | | | | | | | | | | | | | | | | | | | | | | | | |
|  | **P0CG48 - Polyubiquitin-C OS=Homo sapiens GN=UBC PE=1 SV=3 - [UBC\_HUMAN]** | | | | | | | | | | | | | | | | | | | | | | | | | | | | | | |  |
|  | | | | | | | | | | | | | | | | | | | | | | | | | | | | | | | | |
|  | High |  | TITLEVEPSDTIENVK |  | CID |  |  |  | 38.90 |  |  |  | 1.00 |  | 1 |  | Mascot (2) |  | 2 |  | 894.46912 |  | 2.00 |  | 2004 |  | 2004 |  | Image |  | Peak List |  |
|  | | | | | | | | | | | | | | | | | | | | | | | | | | | | | | | | |
|  | High |  | TITLEVEPSDTIENVK |  | CID |  |  |  |  |  | 3.63 |  | 1.00 |  | 1 |  | SEQUEST (4) |  | 2 |  | 894.46912 |  | 2.00 |  | 2004 |  | 2004 |  | Image |  | Peak List |  |
|  | | | | | | | | | | | | | | | | | | | | | | | | | | | | | | | | |
|  | **P10620 - Microsomal glutathione S-transferase 1 OS=Homo sapiens GN=MGST1 PE=1 SV=1 - [MGST1\_HUMAN]** | | | | | | | | | | | | | | | | | | | | | | | | | | | | | | |  |
|  | | | | | | | | | | | | | | | | | | | | | | | | | | | | | | | | |
|  | High |  | VDLTQVMDDEVFMAFASYATIILSK |  | CID |  |  |  | 30.78 |  |  |  | 1.00 |  | 1 |  | Mascot (2) |  | 3 |  | 936.47388 |  | 6.68 |  | 5950 |  | 5950 |  | Image |  | Peak List |  |
|  | | | | | | | | | | | | | | | | | | | | | | | | | | | | | | | | |
|  | **P11310 - Medium-chain specific acyl-CoA dehydrogenase, mitochondrial OS=Homo sapiens GN=ACADM PE=1 SV=1 - [ACADM\_HUMAN]** | | | | | | | | | | | | | | | | | | | | | | | | | | | | | | |  |
|  | | | | | | | | | | | | | | | | | | | | | | | | | | | | | | | | |
|  | High |  | AFAGDIANQLATDAVQILGGNGFNTEYPVEK |  | CID |  |  |  | 24.95 |  |  |  | 1.00 |  | 1 |  | Mascot (2) |  | 3 |  | 1075.20862 |  | 6.37 |  | 4528 |  | 4528 |  | Image |  | Peak List |  |
|  | | | | | | | | | | | | | | | | | | | | | | | | | | | | | | | | |
|  | High |  | AFAGDIANQLATDAVQILGGNGFNTEYPVEK |  | CID |  |  |  |  |  | 4.85 |  | 1.00 |  | 1 |  | SEQUEST (4) |  | 3 |  | 1075.20862 |  | 6.37 |  | 4528 |  | 4528 |  | Image |  | Peak List |  |
|  | | | | | | | | | | | | | | | | | | | | | | | | | | | | | | | | |
|  | **P11586 - C-1-tetrahydrofolate synthase, cytoplasmic OS=Homo sapiens GN=MTHFD1 PE=1 SV=3 - [C1TC\_HUMAN]** | | | | | | | | | | | | | | | | | | | | | | | | | | | | | | |  |
|  | | | | | | | | | | | | | | | | | | | | | | | | | | | | | | | | |
|  | High |  | TAQFDISVASEIMAVLALTTSLEDMR |  | CID |  |  |  | 49.38 |  |  |  | 1.00 |  | 1 |  | Mascot (2) |  | 3 |  | 938.14832 |  | 6.94 |  | 5569 |  | 5569 |  | Image |  | Peak List |  |
|  | | | | | | | | | | | | | | | | | | | | | | | | | | | | | | | | |
|  | High |  | TAQFDISVASEIMAVLALTTSLEDMR |  | CID |  |  |  |  |  | 3.55 |  | 1.00 |  | 1 |  | SEQUEST (4) |  | 3 |  | 938.14832 |  | 6.94 |  | 5569 |  | 5569 |  | Image |  | Peak List |  |
|  | | | | | | | | | | | | | | | | | | | | | | | | | | | | | | | | |
|  | **P14174 - Macrophage migration inhibitory factor OS=Homo sapiens GN=MIF PE=1 SV=4 - [MIF\_HUMAN]** | | | | | | | | | | | | | | | | | | | | | | | | | | | | | | |  |
|  | | | | | | | | | | | | | | | | | | | | | | | | | | | | | | | | |
|  | High |  | PMFIVNTNVPR |  | CID |  |  |  | 40.91 |  |  |  | 1.00 |  | 1 |  | Mascot (2) |  | 2 |  | 644.34814 |  | 0.89 |  | 1865 |  | 1865 |  | Image |  | Peak List |  |
|  | | | | | | | | | | | | | | | | | | | | | | | | | | | | | | | | |
|  | **P20962 - Parathymosin OS=Homo sapiens GN=PTMS PE=1 SV=2 - [PTMS\_HUMAN]** | | | | | | | | | | | | | | | | | | | | | | | | | | | | | | |  |
|  | | | | | | | | | | | | | | | | | | | | | | | | | | | | | | | | |
|  | High |  | SVEAAAELSAK |  | CID |  |  |  | 38.65 |  |  |  | 1.00 |  | 1 |  | Mascot (2) |  | 2 |  | 538.28705 |  | 3.51 |  | 948 |  | 948 |  | Image |  | Peak List |  |
|  | | | | | | | | | | | | | | | | | | | | | | | | | | | | | | | | |
|  | High |  | SVEAAAELSAK |  | CID |  |  |  |  |  | 2.93 |  | 1.00 |  | 1 |  | SEQUEST (4) |  | 2 |  | 538.28705 |  | 3.51 |  | 948 |  | 948 |  | Image |  | Peak List |  |
|  | | | | | | | | | | | | | | | | | | | | | | | | | | | | | | | | |
|  | **P21695 - Glycerol-3-phosphate dehydrogenase [NAD(+)], cytoplasmic OS=Homo sapiens GN=GPD1 PE=1 SV=4 - [GPDA\_HUMAN]** | | | | | | | | | | | | | | | | | | | | | | | | | | | | | | |  |
|  | | | | | | | | | | | | | | | | | | | | | | | | | | | | | | | | |
|  | High |  | LPPNVVAVPDVVQAAEDADILIFVVPHQFIGK |  | CID |  |  |  | 51.26 |  |  |  | 1.00 |  | 1 |  | Mascot (2) |  | 3 |  | 1137.63977 |  | 9.48 |  | 4633 |  | 4633 |  | Image |  | Peak List |  |
|  | | | | | | | | | | | | | | | | | | | | | | | | | | | | | | | | |
|  | High |  | LPPNVVAVPDVVQAAEDADILIFVVPHQFIGK |  | CID |  |  |  |  |  | 4.43 |  | 1.00 |  | 1 |  | SEQUEST (4) |  | 3 |  | 1137.63977 |  | 9.48 |  | 4633 |  | 4633 |  | Image |  | Peak List |  |
|  | | | | | | | | | | | | | | | | | | | | | | | | | | | | | | | | |
|  | **P23378 - Glycine dehydrogenase [decarboxylating], mitochondrial OS=Homo sapiens GN=GLDC PE=1 SV=2 - [GCSP\_HUMAN]** | | | | | | | | | | | | | | | | | | | | | | | | | | | | | | |  |
|  | | | | | | | | | | | | | | | | | | | | | | | | | | | | | | | | |
|  | High |  | EFANIHPFVPLDQAQGYQQLFR |  | CID |  |  |  | 37.35 |  |  |  | 1.00 |  | 1 |  | Mascot (2) |  | 3 |  | 873.44507 |  | 2.11 |  | 2891 |  | 2891 |  | Image |  | Peak List |  |
|  | | | | | | | | | | | | | | | | | | | | | | | | | | | | | | | | |
|  | High |  | EFANIHPFVPLDQAQGYQQLFR |  | CID |  |  |  |  |  | 4.77 |  | 1.00 |  | 1 |  | SEQUEST (4) |  | 3 |  | 873.44507 |  | 2.11 |  | 2891 |  | 2891 |  | Image |  | Peak List |  |
|  | | | | | | | | | | | | | | | | | | | | | | | | | | | | | | | | |
|  | **P30039 - Phenazine biosynthesis-like domain-containing protein OS=Homo sapiens GN=PBLD PE=1 SV=2 - [PBLD\_HUMAN]** | | | | | | | | | | | | | | | | | | | | | | | | | | | | | | |  |
|  | | | | | | | | | | | | | | | | | | | | | | | | | | | | | | | | |
|  | High |  | LPIFIADAFTAR |  | CID |  |  |  | 58.75 |  |  |  | 1.00 |  | 1 |  | Mascot (2) |  | 2 |  | 667.87952 |  | 3.73 |  | 3113 |  | 3113 |  | Image |  | Peak List |  |
|  | | | | | | | | | | | | | | | | | | | | | | | | | | | | | | | | |
|  | High |  | LPIFIADAFTAR |  | CID |  |  |  |  |  | 3.20 |  | 1.00 |  | 1 |  | SEQUEST (4) |  | 2 |  | 667.87952 |  | 3.73 |  | 3113 |  | 3113 |  | Image |  | Peak List |  |
|  | | | | | | | | | | | | | | | | | | | | | | | | | | | | | | | | |
|  | **P30046 - D-dopachrome decarboxylase OS=Homo sapiens GN=DDT PE=1 SV=3 - [DOPD\_HUMAN]** | | | | | | | | | | | | | | | | | | | | | | | | | | | | | | |  |
|  | | | | | | | | | | | | | | | | | | | | | | | | | | | | | | | | |
|  | High |  | PFLELDTNLPANR |  | CID |  |  |  | 70.49 |  |  |  | 1.00 |  | 1 |  | Mascot (2) |  | 2 |  | 750.39801 |  | 2.27 |  | 2298 |  | 2298 |  | Image |  | Peak List |  |
|  | | | | | | | | | | | | | | | | | | | | | | | | | | | | | | | | |
|  | **P34913 - Bifunctional epoxide hydrolase 2 OS=Homo sapiens GN=EPHX2 PE=1 SV=2 - [HYES\_HUMAN]** | | | | | | | | | | | | | | | | | | | | | | | | | | | | | | |  |
|  | | | | | | | | | | | | | | | | | | | | | | | | | | | | | | | | |
|  | High |  | DLGMVTILVQDTDTALKELEK |  | CID |  |  |  | 35.22 |  |  |  | 1.00 |  | 1 |  | Mascot (2) |  | 3 |  | 778.08759 |  | 7.06 |  | 4457 |  | 4457 |  | Image |  | Peak List |  |
|  | | | | | | | | | | | | | | | | | | | | | | | | | | | | | | | | |
|  | High |  | DLGMVTILVQDTDTALKELEK |  | CID |  |  |  |  |  | 2.27 |  | 1.00 |  | 1 |  | SEQUEST (4) |  | 3 |  | 778.08844 |  | 8.16 |  | 4473 |  | 4473 |  | Image |  | Peak List |  |
|  | | | | | | | | | | | | | | | | | | | | | | | | | | | | | | | | |
|  | **P35914 - Hydroxymethylglutaryl-CoA lyase, mitochondrial OS=Homo sapiens GN=HMGCL PE=1 SV=2 - [HMGCL\_HUMAN]** | | | | | | | | | | | | | | | | | | | | | | | | | | | | | | |  |
|  | | | | | | | | | | | | | | | | | | | | | | | | | | | | | | | | |
|  | High |  | EVVIFGAASELFTK |  | CID |  |  |  | 67.77 |  |  |  | 1.00 |  | 1 |  | Mascot (2) |  | 2 |  | 755.91400 |  | 3.62 |  | 3098 |  | 3098 |  | Image |  | Peak List |  |
|  | | | | | | | | | | | | | | | | | | | | | | | | | | | | | | | | |
|  | High |  | EVVIFGAASELFTK |  | CID |  |  |  |  |  | 4.62 |  | 1.00 |  | 1 |  | SEQUEST (4) |  | 2 |  | 755.91400 |  | 3.62 |  | 3098 |  | 3098 |  | Image |  | Peak List |  |
|  | | | | | | | | | | | | | | | | | | | | | | | | | | | | | | | | |
|  | **P42126 - Enoyl-CoA delta isomerase 1, mitochondrial OS=Homo sapiens GN=ECI1 PE=1 SV=1 - [ECI1\_HUMAN]** | | | | | | | | | | | | | | | | | | | | | | | | | | | | | | |  |
|  | | | | | | | | | | | | | | | | | | | | | | | | | | | | | | | | |
|  | High |  | VLVEPDAGAGVAVMK |  | CID |  |  |  | 56.68 |  |  |  | 1.00 |  | 1 |  | Mascot (2) |  | 2 |  | 728.39880 |  | 1.82 |  | 1681 |  | 1681 |  | Image |  | Peak List |  |
|  | | | | | | | | | | | | | | | | | | | | | | | | | | | | | | | | |
|  | High |  | VLVEPDAGAGVAVMK |  | CID |  |  |  |  |  | 3.65 |  | 1.00 |  | 1 |  | SEQUEST (4) |  | 2 |  | 728.39880 |  | 1.82 |  | 1681 |  | 1681 |  | Image |  | Peak List |  |
|  | | | | | | | | | | | | | | | | | | | | | | | | | | | | | | | | |
|  | **P51858 - Hepatoma-derived growth factor OS=Homo sapiens GN=HDGF PE=1 SV=1 - [HDGF\_HUMAN]** | | | | | | | | | | | | | | | | | | | | | | | | | | | | | | |  |
|  | | | | | | | | | | | | | | | | | | | | | | | | | | | | | | | | |
|  | High |  | GPPQEEEEEEDEEEEATKEDAEAPGIR |  | CID |  |  |  | 36.23 |  |  |  | 1.00 |  | 1 |  | Mascot (2) |  | 3 |  | 1014.76770 |  | 2.16 |  | 1196 |  | 1196 |  | Image |  | Peak List |  |
|  | | | | | | | | | | | | | | | | | | | | | | | | | | | | | | | | |
|  | High |  | GPPQEEEEEEDEEEEATKEDAEAPGIR |  | CID |  |  |  |  |  | 3.76 |  | 1.00 |  | 1 |  | SEQUEST (4) |  | 3 |  | 1014.76556 |  | 0.06 |  | 1203 |  | 1203 |  | Image |  | Peak List |  |
|  | | | | | | | | | | | | | | | | | | | | | | | | | | | | | | | | |
|  | **P55263 - Adenosine kinase OS=Homo sapiens GN=ADK PE=1 SV=2 - [ADK\_HUMAN]** | | | | | | | | | | | | | | | | | | | | | | | | | | | | | | |  |
|  | | | | | | | | | | | | | | | | | | | | | | | | | | | | | | | | |
|  | High |  | ENILFGMGNPLLDISAVVDKDFLDK |  | CID |  |  |  | 41.87 |  |  |  | 1.00 |  | 1 |  | Mascot (2) |  | 3 |  | 921.82129 |  | 7.89 |  | 4524 |  | 4524 |  | Image |  | Peak List |  |
|  | | | | | | | | | | | | | | | | | | | | | | | | | | | | | | | | |
|  | High |  | ENILFGMGNPLLDISAVVDKDFLDK |  | CID |  |  |  |  |  | 2.29 |  | 1.00 |  | 1 |  | SEQUEST (4) |  | 3 |  | 921.82129 |  | 7.89 |  | 4524 |  | 4524 |  | Image |  | Peak List |  |
|  | | | | | | | | | | | | | | | | | | | | | | | | | | | | | | | | |
|  | **P61604 - 10 kDa heat shock protein, mitochondrial OS=Homo sapiens GN=HSPE1 PE=1 SV=2 - [CH10\_HUMAN]** | | | | | | | | | | | | | | | | | | | | | | | | | | | | | | |  |
|  | | | | | | | | | | | | | | | | | | | | | | | | | | | | | | | | |
|  | High |  | VLQATVVAVGSGSK |  | CID |  |  |  | 88.65 |  |  |  | 1.00 |  | 1 |  | Mascot (2) |  | 2 |  | 658.38361 |  | 1.42 |  | 1130 |  | 1130 |  | Image |  | Peak List |  |
|  | | | | | | | | | | | | | | | | | | | | | | | | | | | | | | | | |
|  | High |  | VLQATVVAVGSGSK |  | CID |  |  |  |  |  | 4.35 |  | 1.00 |  | 1 |  | SEQUEST (4) |  | 2 |  | 658.38361 |  | 1.42 |  | 1130 |  | 1130 |  | Image |  | Peak List |  |
|  | | | | | | | | | | | | | | | | | | | | | | | | | | | | | | | | |
|  | **P62158 - Calmodulin OS=Homo sapiens GN=CALM1 PE=1 SV=2 - [CALM\_HUMAN]** | | | | | | | | | | | | | | | | | | | | | | | | | | | | | | |  |
|  | | | | | | | | | | | | | | | | | | | | | | | | | | | | | | | | |
|  | High |  | EAFSLFDKDGDGTITTK |  | CID |  |  |  | 59.19 |  |  |  | 1.00 |  | 1 |  | Mascot (2) |  | 2 |  | 922.95135 |  | 2.22 |  | 1834 |  | 1834 |  | Image |  | Peak List |  |
|  | | | | | | | | | | | | | | | | | | | | | | | | | | | | | | | | |
|  | High |  | EAFSLFDKDGDGTITTK |  | CID |  |  |  |  |  | 3.92 |  | 1.00 |  | 1 |  | SEQUEST (4) |  | 2 |  | 922.95135 |  | 2.22 |  | 1834 |  | 1834 |  | Image |  | Peak List |  |
|  | | | | | | | | | | | | | | | | | | | | | | | | | | | | | | | | |
|  | **P63241 - Eukaryotic translation initiation factor 5A-1 OS=Homo sapiens GN=EIF5A PE=1 SV=2 - [IF5A1\_HUMAN]** | | | | | | | | | | | | | | | | | | | | | | | | | | | | | | |  |
|  | | | | | | | | | | | | | | | | | | | | | | | | | | | | | | | | |
|  | High |  | YDCGEEILITVLSAMTEEAAVAIK |  | CID |  |  |  | 22.83 |  |  |  | 1.00 |  | 1 |  | Mascot (2) |  | 3 |  | 857.10400 |  | 7.69 |  | 5654 |  | 5654 |  | Image |  | Peak List |  |
|  | | | | | | | | | | | | | | | | | | | | | | | | | | | | | | | | |
|  | High |  | YDCGEEILITVLSAMTEEAAVAIK |  | CID |  |  |  |  |  | 3.53 |  | 1.00 |  | 1 |  | SEQUEST (4) |  | 3 |  | 857.10400 |  | 7.69 |  | 5654 |  | 5654 |  | Image |  | Peak List |  |
|  | | | | | | | | | | | | | | | | | | | | | | | | | | | | | | | | |
|  | **P68104 - Elongation factor 1-alpha 1 OS=Homo sapiens GN=EEF1A1 PE=1 SV=1 - [EF1A1\_HUMAN]** | | | | | | | | | | | | | | | | | | | | | | | | | | | | | | |  |
|  | | | | | | | | | | | | | | | | | | | | | | | | | | | | | | | | |
|  | High |  | NMITGTSQADCAVLIVAAGVGEFEAGISK |  | CID |  |  |  | 58.83 |  |  |  | 1.00 |  | 1 |  | Mascot (2) |  | 3 |  | 951.48395 |  | 7.11 |  | 4509 |  | 4509 |  | Image |  | Peak List |  |
|  | | | | | | | | | | | | | | | | | | | | | | | | | | | | | | | | |
|  | High |  | NMITGTSQADCAVLIVAAGVGEFEAGISK |  | CID |  |  |  |  |  | 5.64 |  | 1.00 |  | 1 |  | SEQUEST (4) |  | 3 |  | 951.48395 |  | 7.11 |  | 4509 |  | 4509 |  | Image |  | Peak List |  |
|  | | | | | | | | | | | | | | | | | | | | | | | | | | | | | | | | |
|  | **Q06323 - Proteasome activator complex subunit 1 OS=Homo sapiens GN=PSME1 PE=1 SV=1 - [PSME1\_HUMAN]** | | | | | | | | | | | | | | | | | | | | | | | | | | | | | | |  |
|  | | | | | | | | | | | | | | | | | | | | | | | | | | | | | | | | |
|  | High |  | DVIEQLNLVTTWLQLQIPR |  | CID |  |  |  | 25.34 |  |  |  | 1.00 |  | 1 |  | Mascot (2) |  | 3 |  | 760.43579 |  | 7.43 |  | 5160 |  | 5160 |  | Image |  | Peak List |  |
|  | | | | | | | | | | | | | | | | | | | | | | | | | | | | | | | | |
|  | High |  | DVIEQLNLVTTWLQLQIPR |  | CID |  |  |  |  |  | 3.59 |  | 1.00 |  | 1 |  | SEQUEST (4) |  | 3 |  | 760.43579 |  | 7.43 |  | 5160 |  | 5160 |  | Image |  | Peak List |  |
|  | | | | | | | | | | | | | | | | | | | | | | | | | | | | | | | | |
|  | **Q08AH3 - Acyl-coenzyme A synthetase ACSM2A, mitochondrial OS=Homo sapiens GN=ACSM2A PE=1 SV=2 - [ACS2A\_HUMAN]** | | | | | | | | | | | | | | | | | | | | | | | | | | | | | | |  |
|  | | | | | | | | | | | | | | | | | | | | | | | | | | | | | | | | |
|  | High |  | FNFASDVLDHWADMEK |  | CID |  |  |  | 24.84 |  |  |  | 1.00 |  | 1 |  | Mascot (2) |  | 3 |  | 642.29163 |  | 3.53 |  | 3047 |  | 3047 |  | Image |  | Peak List |  |
|  | | | | | | | | | | | | | | | | | | | | | | | | | | | | | | | | |
|  | High |  | FNFASDVLDHWADMEK |  | CID |  |  |  |  |  | 1.65 |  | 1.00 |  | 1 |  | SEQUEST (4) |  | 3 |  | 642.29163 |  | 3.53 |  | 3047 |  | 3047 |  | Image |  | Peak List |  |
|  | | | | | | | | | | | | | | | | | | | | | | | | | | | | | | | | |
|  | **Q0PNE2 - Elongator complex protein 6 OS=Homo sapiens GN=ELP6 PE=1 SV=1 - [ELP6\_HUMAN]** | | | | | | | | | | | | | | | | | | | | | | | | | | | | | | |  |
|  | | | | | | | | | | | | | | | | | | | | | | | | | | | | | | | | |
|  | High |  | EPHPLQFLREANAGNLKPLFEFVREALKPVDSGEAR |  | CID |  |  |  | 23.89 |  |  |  | 1.00 |  | 1 |  | Mascot (2) |  | 4 |  | 1019.54559 |  | 2.24 |  | 4452 |  | 4452 |  | Image |  | Peak List |  |
|  | | | | | | | | | | | | | | | | | | | | | | | | | | | | | | | | |
|  | **Q13011 - Delta(3,5)-Delta(2,4)-dienoyl-CoA isomerase, mitochondrial OS=Homo sapiens GN=ECH1 PE=1 SV=2 - [ECH1\_HUMAN]** | | | | | | | | | | | | | | | | | | | | | | | | | | | | | | |  |
|  | | | | | | | | | | | | | | | | | | | | | | | | | | | | | | | | |
|  | High |  | VFPDKEVMLDAALALAAEISSK |  | CID |  |  |  | 20.58 |  |  |  | 1.00 |  | 1 |  | Mascot (2) |  | 3 |  | 773.42255 |  | 9.38 |  | 4064 |  | 4064 |  | Image |  | Peak List |  |
|  | | | | | | | | | | | | | | | | | | | | | | | | | | | | | | | | |
|  | High |  | VFPDKEVMLDAALALAAEISSK |  | CID |  |  |  |  |  | 2.73 |  | 1.00 |  | 1 |  | SEQUEST (4) |  | 3 |  | 773.42255 |  | 9.38 |  | 4064 |  | 4064 |  | Image |  | Peak List |  |
|  | | | | | | | | | | | | | | | | | | | | | | | | | | | | | | | | |
|  | **Q14697 - Neutral alpha-glucosidase AB OS=Homo sapiens GN=GANAB PE=1 SV=3 - [GANAB\_HUMAN]** | | | | | | | | | | | | | | | | | | | | | | | | | | | | | | |  |
|  | | | | | | | | | | | | | | | | | | | | | | | | | | | | | | | | |
|  | High |  | DLGIFWLNAAETWVDISSNTAGK |  | CID |  |  |  | 35.55 |  |  |  | 1.00 |  | 1 |  | Mascot (2) |  | 3 |  | 836.75812 |  | 7.63 |  | 5004 |  | 5004 |  | Image |  | Peak List |  |
|  | | | | | | | | | | | | | | | | | | | | | | | | | | | | | | | | |
|  | High |  | DLGIFWLNAAETWVDISSNTAGK |  | CID |  |  |  |  |  | 4.16 |  | 1.00 |  | 1 |  | SEQUEST (4) |  | 3 |  | 836.75812 |  | 7.63 |  | 5004 |  | 5004 |  | Image |  | Peak List |  |
|  | | | | | | | | | | | | | | | | | | | | | | | | | | | | | | | | |
|  | **Q96IU4 - Alpha/beta hydrolase domain-containing protein 14B OS=Homo sapiens GN=ABHD14B PE=1 SV=1 - [ABHEB\_HUMAN]** | | | | | | | | | | | | | | | | | | | | | | | | | | | | | | |  |
|  | | | | | | | | | | | | | | | | | | | | | | | | | | | | | | | | |
|  | High |  | TPALIVYGDQDPMGQTSFEHLK |  | CID |  |  |  | 63.55 |  |  |  | 1.00 |  | 1 |  | Mascot (2) |  | 3 |  | 816.40411 |  | 2.68 |  | 2167 |  | 2167 |  | Image |  | Peak List |  |
|  | | | | | | | | | | | | | | | | | | | | | | | | | | | | | | | | |
|  | High |  | TPALIVYGDQDPMGQTSFEHLK |  | CID |  |  |  |  |  | 4.39 |  | 1.00 |  | 1 |  | SEQUEST (4) |  | 3 |  | 816.40411 |  | 2.68 |  | 2167 |  | 2167 |  | Image |  | Peak List |  |
|  | | | | | | | | | | | | | | | | | | | | | | | | | | | | | | | | |
|  | **Q99714 - 3-hydroxyacyl-CoA dehydrogenase type-2 OS=Homo sapiens GN=HSD17B10 PE=1 SV=3 - [HCD2\_HUMAN]** | | | | | | | | | | | | | | | | | | | | | | | | | | | | | | |  |
|  | | | | | | | | | | | | | | | | | | | | | | | | | | | | | | | | |
|  | High |  | LGDPAEYAHLVQAIIENPFLNGEVIR |  | CID |  |  |  | 44.72 |  |  |  | 1.00 |  | 1 |  | Mascot (2) |  | 3 |  | 960.18152 |  | 6.98 |  | 4250 |  | 4250 |  | Image |  | Peak List |  |
|  | | | | | | | | | | | | | | | | | | | | | | | | | | | | | | | | |
|  | High |  | LGDPAEYAHLVQAIIENPFLNGEVIR |  | CID |  |  |  |  |  | 5.00 |  | 1.00 |  | 1 |  | SEQUEST (4) |  | 3 |  | 960.18152 |  | 6.98 |  | 4250 |  | 4250 |  | Image |  | Peak List |  |
|  | | | | | | | | | | | | | | | | | | | | | | | | | | | | | | | | |
|  | **Q9BWD1 - Acetyl-CoA acetyltransferase, cytosolic OS=Homo sapiens GN=ACAT2 PE=1 SV=2 - [THIC\_HUMAN]** | | | | | | | | | | | | | | | | | | | | | | | | | | | | | | |  |
|  | | | | | | | | | | | | | | | | | | | | | | | | | | | | | | | | |
|  | High |  | AGWSLEDVDIFEINEAFAAVSAAIVK |  | CID |  |  |  | 55.22 |  |  |  | 1.00 |  | 1 |  | Mascot (2) |  | 3 |  | 922.48016 |  | 8.15 |  | 5116 |  | 5116 |  | Image |  | Peak List |  |
|  | | | | | | | | | | | | | | | | | | | | | | | | | | | | | | | | |
|  | High |  | AGWSLEDVDIFEINEAFAAVSAAIVK |  | CID |  |  |  |  |  | 5.81 |  | 1.00 |  | 1 |  | SEQUEST (4) |  | 3 |  | 922.48016 |  | 8.15 |  | 5116 |  | 5116 |  | Image |  | Peak List |  |
|  | | | | | | | | | | | | | | | | | | | | | | | | | | | | | | | | |
|  | **Q9BX68 - Histidine triad nucleotide-binding protein 2, mitochondrial OS=Homo sapiens GN=HINT2 PE=1 SV=1 - [HINT2\_HUMAN]** | | | | | | | | | | | | | | | | | | | | | | | | | | | | | | |  |
|  | | | | | | | | | | | | | | | | | | | | | | | | | | | | | | | | |
|  | High |  | ISQAEEEDQQLLGHLLLVAK |  | CID |  |  |  | 38.33 |  |  |  | 1.00 |  | 1 |  | Mascot (2) |  | 3 |  | 745.40948 |  | 4.97 |  | 2809 |  | 2809 |  | Image |  | Peak List |  |
|  | | | | | | | | | | | | | | | | | | | | | | | | | | | | | | | | |
|  | High |  | ISQAEEEDQQLLGHLLLVAK |  | CID |  |  |  |  |  | 3.31 |  | 1.00 |  | 1 |  | SEQUEST (4) |  | 3 |  | 745.40948 |  | 4.97 |  | 2809 |  | 2809 |  | Image |  | Peak List |  |
|  | | | | | | | | | | | | | | | | | | | | | | | | | | | | | | | | |
|  | **Q9H0W9 - Ester hydrolase C11orf54 OS=Homo sapiens GN=C11orf54 PE=1 SV=1 - [CK054\_HUMAN]** | | | | | | | | | | | | | | | | | | | | | | | | | | | | | | |  |
|  | | | | | | | | | | | | | | | | | | | | | | | | | | | | | | | | |
|  | High |  | IAEVGGVPYLLPLVNQK |  | CID |  |  |  | 23.77 |  |  |  | 1.00 |  | 1 |  | Mascot (2) |  | 2 |  | 905.53101 |  | 4.06 |  | 3011 |  | 3011 |  | Image |  | Peak List |  |
|  | | | | | | | | | | | | | | | | | | | | | | | | | | | | | | | | |
|  | High |  | IAEVGGVPYLLPLVNQK |  | CID |  |  |  |  |  | 4.12 |  | 1.00 |  | 1 |  | SEQUEST (4) |  | 2 |  | 905.53101 |  | 4.06 |  | 3011 |  | 3011 |  | Image |  | Peak List |  |
|  | | | | | | | | | | | | | | | | | | | | | | | | | | | | | | | | |
|  | **Q9H2A2 - Aldehyde dehydrogenase family 8 member A1 OS=Homo sapiens GN=ALDH8A1 PE=1 SV=1 - [AL8A1\_HUMAN]** | | | | | | | | | | | | | | | | | | | | | | | | | | | | | | |  |
|  | | | | | | | | | | | | | | | | | | | | | | | | | | | | | | | | |
|  | High |  | VLNQVADLLEQSLEEFAQAESK |  | CID |  |  |  | 39.31 |  |  |  | 1.00 |  | 1 |  | Mascot (2) |  | 3 |  | 821.08789 |  | 1.35 |  | 4543 |  | 4543 |  | Image |  | Peak List |  |
|  | | | | | | | | | | | | | | | | | | | | | | | | | | | | | | | | |
|  | High |  | VLNQVADLLEQSLEEFAQAESK |  | CID |  |  |  |  |  | 4.87 |  | 1.00 |  | 1 |  | SEQUEST (4) |  | 3 |  | 821.08789 |  | 1.35 |  | 4543 |  | 4543 |  | Image |  | Peak List |  |
|  | | | | | | | | | | | | | | | | | | | | | | | | | | | | | | | | |
|  | **Q9Y2S2 - Lambda-crystallin homolog OS=Homo sapiens GN=CRYL1 PE=1 SV=3 - [CRYL1\_HUMAN]** | | | | | | | | | | | | | | | | | | | | | | | | | | | | | | |  |
|  | | | | | | | | | | | | | | | | | | | | | | | | | | | | | | | | |
|  | High |  | LVEEGIVSPSDLDLVMSEGLGMR |  | CID |  |  |  | 28.73 |  |  |  | 1.00 |  | 1 |  | Mascot (2) |  | 3 |  | 816.08496 |  | 8.09 |  | 3667 |  | 3667 |  | Image |  | Peak List |  |
|  | | | | | | | | | | | | | | | | | | | | | | | | | | | | | | | | |
|  | High |  | LVEEGIVSPSDLDLVMSEGLGMR |  | CID |  |  |  |  |  | 3.93 |  | 1.00 |  | 1 |  | SEQUEST (4) |  | 3 |  | 816.08496 |  | 8.09 |  | 3667 |  | 3667 |  | Image |  | Peak List |  |
|  | | | | | | | | | | | | | | | | | | | | | | | | | | | | | | | | |
|  | **B8ZZZ0 - 3-hydroxyisobutyryl-CoA hydrolase, mitochondrial (Fragment) OS=Homo sapiens GN=HIBCH PE=4 SV=1 - [B8ZZZ0\_HUMAN]** | | | | | | | | | | | | | | | | | | | | | | | | | | | | | | |  |
|  | | | | | | | | | | | | | | | | | | | | | | | | | | | | | | | | |
|  | High |  | KWEQDPETFLIIIK |  | CID |  |  |  |  |  | 2.57 |  | 1.00 |  | 1 |  | SEQUEST (4) |  | 3 |  | 587.32800 |  | 3.68 |  | 2666 |  | 2666 |  | Image |  | Peak List |  |
|  | | | | | | | | | | | | | | | | | | | | | | | | | | | | | | | | |
|  | **C9JDL1 - Ketohexokinase (Fragment) OS=Homo sapiens GN=KHK PE=4 SV=1 - [C9JDL1\_HUMAN]** | | | | | | | | | | | | | | | | | | | | | | | | | | | | | | |  |
|  | | | | | | | | | | | | | | | | | | | | | | | | | | | | | | | | |
|  | High |  | TIVLHDTSLPDVSATDFEK |  | CID |  |  |  |  |  | 2.82 |  | 1.00 |  | 1 |  | SEQUEST (4) |  | 3 |  | 696.69019 |  | 3.05 |  | 2067 |  | 2067 |  | Image |  | Peak List |  |
|  | | | | | | | | | | | | | | | | | | | | | | | | | | | | | | | | |
|  | **D6RA51 - Toll-like receptor 3 (Fragment) OS=Homo sapiens GN=TLR3 PE=4 SV=1 - [D6RA51\_HUMAN]** | | | | | | | | | | | | | | | | | | | | | | | | | | | | | | |  |
|  | | | | | | | | | | | | | | | | | | | | | | | | | | | | | | | | |
|  | High |  | TFAFCTNLTELHLmSNSIQK |  | CID |  | M14(Oxidation) |  |  |  | 1.78 |  | 1.00 |  | 1 |  | SEQUEST (4) |  | 3 |  | 772.04480 |  | -0.37 |  | 4039 |  | 4039 |  | Image |  | Peak List |  |
|  | | | | | | | | | | | | | | | | | | | | | | | | | | | | | | | | |
|  | **E7EU82 - SPARC-like protein 1 (Fragment) OS=Homo sapiens GN=SPARCL1 PE=4 SV=2 - [E7EU82\_HUMAN]** | | | | | | | | | | | | | | | | | | | | | | | | | | | | | | |  |
|  | | | | | | | | | | | | | | | | | | | | | | | | | | | | | | | | |
|  | High |  | MKTGLFFLCLLGTAAAIPTNAR |  | CID |  |  |  |  |  | 1.99 |  | 1.00 |  | 1 |  | SEQUEST (4) |  | 3 |  | 770.42725 |  | 7.02 |  | 3889 |  | 3889 |  | Image |  | Peak List |  |
|  | | | | | | | | | | | | | | | | | | | | | | | | | | | | | | | | |
|  | **E7EUE6 - Sodium channel protein type 3 subunit alpha (Fragment) OS=Homo sapiens GN=SCN3A PE=4 SV=1 - [E7EUE6\_HUMAN]** | | | | | | | | | | | | | | | | | | | | | | | | | | | | | | |  |
|  | | | | | | | | | | | | | | | | | | | | | | | | | | | | | | | | |
|  | High |  | CLQWPPSDSAFETNTTSYFNGTmDSNGTFVNVTMSTFNWK |  | CID |  | M23(Oxidation) |  |  |  | 1.52 |  | 1.00 |  | 1 |  | SEQUEST (4) |  | 7 |  | 649.72162 |  | 9.68 |  | 7467 |  | 7467 |  | Image |  | Peak List |  |
|  | | | | | | | | | | | | | | | | | | | | | | | | | | | | | | | | |
|  | **F2Z393 - Transaldolase OS=Homo sapiens GN=TALDO1 PE=3 SV=1 - [F2Z393\_HUMAN]** | | | | | | | | | | | | | | | | | | | | | | | | | | | | | | |  |
|  | | | | | | | | | | | | | | | | | | | | | | | | | | | | | | | | |
|  | High |  | SYEPLEDPGVK |  | CID |  |  |  |  |  | 2.12 |  | 1.00 |  | 1 |  | SEQUEST (4) |  | 2 |  | 617.30420 |  | 1.06 |  | 1146 |  | 1146 |  | Image |  | Peak List |  |
|  | | | | | | | | | | | | | | | | | | | | | | | | | | | | | | | | |
|  | **F5H1J3 - Kelch-like protein 1 OS=Homo sapiens GN=KLHL1 PE=4 SV=1 - [F5H1J3\_HUMAN]** | | | | | | | | | | | | | | | | | | | | | | | | | | | | | | |  |
|  | | | | | | | | | | | | | | | | | | | | | | | | | | | | | | | | |
|  | High |  | EDTIENLLAAACLLQLPQVVEVCCHFLmK |  | CID |  | M28(Oxidation) |  |  |  | 1.44 |  | 1.00 |  | 1 |  | SEQUEST (4) |  | 4 |  | 815.65875 |  | -3.17 |  | 5730 |  | 5730 |  | Image |  | Peak List |  |
|  | | | | | | | | | | | | | | | | | | | | | | | | | | | | | | | | |
|  | **F5H4W0 - Malic enzyme OS=Homo sapiens GN=ME1 PE=3 SV=1 - [F5H4W0\_HUMAN]** | | | | | | | | | | | | | | | | | | | | | | | | | | | | | | |  |
|  | | | | | | | | | | | | | | | | | | | | | | | | | | | | | | | | |
|  | High |  | LSDQTILFQGAGEAALGIAHLIVMALEK |  | CID |  |  |  |  |  | 2.83 |  | 1.00 |  | 1 |  | SEQUEST (4) |  | 3 |  | 970.53882 |  | 7.30 |  | 5367 |  | 5367 |  | Image |  | Peak List |  |
|  | | | | | | | | | | | | | | | | | | | | | | | | | | | | | | | | |
|  | **O00748-2 - Isoform 2 of Cocaine esterase OS=Homo sapiens GN=CES2 - [EST2\_HUMAN]** | | | | | | | | | | | | | | | | | | | | | | | | | | | | | | |  |
|  | | | | | | | | | | | | | | | | | | | | | | | | | | | | | | | | |
|  | High |  | HPQELLASADFQPVPSIVGVNNNEFGWLIPK |  | CID |  |  |  |  |  | 3.41 |  | 1.00 |  | 1 |  | SEQUEST (4) |  | 3 |  | 1139.59692 |  | 3.60 |  | 3554 |  | 3554 |  | Image |  | Peak List |  |
|  | | | | | | | | | | | | | | | | | | | | | | | | | | | | | | | | |
|  | **O15120-2 - Isoform 2 of 1-acyl-sn-glycerol-3-phosphate acyltransferase beta OS=Homo sapiens GN=AGPAT2 - [PLCB\_HUMAN]** | | | | | | | | | | | | | | | | | | | | | | | | | | | | | | |  |
|  | | | | | | | | | | | | | | | | | | | | | | | | | | | | | | | | |
|  | High |  | LQEARPCVIVSNHQSILDmMGLMEVLPERCVQIAK |  | CID |  | M19(Oxidation) |  |  |  | 2.39 |  | 1.00 |  | 1 |  | SEQUEST (4) |  | 4 |  | 992.51013 |  | 2.28 |  | 3633 |  | 3633 |  | Image |  | Peak List |  |
|  | | | | | | | | | | | | | | | | | | | | | | | | | | | | | | | | |
|  | **P01857 - Ig gamma-1 chain C region OS=Homo sapiens GN=IGHG1 PE=1 SV=1 - [IGHG1\_HUMAN]** | | | | | | | | | | | | | | | | | | | | | | | | | | | | | | |  |
|  | | | | | | | | | | | | | | | | | | | | | | | | | | | | | | | | |
|  | High |  | GPSVFPLAPSSK |  | CID |  |  |  |  |  | 2.11 |  | 1.00 |  | 1 |  | SEQUEST (4) |  | 2 |  | 593.82764 |  | 1.08 |  | 1702 |  | 1702 |  | Image |  | Peak List |  |
|  | | | | | | | | | | | | | | | | | | | | | | | | | | | | | | | | |
|  | **P10768 - S-formylglutathione hydrolase OS=Homo sapiens GN=ESD PE=1 SV=2 - [ESTD\_HUMAN]** | | | | | | | | | | | | | | | | | | | | | | | | | | | | | | |  |
|  | | | | | | | | | | | | | | | | | | | | | | | | | | | | | | | | |
|  | High |  | LQEGYDHSYYFIATFITDHIR |  | CID |  |  |  |  |  | 2.34 |  | 1.00 |  | 1 |  | SEQUEST (4) |  | 4 |  | 648.06818 |  | 3.84 |  | 3171 |  | 3171 |  | Image |  | Peak List |  |
|  | | | | | | | | | | | | | | | | | | | | | | | | | | | | | | | | |
|  | **Q02252 - Methylmalonate-semialdehyde dehydrogenase [acylating], mitochondrial OS=Homo sapiens GN=ALDH6A1 PE=1 SV=2 - [MMSA\_HUMAN]** | | | | | | | | | | | | | | | | | | | | | | | | | | | | | | |  |
|  | | | | | | | | | | | | | | | | | | | | | | | | | | | | | | | | |
|  | High |  | WIDIHNPATNEVIGR |  | CID |  |  |  |  |  | 2.70 |  | 1.00 |  | 1 |  | SEQUEST (4) |  | 3 |  | 578.96991 |  | 1.67 |  | 1836 |  | 1836 |  | Image |  | Peak List |  |
|  | | | | | | | | | | | | | | | | | | | | | | | | | | | | | | | | |
|  | **Q13415 - Origin recognition complex subunit 1 OS=Homo sapiens GN=ORC1 PE=1 SV=2 - [ORC1\_HUMAN]** | | | | | | | | | | | | | | | | | | | | | | | | | | | | | | |  |
|  | | | | | | | | | | | | | | | | | | | | | | | | | | | | | | | | |
|  | High |  | MEGLPYPTmSETmAVCSHLGSCR |  | CID |  | M9(Oxidation); M13(Oxidation) |  |  |  | 1.51 |  | 1.00 |  | 1 |  | SEQUEST (4) |  | 3 |  | 844.70184 |  | 8.40 |  | 6287 |  | 6287 |  | Image |  | Peak List |  |
|  | | | | | | | | | | | | | | | | | | | | | | | | | | | | | | | | |
|  | **Q4G0N4-2 - Isoform 2 of NAD kinase domain-containing protein 1 OS=Homo sapiens GN=NADKD1 - [NAKD1\_HUMAN]** | | | | | | | | | | | | | | | | | | | | | | | | | | | | | | |  |
|  | | | | | | | | | | | | | | | | | | | | | | | | | | | | | | | | |
|  | High |  | YAELSEEDLK |  | CID |  |  |  |  |  | 1.63 |  | 1.00 |  | 1 |  | SEQUEST (4) |  | 2 |  | 598.78766 |  | -0.11 |  | 1217 |  | 1217 |  | Image |  | Peak List |  |
|  | | | | | | | | | | | | | | | | | | | | | | | | | | | | | | | | |
|  | **Q53XA7 - Fumarylacetoacetase OS=Homo sapiens GN=DKFZp686F13224 PE=2 SV=1 - [Q53XA7\_HUMAN]** | | | | | | | | | | | | | | | | | | | | | | | | | | | | | | |  |
|  | | | | | | | | | | | | | | | | | | | | | | | | | | | | | | | | |
|  | High |  | SFGTTVSPWVVPMDALMPFAVPNPK |  | CID |  |  |  |  |  | 2.75 |  | 1.00 |  | 1 |  | SEQUEST (4) |  | 3 |  | 896.79688 |  | 7.25 |  | 4180 |  | 4180 |  | Image |  | Peak List |  |
|  | | | | | | | | | | | | | | | | | | | | | | | | | | | | | | | | |
|  | **Q8IZF2-2 - Isoform 2 of Probable G-protein coupled receptor 116 OS=Homo sapiens GN=GPR116 - [GP116\_HUMAN]** | | | | | | | | | | | | | | | | | | | | | | | | | | | | | | |  |
|  | | | | | | | | | | | | | | | | | | | | | | | | | | | | | | | | |
|  | High |  | KIDVmPIQILANEEmK |  | CID |  | M5(Oxidation); M15(Oxidation) |  |  |  | 2.47 |  | 1.00 |  | 1 |  | SEQUEST (4) |  | 3 |  | 635.33618 |  | 3.74 |  | 1894 |  | 1894 |  | Image |  | Peak List |  |
|  | | | | | | | | | | | | | | | | | | | | | | | | | | | | | | | | |
|  | **Q93099 - Homogentisate 1,2-dioxygenase OS=Homo sapiens GN=HGD PE=1 SV=2 - [HGD\_HUMAN]** | | | | | | | | | | | | | | | | | | | | | | | | | | | | | | |  |
|  | | | | | | | | | | | | | | | | | | | | | | | | | | | | | | | | |
|  | High |  | IADGTMAFMFESSLSLAVTKWGLKASR |  | CID |  |  |  |  |  | 3.08 |  | 1.00 |  | 1 |  | SEQUEST (4) |  | 4 |  | 730.13293 |  | 5.04 |  | 3577 |  | 3577 |  | Image |  | Peak List |  |
|  | | | | | | | | | | | | | | | | | | | | | | | | | | | | | | | | |
|  | **Q9HBB2 - Cytoplasmic aconitate hydratase OS=Homo sapiens GN=IRP1 PE=2 SV=1 - [Q9HBB2\_HUMAN]** | | | | | | | | | | | | | | | | | | | | | | | | | | | | | | |  |
|  | | | | | | | | | | | | | | | | | | | | | | | | | | | | | | | | |
|  | High |  | QAPQTIHLPSGEILDVFDAAER |  | CID |  |  |  |  |  | 2.29 |  | 1.00 |  | 1 |  | SEQUEST (4) |  | 3 |  | 803.08331 |  | 4.18 |  | 2860 |  | 2860 |  | Image |  | Peak List |  |
|  | | | | | | | | | | | | | | | | | | | | | | | | | | | | | | | | |
|  | **A6NED0 - Glutathione transferase zeta 1 (Maleylacetoacetate isomerase), isoform CRA\_b OS=Homo sapiens GN=GSTZ1 PE=3 SV=1 - [A6NED0\_HUMAN]** | | | | | | | | | | | | | | | | | | | | | | | | | | | | | | |  |
|  | | | | | | | | | | | | | | | | | | | | | | | | | | | | | | | | |
|  | High |  | DFQALNPMK |  | CID |  |  |  |  |  | 2.23 |  | 1.00 |  | 1 |  | SEQUEST (4) |  | 2 |  | 532.26715 |  | 2.70 |  | 1884 |  | 1884 |  | Image |  | Peak List |  |
|  | | | | | | | | | | | | | | | | | | | | | | | | | | | | | | | | |
|  | **F5GY93 - Cob(I)yrinic acid a,c-diamide adenosyltransferase, mitochondrial (Fragment) OS=Homo sapiens GN=MMAB PE=4 SV=1 - [F5GY93\_HUMAN]** | | | | | | | | | | | | | | | | | | | | | | | | | | | | | | |  |
|  | | | | | | | | | | | | | | | | | | | | | | | | | | | | | | | | |
|  | High |  | DDQVFEAVGTTDELSSAIGFALELVTEK |  | CID |  |  |  |  |  | 2.21 |  | 1.00 |  | 1 |  | SEQUEST (4) |  | 3 |  | 995.50208 |  | 9.80 |  | 5834 |  | 5834 |  | Image |  | Peak List |  |
|  | | | | | | | | | | | | | | | | | | | | | | | | | | | | | | | | |
|  | **F5H2A5 - Aconitate hydratase, mitochondrial OS=Homo sapiens GN=ACO2 PE=4 SV=1 - [F5H2A5\_HUMAN]** | | | | | | | | | | | | | | | | | | | | | | | | | | | | | | |  |
|  | | | | | | | | | | | | | | | | | | | | | | | | | | | | | | | | |
|  | High |  | IVYGHLDDPASQEIER |  | CID |  |  |  |  |  | 2.59 |  | 1.00 |  | 1 |  | SEQUEST (4) |  | 3 |  | 614.64020 |  | 1.71 |  | 1300 |  | 1300 |  | Image |  | Peak List |  |
|  | | | | | | | | | | | | | | | | | | | | | | | | | | | | | | | | |
|  | **F5H6U4 - Microsomal triglyceride transfer protein large subunit OS=Homo sapiens GN=MTTP PE=4 SV=1 - [F5H6U4\_HUMAN]** | | | | | | | | | | | | | | | | | | | | | | | | | | | | | | |  |
|  | | | | | | | | | | | | | | | | | | | | | | | | | | | | | | | | |
|  | High |  | EVLPQLVDAVTSAQTSDSLEAILDFLDFK |  | CID |  |  |  |  |  | 2.53 |  | 1.00 |  | 1 |  | SEQUEST (4) |  | 3 |  | 1055.55591 |  | 8.88 |  | 5390 |  | 5390 |  | Image |  | Peak List |  |
|  | | | | | | | | | | | | | | | | | | | | | | | | | | | | | | | | |
|  | **Q9UIJ7-2 - Isoform 2 of GTP:AMP phosphotransferase, mitochondrial OS=Homo sapiens GN=AK3 - [KAD3\_HUMAN]** | | | | | | | | | | | | | | | | | | | | | | | | | | | | | | |  |
|  | | | | | | | | | | | | | | | | | | | | | | | | | | | | | | | | |
|  | High |  | AYEDQTKPVLEYYQK |  | CID |  |  |  |  |  | 1.79 |  | 1.00 |  | 1 |  | SEQUEST (4) |  | 3 |  | 625.64484 |  | 1.52 |  | 1204 |  | 1204 |  | Image |  | Peak List |  |
|  | | | | | | | | | | | | | | | | | | | | | | | | | | | | | | | | |

  
Top
  

### Search Summary

Workflow created with Discoverer version: 1.4.0.288 (DBVersion:79)
  
  
================================================================================
  
  
Search name: Methanol\_50\_1\_MC3
  
Search description: -
  
Search date: 06/25/2014 16:30:19
  
  
================================================================================
  
  
The pipeline tree:
  
------------------
  
  
    |-(0) Spectrum Files
  
        |-(1) Spectrum Selector
  
            |-(2) Mascot
  
                |-(3) Percolator
  
            |-(4) SEQUEST
  
                |-(3) Percolator
  
  
================================================================================
  
  
Search name: Methanol\_50\_1\_MC3
  
Search description: -
  
Search date: 06/25/2014 16:30:19
  
  
================================================================================
  
  
The pipeline tree:
  
------------------
  
  
    |-(0) Spectrum Files
  
        |-(1) Spectrum Selector
  
            |-(2) Mascot
  
                |-(3) Percolator
  
            |-(4) SEQUEST
  
                |-(3) Percolator
  
  
------------------------------------------------------------------------------
  
Processing node 0: Spectrum Files
  
------------------------------------------------------------------------------
  
  
Input Data:
  
-----------------------------
  
File Name(s): E:\Jos\Methanol\_50\_1.raw
  
  
------------------------------------------------------------------------------
  
Processing node 1: Spectrum Selector
  
------------------------------------------------------------------------------
  
  
1. General Settings:
  
-----------------------------
  
Precursor Selection: Use MS1 Precursor
  
Use New Precursor Reevaluation: True
  
  
2. Spectrum Properties Filter:
  
-----------------------------
  
Lower RT Limit: 0
  
Upper RT Limit: 0
  
First Scan: 0
  
Last Scan: 0
  
Lowest Charge State: 0
  
Highest Charge State: 0
  
Min. Precursor Mass: 350 Da
  
Max. Precursor Mass: 5000 Da
  
Total Intensity Threshold: 0
  
Minimum Peak Count: 1
  
  
3. Scan Event Filters:
  
-----------------------------
  
MS Order: Is MS2
  
Activation Type: Is CID
  
Min. Collision Energy: 0
  
Max. Collision Energy: 1000
  
Scan Type: Is Full
  
Ionization Source: Is Nanospray
  
  
4. Peak Filters:
  
-----------------------------
  
S/N Threshold (FT-only): 1.5
  
  
5. Replacements for Unrecognized Properties:
  
-----------------------------
  
Unrecognized Charge Replacements: Automatic
  
Unrecognized Mass Analyzer Replacements: ITMS
  
Unrecognized MS Order Replacements: MS2
  
Unrecognized Activation Type Replacements: CID
  
Unrecognized Polarity Replacements: +
  
  
6. Just for Testing:
  
-----------------------------
  
Precursor Clipping Range Before: 2.5 Da
  
Precursor Clipping Range After: 5.5 Da
  
  
------------------------------------------------------------------------------
  
Processing node 2: Mascot
  
------------------------------------------------------------------------------
  
  
1. Input Data:
  
-----------------------------
  
Protein Database: SwissProt
  
Enzyme Name: Trypsin
  
Maximum Missed Cleavage Sites: 3
  
Instrument: Default
  
Taxonomy: . . . . . . . . . . . . . . . . Homo sapiens (human)
  
  
1.1 Peptide Scoring Options:
  
-----------------------------
  
Peptide Cut Off Score: 10
  
Peptide Without Protein Cut Off Score: 5
  
  
1.2 Protein Scoring Options:
  
-----------------------------
  
Use MudPIT Scoring: Automatic
  
Protein Relevance Threshold: 20
  
Protein Relevance Factor: 1
  
  
2. Tolerances:
  
-----------------------------
  
Precursor Mass Tolerance: 10 ppm
  
Fragment Mass Tolerance: 0.8 Da
  
Use Average Precursor Mass: False
  
  
4. Dynamic Modifications:
  
-----------------------------
  
1. Dynamic Modification: Oxidation (M)
  
  
------------------------------------------------------------------------------
  
Processing node 3: Percolator
  
------------------------------------------------------------------------------
  
  
1. Input Data:
  
-----------------------------
  
Maximum Delta Cn: 0.05
  
  
2. Decoy Database Search:
  
-----------------------------
  
Target FDR (Strict): 0.01
  
Target FDR (Relaxed): 0.05
  
Validation based on: q-Value
  
  
------------------------------------------------------------------------------
  
Processing node 4: SEQUEST
  
------------------------------------------------------------------------------
  
  
1. Input Data:
  
-----------------------------
  
Protein Database: HUMAN\_swiss\_Jos.fasta
  
Enzyme Name: Trypsin (Full)
  
Maximum Missed Cleavage Sites: 3
  
  
1.1 Peptide Scoring Options:
  
-----------------------------
  
Maximum Peptides Considered: 500
  
Maximum Peptides Output: 10
  
Calculate Probability Scores: False
  
Absolute XCorr Threshold: 0.4
  
Fragment Ion Cutoff Percentage: 0.1
  
Peptide Without Protein XCorr Threshold: 1.5
  
  
1.2 Protein Scoring Options:
  
-----------------------------
  
Maximum Protein References Per Peptide: 100
  
Protein Relevance Threshold: 1.5
  
Peptide Relevance Factor: 0.4
  
  
2. Tolerances:
  
-----------------------------
  
Precursor Mass Tolerance: 10 ppm
  
Fragment Mass Tolerance: 0.8 Da
  
Use Average Precursor Mass: False
  
Use Average Fragment Masses: False
  
  
3. Ion Series:
  
-----------------------------
  
Use Neutral Loss a Ions: True
  
Use Neutral Loss b Ions: True
  
Use Neutral Loss y Ions: True
  
Weight of a Ions: 0
  
Weight of b Ions: 1
  
Weight of c Ions: 0
  
Weight of x Ions: 0
  
Weight of y Ions: 1
  
Weight of z Ions: 0
  
  
4. Dynamic Modifications:
  
-----------------------------
  
Max. Modifications Per Peptide: 4
  
1. Dynamic Modification: Oxidation / +15.995 Da (M)
  
  
================================================================================
  
  
Processing details:
  
  
06/25/2014 04:39 PM (4):SEQUEST: Total search time was 4 min 48 s.
  
06/25/2014 04:39 PM (3):Percolator: Performing percolator for SEQUEST (4) took 51.1 s.
  
06/25/2014 04:39 PM (4):SEQUEST: Search completed
  
06/25/2014 04:39 PM (4):SEQUEST: 9275 protein(s) + 8582 decoy proteins scored and inserted into result file in 5.4 s.
  
06/25/2014 04:39 PM (4):SEQUEST: 9275 protein(s) scored
  
06/25/2014 04:39 PM (4):SEQUEST: Search result finalization started.
  
06/25/2014 04:39 PM (3):Percolator: Start reading Percolator results
  
06/25/2014 04:39 PM (3):Percolator: Processing took 6.599 cpu seconds or 6 seconds wall time
  
06/25/2014 04:39 PM (3):Percolator: PSMId score q-value posterior\_error\_prob peptide proteinIds
  
06/25/2014 04:39 PM (3):Percolator: Calibrating statistics - calculating Posterior error probabilities (PEPs)
  
06/25/2014 04:39 PM (3):Percolator: New pi\_0 estimate on merged list gives 814 peptides over q=0.0100
  
06/25/2014 04:39 PM (3):Percolator: Calibrating statistics - calculating q values
  
06/25/2014 04:39 PM (3):Percolator: Selecting pi\_0=0.7857
  
06/25/2014 04:39 PM (3):Percolator: Tossing out "redundant" PSMs keeping only the best scoring PSM for each unique peptide.
  
06/25/2014 04:39 PM (3):Percolator: Merging results from 3 datasets
  
06/25/2014 04:39 PM (3):Percolator: Found 1251 target PSMs scoring over 1.0000% FDR level on testset
  
06/25/2014 04:39 PM (3):Percolator: 0.6816 -0.0017 1.3836 0.0237 -0.0057 -0.0000 -5.4227 0.0849 59.3691 -0.2638 -0.0040 0.0000 -1.8741 0.3103 2.1191 2.1976 3.0221 -1.0429 0.0000 -0.7810 0.0243 -0.0184 0.0003 -0.5349 0.2676 0.2667 0.3651 -1.5756 -0.0004 -12.8013 0.0015 -12.2201 0.0013 2.8629
  
06/25/2014 04:39 PM (3):Percolator: 0.62 -0.5373 0.3852 0.8748 -0.1541 -0.0343 -0.0819 0.4726 0.5359 -0.7573 -0.0331 0.0000 -0.9081 0.1541 0.7079 0.4290 0.3616 -1.1405 0.0000 -0.4814 0.3149 -0.3349 0.0052 -1.4811 0.7485 0.5841 0.9870 -0.3223 -0.1067 -1.2771 0.2578 -1.1536 0.2449 -4.0960
  
06/25/2014 04:39 PM (3):Percolator: XCorr SpScore Delta Cn From Second PSM Binomial Score Isolation Interference [%] MH+ [Da] Delta Mass [Da] Delta Mass [ppm] Absolute Delta Mass [Da] Absolute Delta Mass [ppm] Peptide Length Is z=1 Is z=2 Is z=3 Is z=4 Is z=5 Is z>5 # Missed Cleavages Log Peptides Matched Log Total Intensity Fraction Matched Intensity [%] Fragment Coverage Series A, B, C [%] Fragment Coverage Series X, Y, Z [%] Log Matched Fragment Series Intensities A, B, C Log Matched Fragment Series Intensities X, Y, Z Longest Sequence Series A, B, C Longest Sequence Series X, Y, Z IQR Fragment Delta Mass [Da] IQR Fragment Delta Mass [ppm] Mean Fragment Delta Mass [Da] Mean Fragment Delta Mass [ppm] Mean Absolute Fragment Delta Mass [Da] Mean Absolute Fragment Delta Mass [ppm] m0
  
06/25/2014 04:39 PM (3):Percolator: # first line contains normalized weights, second line the raw weights
  
06/25/2014 04:39 PM (3):Percolator: Obtained weights (only showing weights of first cross validation set)
  
06/25/2014 04:39 PM (3):Percolator: Iteration 10 : After the iteration step, 1332 target PSMs with q<0.01 were estimated by cross validation
  
06/25/2014 04:39 PM (3):Percolator: Iteration 9 : After the iteration step, 1332 target PSMs with q<0.01 were estimated by cross validation
  
06/25/2014 04:39 PM (3):Percolator: Iteration 8 : After the iteration step, 1331 target PSMs with q<0.01 were estimated by cross validation
  
06/25/2014 04:39 PM (3):Percolator: Iteration 7 : After the iteration step, 1329 target PSMs with q<0.01 were estimated by cross validation
  
06/25/2014 04:39 PM (3):Percolator: Iteration 6 : After the iteration step, 1328 target PSMs with q<0.01 were estimated by cross validation
  
06/25/2014 04:39 PM (3):Percolator: Iteration 5 : After the iteration step, 1321 target PSMs with q<0.01 were estimated by cross validation
  
06/25/2014 04:39 PM (3):Percolator: Iteration 4 : After the iteration step, 1320 target PSMs with q<0.01 were estimated by cross validation
  
06/25/2014 04:39 PM (3):Percolator: Iteration 3 : After the iteration step, 1314 target PSMs with q<0.01 were estimated by cross validation
  
06/25/2014 04:39 PM (3):Percolator: Iteration 2 : After the iteration step, 1307 target PSMs with q<0.01 were estimated by cross validation
  
06/25/2014 04:39 PM (3):Percolator: Iteration 1 : After the iteration step, 1248 target PSMs with q<0.01 were estimated by cross validation
  
06/25/2014 04:39 PM (3):Percolator: ---Training with Cpos selected by cross validation, Cneg selected by cross validation, fdr=0.01
  
06/25/2014 04:39 PM (3):Percolator: Reading in data and feature calculation took 7.456 cpu seconds or 8 seconds wall time
  
06/25/2014 04:39 PM (3):Percolator: Estimating 793 over q=0.01 in initial direction
  
06/25/2014 04:39 PM (3):Percolator: Selected feature number 4 as initial search direction, could separate 530 positives in that direction
  
06/25/2014 04:39 PM (3):Percolator: Selected feature number 27 as initial search direction, could separate 496 positives in that direction
  
06/25/2014 04:39 PM (3):Percolator: Selected feature number 27 as initial search direction, could separate 533 positives in that direction
  
06/25/2014 04:39 PM (3):Percolator: selecting cneg by cross validation
  
06/25/2014 04:39 PM (3):Percolator: selecting cpos by cross validation
  
06/25/2014 04:39 PM (3):Percolator: Train/test set contains 4683 positives and 5092 negatives, size ratio=0.919678 and pi0=1
  
06/25/2014 04:39 PM (3):Percolator: 31e77142-29e9-402c-9ec2-8468a9513af0 e39a792e-622c-452d-b49b-59809cad79d0 Delta Cn From Second PSM Binomial Score b8754504-e95e-476b-b9a4-454d4bb53aeb 1d91a87b-953a-4887-9f22-f75a497a3538 Delta Mass [Da] Delta Mass [ppm] Absolute Delta Mass [Da] Absolute Delta Mass [ppm] Peptide Length Is z=1 Is z=2 Is z=3 Is z=4 Is z=5 Is z>5 041eb6d5-e486-44a0-9bc1-19e25811c686 Log Peptides Matched Log Total Intensity Fraction Matched Intensity [%] Fragment Coverage Series A, B, C [%] Fragment Coverage Series X, Y, Z [%] Log Matched Fragment Series Intensities A, B, C Log Matched Fragment Series Intensities X, Y, Z Longest Sequence Series A, B, C Longest Sequence Series X, Y, Z IQR Fragment Delta Mass [Da] IQR Fragment Delta Mass [ppm] Mean Fragment Delta Mass [Da] Mean Fragment Delta Mass [ppm] Mean Absolute Fragment Delta Mass [Da] Mean Absolute Fragment Delta Mass [ppm]
  
06/25/2014 04:39 PM (3):Percolator: Features:
  
06/25/2014 04:39 PM (3):Percolator: enzyme=Trypsin
  
06/25/2014 04:39 PM (3):Percolator: Hyperparameters fdr=0.01, Cpos=0, Cneg=0, maxNiter=10
  
06/25/2014 04:39 PM (3):Percolator: Started Wed Jun 25 16:39:20 2014
  
06/25/2014 04:39 PM (3):Percolator: C:\Program Files\Thermo\Discoverer 1.4\Tools\Percolator\percolator.exe -X C:\ProgramData\Thermo\Discoverer 1.4\Scratch\d4b2269b-294a-4e70-97a5-1d0e797ca79f\output.xml -Z C:\ProgramData\Thermo\Discoverer 1.4\Scratch\d4b2269b-294a-4e70-97a5-1d0e797ca79f\input.xml
  
06/25/2014 04:39 PM (3):Percolator: Issued command:
  
06/25/2014 04:39 PM (3):Percolator: Department of Genome Sciences at the University of Washington.
  
06/25/2014 04:39 PM (3):Percolator: Written by Lukas K+�ll (lukall@u.washington.edu) in the
  
06/25/2014 04:39 PM (3):Percolator: Copyright (c) 2006-9 University of Washington. All rights reserved.
  
06/25/2014 04:39 PM (3):Percolator: Percolator version 2.04, Build Date Feb 1 2012 03:35:34
  
06/25/2014 04:39 PM (3):Percolator: Starting Percolator
  
06/25/2014 04:39 PM (3):Percolator: The input file contains 4683 peptides, 5092 decoy peptides and 33 features.
  
06/25/2014 04:39 PM (3):Percolator: Creating input file for SEQUEST (4) took 34.9 s.
  
06/25/2014 04:38 PM (3):Percolator: Start calculating features for peptides of SEQUEST (4)
  
06/25/2014 04:38 PM (2):Mascot: Total search time was 2 min 11 s.
  
06/25/2014 04:38 PM (3):Percolator: Performing percolator for Mascot (2) took 42.3 s.
  
06/25/2014 04:38 PM (2):Mascot: Search completed
  
06/25/2014 04:38 PM (2):Mascot: 241 protein(s) + 31 decoy proteins scored and inserted into result file in 0.7 s.
  
06/25/2014 04:38 PM (2):Mascot: 241 protein(s) scored
  
06/25/2014 04:38 PM (2):Mascot: Search result finalization started.
  
06/25/2014 04:38 PM (3):Percolator: Start reading Percolator results
  
06/25/2014 04:38 PM (3):Percolator: Processing took 5.289 cpu seconds or 5 seconds wall time
  
06/25/2014 04:38 PM (3):Percolator: PSMId score q-value posterior\_error\_prob peptide proteinIds
  
06/25/2014 04:38 PM (3):Percolator: Calibrating statistics - calculating Posterior error probabilities (PEPs)
  
06/25/2014 04:38 PM (3):Percolator: New pi\_0 estimate on merged list gives 792 peptides over q=0.0100
  
06/25/2014 04:38 PM (3):Percolator: Calibrating statistics - calculating q values
  
06/25/2014 04:38 PM (3):Percolator: Selecting pi\_0=0.7280
  
06/25/2014 04:38 PM (3):Percolator: Tossing out "redundant" PSMs keeping only the best scoring PSM for each unique peptide.
  
06/25/2014 04:38 PM (3):Percolator: Merging results from 3 datasets
  
06/25/2014 04:38 PM (3):Percolator: Found 1212 target PSMs scoring over 1.0000% FDR level on testset
  
06/25/2014 04:38 PM (3):Percolator: 0.0420 0.2030 0.0140 0.0088 -0.0000 -4.6403 0.1226 58.3481 -0.1902 -0.0201 0.0000 -0.6229 0.4849 -0.0971 0.2118 1.0360 -0.8625 0.0000 -0.3547 0.0133 -0.0355 -0.0041 -0.0989 0.2942 0.3895 0.4477 0.5544 -0.0009 -4.7730 -0.0012 -6.8503 -0.0009 -0.2258
  
06/25/2014 04:38 PM (3):Percolator: 0.65 0.0595 0.5534 0.2391 -0.0154 -0.0756 0.6750 0.5855 -0.5535 -0.1951 0.0000 -0.2884 0.2422 -0.0349 0.0441 0.1285 -0.9566 0.0000 -0.2878 0.2048 -0.7336 -0.0948 -0.5833 0.8932 0.9383 1.3704 0.1297 -0.2904 -0.5432 -0.2028 -0.7514 -0.1932 -2.6670
  
06/25/2014 04:38 PM (3):Percolator: IonScore Delta Cn From Second PSM Binomial Score Isolation Interference [%] MH+ [Da] Delta Mass [Da] Delta Mass [ppm] Absolute Delta Mass [Da] Absolute Delta Mass [ppm] Peptide Length Is z=1 Is z=2 Is z=3 Is z=4 Is z=5 Is z>5 # Missed Cleavages Log Peptides Matched Log Total Intensity Fraction Matched Intensity [%] Fragment Coverage Series A, B, C [%] Fragment Coverage Series X, Y, Z [%] Log Matched Fragment Series Intensities A, B, C Log Matched Fragment Series Intensities X, Y, Z Longest Sequence Series A, B, C Longest Sequence Series X, Y, Z IQR Fragment Delta Mass [Da] IQR Fragment Delta Mass [ppm] Mean Fragment Delta Mass [Da] Mean Fragment Delta Mass [ppm] Mean Absolute Fragment Delta Mass [Da] Mean Absolute Fragment Delta Mass [ppm] m0
  
06/25/2014 04:38 PM (3):Percolator: # first line contains normalized weights, second line the raw weights
  
06/25/2014 04:38 PM (3):Percolator: Obtained weights (only showing weights of first cross validation set)
  
06/25/2014 04:38 PM (3):Percolator: Iteration 10 : After the iteration step, 1288 target PSMs with q<0.01 were estimated by cross validation
  
06/25/2014 04:38 PM (3):Percolator: Iteration 9 : After the iteration step, 1287 target PSMs with q<0.01 were estimated by cross validation
  
06/25/2014 04:38 PM (3):Percolator: Iteration 8 : After the iteration step, 1288 target PSMs with q<0.01 were estimated by cross validation
  
06/25/2014 04:38 PM (3):Percolator: Iteration 7 : After the iteration step, 1286 target PSMs with q<0.01 were estimated by cross validation
  
06/25/2014 04:38 PM (3):Percolator: Iteration 6 : After the iteration step, 1285 target PSMs with q<0.01 were estimated by cross validation
  
06/25/2014 04:38 PM (3):Percolator: Iteration 5 : After the iteration step, 1282 target PSMs with q<0.01 were estimated by cross validation
  
06/25/2014 04:38 PM (3):Percolator: Iteration 4 : After the iteration step, 1279 target PSMs with q<0.01 were estimated by cross validation
  
06/25/2014 04:38 PM (3):Percolator: Iteration 3 : After the iteration step, 1276 target PSMs with q<0.01 were estimated by cross validation
  
06/25/2014 04:38 PM (3):Percolator: Iteration 2 : After the iteration step, 1267 target PSMs with q<0.01 were estimated by cross validation
  
06/25/2014 04:38 PM (3):Percolator: Iteration 1 : After the iteration step, 1221 target PSMs with q<0.01 were estimated by cross validation
  
06/25/2014 04:38 PM (3):Percolator: ---Training with Cpos selected by cross validation, Cneg selected by cross validation, fdr=0.01
  
06/25/2014 04:38 PM (3):Percolator: Reading in data and feature calculation took 5.584 cpu seconds or 5 seconds wall time
  
06/25/2014 04:38 PM (3):Percolator: Estimating 824 over q=0.01 in initial direction
  
06/25/2014 04:38 PM (3):Percolator: Selected feature number 1 as initial search direction, could separate 605 positives in that direction
  
06/25/2014 04:38 PM (3):Percolator: Selected feature number 26 as initial search direction, could separate 471 positives in that direction
  
06/25/2014 04:38 PM (3):Percolator: Selected feature number 26 as initial search direction, could separate 478 positives in that direction
  
06/25/2014 04:38 PM (3):Percolator: selecting cneg by cross validation
  
06/25/2014 04:38 PM (3):Percolator: selecting cpos by cross validation
  
06/25/2014 04:38 PM (3):Percolator: Train/test set contains 3763 positives and 3719 negatives, size ratio=1.01183 and pi0=1
  
06/25/2014 04:38 PM (3):Percolator: e6e22773-e9a6-4a26-9694-1ca77a797099 Delta Cn From Second PSM Binomial Score b8754504-e95e-476b-b9a4-454d4bb53aeb 1d91a87b-953a-4887-9f22-f75a497a3538 Delta Mass [Da] Delta Mass [ppm] Absolute Delta Mass [Da] Absolute Delta Mass [ppm] Peptide Length Is z=1 Is z=2 Is z=3 Is z=4 Is z=5 Is z>5 041eb6d5-e486-44a0-9bc1-19e25811c686 Log Peptides Matched Log Total Intensity Fraction Matched Intensity [%] Fragment Coverage Series A, B, C [%] Fragment Coverage Series X, Y, Z [%] Log Matched Fragment Series Intensities A, B, C Log Matched Fragment Series Intensities X, Y, Z Longest Sequence Series A, B, C Longest Sequence Series X, Y, Z IQR Fragment Delta Mass [Da] IQR Fragment Delta Mass [ppm] Mean Fragment Delta Mass [Da] Mean Fragment Delta Mass [ppm] Mean Absolute Fragment Delta Mass [Da] Mean Absolute Fragment Delta Mass [ppm]
  
06/25/2014 04:38 PM (3):Percolator: Features:
  
06/25/2014 04:38 PM (3):Percolator: enzyme=Trypsin
  
06/25/2014 04:38 PM (3):Percolator: Hyperparameters fdr=0.01, Cpos=0, Cneg=0, maxNiter=10
  
06/25/2014 04:38 PM (3):Percolator: Started Wed Jun 25 16:38:31 2014
  
06/25/2014 04:38 PM (3):Percolator: C:\Program Files\Thermo\Discoverer 1.4\Tools\Percolator\percolator.exe -X C:\ProgramData\Thermo\Discoverer 1.4\Scratch\15597b69-c8e6-45d5-bf45-1f6f93d800a5\output.xml -Z C:\ProgramData\Thermo\Discoverer 1.4\Scratch\15597b69-c8e6-45d5-bf45-1f6f93d800a5\input.xml
  
06/25/2014 04:38 PM (3):Percolator: Issued command:
  
06/25/2014 04:38 PM (3):Percolator: Department of Genome Sciences at the University of Washington.
  
06/25/2014 04:38 PM (3):Percolator: Written by Lukas K+�ll (lukall@u.washington.edu) in the
  
06/25/2014 04:38 PM (3):Percolator: Copyright (c) 2006-9 University of Washington. All rights reserved.
  
06/25/2014 04:38 PM (3):Percolator: Percolator version 2.04, Build Date Feb 1 2012 03:35:34
  
06/25/2014 04:38 PM (3):Percolator: Starting Percolator
  
06/25/2014 04:38 PM (3):Percolator: The input file contains 3763 peptides, 3719 decoy peptides and 32 features.
  
06/25/2014 04:38 PM (3):Percolator: Creating input file for Mascot (2) took 29.7 s.
  
06/25/2014 04:38 PM (3):Percolator: Start calculating features for peptides of Mascot (2)
  
06/25/2014 04:38 PM (2):Mascot: Used mascot server http://fenn.bham.ac.uk/mascot/ with Mascot version 2.4.1
  
06/25/2014 04:38 PM (2):Mascot: Sending 3299 peptide hits (13811 peptides) to result file
  
06/25/2014 04:38 PM (2):Mascot: Sending 3041 decoy peptide hits (12280 peptides) to result file
  
06/25/2014 04:37 PM (2):Mascot: Reading decoy results
  
06/25/2014 04:37 PM (2):Mascot: Start translating results
  
06/25/2014 04:37 PM (2):Mascot: Start mapping modifications
  
06/25/2014 04:37 PM (2):Mascot: Received 256 proteins from Mascot server
  
06/25/2014 04:37 PM (2):Mascot: Start mapping 256 proteins
  
06/25/2014 04:37 PM (2):Mascot: Start parsing results
  
06/25/2014 04:37 PM (2):Mascot: Received Mascot result file (filename=../data/20140625/F004276.dat)
  
06/25/2014 04:37 PM (2):Mascot: Mascot Server completed
  
06/25/2014 04:36 PM (2):Mascot: Mascot result on server (filename=../data/20140625/F004276.dat)
  
06/25/2014 04:35 PM (2):Mascot: Start searching 4698 spectra
  
06/25/2014 04:35 PM (4):SEQUEST: Sending 698 decoy peptide hits (3530 peptides) to result file
  
06/25/2014 04:35 PM (4):SEQUEST: Starting SEQUEST decoy search
  
06/25/2014 04:35 PM (4):SEQUEST: Sending 698 peptide hits (3542 peptides) to result file
  
06/25/2014 04:35 PM (4):SEQUEST: Starting SEQUEST (search spectra 4000 - 4698)
  
06/25/2014 04:35 PM (4):SEQUEST: Sending 1000 decoy peptide hits (5257 peptides) to result file
  
06/25/2014 04:35 PM (4):SEQUEST: Starting SEQUEST decoy search
  
06/25/2014 04:35 PM (4):SEQUEST: Sending 1000 peptide hits (5273 peptides) to result file
  
06/25/2014 04:35 PM (4):SEQUEST: Starting SEQUEST (search spectra 3000 - 4000)
  
06/25/2014 04:35 PM (4):SEQUEST: Sending 1000 decoy peptide hits (7624 peptides) to result file
  
06/25/2014 04:35 PM (4):SEQUEST: Starting SEQUEST decoy search
  
06/25/2014 04:35 PM (4):SEQUEST: Sending 1000 peptide hits (7592 peptides) to result file
  
06/25/2014 04:34 PM (4):SEQUEST: Starting SEQUEST (search spectra 2000 - 3000)
  
06/25/2014 04:34 PM (4):SEQUEST: Sending 1000 decoy peptide hits (9583 peptides) to result file
  
06/25/2014 04:34 PM (4):SEQUEST: Starting SEQUEST decoy search
  
06/25/2014 04:34 PM (4):SEQUEST: Sending 1000 peptide hits (9562 peptides) to result file
  
06/25/2014 04:34 PM (4):SEQUEST: Starting SEQUEST (search spectra 1000 - 2000)
  
06/25/2014 04:34 PM (4):SEQUEST: Sending 1000 decoy peptide hits (9654 peptides) to result file
  
06/25/2014 04:33 PM (4):SEQUEST: Starting SEQUEST decoy search
  
06/25/2014 04:33 PM (4):SEQUEST: Sending 1000 peptide hits (9661 peptides) to result file
  
06/25/2014 04:33 PM (4):SEQUEST: Starting SEQUEST (search spectra 0 - 1000)
  
06/25/2014 04:33 PM (4):SEQUEST: FASTA indexing completed in 1 min 30 s.
  
06/25/2014 04:32 PM (4):SEQUEST: Building new decoy FASTA index. This may take up to several hours...
  
06/25/2014 04:32 PM (4):SEQUEST: Looking for existing decoy FASTA index.
  
06/25/2014 04:32 PM (4):SEQUEST: FASTA indexing completed in 1 min 28 s.
  
06/25/2014 04:30 PM (4):SEQUEST: Building new target FASTA index. This may take up to several hours...
  
06/25/2014 04:30 PM (4):SEQUEST: Looking for existing target FASTA index.
  
06/25/2014 04:30 PM (2):Mascot: Use mascot server http://fenn.bham.ac.uk/mascot/ with Mascot version 2.4.1
  
06/25/2014 04:30 PM (1):Spectrum Selector: Reading from File 1 of 1:E:\Jos\Methanol\_50\_1.raw (7928 spectra total)
  
  
  
Top
