# Supplementary material for: Top-Down and Bottom-Up Identification of Proteins by Liquid Extraction Surface Analysis Mass Spectrometry of Healthy and Diseased Human Liver Tissue
Source: J Am Soc Mass Spectrom. 2014 Sep 3;25(11):1953–61. doi: 10.1007/s13361-014-0967-z (PMC4197381; doi:10.1007/s13361-014-0967-z)
Supplement: Supplementary file 7 — (ZIP 2096 kb) [file 13361_2014_967_MOESM7_ESM.zip › index.html]

Annotated spectra


## Annotated spectra of Methanol\_50\_2\_MC3.msf

go to Peptides  
go to Search Summary  

### Peptides

|  |  |  |  |  |  |  |  |  |  |  |  |  |  |  |  |  |  |  |  |  |  |  |  |  |  |  |  |  |  |  |  |  |
| --- | --- | --- | --- | --- | --- | --- | --- | --- | --- | --- | --- | --- | --- | --- | --- | --- | --- | --- | --- | --- | --- | --- | --- | --- | --- | --- | --- | --- | --- | --- | --- | --- |
|  | | | | | | | | | | | | | | | | | | | | | | | | | | | | | | | | |
| Confidence Sequence Activation Type Modifications IonScore XCorr ΔScore Rank Search Engine Charge Precursor m/z [Da] ΔM [ppm] First Scan Last Scan Annotated Spectrum Peak List  | | | | | | | | | | | | | | | | | | | | | | | | | | | | | | | | |
|  | | | | | | | | | | | | | | | | | | | | | | | | | | | | | | | | |
|  | **O00757 - Fructose-1,6-bisphosphatase isozyme 2 OS=Homo sapiens GN=FBP2 PE=1 SV=2 - [F16P2\_HUMAN]** | | | | | | | | | | | | | | | | | | | | | | | | | | | | | | |  |
|  | | | | | | | | | | | | | | | | | | | | | | | | | | | | | | | | |
|  | High |  | IYSLNEGYAK |  | CID |  |  |  | 32.76 |  |  |  | 1.00 |  | 1 |  | Mascot (2) |  | 2 |  | 579.29584 |  | 0.57 |  | 1087 |  | 1087 |  | Image |  | Peak List |  |
|  | | | | | | | | | | | | | | | | | | | | | | | | | | | | | | | | |
|  | High |  | IYSLNEGYAK |  | CID |  |  |  |  |  | 2.57 |  | 1.00 |  | 1 |  | SEQUEST (4) |  | 2 |  | 579.29584 |  | 0.57 |  | 1087 |  | 1087 |  | Image |  | Peak List |  |
|  | | | | | | | | | | | | | | | | | | | | | | | | | | | | | | | | |
|  | **P00738 - Haptoglobin OS=Homo sapiens GN=HP PE=1 SV=1 - [HPT\_HUMAN]** | | | | | | | | | | | | | | | | | | | | | | | | | | | | | | |  |
|  | | | | | | | | | | | | | | | | | | | | | | | | | | | | | | | | |
|  | High |  | TEGDGVYTLNNEK |  | CID |  |  |  | 39.82 |  |  |  | 1.00 |  | 1 |  | Mascot (2) |  | 2 |  | 720.33759 |  | 2.06 |  | 964 |  | 964 |  | Image |  | Peak List |  |
|  | | | | | | | | | | | | | | | | | | | | | | | | | | | | | | | | |
|  | High |  | TEGDGVYTLNNEK |  | CID |  |  |  |  |  | 2.75 |  | 1.00 |  | 1 |  | SEQUEST (4) |  | 2 |  | 720.33759 |  | 2.06 |  | 964 |  | 964 |  | Image |  | Peak List |  |
|  | | | | | | | | | | | | | | | | | | | | | | | | | | | | | | | | |
|  | **P04040 - Catalase OS=Homo sapiens GN=CAT PE=1 SV=3 - [CATA\_HUMAN]** | | | | | | | | | | | | | | | | | | | | | | | | | | | | | | |  |
|  | | | | | | | | | | | | | | | | | | | | | | | | | | | | | | | | |
|  | High |  | NAIHTFVQSGSHLAAR |  | CID |  |  |  | 20.38 |  |  |  | 1.00 |  | 1 |  | Mascot (2) |  | 3 |  | 570.30121 |  | 0.72 |  | 962 |  | 962 |  | Image |  | Peak List |  |
|  | | | | | | | | | | | | | | | | | | | | | | | | | | | | | | | | |
|  | High |  | NAIHTFVQSGSHLAAR |  | CID |  |  |  |  |  | 2.04 |  | 1.00 |  | 1 |  | SEQUEST (4) |  | 3 |  | 570.30121 |  | 0.72 |  | 962 |  | 962 |  | Image |  | Peak List |  |
|  | | | | | | | | | | | | | | | | | | | | | | | | | | | | | | | | |
|  | **P04075 - Fructose-bisphosphate aldolase A OS=Homo sapiens GN=ALDOA PE=1 SV=2 - [ALDOA\_HUMAN]** | | | | | | | | | | | | | | | | | | | | | | | | | | | | | | |  |
|  | | | | | | | | | | | | | | | | | | | | | | | | | | | | | | | | |
|  | High |  | GILAADESTGSIAK |  | CID |  |  |  | 42.33 |  |  |  | 1.00 |  | 1 |  | Mascot (2) |  | 2 |  | 666.85455 |  | 0.91 |  | 1217 |  | 1217 |  | Image |  | Peak List |  |
|  | | | | | | | | | | | | | | | | | | | | | | | | | | | | | | | | |
|  | High |  | GILAADESTGSIAK |  | CID |  |  |  |  |  | 3.18 |  | 1.00 |  | 1 |  | SEQUEST (4) |  | 2 |  | 666.85455 |  | 0.91 |  | 1217 |  | 1217 |  | Image |  | Peak List |  |
|  | | | | | | | | | | | | | | | | | | | | | | | | | | | | | | | | |
|  | **P11310 - Medium-chain specific acyl-CoA dehydrogenase, mitochondrial OS=Homo sapiens GN=ACADM PE=1 SV=1 - [ACADM\_HUMAN]** | | | | | | | | | | | | | | | | | | | | | | | | | | | | | | |  |
|  | | | | | | | | | | | | | | | | | | | | | | | | | | | | | | | | |
|  | High |  | AFAGDIANQLATDAVQILGGNGFNTEYPVEK |  | CID |  |  |  | 35.48 |  |  |  | 1.00 |  | 1 |  | Mascot (2) |  | 3 |  | 1075.21106 |  | 8.65 |  | 4476 |  | 4476 |  | Image |  | Peak List |  |
|  | | | | | | | | | | | | | | | | | | | | | | | | | | | | | | | | |
|  | High |  | AFAGDIANQLATDAVQILGGNGFNTEYPVEK |  | CID |  |  |  |  |  | 4.20 |  | 1.00 |  | 1 |  | SEQUEST (4) |  | 3 |  | 1075.20959 |  | 7.28 |  | 4455 |  | 4455 |  | Image |  | Peak List |  |
|  | | | | | | | | | | | | | | | | | | | | | | | | | | | | | | | | |
|  | **P11586 - C-1-tetrahydrofolate synthase, cytoplasmic OS=Homo sapiens GN=MTHFD1 PE=1 SV=3 - [C1TC\_HUMAN]** | | | | | | | | | | | | | | | | | | | | | | | | | | | | | | |  |
|  | | | | | | | | | | | | | | | | | | | | | | | | | | | | | | | | |
|  | High |  | TAQFDISVASEIMAVLALTTSLEDMR |  | CID |  |  |  | 31.14 |  |  |  | 1.00 |  | 1 |  | Mascot (2) |  | 3 |  | 938.14764 |  | 6.23 |  | 5589 |  | 5589 |  | Image |  | Peak List |  |
|  | | | | | | | | | | | | | | | | | | | | | | | | | | | | | | | | |
|  | High |  | TAQFDISVASEIMAVLALTTSLEDMR |  | CID |  |  |  |  |  | 3.11 |  | 1.00 |  | 1 |  | SEQUEST (4) |  | 3 |  | 938.14764 |  | 6.23 |  | 5589 |  | 5589 |  | Image |  | Peak List |  |
|  | | | | | | | | | | | | | | | | | | | | | | | | | | | | | | | | |
|  | **P21695 - Glycerol-3-phosphate dehydrogenase [NAD(+)], cytoplasmic OS=Homo sapiens GN=GPD1 PE=1 SV=4 - [GPDA\_HUMAN]** | | | | | | | | | | | | | | | | | | | | | | | | | | | | | | |  |
|  | | | | | | | | | | | | | | | | | | | | | | | | | | | | | | | | |
|  | High |  | LPPNVVAVPDVVQAAEDADILIFVVPHQFIGK |  | CID |  |  |  | 29.08 |  |  |  | 1.00 |  | 1 |  | Mascot (2) |  | 3 |  | 1137.63989 |  | 9.59 |  | 4599 |  | 4599 |  | Image |  | Peak List |  |
|  | | | | | | | | | | | | | | | | | | | | | | | | | | | | | | | | |
|  | High |  | LPPNVVAVPDVVQAAEDADILIFVVPHQFIGK |  | CID |  |  |  |  |  | 4.50 |  | 1.00 |  | 1 |  | SEQUEST (4) |  | 3 |  | 1137.63989 |  | 9.59 |  | 4599 |  | 4599 |  | Image |  | Peak List |  |
|  | | | | | | | | | | | | | | | | | | | | | | | | | | | | | | | | |
|  | **P21980 - Protein-glutamine gamma-glutamyltransferase 2 OS=Homo sapiens GN=TGM2 PE=1 SV=2 - [TGM2\_HUMAN]** | | | | | | | | | | | | | | | | | | | | | | | | | | | | | | |  |
|  | | | | | | | | | | | | | | | | | | | | | | | | | | | | | | | | |
|  | High |  | NIPWNFGQFEDGILDICLILLDVNPK |  | CID |  |  |  | 24.49 |  |  |  | 1.00 |  | 1 |  | Mascot (2) |  | 3 |  | 996.19354 |  | 9.25 |  | 6162 |  | 6162 |  | Image |  | Peak List |  |
|  | | | | | | | | | | | | | | | | | | | | | | | | | | | | | | | | |
|  | High |  | NIPWNFGQFEDGILDICLILLDVNPK |  | CID |  |  |  |  |  | 2.72 |  | 1.00 |  | 1 |  | SEQUEST (4) |  | 3 |  | 996.19354 |  | 9.25 |  | 6162 |  | 6162 |  | Image |  | Peak List |  |
|  | | | | | | | | | | | | | | | | | | | | | | | | | | | | | | | | |
|  | **P23378 - Glycine dehydrogenase [decarboxylating], mitochondrial OS=Homo sapiens GN=GLDC PE=1 SV=2 - [GCSP\_HUMAN]** | | | | | | | | | | | | | | | | | | | | | | | | | | | | | | |  |
|  | | | | | | | | | | | | | | | | | | | | | | | | | | | | | | | | |
|  | High |  | EFANIHPFVPLDQAQGYQQLFR |  | CID |  |  |  | 34.99 |  |  |  | 1.00 |  | 1 |  | Mascot (2) |  | 3 |  | 873.44623 |  | 3.44 |  | 2871 |  | 2871 |  | Image |  | Peak List |  |
|  | | | | | | | | | | | | | | | | | | | | | | | | | | | | | | | | |
|  | High |  | EFANIHPFVPLDQAQGYQQLFR |  | CID |  |  |  |  |  | 5.09 |  | 1.00 |  | 1 |  | SEQUEST (4) |  | 3 |  | 873.44623 |  | 3.44 |  | 2871 |  | 2871 |  | Image |  | Peak List |  |
|  | | | | | | | | | | | | | | | | | | | | | | | | | | | | | | | | |
|  | **P30039 - Phenazine biosynthesis-like domain-containing protein OS=Homo sapiens GN=PBLD PE=1 SV=2 - [PBLD\_HUMAN]** | | | | | | | | | | | | | | | | | | | | | | | | | | | | | | |  |
|  | | | | | | | | | | | | | | | | | | | | | | | | | | | | | | | | |
|  | High |  | LPIFIADAFTAR |  | CID |  |  |  | 60.72 |  |  |  | 1.00 |  | 1 |  | Mascot (2) |  | 2 |  | 667.87927 |  | 3.37 |  | 3095 |  | 3095 |  | Image |  | Peak List |  |
|  | | | | | | | | | | | | | | | | | | | | | | | | | | | | | | | | |
|  | High |  | LPIFIADAFTAR |  | CID |  |  |  |  |  | 3.43 |  | 1.00 |  | 1 |  | SEQUEST (4) |  | 2 |  | 667.87927 |  | 3.37 |  | 3095 |  | 3095 |  | Image |  | Peak List |  |
|  | | | | | | | | | | | | | | | | | | | | | | | | | | | | | | | | |
|  | **P34896 - Serine hydroxymethyltransferase, cytosolic OS=Homo sapiens GN=SHMT1 PE=1 SV=1 - [GLYC\_HUMAN]** | | | | | | | | | | | | | | | | | | | | | | | | | | | | | | |  |
|  | | | | | | | | | | | | | | | | | | | | | | | | | | | | | | | | |
|  | High |  | ISATSIFFESMPYK |  | CID |  |  |  | 22.92 |  |  |  | 1.00 |  | 1 |  | Mascot (2) |  | 2 |  | 810.90479 |  | 2.73 |  | 2926 |  | 2926 |  | Image |  | Peak List |  |
|  | | | | | | | | | | | | | | | | | | | | | | | | | | | | | | | | |
|  | High |  | ISATSIFFESMPYK |  | CID |  |  |  |  |  | 2.66 |  | 1.00 |  | 1 |  | SEQUEST (4) |  | 2 |  | 810.90479 |  | 2.73 |  | 2926 |  | 2926 |  | Image |  | Peak List |  |
|  | | | | | | | | | | | | | | | | | | | | | | | | | | | | | | | | |
|  | **P34913 - Bifunctional epoxide hydrolase 2 OS=Homo sapiens GN=EPHX2 PE=1 SV=2 - [HYES\_HUMAN]** | | | | | | | | | | | | | | | | | | | | | | | | | | | | | | |  |
|  | | | | | | | | | | | | | | | | | | | | | | | | | | | | | | | | |
|  | High |  | DLGMVTILVQDTDTALKELEK |  | CID |  |  |  | 24.92 |  |  |  | 1.00 |  | 1 |  | Mascot (2) |  | 3 |  | 778.08783 |  | 7.38 |  | 4445 |  | 4445 |  | Image |  | Peak List |  |
|  | | | | | | | | | | | | | | | | | | | | | | | | | | | | | | | | |
|  | High |  | DLGMVTILVQDTDTALKELEK |  | CID |  |  |  |  |  | 2.15 |  | 1.00 |  | 1 |  | SEQUEST (4) |  | 3 |  | 778.08783 |  | 7.38 |  | 4445 |  | 4445 |  | Image |  | Peak List |  |
|  | | | | | | | | | | | | | | | | | | | | | | | | | | | | | | | | |
|  | **P42126 - Enoyl-CoA delta isomerase 1, mitochondrial OS=Homo sapiens GN=ECI1 PE=1 SV=1 - [ECI1\_HUMAN]** | | | | | | | | | | | | | | | | | | | | | | | | | | | | | | |  |
|  | | | | | | | | | | | | | | | | | | | | | | | | | | | | | | | | |
|  | High |  | VLVEPDAGAGVAVMK |  | CID |  |  |  | 47.58 |  |  |  | 1.00 |  | 1 |  | Mascot (2) |  | 2 |  | 728.39801 |  | 0.73 |  | 1657 |  | 1657 |  | Image |  | Peak List |  |
|  | | | | | | | | | | | | | | | | | | | | | | | | | | | | | | | | |
|  | High |  | VLVEPDAGAGVAVMK |  | CID |  |  |  |  |  | 2.89 |  | 1.00 |  | 1 |  | SEQUEST (4) |  | 2 |  | 728.39801 |  | 0.73 |  | 1657 |  | 1657 |  | Image |  | Peak List |  |
|  | | | | | | | | | | | | | | | | | | | | | | | | | | | | | | | | |
|  | **P51858 - Hepatoma-derived growth factor OS=Homo sapiens GN=HDGF PE=1 SV=1 - [HDGF\_HUMAN]** | | | | | | | | | | | | | | | | | | | | | | | | | | | | | | |  |
|  | | | | | | | | | | | | | | | | | | | | | | | | | | | | | | | | |
|  | High |  | GPPQEEEEEEDEEEEATKEDAEAPGIR |  | CID |  |  |  | 39.23 |  |  |  | 1.00 |  | 1 |  | Mascot (2) |  | 3 |  | 1014.76727 |  | 1.74 |  | 1161 |  | 1161 |  | Image |  | Peak List |  |
|  | | | | | | | | | | | | | | | | | | | | | | | | | | | | | | | | |
|  | High |  | GPPQEEEEEEDEEEEATKEDAEAPGIR |  | CID |  |  |  |  |  | 4.53 |  | 1.00 |  | 1 |  | SEQUEST (4) |  | 3 |  | 1014.76727 |  | 1.74 |  | 1161 |  | 1161 |  | Image |  | Peak List |  |
|  | | | | | | | | | | | | | | | | | | | | | | | | | | | | | | | | |
|  | **P55263 - Adenosine kinase OS=Homo sapiens GN=ADK PE=1 SV=2 - [ADK\_HUMAN]** | | | | | | | | | | | | | | | | | | | | | | | | | | | | | | |  |
|  | | | | | | | | | | | | | | | | | | | | | | | | | | | | | | | | |
|  | High |  | ENILFGMGNPLLDISAVVDKDFLDK |  | CID |  |  |  | 28.27 |  |  |  | 1.00 |  | 1 |  | Mascot (2) |  | 3 |  | 921.82294 |  | 9.68 |  | 4507 |  | 4507 |  | Image |  | Peak List |  |
|  | | | | | | | | | | | | | | | | | | | | | | | | | | | | | | | | |
|  | High |  | ENILFGMGNPLLDISAVVDKDFLDK |  | CID |  |  |  |  |  | 2.84 |  | 1.00 |  | 1 |  | SEQUEST (4) |  | 3 |  | 921.82294 |  | 9.68 |  | 4507 |  | 4507 |  | Image |  | Peak List |  |
|  | | | | | | | | | | | | | | | | | | | | | | | | | | | | | | | | |
|  | **P68104 - Elongation factor 1-alpha 1 OS=Homo sapiens GN=EEF1A1 PE=1 SV=1 - [EF1A1\_HUMAN]** | | | | | | | | | | | | | | | | | | | | | | | | | | | | | | |  |
|  | | | | | | | | | | | | | | | | | | | | | | | | | | | | | | | | |
|  | High |  | NMITGTSQADCAVLIVAAGVGEFEAGISK |  | CID |  |  |  | 46.79 |  |  |  | 1.00 |  | 1 |  | Mascot (2) |  | 3 |  | 951.48553 |  | 8.77 |  | 4360 |  | 4360 |  | Image |  | Peak List |  |
|  | | | | | | | | | | | | | | | | | | | | | | | | | | | | | | | | |
|  | High |  | NMITGTSQADCAVLIVAAGVGEFEAGISK |  | CID |  |  |  |  |  | 5.37 |  | 1.00 |  | 1 |  | SEQUEST (4) |  | 3 |  | 951.48553 |  | 8.77 |  | 4360 |  | 4360 |  | Image |  | Peak List |  |
|  | | | | | | | | | | | | | | | | | | | | | | | | | | | | | | | | |
|  | **Q08257 - Quinone oxidoreductase OS=Homo sapiens GN=CRYZ PE=1 SV=1 - [QOR\_HUMAN]** | | | | | | | | | | | | | | | | | | | | | | | | | | | | | | |  |
|  | | | | | | | | | | | | | | | | | | | | | | | | | | | | | | | | |
|  | High |  | GIDIIIEMLANVNLSK |  | CID |  |  |  | 45.60 |  |  |  | 1.00 |  | 1 |  | Mascot (2) |  | 2 |  | 871.99677 |  | 8.07 |  | 4488 |  | 4488 |  | Image |  | Peak List |  |
|  | | | | | | | | | | | | | | | | | | | | | | | | | | | | | | | | |
|  | High |  | GIDIIIEMLANVNLSK |  | CID |  |  |  |  |  | 3.83 |  | 1.00 |  | 1 |  | SEQUEST (4) |  | 3 |  | 581.66577 |  | 6.07 |  | 4520 |  | 4520 |  | Image |  | Peak List |  |
|  | | | | | | | | | | | | | | | | | | | | | | | | | | | | | | | | |
|  | **Q14353 - Guanidinoacetate N-methyltransferase OS=Homo sapiens GN=GAMT PE=1 SV=1 - [GAMT\_HUMAN]** | | | | | | | | | | | | | | | | | | | | | | | | | | | | | | |  |
|  | | | | | | | | | | | | | | | | | | | | | | | | | | | | | | | | |
|  | High |  | YSDITIMFEETQVPALLEAGFR |  | CID |  |  |  | 45.16 |  |  |  | 1.00 |  | 1 |  | Mascot (2) |  | 3 |  | 844.09625 |  | 8.17 |  | 4135 |  | 4135 |  | Image |  | Peak List |  |
|  | | | | | | | | | | | | | | | | | | | | | | | | | | | | | | | | |
|  | High |  | YSDITIMFEETQVPALLEAGFR |  | CID |  |  |  |  |  | 4.63 |  | 1.00 |  | 1 |  | SEQUEST (4) |  | 3 |  | 844.09625 |  | 8.17 |  | 4135 |  | 4135 |  | Image |  | Peak List |  |
|  | | | | | | | | | | | | | | | | | | | | | | | | | | | | | | | | |
|  | **Q14697 - Neutral alpha-glucosidase AB OS=Homo sapiens GN=GANAB PE=1 SV=3 - [GANAB\_HUMAN]** | | | | | | | | | | | | | | | | | | | | | | | | | | | | | | |  |
|  | | | | | | | | | | | | | | | | | | | | | | | | | | | | | | | | |
|  | High |  | DLGIFWLNAAETWVDISSNTAGK |  | CID |  |  |  | 22.96 |  |  |  | 1.00 |  | 1 |  | Mascot (2) |  | 3 |  | 836.75946 |  | 9.24 |  | 4983 |  | 4983 |  | Image |  | Peak List |  |
|  | | | | | | | | | | | | | | | | | | | | | | | | | | | | | | | | |
|  | High |  | DLGIFWLNAAETWVDISSNTAGK |  | CID |  |  |  |  |  | 4.63 |  | 1.00 |  | 1 |  | SEQUEST (4) |  | 3 |  | 836.75946 |  | 9.24 |  | 4983 |  | 4983 |  | Image |  | Peak List |  |
|  | | | | | | | | | | | | | | | | | | | | | | | | | | | | | | | | |
|  | **Q8IZF2 - Probable G-protein coupled receptor 116 OS=Homo sapiens GN=GPR116 PE=1 SV=3 - [GP116\_HUMAN]** | | | | | | | | | | | | | | | | | | | | | | | | | | | | | | |  |
|  | | | | | | | | | | | | | | | | | | | | | | | | | | | | | | | | |
|  | High |  | KIDVmPIQILANEEmK |  | CID |  | M5(Oxidation); M15(Oxidation) |  | 20.14 |  |  |  | 1.00 |  | 1 |  | Mascot (2) |  | 3 |  | 635.33594 |  | 3.36 |  | 1879 |  | 1879 |  | Image |  | Peak List |  |
|  | | | | | | | | | | | | | | | | | | | | | | | | | | | | | | | | |
|  | High |  | KIDVmPIQILANEEmK |  | CID |  | M5(Oxidation); M15(Oxidation) |  |  |  | 2.87 |  | 1.00 |  | 1 |  | SEQUEST (4) |  | 3 |  | 635.33594 |  | 3.36 |  | 1879 |  | 1879 |  | Image |  | Peak List |  |
|  | | | | | | | | | | | | | | | | | | | | | | | | | | | | | | | | |
|  | **Q96IU4 - Alpha/beta hydrolase domain-containing protein 14B OS=Homo sapiens GN=ABHD14B PE=1 SV=1 - [ABHEB\_HUMAN]** | | | | | | | | | | | | | | | | | | | | | | | | | | | | | | |  |
|  | | | | | | | | | | | | | | | | | | | | | | | | | | | | | | | | |
|  | High |  | TPALIVYGDQDPMGQTSFEHLK |  | CID |  |  |  | 45.64 |  |  |  | 1.00 |  | 1 |  | Mascot (2) |  | 3 |  | 816.40320 |  | 1.55 |  | 2149 |  | 2149 |  | Image |  | Peak List |  |
|  | | | | | | | | | | | | | | | | | | | | | | | | | | | | | | | | |
|  | High |  | TPALIVYGDQDPMGQTSFEHLK |  | CID |  |  |  |  |  | 4.45 |  | 1.00 |  | 1 |  | SEQUEST (4) |  | 3 |  | 816.40320 |  | 1.55 |  | 2149 |  | 2149 |  | Image |  | Peak List |  |
|  | | | | | | | | | | | | | | | | | | | | | | | | | | | | | | | | |
|  | **Q9BWD1 - Acetyl-CoA acetyltransferase, cytosolic OS=Homo sapiens GN=ACAT2 PE=1 SV=2 - [THIC\_HUMAN]** | | | | | | | | | | | | | | | | | | | | | | | | | | | | | | |  |
|  | | | | | | | | | | | | | | | | | | | | | | | | | | | | | | | | |
|  | High |  | AGWSLEDVDIFEINEAFAAVSAAIVK |  | CID |  |  |  | 48.31 |  |  |  | 1.00 |  | 1 |  | Mascot (2) |  | 3 |  | 922.47894 |  | 6.83 |  | 5106 |  | 5106 |  | Image |  | Peak List |  |
|  | | | | | | | | | | | | | | | | | | | | | | | | | | | | | | | | |
|  | High |  | AGWSLEDVDIFEINEAFAAVSAAIVK |  | CID |  |  |  |  |  | 4.99 |  | 1.00 |  | 1 |  | SEQUEST (4) |  | 3 |  | 922.48175 |  | 9.87 |  | 5113 |  | 5113 |  | Image |  | Peak List |  |
|  | | | | | | | | | | | | | | | | | | | | | | | | | | | | | | | | |
|  | **Q9BX68 - Histidine triad nucleotide-binding protein 2, mitochondrial OS=Homo sapiens GN=HINT2 PE=1 SV=1 - [HINT2\_HUMAN]** | | | | | | | | | | | | | | | | | | | | | | | | | | | | | | |  |
|  | | | | | | | | | | | | | | | | | | | | | | | | | | | | | | | | |
|  | High |  | ISQAEEEDQQLLGHLLLVAK |  | CID |  |  |  | 42.66 |  |  |  | 1.00 |  | 1 |  | Mascot (2) |  | 3 |  | 745.40857 |  | 3.74 |  | 2797 |  | 2797 |  | Image |  | Peak List |  |
|  | | | | | | | | | | | | | | | | | | | | | | | | | | | | | | | | |
|  | High |  | ISQAEEEDQQLLGHLLLVAK |  | CID |  |  |  |  |  | 3.42 |  | 1.00 |  | 1 |  | SEQUEST (4) |  | 3 |  | 745.40857 |  | 3.74 |  | 2797 |  | 2797 |  | Image |  | Peak List |  |
|  | | | | | | | | | | | | | | | | | | | | | | | | | | | | | | | | |
|  | **Q9H2A2 - Aldehyde dehydrogenase family 8 member A1 OS=Homo sapiens GN=ALDH8A1 PE=1 SV=1 - [AL8A1\_HUMAN]** | | | | | | | | | | | | | | | | | | | | | | | | | | | | | | |  |
|  | | | | | | | | | | | | | | | | | | | | | | | | | | | | | | | | |
|  | High |  | VLNQVADLLEQSLEEFAQAESK |  | CID |  |  |  | 41.94 |  |  |  | 1.00 |  | 1 |  | Mascot (2) |  | 3 |  | 821.09247 |  | 6.93 |  | 4533 |  | 4533 |  | Image |  | Peak List |  |
|  | | | | | | | | | | | | | | | | | | | | | | | | | | | | | | | | |
|  | High |  | VLNQVADLLEQSLEEFAQAESK |  | CID |  |  |  |  |  | 4.63 |  | 1.00 |  | 1 |  | SEQUEST (4) |  | 3 |  | 821.09247 |  | 6.93 |  | 4533 |  | 4533 |  | Image |  | Peak List |  |
|  | | | | | | | | | | | | | | | | | | | | | | | | | | | | | | | | |
|  | **B4E152 - Pyridoxine-5'-phosphate oxidase OS=Homo sapiens GN=PNPO PE=2 SV=1 - [B4E152\_HUMAN]** | | | | | | | | | | | | | | | | | | | | | | | | | | | | | | |  |
|  | | | | | | | | | | | | | | | | | | | | | | | | | | | | | | | | |
|  | High |  | EAFEETHLTSLDPVK |  | CID |  |  |  |  |  | 2.37 |  | 1.00 |  | 1 |  | SEQUEST (4) |  | 3 |  | 572.62158 |  | 0.85 |  | 1609 |  | 1609 |  | Image |  | Peak List |  |
|  | | | | | | | | | | | | | | | | | | | | | | | | | | | | | | | | |
|  | **B8ZZZ0 - 3-hydroxyisobutyryl-CoA hydrolase, mitochondrial (Fragment) OS=Homo sapiens GN=HIBCH PE=4 SV=1 - [B8ZZZ0\_HUMAN]** | | | | | | | | | | | | | | | | | | | | | | | | | | | | | | |  |
|  | | | | | | | | | | | | | | | | | | | | | | | | | | | | | | | | |
|  | High |  | KWEQDPETFLIIIK |  | CID |  |  |  |  |  | 2.70 |  | 1.00 |  | 1 |  | SEQUEST (4) |  | 3 |  | 587.32855 |  | 4.61 |  | 2646 |  | 2646 |  | Image |  | Peak List |  |
|  | | | | | | | | | | | | | | | | | | | | | | | | | | | | | | | | |
|  | **F2Z393 - Transaldolase OS=Homo sapiens GN=TALDO1 PE=3 SV=1 - [F2Z393\_HUMAN]** | | | | | | | | | | | | | | | | | | | | | | | | | | | | | | |  |
|  | | | | | | | | | | | | | | | | | | | | | | | | | | | | | | | | |
|  | High |  | SYEPLEDPGVK |  | CID |  |  |  |  |  | 2.14 |  | 1.00 |  | 1 |  | SEQUEST (4) |  | 2 |  | 617.30426 |  | 1.16 |  | 1137 |  | 1137 |  | Image |  | Peak List |  |
|  | | | | | | | | | | | | | | | | | | | | | | | | | | | | | | | | |
|  | **F6USW4 - F-actin-capping protein subunit beta (Fragment) OS=Homo sapiens GN=CAPZB PE=4 SV=1 - [F6USW4\_HUMAN]** | | | | | | | | | | | | | | | | | | | | | | | | | | | | | | |  |
|  | | | | | | | | | | | | | | | | | | | | | | | | | | | | | | | | |
|  | High |  | NLSDLIDLVPSLCEDLLSSVDQPLK |  | CID |  |  |  |  |  | 1.76 |  | 1.00 |  | 1 |  | SEQUEST (4) |  | 3 |  | 909.48517 |  | 8.80 |  | 6247 |  | 6247 |  | Image |  | Peak List |  |
|  | | | | | | | | | | | | | | | | | | | | | | | | | | | | | | | | |
|  | **F8WEC7 - Zinc finger protein 473 OS=Homo sapiens GN=ZNF473 PE=4 SV=1 - [F8WEC7\_HUMAN]** | | | | | | | | | | | | | | | | | | | | | | | | | | | | | | |  |
|  | | | | | | | | | | | | | | | | | | | | | | | | | | | | | | | | |
|  | High |  | KHLIQHQKTHAAK |  | CID |  |  |  |  |  | 1.15 |  | 1.00 |  | 1 |  | SEQUEST (4) |  | 3 |  | 513.97009 |  | 5.86 |  | 879 |  | 879 |  | Image |  | Peak List |  |
|  | | | | | | | | | | | | | | | | | | | | | | | | | | | | | | | | |
|  | **H3BQ84 - Acyl-coenzyme A synthetase ACSM2B, mitochondrial (Fragment) OS=Homo sapiens GN=ACSM2B PE=4 SV=1 - [H3BQ84\_HUMAN]** | | | | | | | | | | | | | | | | | | | | | | | | | | | | | | |  |
|  | | | | | | | | | | | | | | | | | | | | | | | | | | | | | | | | |
|  | High |  | FNFASDVLDHWADMEK |  | CID |  |  |  |  |  | 1.64 |  | 1.00 |  | 1 |  | SEQUEST (4) |  | 3 |  | 642.29144 |  | 3.25 |  | 3039 |  | 3039 |  | Image |  | Peak List |  |
|  | | | | | | | | | | | | | | | | | | | | | | | | | | | | | | | | |
|  | **O00748 - Cocaine esterase OS=Homo sapiens GN=CES2 PE=1 SV=1 - [EST2\_HUMAN]** | | | | | | | | | | | | | | | | | | | | | | | | | | | | | | |  |
|  | | | | | | | | | | | | | | | | | | | | | | | | | | | | | | | | |
|  | High |  | ADHGDELPFVFR |  | CID |  |  |  |  |  | 1.75 |  | 1.00 |  | 1 |  | SEQUEST (4) |  | 3 |  | 468.23022 |  | 0.76 |  | 2072 |  | 2072 |  | Image |  | Peak List |  |
|  | | | | | | | | | | | | | | | | | | | | | | | | | | | | | | | | |
|  | **O95718-3 - Isoform 3 of Steroid hormone receptor ERR2 OS=Homo sapiens GN=ESRRB - [ERR2\_HUMAN]** | | | | | | | | | | | | | | | | | | | | | | | | | | | | | | |  |
|  | | | | | | | | | | | | | | | | | | | | | | | | | | | | | | | | |
|  | High |  | LYAMPPPGmPEGDIK |  | CID |  | M9(Oxidation) |  |  |  | 1.65 |  | 1.00 |  | 1 |  | SEQUEST (4) |  | 2 |  | 816.39270 |  | -1.69 |  | 1633 |  | 1633 |  | Image |  | Peak List |  |
|  | | | | | | | | | | | | | | | | | | | | | | | | | | | | | | | | |
|  | **P06744 - Glucose-6-phosphate isomerase OS=Homo sapiens GN=GPI PE=1 SV=4 - [G6PI\_HUMAN]** | | | | | | | | | | | | | | | | | | | | | | | | | | | | | | |  |
|  | | | | | | | | | | | | | | | | | | | | | | | | | | | | | | | | |
|  | High |  | IFVQGIIWDINSFDQWGVELGK |  | CID |  |  |  |  |  | 2.57 |  | 1.00 |  | 1 |  | SEQUEST (4) |  | 3 |  | 855.45239 |  | 9.40 |  | 4401 |  | 4401 |  | Image |  | Peak List |  |
|  | | | | | | | | | | | | | | | | | | | | | | | | | | | | | | | | |
|  | **P10768 - S-formylglutathione hydrolase OS=Homo sapiens GN=ESD PE=1 SV=2 - [ESTD\_HUMAN]** | | | | | | | | | | | | | | | | | | | | | | | | | | | | | | |  |
|  | | | | | | | | | | | | | | | | | | | | | | | | | | | | | | | | |
|  | High |  | LQEGYDHSYYFIATFITDHIR |  | CID |  |  |  |  |  | 2.30 |  | 1.00 |  | 1 |  | SEQUEST (4) |  | 4 |  | 648.06757 |  | 2.90 |  | 3160 |  | 3160 |  | Image |  | Peak List |  |
|  | | | | | | | | | | | | | | | | | | | | | | | | | | | | | | | | |
|  | **P46939 - Utrophin OS=Homo sapiens GN=UTRN PE=1 SV=2 - [UTRO\_HUMAN]** | | | | | | | | | | | | | | | | | | | | | | | | | | | | | | |  |
|  | | | | | | | | | | | | | | | | | | | | | | | | | | | | | | | | |
|  | High |  | mGQLASGIRSSLLPTDYLVEINK |  | CID |  | M1(Oxidation) |  |  |  | 1.62 |  | 1.00 |  | 1 |  | SEQUEST (4) |  | 3 |  | 841.11816 |  | 2.69 |  | 5451 |  | 5451 |  | Image |  | Peak List |  |
|  | | | | | | | | | | | | | | | | | | | | | | | | | | | | | | | | |
|  | **P49419-3 - Isoform 3 of Alpha-aminoadipic semialdehyde dehydrogenase OS=Homo sapiens GN=ALDH7A1 - [AL7A1\_HUMAN]** | | | | | | | | | | | | | | | | | | | | | | | | | | | | | | |  |
|  | | | | | | | | | | | | | | | | | | | | | | | | | | | | | | | | |
|  | High |  | FKNEEEVFAWNNEVK |  | CID |  |  |  |  |  | 2.28 |  | 1.00 |  | 1 |  | SEQUEST (4) |  | 3 |  | 628.30536 |  | 2.35 |  | 1656 |  | 1656 |  | Image |  | Peak List |  |
|  | | | | | | | | | | | | | | | | | | | | | | | | | | | | | | | | |
|  | **H3BQ75 - Protein ST20-MTHFS OS=Homo sapiens GN=ST20-MTHFS PE=4 SV=1 - [H3BQ75\_HUMAN]** | | | | | | | | | | | | | | | | | | | | | | | | | | | | | | |  |
|  | | | | | | | | | | | | | | | | | | | | | | | | | | | | | | | | |
|  | High |  | EEALSTGGLDLIFMPGLGFDK |  | CID |  |  |  |  |  | 2.97 |  | 1.00 |  | 1 |  | SEQUEST (4) |  | 2 |  | 1105.55188 |  | -3.89 |  | 4041 |  | 4041 |  | Image |  | Peak List |  |
|  | | | | | | | | | | | | | | | | | | | | | | | | | | | | | | | | |
|  | **Q02252 - Methylmalonate-semialdehyde dehydrogenase [acylating], mitochondrial OS=Homo sapiens GN=ALDH6A1 PE=1 SV=2 - [MMSA\_HUMAN]** | | | | | | | | | | | | | | | | | | | | | | | | | | | | | | |  |
|  | | | | | | | | | | | | | | | | | | | | | | | | | | | | | | | | |
|  | High |  | WIDIHNPATNEVIGR |  | CID |  |  |  |  |  | 2.53 |  | 1.00 |  | 1 |  | SEQUEST (4) |  | 3 |  | 578.97034 |  | 2.41 |  | 1783 |  | 1783 |  | Image |  | Peak List |  |
|  | | | | | | | | | | | | | | | | | | | | | | | | | | | | | | | | |
|  | **Q4G0N4-2 - Isoform 2 of NAD kinase domain-containing protein 1 OS=Homo sapiens GN=NADKD1 - [NAKD1\_HUMAN]** | | | | | | | | | | | | | | | | | | | | | | | | | | | | | | |  |
|  | | | | | | | | | | | | | | | | | | | | | | | | | | | | | | | | |
|  | High |  | YAELSEEDLK |  | CID |  |  |  |  |  | 1.57 |  | 1.00 |  | 1 |  | SEQUEST (4) |  | 2 |  | 598.78809 |  | 0.61 |  | 1207 |  | 1207 |  | Image |  | Peak List |  |
|  | | | | | | | | | | | | | | | | | | | | | | | | | | | | | | | | |
|  | **Q6ZRV2 - Protein FAM83H OS=Homo sapiens GN=FAM83H PE=1 SV=3 - [FA83H\_HUMAN]** | | | | | | | | | | | | | | | | | | | | | | | | | | | | | | |  |
|  | | | | | | | | | | | | | | | | | | | | | | | | | | | | | | | | |
|  | High |  | mDAYALAPYAGAGPLVGVPGVGAPTPFSFPK |  | CID |  | M1(Oxidation) |  |  |  | 2.29 |  | 1.00 |  | 1 |  | SEQUEST (4) |  | 4 |  | 759.38550 |  | -6.03 |  | 4692 |  | 4692 |  | Image |  | Peak List |  |
|  | | | | | | | | | | | | | | | | | | | | | | | | | | | | | | | | |
|  | **Q7Z5P9-2 - Isoform 2 of Mucin-19 OS=Homo sapiens GN=MUC19 - [MUC19\_HUMAN]** | | | | | | | | | | | | | | | | | | | | | | | | | | | | | | |  |
|  | | | | | | | | | | | | | | | | | | | | | | | | | | | | | | | | |
|  | High |  | TGTTGQSGAESGTTEPSAR |  | CID |  |  |  |  |  | 1.85 |  | 1.00 |  | 1 |  | SEQUEST (4) |  | 3 |  | 598.94385 |  | 3.83 |  | 2067 |  | 2067 |  | Image |  | Peak List |  |
|  | | | | | | | | | | | | | | | | | | | | | | | | | | | | | | | | |
|  | **Q96DG6 - Carboxymethylenebutenolidase homolog OS=Homo sapiens GN=CMBL PE=1 SV=1 - [CMBL\_HUMAN]** | | | | | | | | | | | | | | | | | | | | | | | | | | | | | | |  |
|  | | | | | | | | | | | | | | | | | | | | | | | | | | | | | | | | |
|  | High |  | NLIEWLNK |  | CID |  |  |  |  |  | 1.88 |  | 1.00 |  | 1 |  | SEQUEST (4) |  | 2 |  | 515.29144 |  | 2.73 |  | 2377 |  | 2377 |  | Image |  | Peak List |  |
|  | | | | | | | | | | | | | | | | | | | | | | | | | | | | | | | | |
|  | **Q9P1F3 - Costars family protein ABRACL OS=Homo sapiens GN=ABRACL PE=1 SV=1 - [ABRAL\_HUMAN]** | | | | | | | | | | | | | | | | | | | | | | | | | | | | | | |  |
|  | | | | | | | | | | | | | | | | | | | | | | | | | | | | | | | | |
|  | High |  | IVTYPGELLLQGVHDDVDIILLQD |  | CID |  |  |  |  |  | 2.31 |  | 1.00 |  | 1 |  | SEQUEST (4) |  | 3 |  | 893.48706 |  | 6.64 |  | 4497 |  | 4497 |  | Image |  | Peak List |  |
|  | | | | | | | | | | | | | | | | | | | | | | | | | | | | | | | | |
|  | **B3KQU2 - Protein disulfide-isomerase A2 OS=Homo sapiens GN=PDIA2 PE=2 SV=1 - [B3KQU2\_HUMAN]** | | | | | | | | | | | | | | | | | | | | | | | | | | | | | | |  |
|  | | | | | | | | | | | | | | | | | | | | | | | | | | | | | | | | |
|  | High |  | ADFPVDEELGLDLGDLSR |  | CID |  |  |  |  |  | 1.60 |  | 1.00 |  | 1 |  | SEQUEST (4) |  | 3 |  | 654.32281 |  | 2.01 |  | 967 |  | 967 |  | Image |  | Peak List |  |
|  | | | | | | | | | | | | | | | | | | | | | | | | | | | | | | | | |
|  | **H0YKG9 - EP300-interacting inhibitor of differentiation 1 OS=Homo sapiens GN=EID1 PE=4 SV=1 - [H0YKG9\_HUMAN]** | | | | | | | | | | | | | | | | | | | | | | | | | | | | | | |  |
|  | | | | | | | | | | | | | | | | | | | | | | | | | | | | | | | | |
|  | High |  | TPFDQLAFIEELFSLmVVNRLTEELGCDEIIDRE |  | CID |  | M16(Oxidation) |  |  |  | 1.81 |  | 1.00 |  | 1 |  | SEQUEST (4) |  | 3 |  | 1334.33667 |  | 9.85 |  | 5780 |  | 5780 |  | Image |  | Peak List |  |
|  | | | | | | | | | | | | | | | | | | | | | | | | | | | | | | | | |

  
Top
  

### Search Summary

Workflow created with Discoverer version: 1.4.0.288 (DBVersion:79)
  
  
================================================================================
  
  
Search name: Methanol\_50\_2\_MC3
  
Search description: -
  
Search date: 06/25/2014 16:30:45
  
  
================================================================================
  
  
The pipeline tree:
  
------------------
  
  
    |-(0) Spectrum Files
  
        |-(1) Spectrum Selector
  
            |-(2) Mascot
  
                |-(3) Percolator
  
            |-(4) SEQUEST
  
                |-(3) Percolator
  
  
================================================================================
  
  
Search name: Methanol\_50\_2\_MC3
  
Search description: -
  
Search date: 06/25/2014 16:30:45
  
  
================================================================================
  
  
The pipeline tree:
  
------------------
  
  
    |-(0) Spectrum Files
  
        |-(1) Spectrum Selector
  
            |-(2) Mascot
  
                |-(3) Percolator
  
            |-(4) SEQUEST
  
                |-(3) Percolator
  
  
------------------------------------------------------------------------------
  
Processing node 0: Spectrum Files
  
------------------------------------------------------------------------------
  
  
Input Data:
  
-----------------------------
  
File Name(s): E:\Jos\Methanol\_50\_2.raw
  
  
------------------------------------------------------------------------------
  
Processing node 1: Spectrum Selector
  
------------------------------------------------------------------------------
  
  
1. General Settings:
  
-----------------------------
  
Precursor Selection: Use MS1 Precursor
  
Use New Precursor Reevaluation: True
  
  
2. Spectrum Properties Filter:
  
-----------------------------
  
Lower RT Limit: 0
  
Upper RT Limit: 0
  
First Scan: 0
  
Last Scan: 0
  
Lowest Charge State: 0
  
Highest Charge State: 0
  
Min. Precursor Mass: 350 Da
  
Max. Precursor Mass: 5000 Da
  
Total Intensity Threshold: 0
  
Minimum Peak Count: 1
  
  
3. Scan Event Filters:
  
-----------------------------
  
MS Order: Is MS2
  
Activation Type: Is CID
  
Min. Collision Energy: 0
  
Max. Collision Energy: 1000
  
Scan Type: Is Full
  
Ionization Source: Is Nanospray
  
  
4. Peak Filters:
  
-----------------------------
  
S/N Threshold (FT-only): 1.5
  
  
5. Replacements for Unrecognized Properties:
  
-----------------------------
  
Unrecognized Charge Replacements: Automatic
  
Unrecognized Mass Analyzer Replacements: ITMS
  
Unrecognized MS Order Replacements: MS2
  
Unrecognized Activation Type Replacements: CID
  
Unrecognized Polarity Replacements: +
  
  
6. Just for Testing:
  
-----------------------------
  
Precursor Clipping Range Before: 2.5 Da
  
Precursor Clipping Range After: 5.5 Da
  
  
------------------------------------------------------------------------------
  
Processing node 2: Mascot
  
------------------------------------------------------------------------------
  
  
1. Input Data:
  
-----------------------------
  
Protein Database: SwissProt
  
Enzyme Name: Trypsin
  
Maximum Missed Cleavage Sites: 3
  
Instrument: Default
  
Taxonomy: . . . . . . . . . . . . . . . . Homo sapiens (human)
  
  
1.1 Peptide Scoring Options:
  
-----------------------------
  
Peptide Cut Off Score: 10
  
Peptide Without Protein Cut Off Score: 5
  
  
1.2 Protein Scoring Options:
  
-----------------------------
  
Use MudPIT Scoring: Automatic
  
Protein Relevance Threshold: 20
  
Protein Relevance Factor: 1
  
  
2. Tolerances:
  
-----------------------------
  
Precursor Mass Tolerance: 10 ppm
  
Fragment Mass Tolerance: 0.8 Da
  
Use Average Precursor Mass: False
  
  
4. Dynamic Modifications:
  
-----------------------------
  
1. Dynamic Modification: Oxidation (M)
  
  
------------------------------------------------------------------------------
  
Processing node 3: Percolator
  
------------------------------------------------------------------------------
  
  
1. Input Data:
  
-----------------------------
  
Maximum Delta Cn: 0.05
  
  
2. Decoy Database Search:
  
-----------------------------
  
Target FDR (Strict): 0.01
  
Target FDR (Relaxed): 0.05
  
Validation based on: q-Value
  
  
------------------------------------------------------------------------------
  
Processing node 4: SEQUEST
  
------------------------------------------------------------------------------
  
  
1. Input Data:
  
-----------------------------
  
Protein Database: HUMAN\_swiss\_Jos.fasta
  
Enzyme Name: Trypsin (Full)
  
Maximum Missed Cleavage Sites: 3
  
  
1.1 Peptide Scoring Options:
  
-----------------------------
  
Maximum Peptides Considered: 500
  
Maximum Peptides Output: 10
  
Calculate Probability Scores: False
  
Absolute XCorr Threshold: 0.4
  
Fragment Ion Cutoff Percentage: 0.1
  
Peptide Without Protein XCorr Threshold: 1.5
  
  
1.2 Protein Scoring Options:
  
-----------------------------
  
Maximum Protein References Per Peptide: 100
  
Protein Relevance Threshold: 1.5
  
Peptide Relevance Factor: 0.4
  
  
2. Tolerances:
  
-----------------------------
  
Precursor Mass Tolerance: 10 ppm
  
Fragment Mass Tolerance: 0.8 Da
  
Use Average Precursor Mass: False
  
Use Average Fragment Masses: False
  
  
3. Ion Series:
  
-----------------------------
  
Use Neutral Loss a Ions: True
  
Use Neutral Loss b Ions: True
  
Use Neutral Loss y Ions: True
  
Weight of a Ions: 0
  
Weight of b Ions: 1
  
Weight of c Ions: 0
  
Weight of x Ions: 0
  
Weight of y Ions: 1
  
Weight of z Ions: 0
  
  
4. Dynamic Modifications:
  
-----------------------------
  
Max. Modifications Per Peptide: 4
  
1. Dynamic Modification: Oxidation / +15.995 Da (M)
  
  
================================================================================
  
  
Processing details:
  
  
06/25/2014 04:45 PM (4):SEQUEST: Total search time was 1 min 39 s.
  
06/25/2014 04:45 PM (3):Percolator: Performing percolator for SEQUEST (4) took 46.5 s.
  
06/25/2014 04:45 PM (4):SEQUEST: Search completed
  
06/25/2014 04:45 PM (4):SEQUEST: 8444 protein(s) + 8034 decoy proteins scored and inserted into result file in 4.9 s.
  
06/25/2014 04:45 PM (4):SEQUEST: 8444 protein(s) scored
  
06/25/2014 04:45 PM (4):SEQUEST: Search result finalization started.
  
06/25/2014 04:45 PM (3):Percolator: Start reading Percolator results
  
06/25/2014 04:45 PM (3):Percolator: Processing took 5.569 cpu seconds or 6 seconds wall time
  
06/25/2014 04:45 PM (3):Percolator: Calibrating statistics - calculating Posterior error probabilities (PEPs)
  
06/25/2014 04:45 PM (3):Percolator: PSMId score q-value posterior\_error\_prob peptide proteinIds
  
06/25/2014 04:45 PM (3):Percolator: New pi\_0 estimate on merged list gives 783 peptides over q=0.0100
  
06/25/2014 04:45 PM (3):Percolator: Calibrating statistics - calculating q values
  
06/25/2014 04:45 PM (3):Percolator: Selecting pi\_0=0.7805
  
06/25/2014 04:45 PM (3):Percolator: Tossing out "redundant" PSMs keeping only the best scoring PSM for each unique peptide.
  
06/25/2014 04:45 PM (3):Percolator: Merging results from 3 datasets
  
06/25/2014 04:45 PM (3):Percolator: Found 1157 target PSMs scoring over 1.0000% FDR level on testset
  
06/25/2014 04:45 PM (3):Percolator: 1.1680 -0.0017 -0.5269 0.0415 0.0142 -0.0007 -15.0266 0.0818 89.7585 -0.2929 -0.0409 0.0000 -0.9666 0.1550 1.6380 0.0161 0.3909 -1.1484 0.0000 -0.6863 0.0318 -0.0506 0.0037 -0.6090 0.3853 0.3317 0.2045 -0.2375 -0.0011 -10.0869 0.0022 -6.6007 0.0021 2.3490
  
06/25/2014 04:45 PM (3):Percolator: 1.05 -0.5389 -0.1468 1.5115 0.3893 -0.6524 -0.2224 0.4549 0.8014 -0.8455 -0.3346 0.0000 -0.4712 0.0769 0.5515 0.0027 0.0461 -1.2535 0.0000 -0.4091 0.4016 -0.9403 0.0726 -1.6166 1.0293 0.7173 0.5429 -0.0488 -0.3347 -1.0314 0.3770 -0.6319 0.4089 -3.7530
  
06/25/2014 04:45 PM (3):Percolator: XCorr SpScore Delta Cn From Second PSM Binomial Score Isolation Interference [%] MH+ [Da] Delta Mass [Da] Delta Mass [ppm] Absolute Delta Mass [Da] Absolute Delta Mass [ppm] Peptide Length Is z=1 Is z=2 Is z=3 Is z=4 Is z=5 Is z>5 # Missed Cleavages Log Peptides Matched Log Total Intensity Fraction Matched Intensity [%] Fragment Coverage Series A, B, C [%] Fragment Coverage Series X, Y, Z [%] Log Matched Fragment Series Intensities A, B, C Log Matched Fragment Series Intensities X, Y, Z Longest Sequence Series A, B, C Longest Sequence Series X, Y, Z IQR Fragment Delta Mass [Da] IQR Fragment Delta Mass [ppm] Mean Fragment Delta Mass [Da] Mean Fragment Delta Mass [ppm] Mean Absolute Fragment Delta Mass [Da] Mean Absolute Fragment Delta Mass [ppm] m0
  
06/25/2014 04:45 PM (3):Percolator: # first line contains normalized weights, second line the raw weights
  
06/25/2014 04:45 PM (3):Percolator: Obtained weights (only showing weights of first cross validation set)
  
06/25/2014 04:45 PM (3):Percolator: Iteration 10 : After the iteration step, 1234 target PSMs with q<0.01 were estimated by cross validation
  
06/25/2014 04:45 PM (3):Percolator: Iteration 9 : After the iteration step, 1235 target PSMs with q<0.01 were estimated by cross validation
  
06/25/2014 04:45 PM (3):Percolator: Iteration 8 : After the iteration step, 1234 target PSMs with q<0.01 were estimated by cross validation
  
06/25/2014 04:45 PM (3):Percolator: Iteration 7 : After the iteration step, 1234 target PSMs with q<0.01 were estimated by cross validation
  
06/25/2014 04:45 PM (3):Percolator: Iteration 6 : After the iteration step, 1232 target PSMs with q<0.01 were estimated by cross validation
  
06/25/2014 04:45 PM (3):Percolator: Iteration 5 : After the iteration step, 1230 target PSMs with q<0.01 were estimated by cross validation
  
06/25/2014 04:45 PM (3):Percolator: Iteration 4 : After the iteration step, 1224 target PSMs with q<0.01 were estimated by cross validation
  
06/25/2014 04:45 PM (3):Percolator: Iteration 3 : After the iteration step, 1220 target PSMs with q<0.01 were estimated by cross validation
  
06/25/2014 04:45 PM (3):Percolator: Iteration 2 : After the iteration step, 1202 target PSMs with q<0.01 were estimated by cross validation
  
06/25/2014 04:45 PM (3):Percolator: Iteration 1 : After the iteration step, 1152 target PSMs with q<0.01 were estimated by cross validation
  
06/25/2014 04:45 PM (3):Percolator: ---Training with Cpos selected by cross validation, Cneg selected by cross validation, fdr=0.01
  
06/25/2014 04:45 PM (3):Percolator: Reading in data and feature calculation took 7.035 cpu seconds or 7 seconds wall time
  
06/25/2014 04:45 PM (3):Percolator: Estimating 709 over q=0.01 in initial direction
  
06/25/2014 04:45 PM (3):Percolator: Selected feature number 1 as initial search direction, could separate 494 positives in that direction
  
06/25/2014 04:45 PM (3):Percolator: Selected feature number 27 as initial search direction, could separate 508 positives in that direction
  
06/25/2014 04:45 PM (3):Percolator: Selected feature number 27 as initial search direction, could separate 513 positives in that direction
  
06/25/2014 04:45 PM (3):Percolator: selecting cneg by cross validation
  
06/25/2014 04:45 PM (3):Percolator: selecting cpos by cross validation
  
06/25/2014 04:45 PM (3):Percolator: Train/test set contains 4418 positives and 4817 negatives, size ratio=0.917168 and pi0=1
  
06/25/2014 04:45 PM (3):Percolator: 31e77142-29e9-402c-9ec2-8468a9513af0 e39a792e-622c-452d-b49b-59809cad79d0 Delta Cn From Second PSM Binomial Score b8754504-e95e-476b-b9a4-454d4bb53aeb 1d91a87b-953a-4887-9f22-f75a497a3538 Delta Mass [Da] Delta Mass [ppm] Absolute Delta Mass [Da] Absolute Delta Mass [ppm] Peptide Length Is z=1 Is z=2 Is z=3 Is z=4 Is z=5 Is z>5 041eb6d5-e486-44a0-9bc1-19e25811c686 Log Peptides Matched Log Total Intensity Fraction Matched Intensity [%] Fragment Coverage Series A, B, C [%] Fragment Coverage Series X, Y, Z [%] Log Matched Fragment Series Intensities A, B, C Log Matched Fragment Series Intensities X, Y, Z Longest Sequence Series A, B, C Longest Sequence Series X, Y, Z IQR Fragment Delta Mass [Da] IQR Fragment Delta Mass [ppm] Mean Fragment Delta Mass [Da] Mean Fragment Delta Mass [ppm] Mean Absolute Fragment Delta Mass [Da] Mean Absolute Fragment Delta Mass [ppm]
  
06/25/2014 04:45 PM (3):Percolator: Features:
  
06/25/2014 04:45 PM (3):Percolator: enzyme=Trypsin
  
06/25/2014 04:45 PM (3):Percolator: Hyperparameters fdr=0.01, Cpos=0, Cneg=0, maxNiter=10
  
06/25/2014 04:45 PM (3):Percolator: Started Wed Jun 25 16:45:29 2014
  
06/25/2014 04:45 PM (3):Percolator: C:\Program Files\Thermo\Discoverer 1.4\Tools\Percolator\percolator.exe -X C:\ProgramData\Thermo\Discoverer 1.4\Scratch\f7aa7d37-e597-4db7-92d0-d8a702971aa0\output.xml -Z C:\ProgramData\Thermo\Discoverer 1.4\Scratch\f7aa7d37-e597-4db7-92d0-d8a702971aa0\input.xml
  
06/25/2014 04:45 PM (3):Percolator: Issued command:
  
06/25/2014 04:45 PM (3):Percolator: Department of Genome Sciences at the University of Washington.
  
06/25/2014 04:45 PM (3):Percolator: Written by Lukas K+�ll (lukall@u.washington.edu) in the
  
06/25/2014 04:45 PM (3):Percolator: Copyright (c) 2006-9 University of Washington. All rights reserved.
  
06/25/2014 04:45 PM (3):Percolator: Percolator version 2.04, Build Date Feb 1 2012 03:35:34
  
06/25/2014 04:45 PM (3):Percolator: Starting Percolator
  
06/25/2014 04:45 PM (3):Percolator: The input file contains 4418 peptides, 4817 decoy peptides and 33 features.
  
06/25/2014 04:45 PM (3):Percolator: Creating input file for SEQUEST (4) took 31.8 s.
  
06/25/2014 04:44 PM (3):Percolator: Start calculating features for peptides of SEQUEST (4)
  
06/25/2014 04:44 PM (2):Mascot: Total search time was 2 min 9 s.
  
06/25/2014 04:44 PM (3):Percolator: Performing percolator for Mascot (2) took 42.5 s.
  
06/25/2014 04:44 PM (2):Mascot: Search completed
  
06/25/2014 04:44 PM (2):Mascot: 216 protein(s) + 37 decoy proteins scored and inserted into result file in 0.7 s.
  
06/25/2014 04:44 PM (2):Mascot: 216 protein(s) scored
  
06/25/2014 04:44 PM (2):Mascot: Search result finalization started.
  
06/25/2014 04:44 PM (3):Percolator: Start reading Percolator results
  
06/25/2014 04:44 PM (3):Percolator: Processing took 7.847 cpu seconds or 7 seconds wall time
  
06/25/2014 04:44 PM (3):Percolator: PSMId score q-value posterior\_error\_prob peptide proteinIds
  
06/25/2014 04:44 PM (3):Percolator: Calibrating statistics - calculating Posterior error probabilities (PEPs)
  
06/25/2014 04:44 PM (3):Percolator: New pi\_0 estimate on merged list gives 786 peptides over q=0.0100
  
06/25/2014 04:44 PM (3):Percolator: Calibrating statistics - calculating q values
  
06/25/2014 04:44 PM (3):Percolator: Selecting pi\_0=0.7284
  
06/25/2014 04:44 PM (3):Percolator: Tossing out "redundant" PSMs keeping only the best scoring PSM for each unique peptide.
  
06/25/2014 04:44 PM (3):Percolator: Merging results from 3 datasets
  
06/25/2014 04:44 PM (3):Percolator: Found 1138 target PSMs scoring over 1.0000% FDR level on testset
  
06/25/2014 04:44 PM (3):Percolator: 0.0230 0.4200 0.0083 0.0021 0.0001 12.7983 0.0071 23.9858 -0.1167 -0.0153 0.0000 -0.3067 0.3669 -0.0807 -0.0959 -0.6511 -0.4869 0.0000 -0.2361 0.0012 -0.0085 -0.0030 -0.0222 0.1042 0.1264 0.2086 -0.0691 -0.0002 -1.2013 -0.0004 -3.9117 0.0000 -0.2289
  
06/25/2014 04:44 PM (3):Percolator: 0.35 0.1260 0.3227 0.0575 0.0764 0.2059 0.0389 0.2400 -0.3382 -0.1486 0.0000 -0.1444 0.1831 -0.0284 -0.0179 -0.0810 -0.5394 0.0000 -0.1932 0.0182 -0.1809 -0.0704 -0.1267 0.3070 0.3009 0.6253 -0.0163 -0.0759 -0.1453 -0.0721 -0.4401 0.0023 -1.6455
  
06/25/2014 04:44 PM (3):Percolator: IonScore Delta Cn From Second PSM Binomial Score Isolation Interference [%] MH+ [Da] Delta Mass [Da] Delta Mass [ppm] Absolute Delta Mass [Da] Absolute Delta Mass [ppm] Peptide Length Is z=1 Is z=2 Is z=3 Is z=4 Is z=5 Is z>5 # Missed Cleavages Log Peptides Matched Log Total Intensity Fraction Matched Intensity [%] Fragment Coverage Series A, B, C [%] Fragment Coverage Series X, Y, Z [%] Log Matched Fragment Series Intensities A, B, C Log Matched Fragment Series Intensities X, Y, Z Longest Sequence Series A, B, C Longest Sequence Series X, Y, Z IQR Fragment Delta Mass [Da] IQR Fragment Delta Mass [ppm] Mean Fragment Delta Mass [Da] Mean Fragment Delta Mass [ppm] Mean Absolute Fragment Delta Mass [Da] Mean Absolute Fragment Delta Mass [ppm] m0
  
06/25/2014 04:44 PM (3):Percolator: # first line contains normalized weights, second line the raw weights
  
06/25/2014 04:44 PM (3):Percolator: Obtained weights (only showing weights of first cross validation set)
  
06/25/2014 04:44 PM (3):Percolator: Iteration 10 : After the iteration step, 1192 target PSMs with q<0.01 were estimated by cross validation
  
06/25/2014 04:44 PM (3):Percolator: Iteration 9 : After the iteration step, 1191 target PSMs with q<0.01 were estimated by cross validation
  
06/25/2014 04:44 PM (3):Percolator: Iteration 8 : After the iteration step, 1190 target PSMs with q<0.01 were estimated by cross validation
  
06/25/2014 04:44 PM (3):Percolator: Iteration 7 : After the iteration step, 1190 target PSMs with q<0.01 were estimated by cross validation
  
06/25/2014 04:44 PM (3):Percolator: Iteration 6 : After the iteration step, 1189 target PSMs with q<0.01 were estimated by cross validation
  
06/25/2014 04:44 PM (3):Percolator: Iteration 5 : After the iteration step, 1189 target PSMs with q<0.01 were estimated by cross validation
  
06/25/2014 04:44 PM (3):Percolator: Iteration 4 : After the iteration step, 1187 target PSMs with q<0.01 were estimated by cross validation
  
06/25/2014 04:44 PM (3):Percolator: Iteration 3 : After the iteration step, 1185 target PSMs with q<0.01 were estimated by cross validation
  
06/25/2014 04:44 PM (3):Percolator: Iteration 2 : After the iteration step, 1181 target PSMs with q<0.01 were estimated by cross validation
  
06/25/2014 04:44 PM (3):Percolator: Iteration 1 : After the iteration step, 1150 target PSMs with q<0.01 were estimated by cross validation
  
06/25/2014 04:44 PM (3):Percolator: ---Training with Cpos selected by cross validation, Cneg selected by cross validation, fdr=0.01
  
06/25/2014 04:44 PM (3):Percolator: Reading in data and feature calculation took 5.319 cpu seconds or 6 seconds wall time
  
06/25/2014 04:44 PM (3):Percolator: Estimating 826 over q=0.01 in initial direction
  
06/25/2014 04:44 PM (3):Percolator: Selected feature number 1 as initial search direction, could separate 558 positives in that direction
  
06/25/2014 04:44 PM (3):Percolator: Selected feature number 1 as initial search direction, could separate 543 positives in that direction
  
06/25/2014 04:44 PM (3):Percolator: Selected feature number 1 as initial search direction, could separate 552 positives in that direction
  
06/25/2014 04:44 PM (3):Percolator: selecting cneg by cross validation
  
06/25/2014 04:44 PM (3):Percolator: selecting cpos by cross validation
  
06/25/2014 04:44 PM (3):Percolator: Train/test set contains 3605 positives and 3521 negatives, size ratio=1.02386 and pi0=1
  
06/25/2014 04:44 PM (3):Percolator: e6e22773-e9a6-4a26-9694-1ca77a797099 Delta Cn From Second PSM Binomial Score b8754504-e95e-476b-b9a4-454d4bb53aeb 1d91a87b-953a-4887-9f22-f75a497a3538 Delta Mass [Da] Delta Mass [ppm] Absolute Delta Mass [Da] Absolute Delta Mass [ppm] Peptide Length Is z=1 Is z=2 Is z=3 Is z=4 Is z=5 Is z>5 041eb6d5-e486-44a0-9bc1-19e25811c686 Log Peptides Matched Log Total Intensity Fraction Matched Intensity [%] Fragment Coverage Series A, B, C [%] Fragment Coverage Series X, Y, Z [%] Log Matched Fragment Series Intensities A, B, C Log Matched Fragment Series Intensities X, Y, Z Longest Sequence Series A, B, C Longest Sequence Series X, Y, Z IQR Fragment Delta Mass [Da] IQR Fragment Delta Mass [ppm] Mean Fragment Delta Mass [Da] Mean Fragment Delta Mass [ppm] Mean Absolute Fragment Delta Mass [Da] Mean Absolute Fragment Delta Mass [ppm]
  
06/25/2014 04:44 PM (3):Percolator: Features:
  
06/25/2014 04:44 PM (3):Percolator: enzyme=Trypsin
  
06/25/2014 04:44 PM (3):Percolator: Hyperparameters fdr=0.01, Cpos=0, Cneg=0, maxNiter=10
  
06/25/2014 04:44 PM (3):Percolator: Started Wed Jun 25 16:44:40 2014
  
06/25/2014 04:44 PM (3):Percolator: C:\Program Files\Thermo\Discoverer 1.4\Tools\Percolator\percolator.exe -X C:\ProgramData\Thermo\Discoverer 1.4\Scratch\1bcac45e-00b3-4b83-ba7c-699cd5afad1e\output.xml -Z C:\ProgramData\Thermo\Discoverer 1.4\Scratch\1bcac45e-00b3-4b83-ba7c-699cd5afad1e\input.xml
  
06/25/2014 04:44 PM (3):Percolator: Issued command:
  
06/25/2014 04:44 PM (3):Percolator: Department of Genome Sciences at the University of Washington.
  
06/25/2014 04:44 PM (3):Percolator: Written by Lukas K+�ll (lukall@u.washington.edu) in the
  
06/25/2014 04:44 PM (3):Percolator: Copyright (c) 2006-9 University of Washington. All rights reserved.
  
06/25/2014 04:44 PM (3):Percolator: Percolator version 2.04, Build Date Feb 1 2012 03:35:34
  
06/25/2014 04:44 PM (3):Percolator: Starting Percolator
  
06/25/2014 04:44 PM (3):Percolator: The input file contains 3605 peptides, 3521 decoy peptides and 32 features.
  
06/25/2014 04:44 PM (3):Percolator: Creating input file for Mascot (2) took 27.7 s.
  
06/25/2014 04:44 PM (3):Percolator: Start calculating features for peptides of Mascot (2)
  
06/25/2014 04:44 PM (2):Mascot: Used mascot server http://fenn.bham.ac.uk/mascot/ with Mascot version 2.4.1
  
06/25/2014 04:44 PM (2):Mascot: Sending 3163 peptide hits (13312 peptides) to result file
  
06/25/2014 04:44 PM (2):Mascot: Sending 2894 decoy peptide hits (12175 peptides) to result file
  
06/25/2014 04:44 PM (2):Mascot: Reading decoy results
  
06/25/2014 04:44 PM (2):Mascot: Start translating results
  
06/25/2014 04:44 PM (2):Mascot: Start mapping modifications
  
06/25/2014 04:44 PM (2):Mascot: Received 234 proteins from Mascot server
  
06/25/2014 04:44 PM (2):Mascot: Start mapping 234 proteins
  
06/25/2014 04:44 PM (2):Mascot: Start parsing results
  
06/25/2014 04:44 PM (2):Mascot: Received Mascot result file (filename=../data/20140625/F004277.dat)
  
06/25/2014 04:43 PM (2):Mascot: Mascot Server completed
  
06/25/2014 04:42 PM (2):Mascot: Mascot result on server (filename=../data/20140625/F004277.dat)
  
06/25/2014 04:42 PM (2):Mascot: Start searching 4617 spectra
  
06/25/2014 04:42 PM (4):SEQUEST: Sending 617 decoy peptide hits (2706 peptides) to result file
  
06/25/2014 04:42 PM (4):SEQUEST: Starting SEQUEST decoy search
  
06/25/2014 04:42 PM (4):SEQUEST: Sending 617 peptide hits (2681 peptides) to result file
  
06/25/2014 04:41 PM (4):SEQUEST: Starting SEQUEST (search spectra 4000 - 4617)
  
06/25/2014 04:41 PM (4):SEQUEST: Sending 1000 decoy peptide hits (4403 peptides) to result file
  
06/25/2014 04:41 PM (4):SEQUEST: Starting SEQUEST decoy search
  
06/25/2014 04:41 PM (4):SEQUEST: Sending 1000 peptide hits (4400 peptides) to result file
  
06/25/2014 04:41 PM (4):SEQUEST: Starting SEQUEST (search spectra 3000 - 4000)
  
06/25/2014 04:41 PM (4):SEQUEST: Sending 1000 decoy peptide hits (7169 peptides) to result file
  
06/25/2014 04:41 PM (4):SEQUEST: Starting SEQUEST decoy search
  
06/25/2014 04:41 PM (4):SEQUEST: Sending 1000 peptide hits (7192 peptides) to result file
  
06/25/2014 04:41 PM (4):SEQUEST: Starting SEQUEST (search spectra 2000 - 3000)
  
06/25/2014 04:41 PM (4):SEQUEST: Sending 1000 decoy peptide hits (9535 peptides) to result file
  
06/25/2014 04:40 PM (4):SEQUEST: Starting SEQUEST decoy search
  
06/25/2014 04:40 PM (4):SEQUEST: Sending 1000 peptide hits (9520 peptides) to result file
  
06/25/2014 04:40 PM (4):SEQUEST: Starting SEQUEST (search spectra 1000 - 2000)
  
06/25/2014 04:40 PM (4):SEQUEST: Sending 1000 decoy peptide hits (9697 peptides) to result file
  
06/25/2014 04:40 PM (4):SEQUEST: Starting SEQUEST decoy search
  
06/25/2014 04:40 PM (4):SEQUEST: Sending 1000 peptide hits (9691 peptides) to result file
  
06/25/2014 04:39 PM (4):SEQUEST: Starting SEQUEST (search spectra 0 - 1000)
  
06/25/2014 04:39 PM (4):SEQUEST: There is already an adequate decoy FASTA index.
  
06/25/2014 04:39 PM (4):SEQUEST: Looking for existing decoy FASTA index.
  
06/25/2014 04:39 PM (4):SEQUEST: There is already an adequate target FASTA index.
  
06/25/2014 04:39 PM (4):SEQUEST: Looking for existing target FASTA index.
  
06/25/2014 04:39 PM (2):Mascot: Use mascot server http://fenn.bham.ac.uk/mascot/ with Mascot version 2.4.1
  
06/25/2014 04:39 PM (1):Spectrum Selector: Reading from File 1 of 1:E:\Jos\Methanol\_50\_2.raw (7882 spectra total)
  
  
  
Top
