# Supplementary material for: Top-Down and Bottom-Up Identification of Proteins by Liquid Extraction Surface Analysis Mass Spectrometry of Healthy and Diseased Human Liver Tissue
Source: J Am Soc Mass Spectrom. 2014 Sep 3;25(11):1953–61. doi: 10.1007/s13361-014-0967-z (PMC4197381; doi:10.1007/s13361-014-0967-z)
Supplement: Supplementary file 8 — (ZIP 1626 kb) [file 13361_2014_967_MOESM8_ESM.zip › index.html]

Annotated spectra


## Annotated spectra of Methanol\_70\_1\_MC3.msf

go to Peptides  
go to Search Summary  

### Peptides

|  |  |  |  |  |  |  |  |  |  |  |  |  |  |  |  |  |  |  |  |  |  |  |  |  |  |  |  |  |  |  |  |  |
| --- | --- | --- | --- | --- | --- | --- | --- | --- | --- | --- | --- | --- | --- | --- | --- | --- | --- | --- | --- | --- | --- | --- | --- | --- | --- | --- | --- | --- | --- | --- | --- | --- |
|  | | | | | | | | | | | | | | | | | | | | | | | | | | | | | | | | |
| Confidence Sequence Activation Type Modifications IonScore XCorr ΔScore Rank Search Engine Charge Precursor m/z [Da] ΔM [ppm] First Scan Last Scan Annotated Spectrum Peak List  | | | | | | | | | | | | | | | | | | | | | | | | | | | | | | | | |
|  | | | | | | | | | | | | | | | | | | | | | | | | | | | | | | | | |
|  | **P00367 - Glutamate dehydrogenase 1, mitochondrial OS=Homo sapiens GN=GLUD1 PE=1 SV=2 - [DHE3\_HUMAN]** | | | | | | | | | | | | | | | | | | | | | | | | | | | | | | |  |
|  | | | | | | | | | | | | | | | | | | | | | | | | | | | | | | | | |
|  | High |  | YNLGLDLR |  | CID |  |  |  | 29.41 |  |  |  | 1.00 |  | 1 |  | Mascot (2) |  | 2 |  | 482.26669 |  | 0.28 |  | 1832 |  | 1832 |  | Image |  | Peak List |  |
|  | | | | | | | | | | | | | | | | | | | | | | | | | | | | | | | | |
|  | High |  | YNLGLDLR |  | CID |  |  |  |  |  | 1.87 |  | 1.00 |  | 1 |  | SEQUEST (4) |  | 2 |  | 482.26669 |  | 0.28 |  | 1832 |  | 1832 |  | Image |  | Peak List |  |
|  | | | | | | | | | | | | | | | | | | | | | | | | | | | | | | | | |
|  | **P00505 - Aspartate aminotransferase, mitochondrial OS=Homo sapiens GN=GOT2 PE=1 SV=3 - [AATM\_HUMAN]** | | | | | | | | | | | | | | | | | | | | | | | | | | | | | | |  |
|  | | | | | | | | | | | | | | | | | | | | | | | | | | | | | | | | |
|  | High |  | ILIRPMYSNPPLNGAR |  | CID |  |  |  | 20.77 |  |  |  | 1.00 |  | 1 |  | Mascot (2) |  | 3 |  | 604.67188 |  | 3.37 |  | 1427 |  | 1427 |  | Image |  | Peak List |  |
|  | | | | | | | | | | | | | | | | | | | | | | | | | | | | | | | | |
|  | High |  | ILIRPMYSNPPLNGAR |  | CID |  |  |  |  |  | 2.45 |  | 1.00 |  | 1 |  | SEQUEST (4) |  | 3 |  | 604.67163 |  | 2.96 |  | 1405 |  | 1405 |  | Image |  | Peak List |  |
|  | | | | | | | | | | | | | | | | | | | | | | | | | | | | | | | | |
|  | **P00738 - Haptoglobin OS=Homo sapiens GN=HP PE=1 SV=1 - [HPT\_HUMAN]** | | | | | | | | | | | | | | | | | | | | | | | | | | | | | | |  |
|  | | | | | | | | | | | | | | | | | | | | | | | | | | | | | | | | |
|  | High |  | VGYVSGWGR |  | CID |  |  |  | 26.42 |  |  |  | 1.00 |  | 1 |  | Mascot (2) |  | 2 |  | 490.75247 |  | 2.84 |  | 1206 |  | 1206 |  | Image |  | Peak List |  |
|  | | | | | | | | | | | | | | | | | | | | | | | | | | | | | | | | |
|  | High |  | VGYVSGWGR |  | CID |  |  |  |  |  | 1.78 |  | 1.00 |  | 1 |  | SEQUEST (4) |  | 2 |  | 490.75247 |  | 2.84 |  | 1206 |  | 1206 |  | Image |  | Peak List |  |
|  | | | | | | | | | | | | | | | | | | | | | | | | | | | | | | | | |
|  | **P00915 - Carbonic anhydrase 1 OS=Homo sapiens GN=CA1 PE=1 SV=2 - [CAH1\_HUMAN]** | | | | | | | | | | | | | | | | | | | | | | | | | | | | | | |  |
|  | | | | | | | | | | | | | | | | | | | | | | | | | | | | | | | | |
|  | High |  | LYPIANGNNQSPVDIK |  | CID |  |  |  | 34.63 |  |  |  | 1.00 |  | 1 |  | Mascot (2) |  | 2 |  | 871.95831 |  | 1.21 |  | 1597 |  | 1597 |  | Image |  | Peak List |  |
|  | | | | | | | | | | | | | | | | | | | | | | | | | | | | | | | | |
|  | High |  | LYPIANGNNQSPVDIK |  | CID |  |  |  |  |  | 3.61 |  | 1.00 |  | 1 |  | SEQUEST (4) |  | 2 |  | 871.95831 |  | 1.21 |  | 1597 |  | 1597 |  | Image |  | Peak List |  |
|  | | | | | | | | | | | | | | | | | | | | | | | | | | | | | | | | |
|  | **P01009 - Alpha-1-antitrypsin OS=Homo sapiens GN=SERPINA1 PE=1 SV=3 - [A1AT\_HUMAN]** | | | | | | | | | | | | | | | | | | | | | | | | | | | | | | |  |
|  | | | | | | | | | | | | | | | | | | | | | | | | | | | | | | | | |
|  | High |  | DTEEEDFHVDQVTTVK |  | CID |  |  |  | 65.40 |  |  |  | 1.00 |  | 1 |  | Mascot (2) |  | 2 |  | 946.43494 |  | 3.66 |  | 1299 |  | 1299 |  | Image |  | Peak List |  |
|  | | | | | | | | | | | | | | | | | | | | | | | | | | | | | | | | |
|  | High |  | DTEEEDFHVDQVTTVK |  | CID |  |  |  |  |  | 4.93 |  | 1.00 |  | 1 |  | SEQUEST (4) |  | 2 |  | 946.43494 |  | 3.66 |  | 1299 |  | 1299 |  | Image |  | Peak List |  |
|  | | | | | | | | | | | | | | | | | | | | | | | | | | | | | | | | |
|  | **P01040 - Cystatin-A OS=Homo sapiens GN=CSTA PE=1 SV=1 - [CYTA\_HUMAN]** | | | | | | | | | | | | | | | | | | | | | | | | | | | | | | |  |
|  | | | | | | | | | | | | | | | | | | | | | | | | | | | | | | | | |
|  | High |  | NKDDELTGF |  | CID |  |  |  | 42.06 |  |  |  | 1.00 |  | 1 |  | Mascot (2) |  | 2 |  | 519.74109 |  | 0.98 |  | 1325 |  | 1325 |  | Image |  | Peak List |  |
|  | | | | | | | | | | | | | | | | | | | | | | | | | | | | | | | | |
|  | **P01877 - Ig alpha-2 chain C region OS=Homo sapiens GN=IGHA2 PE=1 SV=3 - [IGHA2\_HUMAN]** | | | | | | | | | | | | | | | | | | | | | | | | | | | | | | |  |
|  | | | | | | | | | | | | | | | | | | | | | | | | | | | | | | | | |
|  | High |  | WLQGSQELPR |  | CID |  |  |  | 23.86 |  |  |  | 1.00 |  | 1 |  | Mascot (2) |  | 2 |  | 607.32202 |  | 3.56 |  | 1259 |  | 1259 |  | Image |  | Peak List |  |
|  | | | | | | | | | | | | | | | | | | | | | | | | | | | | | | | | |
|  | **P07900 - Heat shock protein HSP 90-alpha OS=Homo sapiens GN=HSP90AA1 PE=1 SV=5 - [HS90A\_HUMAN]** | | | | | | | | | | | | | | | | | | | | | | | | | | | | | | |  |
|  | | | | | | | | | | | | | | | | | | | | | | | | | | | | | | | | |
|  | High |  | DLVILLYETALLSSGFSLEDPQTHANR |  | CID |  |  |  | 65.16 |  |  |  | 1.00 |  | 1 |  | Mascot (2) |  | 3 |  | 1001.52917 |  | 8.63 |  | 4582 |  | 4582 |  | Image |  | Peak List |  |
|  | | | | | | | | | | | | | | | | | | | | | | | | | | | | | | | | |
|  | High |  | DLVILLYETALLSSGFSLEDPQTHANR |  | CID |  |  |  |  |  | 3.54 |  | 1.00 |  | 1 |  | SEQUEST (4) |  | 3 |  | 1001.52917 |  | 8.63 |  | 4582 |  | 4582 |  | Image |  | Peak List |  |
|  | | | | | | | | | | | | | | | | | | | | | | | | | | | | | | | | |
|  | **P09417 - Dihydropteridine reductase OS=Homo sapiens GN=QDPR PE=1 SV=2 - [DHPR\_HUMAN]** | | | | | | | | | | | | | | | | | | | | | | | | | | | | | | |  |
|  | | | | | | | | | | | | | | | | | | | | | | | | | | | | | | | | |
|  | High |  | SMPEADFSSWTPLEFLVETFHDWITGK |  | CID |  |  |  | 20.03 |  |  |  | 1.00 |  | 1 |  | Mascot (2) |  | 3 |  | 1057.50708 |  | 7.88 |  | 5608 |  | 5608 |  | Image |  | Peak List |  |
|  | | | | | | | | | | | | | | | | | | | | | | | | | | | | | | | | |
|  | High |  | SMPEADFSSWTPLEFLVETFHDWITGK |  | CID |  |  |  |  |  | 1.71 |  | 1.00 |  | 1 |  | SEQUEST (4) |  | 3 |  | 1057.50684 |  | 7.65 |  | 5657 |  | 5657 |  | Image |  | Peak List |  |
|  | | | | | | | | | | | | | | | | | | | | | | | | | | | | | | | | |
|  | **P10599 - Thioredoxin OS=Homo sapiens GN=TXN PE=1 SV=3 - [THIO\_HUMAN]** | | | | | | | | | | | | | | | | | | | | | | | | | | | | | | |  |
|  | | | | | | | | | | | | | | | | | | | | | | | | | | | | | | | | |
|  | High |  | EKLEATINELV |  | CID |  |  |  | 33.82 |  |  |  | 1.00 |  | 1 |  | Mascot (2) |  | 2 |  | 629.84918 |  | 1.68 |  | 2171 |  | 2171 |  | Image |  | Peak List |  |
|  | | | | | | | | | | | | | | | | | | | | | | | | | | | | | | | | |
|  | **P14174 - Macrophage migration inhibitory factor OS=Homo sapiens GN=MIF PE=1 SV=4 - [MIF\_HUMAN]** | | | | | | | | | | | | | | | | | | | | | | | | | | | | | | |  |
|  | | | | | | | | | | | | | | | | | | | | | | | | | | | | | | | | |
|  | High |  | PMFIVNTNVPR |  | CID |  |  |  | 56.25 |  |  |  | 1.00 |  | 1 |  | Mascot (2) |  | 2 |  | 644.34821 |  | 0.98 |  | 1831 |  | 1831 |  | Image |  | Peak List |  |
|  | | | | | | | | | | | | | | | | | | | | | | | | | | | | | | | | |
|  | **P14625 - Endoplasmin OS=Homo sapiens GN=HSP90B1 PE=1 SV=1 - [ENPL\_HUMAN]** | | | | | | | | | | | | | | | | | | | | | | | | | | | | | | |  |
|  | | | | | | | | | | | | | | | | | | | | | | | | | | | | | | | | |
|  | High |  | GTTITLVLKEEASDYLELDTIK |  | CID |  |  |  | 36.28 |  |  |  | 1.00 |  | 1 |  | Mascot (2) |  | 3 |  | 818.11066 |  | 4.26 |  | 2968 |  | 2968 |  | Image |  | Peak List |  |
|  | | | | | | | | | | | | | | | | | | | | | | | | | | | | | | | | |
|  | High |  | GTTITLVLKEEASDYLELDTIK |  | CID |  |  |  |  |  | 4.77 |  | 1.00 |  | 1 |  | SEQUEST (4) |  | 3 |  | 818.11127 |  | 5.00 |  | 2983 |  | 2983 |  | Image |  | Peak List |  |
|  | | | | | | | | | | | | | | | | | | | | | | | | | | | | | | | | |
|  | **P18859 - ATP synthase-coupling factor 6, mitochondrial OS=Homo sapiens GN=ATP5J PE=1 SV=1 - [ATP5J\_HUMAN]** | | | | | | | | | | | | | | | | | | | | | | | | | | | | | | |  |
|  | | | | | | | | | | | | | | | | | | | | | | | | | | | | | | | | |
|  | High |  | QTSGGPVDASSEYQQELER |  | CID |  |  |  | 58.85 |  |  |  | 1.00 |  | 1 |  | Mascot (2) |  | 2 |  | 1040.97852 |  | 3.79 |  | 1285 |  | 1285 |  | Image |  | Peak List |  |
|  | | | | | | | | | | | | | | | | | | | | | | | | | | | | | | | | |
|  | High |  | QTSGGPVDASSEYQQELER |  | CID |  |  |  |  |  | 2.86 |  | 1.00 |  | 1 |  | SEQUEST (4) |  | 2 |  | 1040.97852 |  | 3.79 |  | 1285 |  | 1285 |  | Image |  | Peak List |  |
|  | | | | | | | | | | | | | | | | | | | | | | | | | | | | | | | | |
|  | **P20962 - Parathymosin OS=Homo sapiens GN=PTMS PE=1 SV=2 - [PTMS\_HUMAN]** | | | | | | | | | | | | | | | | | | | | | | | | | | | | | | |  |
|  | | | | | | | | | | | | | | | | | | | | | | | | | | | | | | | | |
|  | High |  | SVEAAAELSAK |  | CID |  |  |  | 36.70 |  |  |  | 1.00 |  | 1 |  | Mascot (2) |  | 2 |  | 538.28607 |  | 1.70 |  | 909 |  | 909 |  | Image |  | Peak List |  |
|  | | | | | | | | | | | | | | | | | | | | | | | | | | | | | | | | |
|  | High |  | SVEAAAELSAK |  | CID |  |  |  |  |  | 2.81 |  | 1.00 |  | 1 |  | SEQUEST (4) |  | 2 |  | 538.28607 |  | 1.70 |  | 909 |  | 909 |  | Image |  | Peak List |  |
|  | | | | | | | | | | | | | | | | | | | | | | | | | | | | | | | | |
|  | **P21549 - Serine--pyruvate aminotransferase OS=Homo sapiens GN=AGXT PE=1 SV=1 - [SPYA\_HUMAN]** | | | | | | | | | | | | | | | | | | | | | | | | | | | | | | |  |
|  | | | | | | | | | | | | | | | | | | | | | | | | | | | | | | | | |
|  | High |  | DIVSYVIDHFDIEIMGGLGPSTGK |  | CID |  |  |  | 39.20 |  |  |  | 1.00 |  | 1 |  | Mascot (2) |  | 3 |  | 855.10352 |  | 8.19 |  | 4549 |  | 4549 |  | Image |  | Peak List |  |
|  | | | | | | | | | | | | | | | | | | | | | | | | | | | | | | | | |
|  | High |  | DIVSYVIDHFDIEIMGGLGPSTGK |  | CID |  |  |  |  |  | 3.31 |  | 1.00 |  | 1 |  | SEQUEST (4) |  | 3 |  | 855.10352 |  | 8.19 |  | 4549 |  | 4549 |  | Image |  | Peak List |  |
|  | | | | | | | | | | | | | | | | | | | | | | | | | | | | | | | | |
|  | **P21980 - Protein-glutamine gamma-glutamyltransferase 2 OS=Homo sapiens GN=TGM2 PE=1 SV=2 - [TGM2\_HUMAN]** | | | | | | | | | | | | | | | | | | | | | | | | | | | | | | |  |
|  | | | | | | | | | | | | | | | | | | | | | | | | | | | | | | | | |
|  | High |  | NIPWNFGQFEDGILDICLILLDVNPK |  | CID |  |  |  | 22.57 |  |  |  | 1.00 |  | 1 |  | Mascot (2) |  | 3 |  | 996.19232 |  | 8.03 |  | 6106 |  | 6106 |  | Image |  | Peak List |  |
|  | | | | | | | | | | | | | | | | | | | | | | | | | | | | | | | | |
|  | High |  | NIPWNFGQFEDGILDICLILLDVNPK |  | CID |  |  |  |  |  | 3.52 |  | 1.00 |  | 1 |  | SEQUEST (4) |  | 3 |  | 996.19232 |  | 8.03 |  | 6106 |  | 6106 |  | Image |  | Peak List |  |
|  | | | | | | | | | | | | | | | | | | | | | | | | | | | | | | | | |
|  | **P23141 - Liver carboxylesterase 1 OS=Homo sapiens GN=CES1 PE=1 SV=2 - [EST1\_HUMAN]** | | | | | | | | | | | | | | | | | | | | | | | | | | | | | | |  |
|  | | | | | | | | | | | | | | | | | | | | | | | | | | | | | | | | |
|  | High |  | DLFLDLIADVMFGVPSVIVAR |  | CID |  |  |  | 52.85 |  |  |  | 1.00 |  | 1 |  | Mascot (2) |  | 3 |  | 764.09375 |  | 6.55 |  | 6393 |  | 6393 |  | Image |  | Peak List |  |
|  | | | | | | | | | | | | | | | | | | | | | | | | | | | | | | | | |
|  | High |  | DLFLDLIADVMFGVPSVIVAR |  | CID |  |  |  |  |  | 4.57 |  | 1.00 |  | 1 |  | SEQUEST (4) |  | 3 |  | 764.09375 |  | 6.55 |  | 6393 |  | 6393 |  | Image |  | Peak List |  |
|  | | | | | | | | | | | | | | | | | | | | | | | | | | | | | | | | |
|  | **P30046 - D-dopachrome decarboxylase OS=Homo sapiens GN=DDT PE=1 SV=3 - [DOPD\_HUMAN]** | | | | | | | | | | | | | | | | | | | | | | | | | | | | | | |  |
|  | | | | | | | | | | | | | | | | | | | | | | | | | | | | | | | | |
|  | High |  | SHSAHFFEFLTK |  | CID |  |  |  | 26.06 |  |  |  | 1.00 |  | 1 |  | Mascot (2) |  | 3 |  | 484.24277 |  | 1.60 |  | 1437 |  | 1437 |  | Image |  | Peak List |  |
|  | | | | | | | | | | | | | | | | | | | | | | | | | | | | | | | | |
|  | High |  | SHSAHFFEFLTK |  | CID |  |  |  |  |  | 2.99 |  | 1.00 |  | 1 |  | SEQUEST (4) |  | 3 |  | 484.24277 |  | 1.60 |  | 1437 |  | 1437 |  | Image |  | Peak List |  |
|  | | | | | | | | | | | | | | | | | | | | | | | | | | | | | | | | |
|  | **P30084 - Enoyl-CoA hydratase, mitochondrial OS=Homo sapiens GN=ECHS1 PE=1 SV=4 - [ECHM\_HUMAN]** | | | | | | | | | | | | | | | | | | | | | | | | | | | | | | |  |
|  | | | | | | | | | | | | | | | | | | | | | | | | | | | | | | | | |
|  | High |  | ESVNAAFEMTLTEGSK |  | CID |  |  |  | 52.96 |  |  |  | 1.00 |  | 1 |  | Mascot (2) |  | 2 |  | 857.40619 |  | 2.93 |  | 2075 |  | 2075 |  | Image |  | Peak List |  |
|  | | | | | | | | | | | | | | | | | | | | | | | | | | | | | | | | |
|  | High |  | ESVNAAFEMTLTEGSK |  | CID |  |  |  |  |  | 3.52 |  | 1.00 |  | 1 |  | SEQUEST (4) |  | 2 |  | 857.40619 |  | 2.93 |  | 2075 |  | 2075 |  | Image |  | Peak List |  |
|  | | | | | | | | | | | | | | | | | | | | | | | | | | | | | | | | |
|  | **P35914 - Hydroxymethylglutaryl-CoA lyase, mitochondrial OS=Homo sapiens GN=HMGCL PE=1 SV=2 - [HMGCL\_HUMAN]** | | | | | | | | | | | | | | | | | | | | | | | | | | | | | | |  |
|  | | | | | | | | | | | | | | | | | | | | | | | | | | | | | | | | |
|  | High |  | EVVIFGAASELFTK |  | CID |  |  |  | 61.46 |  |  |  | 1.00 |  | 1 |  | Mascot (2) |  | 2 |  | 755.91357 |  | 3.06 |  | 3057 |  | 3057 |  | Image |  | Peak List |  |
|  | | | | | | | | | | | | | | | | | | | | | | | | | | | | | | | | |
|  | High |  | EVVIFGAASELFTK |  | CID |  |  |  |  |  | 4.38 |  | 1.00 |  | 1 |  | SEQUEST (4) |  | 2 |  | 755.91357 |  | 3.06 |  | 3057 |  | 3057 |  | Image |  | Peak List |  |
|  | | | | | | | | | | | | | | | | | | | | | | | | | | | | | | | | |
|  | **P61604 - 10 kDa heat shock protein, mitochondrial OS=Homo sapiens GN=HSPE1 PE=1 SV=2 - [CH10\_HUMAN]** | | | | | | | | | | | | | | | | | | | | | | | | | | | | | | |  |
|  | | | | | | | | | | | | | | | | | | | | | | | | | | | | | | | | |
|  | High |  | VLQATVVAVGSGSK |  | CID |  |  |  | 61.72 |  |  |  | 1.00 |  | 1 |  | Mascot (2) |  | 2 |  | 658.38300 |  | 0.50 |  | 1141 |  | 1141 |  | Image |  | Peak List |  |
|  | | | | | | | | | | | | | | | | | | | | | | | | | | | | | | | | |
|  | High |  | VLQATVVAVGSGSK |  | CID |  |  |  |  |  | 4.68 |  | 1.00 |  | 1 |  | SEQUEST (4) |  | 2 |  | 658.38324 |  | 0.87 |  | 1132 |  | 1132 |  | Image |  | Peak List |  |
|  | | | | | | | | | | | | | | | | | | | | | | | | | | | | | | | | |
|  | **Q06830 - Peroxiredoxin-1 OS=Homo sapiens GN=PRDX1 PE=1 SV=1 - [PRDX1\_HUMAN]** | | | | | | | | | | | | | | | | | | | | | | | | | | | | | | |  |
|  | | | | | | | | | | | | | | | | | | | | | | | | | | | | | | | | |
|  | High |  | LVQAFQFTDK |  | CID |  |  |  | 35.49 |  |  |  | 1.00 |  | 1 |  | Mascot (2) |  | 2 |  | 598.82080 |  | 2.74 |  | 1657 |  | 1657 |  | Image |  | Peak List |  |
|  | | | | | | | | | | | | | | | | | | | | | | | | | | | | | | | | |
|  | **Q08257 - Quinone oxidoreductase OS=Homo sapiens GN=CRYZ PE=1 SV=1 - [QOR\_HUMAN]** | | | | | | | | | | | | | | | | | | | | | | | | | | | | | | |  |
|  | | | | | | | | | | | | | | | | | | | | | | | | | | | | | | | | |
|  | High |  | GIDIIIEMLANVNLSK |  | CID |  |  |  | 42.55 |  |  |  | 1.00 |  | 1 |  | Mascot (2) |  | 2 |  | 871.99713 |  | 8.49 |  | 4461 |  | 4461 |  | Image |  | Peak List |  |
|  | | | | | | | | | | | | | | | | | | | | | | | | | | | | | | | | |
|  | High |  | GIDIIIEMLANVNLSK |  | CID |  |  |  |  |  | 3.46 |  | 1.00 |  | 1 |  | SEQUEST (4) |  | 2 |  | 871.99713 |  | 8.49 |  | 4461 |  | 4461 |  | Image |  | Peak List |  |
|  | | | | | | | | | | | | | | | | | | | | | | | | | | | | | | | | |
|  | **Q5VSP4 - Putative lipocalin 1-like protein 1 OS=Homo sapiens GN=LCN1P1 PE=5 SV=1 - [LC1L1\_HUMAN]** | | | | | | | | | | | | | | | | | | | | | | | | | | | | | | |  |
|  | | | | | | | | | | | | | | | | | | | | | | | | | | | | | | | | |
|  | High |  | GLSTESILIPR |  | CID |  |  |  | 33.46 |  |  |  | 1.00 |  | 1 |  | Mascot (2) |  | 2 |  | 593.34656 |  | 1.70 |  | 1948 |  | 1948 |  | Image |  | Peak List |  |
|  | | | | | | | | | | | | | | | | | | | | | | | | | | | | | | | | |
|  | High |  | GLSTESILIPR |  | CID |  |  |  |  |  | 2.63 |  | 1.00 |  | 1 |  | SEQUEST (4) |  | 2 |  | 593.34656 |  | 1.70 |  | 1948 |  | 1948 |  | Image |  | Peak List |  |
|  | | | | | | | | | | | | | | | | | | | | | | | | | | | | | | | | |
|  | **Q93088 - Betaine--homocysteine S-methyltransferase 1 OS=Homo sapiens GN=BHMT PE=1 SV=2 - [BHMT1\_HUMAN]** | | | | | | | | | | | | | | | | | | | | | | | | | | | | | | |  |
|  | | | | | | | | | | | | | | | | | | | | | | | | | | | | | | | | |
|  | High |  | ISGQEVNEAACDIAR |  | CID |  |  |  | 60.07 |  |  |  | 1.00 |  | 1 |  | Mascot (2) |  | 2 |  | 788.37695 |  | 2.16 |  | 1344 |  | 1344 |  | Image |  | Peak List |  |
|  | | | | | | | | | | | | | | | | | | | | | | | | | | | | | | | | |
|  | High |  | ISGQEVNEAACDIAR |  | CID |  |  |  |  |  | 2.92 |  | 1.00 |  | 1 |  | SEQUEST (4) |  | 2 |  | 788.37695 |  | 2.16 |  | 1344 |  | 1344 |  | Image |  | Peak List |  |
|  | | | | | | | | | | | | | | | | | | | | | | | | | | | | | | | | |
|  | **Q99497 - Protein DJ-1 OS=Homo sapiens GN=PARK7 PE=1 SV=2 - [PARK7\_HUMAN]** | | | | | | | | | | | | | | | | | | | | | | | | | | | | | | |  |
|  | | | | | | | | | | | | | | | | | | | | | | | | | | | | | | | | |
|  | High |  | GPGTSFEFALAIVEALNGK |  | CID |  |  |  | 65.02 |  |  |  | 1.00 |  | 1 |  | Mascot (2) |  | 2 |  | 961.01636 |  | 9.77 |  | 4302 |  | 4302 |  | Image |  | Peak List |  |
|  | | | | | | | | | | | | | | | | | | | | | | | | | | | | | | | | |
|  | High |  | GPGTSFEFALAIVEALNGK |  | CID |  |  |  |  |  | 5.31 |  | 1.00 |  | 1 |  | SEQUEST (4) |  | 2 |  | 961.01636 |  | 9.77 |  | 4302 |  | 4302 |  | Image |  | Peak List |  |
|  | | | | | | | | | | | | | | | | | | | | | | | | | | | | | | | | |
|  | **Q9BWD1 - Acetyl-CoA acetyltransferase, cytosolic OS=Homo sapiens GN=ACAT2 PE=1 SV=2 - [THIC\_HUMAN]** | | | | | | | | | | | | | | | | | | | | | | | | | | | | | | |  |
|  | | | | | | | | | | | | | | | | | | | | | | | | | | | | | | | | |
|  | High |  | AGWSLEDVDIFEINEAFAAVSAAIVK |  | CID |  |  |  | 27.60 |  |  |  | 1.00 |  | 1 |  | Mascot (2) |  | 3 |  | 922.48029 |  | 8.29 |  | 5057 |  | 5057 |  | Image |  | Peak List |  |
|  | | | | | | | | | | | | | | | | | | | | | | | | | | | | | | | | |
|  | High |  | AGWSLEDVDIFEINEAFAAVSAAIVK |  | CID |  |  |  |  |  | 4.15 |  | 1.00 |  | 1 |  | SEQUEST (4) |  | 3 |  | 922.48029 |  | 8.29 |  | 5057 |  | 5057 |  | Image |  | Peak List |  |
|  | | | | | | | | | | | | | | | | | | | | | | | | | | | | | | | | |
|  | **Q9BX68 - Histidine triad nucleotide-binding protein 2, mitochondrial OS=Homo sapiens GN=HINT2 PE=1 SV=1 - [HINT2\_HUMAN]** | | | | | | | | | | | | | | | | | | | | | | | | | | | | | | |  |
|  | | | | | | | | | | | | | | | | | | | | | | | | | | | | | | | | |
|  | High |  | ISQAEEEDQQLLGHLLLVAK |  | CID |  |  |  | 28.43 |  |  |  | 1.00 |  | 1 |  | Mascot (2) |  | 3 |  | 745.40875 |  | 3.99 |  | 2763 |  | 2763 |  | Image |  | Peak List |  |
|  | | | | | | | | | | | | | | | | | | | | | | | | | | | | | | | | |
|  | High |  | ISQAEEEDQQLLGHLLLVAK |  | CID |  |  |  |  |  | 3.23 |  | 1.00 |  | 1 |  | SEQUEST (4) |  | 3 |  | 745.40869 |  | 3.91 |  | 2834 |  | 2834 |  | Image |  | Peak List |  |
|  | | | | | | | | | | | | | | | | | | | | | | | | | | | | | | | | |
|  | **Q9H0W9 - Ester hydrolase C11orf54 OS=Homo sapiens GN=C11orf54 PE=1 SV=1 - [CK054\_HUMAN]** | | | | | | | | | | | | | | | | | | | | | | | | | | | | | | |  |
|  | | | | | | | | | | | | | | | | | | | | | | | | | | | | | | | | |
|  | High |  | IAEVGGVPYLLPLVNQK |  | CID |  |  |  | 20.40 |  |  |  | 1.00 |  | 1 |  | Mascot (2) |  | 2 |  | 905.53113 |  | 4.20 |  | 2955 |  | 2955 |  | Image |  | Peak List |  |
|  | | | | | | | | | | | | | | | | | | | | | | | | | | | | | | | | |
|  | High |  | IAEVGGVPYLLPLVNQK |  | CID |  |  |  |  |  | 2.96 |  | 1.00 |  | 1 |  | SEQUEST (4) |  | 2 |  | 905.53113 |  | 4.20 |  | 2955 |  | 2955 |  | Image |  | Peak List |  |
|  | | | | | | | | | | | | | | | | | | | | | | | | | | | | | | | | |
|  | **A2A2S5 - Ribosome binding protein 1 homolog 180kDa (Dog) (Fragment) OS=Homo sapiens GN=RRBP1 PE=4 SV=1 - [A2A2S5\_HUMAN]** | | | | | | | | | | | | | | | | | | | | | | | | | | | | | | |  |
|  | | | | | | | | | | | | | | | | | | | | | | | | | | | | | | | | |
|  | High |  | EVPMVVVPPVGAK |  | CID |  |  |  |  |  | 2.15 |  | 1.00 |  | 1 |  | SEQUEST (4) |  | 2 |  | 661.38257 |  | 2.23 |  | 1990 |  | 1990 |  | Image |  | Peak List |  |
|  | | | | | | | | | | | | | | | | | | | | | | | | | | | | | | | | |
|  | **C9JEY0 - 3-ketoacyl-CoA thiolase (Fragment) OS=Homo sapiens GN=HADHB PE=4 SV=1 - [C9JEY0\_HUMAN]** | | | | | | | | | | | | | | | | | | | | | | | | | | | | | | |  |
|  | | | | | | | | | | | | | | | | | | | | | | | | | | | | | | | | |
|  | High |  | TPAHTVTmACISANQAMTTGVG |  | CID |  | M8(Oxidation) |  |  |  | 1.36 |  | 1.00 |  | 1 |  | SEQUEST (4) |  | 2 |  | 1089.51196 |  | 8.15 |  | 5334 |  | 5334 |  | Image |  | Peak List |  |
|  | | | | | | | | | | | | | | | | | | | | | | | | | | | | | | | | |
|  | **E9PIW2 - Disks large homolog 2 OS=Homo sapiens GN=DLG2 PE=4 SV=1 - [E9PIW2\_HUMAN]** | | | | | | | | | | | | | | | | | | | | | | | | | | | | | | |  |
|  | | | | | | | | | | | | | | | | | | | | | | | | | | | | | | | | |
|  | High |  | EQSEQETSDPERGQEDLILSYEPVTR |  | CID |  |  |  |  |  | 0.56 |  | 1.00 |  | 1 |  | SEQUEST (4) |  | 2 |  | 1518.22058 |  | 8.62 |  | 6260 |  | 6260 |  | Image |  | Peak List |  |
|  | | | | | | | | | | | | | | | | | | | | | | | | | | | | | | | | |
|  | **E9PN89 - Heat shock cognate 71 kDa protein (Fragment) OS=Homo sapiens GN=HSPA8 PE=3 SV=1 - [E9PN89\_HUMAN]** | | | | | | | | | | | | | | | | | | | | | | | | | | | | | | |  |
|  | | | | | | | | | | | | | | | | | | | | | | | | | | | | | | | | |
|  | High |  | NVLIFDLGGGTFDVSILTIEDGIFEVK |  | CID |  |  |  |  |  | 2.02 |  | 1.00 |  | 1 |  | SEQUEST (4) |  | 3 |  | 971.19098 |  | 8.35 |  | 5075 |  | 5075 |  | Image |  | Peak List |  |
|  | | | | | | | | | | | | | | | | | | | | | | | | | | | | | | | | |
|  | **G3V3L6 - Methylenetetrahydrofolate dehydrogenase OS=Homo sapiens GN=MTHFD1 PE=3 SV=1 - [G3V3L6\_HUMAN]** | | | | | | | | | | | | | | | | | | | | | | | | | | | | | | |  |
|  | | | | | | | | | | | | | | | | | | | | | | | | | | | | | | | | |
|  | High |  | TAQFDISVASEIMAVLALTTSLEDMR |  | CID |  |  |  |  |  | 2.49 |  | 1.00 |  | 1 |  | SEQUEST (4) |  | 3 |  | 938.14874 |  | 7.40 |  | 5517 |  | 5517 |  | Image |  | Peak List |  |
|  | | | | | | | | | | | | | | | | | | | | | | | | | | | | | | | | |
|  | **H3BSW3 - Adenine phosphoribosyltransferase OS=Homo sapiens GN=APRT PE=4 SV=1 - [H3BSW3\_HUMAN]** | | | | | | | | | | | | | | | | | | | | | | | | | | | | | | |  |
|  | | | | | | | | | | | | | | | | | | | | | | | | | | | | | | | | |
|  | High |  | SFPDFPTPGVVFR |  | CID |  |  |  |  |  | 2.03 |  | 1.00 |  | 1 |  | SEQUEST (4) |  | 2 |  | 733.38086 |  | 4.74 |  | 2858 |  | 2858 |  | Image |  | Peak List |  |
|  | | | | | | | | | | | | | | | | | | | | | | | | | | | | | | | | |
|  | **O43598-2 - Isoform 2 of Deoxyribonucleoside 5'-monophosphate N-glycosidase OS=Homo sapiens GN=RCL - [RCL\_HUMAN]** | | | | | | | | | | | | | | | | | | | | | | | | | | | | | | |  |
|  | | | | | | | | | | | | | | | | | | | | | | | | | | | | | | | | |
|  | High |  | FGTVLTEHVAAAELGAR |  | CID |  |  |  |  |  | 1.95 |  | 1.00 |  | 1 |  | SEQUEST (4) |  | 3 |  | 581.31348 |  | 1.51 |  | 1651 |  | 1651 |  | Image |  | Peak List |  |
|  | | | | | | | | | | | | | | | | | | | | | | | | | | | | | | | | |
|  | **P09467 - Fructose-1,6-bisphosphatase 1 OS=Homo sapiens GN=FBP1 PE=1 SV=5 - [F16P1\_HUMAN]** | | | | | | | | | | | | | | | | | | | | | | | | | | | | | | |  |
|  | | | | | | | | | | | | | | | | | | | | | | | | | | | | | | | | |
|  | High |  | KLDVLSNDLVMNMLK |  | CID |  |  |  |  |  | 1.98 |  | 1.00 |  | 1 |  | SEQUEST (4) |  | 3 |  | 578.31763 |  | 2.68 |  | 2755 |  | 2755 |  | Image |  | Peak List |  |
|  | | | | | | | | | | | | | | | | | | | | | | | | | | | | | | | | |
|  | **Q6ZMS4-2 - Isoform 2 of Putative zinc finger protein 852 OS=Homo sapiens GN=ZNF852 - [ZN852\_HUMAN]** | | | | | | | | | | | | | | | | | | | | | | | | | | | | | | |  |
|  | | | | | | | | | | | | | | | | | | | | | | | | | | | | | | | | |
|  | High |  | AFNQISQLVEHERIHTGEKPFKCSECGK |  | CID |  |  |  |  |  | 2.27 |  | 1.00 |  | 1 |  | SEQUEST (4) |  | 5 |  | 643.91400 |  | -9.86 |  | 4056 |  | 4056 |  | Image |  | Peak List |  |
|  | | | | | | | | | | | | | | | | | | | | | | | | | | | | | | | | |
|  | **Q6ZMZ3-3 - Isoform 3 of Nesprin-3 OS=Homo sapiens GN=C14orf49 - [SYNE3\_HUMAN]** | | | | | | | | | | | | | | | | | | | | | | | | | | | | | | |  |
|  | | | | | | | | | | | | | | | | | | | | | | | | | | | | | | | | |
|  | High |  | DFPRGEESLETLEEQSAGVIRNTSPLGAEK |  | CID |  |  |  |  |  | 2.39 |  | 1.00 |  | 1 |  | SEQUEST (4) |  | 4 |  | 815.65942 |  | 2.45 |  | 4281 |  | 4281 |  | Image |  | Peak List |  |
|  | | | | | | | | | | | | | | | | | | | | | | | | | | | | | | | | |
|  | **Q9BSH5 - Haloacid dehalogenase-like hydrolase domain-containing protein 3 OS=Homo sapiens GN=HDHD3 PE=1 SV=1 - [HDHD3\_HUMAN]** | | | | | | | | | | | | | | | | | | | | | | | | | | | | | | |  |
|  | | | | | | | | | | | | | | | | | | | | | | | | | | | | | | | | |
|  | High |  | AHGLEVEPSALEQGFR |  | CID |  |  |  |  |  | 1.99 |  | 1.00 |  | 1 |  | SEQUEST (4) |  | 3 |  | 580.63031 |  | 2.97 |  | 1656 |  | 1656 |  | Image |  | Peak List |  |
|  | | | | | | | | | | | | | | | | | | | | | | | | | | | | | | | | |
|  | **Q9H1X5 - Prostaglandin reductase 1 OS=Homo sapiens GN=PTGR1 PE=2 SV=1 - [Q9H1X5\_HUMAN]** | | | | | | | | | | | | | | | | | | | | | | | | | | | | | | |  |
|  | | | | | | | | | | | | | | | | | | | | | | | | | | | | | | | | |
|  | High |  | NGEVLLEALFLTVDPYMR |  | CID |  |  |  |  |  | 2.65 |  | 1.00 |  | 1 |  | SEQUEST (4) |  | 3 |  | 694.03571 |  | 6.79 |  | 4765 |  | 4765 |  | Image |  | Peak List |  |
|  | | | | | | | | | | | | | | | | | | | | | | | | | | | | | | | | |

  
Top
  

### Search Summary

Workflow created with Discoverer version: 1.4.0.288 (DBVersion:79)
  
  
================================================================================
  
  
Search name: Methanol\_70\_1\_MC3
  
Search description: -
  
Search date: 06/25/2014 16:31:10
  
  
================================================================================
  
  
The pipeline tree:
  
------------------
  
  
    |-(0) Spectrum Files
  
        |-(1) Spectrum Selector
  
            |-(2) Mascot
  
                |-(3) Percolator
  
            |-(4) SEQUEST
  
                |-(3) Percolator
  
  
================================================================================
  
  
Search name: Methanol\_70\_1\_MC3
  
Search description: -
  
Search date: 06/25/2014 16:31:10
  
  
================================================================================
  
  
The pipeline tree:
  
------------------
  
  
    |-(0) Spectrum Files
  
        |-(1) Spectrum Selector
  
            |-(2) Mascot
  
                |-(3) Percolator
  
            |-(4) SEQUEST
  
                |-(3) Percolator
  
  
------------------------------------------------------------------------------
  
Processing node 0: Spectrum Files
  
------------------------------------------------------------------------------
  
  
Input Data:
  
-----------------------------
  
File Name(s): E:\Jos\Methanol\_70\_1.raw
  
  
------------------------------------------------------------------------------
  
Processing node 1: Spectrum Selector
  
------------------------------------------------------------------------------
  
  
1. General Settings:
  
-----------------------------
  
Precursor Selection: Use MS1 Precursor
  
Use New Precursor Reevaluation: True
  
  
2. Spectrum Properties Filter:
  
-----------------------------
  
Lower RT Limit: 0
  
Upper RT Limit: 0
  
First Scan: 0
  
Last Scan: 0
  
Lowest Charge State: 0
  
Highest Charge State: 0
  
Min. Precursor Mass: 350 Da
  
Max. Precursor Mass: 5000 Da
  
Total Intensity Threshold: 0
  
Minimum Peak Count: 1
  
  
3. Scan Event Filters:
  
-----------------------------
  
MS Order: Is MS2
  
Activation Type: Is CID
  
Min. Collision Energy: 0
  
Max. Collision Energy: 1000
  
Scan Type: Is Full
  
Ionization Source: Is Nanospray
  
  
4. Peak Filters:
  
-----------------------------
  
S/N Threshold (FT-only): 1.5
  
  
5. Replacements for Unrecognized Properties:
  
-----------------------------
  
Unrecognized Charge Replacements: Automatic
  
Unrecognized Mass Analyzer Replacements: ITMS
  
Unrecognized MS Order Replacements: MS2
  
Unrecognized Activation Type Replacements: CID
  
Unrecognized Polarity Replacements: +
  
  
6. Just for Testing:
  
-----------------------------
  
Precursor Clipping Range Before: 2.5 Da
  
Precursor Clipping Range After: 5.5 Da
  
  
------------------------------------------------------------------------------
  
Processing node 2: Mascot
  
------------------------------------------------------------------------------
  
  
1. Input Data:
  
-----------------------------
  
Protein Database: SwissProt
  
Enzyme Name: Trypsin
  
Maximum Missed Cleavage Sites: 3
  
Instrument: Default
  
Taxonomy: . . . . . . . . . . . . . . . . Homo sapiens (human)
  
  
1.1 Peptide Scoring Options:
  
-----------------------------
  
Peptide Cut Off Score: 10
  
Peptide Without Protein Cut Off Score: 5
  
  
1.2 Protein Scoring Options:
  
-----------------------------
  
Use MudPIT Scoring: Automatic
  
Protein Relevance Threshold: 20
  
Protein Relevance Factor: 1
  
  
2. Tolerances:
  
-----------------------------
  
Precursor Mass Tolerance: 10 ppm
  
Fragment Mass Tolerance: 0.8 Da
  
Use Average Precursor Mass: False
  
  
4. Dynamic Modifications:
  
-----------------------------
  
1. Dynamic Modification: Oxidation (M)
  
  
------------------------------------------------------------------------------
  
Processing node 3: Percolator
  
------------------------------------------------------------------------------
  
  
1. Input Data:
  
-----------------------------
  
Maximum Delta Cn: 0.05
  
  
2. Decoy Database Search:
  
-----------------------------
  
Target FDR (Strict): 0.01
  
Target FDR (Relaxed): 0.05
  
Validation based on: q-Value
  
  
------------------------------------------------------------------------------
  
Processing node 4: SEQUEST
  
------------------------------------------------------------------------------
  
  
1. Input Data:
  
-----------------------------
  
Protein Database: HUMAN\_swiss\_Jos.fasta
  
Enzyme Name: Trypsin (Full)
  
Maximum Missed Cleavage Sites: 3
  
  
1.1 Peptide Scoring Options:
  
-----------------------------
  
Maximum Peptides Considered: 500
  
Maximum Peptides Output: 10
  
Calculate Probability Scores: False
  
Absolute XCorr Threshold: 0.4
  
Fragment Ion Cutoff Percentage: 0.1
  
Peptide Without Protein XCorr Threshold: 1.5
  
  
1.2 Protein Scoring Options:
  
-----------------------------
  
Maximum Protein References Per Peptide: 100
  
Protein Relevance Threshold: 1.5
  
Peptide Relevance Factor: 0.4
  
  
2. Tolerances:
  
-----------------------------
  
Precursor Mass Tolerance: 10 ppm
  
Fragment Mass Tolerance: 0.8 Da
  
Use Average Precursor Mass: False
  
Use Average Fragment Masses: False
  
  
3. Ion Series:
  
-----------------------------
  
Use Neutral Loss a Ions: True
  
Use Neutral Loss b Ions: True
  
Use Neutral Loss y Ions: True
  
Weight of a Ions: 0
  
Weight of b Ions: 1
  
Weight of c Ions: 0
  
Weight of x Ions: 0
  
Weight of y Ions: 1
  
Weight of z Ions: 0
  
  
4. Dynamic Modifications:
  
-----------------------------
  
Max. Modifications Per Peptide: 4
  
1. Dynamic Modification: Oxidation / +15.995 Da (M)
  
  
================================================================================
  
  
Processing details:
  
  
06/25/2014 04:50 PM (4):SEQUEST: Total search time was 1 min 20 s.
  
06/25/2014 04:50 PM (3):Percolator: Performing percolator for SEQUEST (4) took 40.6 s.
  
06/25/2014 04:50 PM (4):SEQUEST: Search completed
  
06/25/2014 04:50 PM (4):SEQUEST: 5992 protein(s) + 5444 decoy proteins scored and inserted into result file in 3.6 s.
  
06/25/2014 04:50 PM (4):SEQUEST: 5992 protein(s) scored
  
06/25/2014 04:50 PM (4):SEQUEST: Search result finalization started.
  
06/25/2014 04:50 PM (3):Percolator: Start reading Percolator results
  
06/25/2014 04:50 PM (3):Percolator: Processing took 6.084 cpu seconds or 6 seconds wall time
  
06/25/2014 04:50 PM (3):Percolator: Calibrating statistics - calculating Posterior error probabilities (PEPs)
  
06/25/2014 04:50 PM (3):Percolator: PSMId score q-value posterior\_error\_prob peptide proteinIds
  
06/25/2014 04:50 PM (3):Percolator: New pi\_0 estimate on merged list gives 288 peptides over q=0.0100
  
06/25/2014 04:50 PM (3):Percolator: Calibrating statistics - calculating q values
  
06/25/2014 04:50 PM (3):Percolator: Selecting pi\_0=0.8991
  
06/25/2014 04:50 PM (3):Percolator: Tossing out "redundant" PSMs keeping only the best scoring PSM for each unique peptide.
  
06/25/2014 04:50 PM (3):Percolator: Merging results from 3 datasets
  
06/25/2014 04:50 PM (3):Percolator: Found 455 target PSMs scoring over 1.0000% FDR level on testset
  
06/25/2014 04:50 PM (3):Percolator: 0.6194 -0.0004 2.0870 0.0083 0.0085 0.0005 17.8543 0.1496 55.0714 -0.2615 -0.1368 0.0000 -2.0414 0.2510 1.8808 2.7884 -1.2736 -1.8707 0.0000 -1.4585 0.0632 -0.0222 -0.0328 -0.4595 0.3324 0.2688 0.9011 -4.8798 -0.0027 -6.7726 0.0014 -9.9534 0.0021 6.4470
  
06/25/2014 04:50 PM (3):Percolator: 0.437 -0.0805 0.5785 0.2331 0.2357 0.4305 0.2740 0.8551 0.5022 -0.7564 -1.1848 0.0000 -0.9898 0.1216 0.6965 0.7066 -0.1429 -1.9909 0.0000 -0.7658 0.7709 -0.3674 -0.5786 -1.3156 0.9621 0.4487 1.7924 -1.0166 -0.8731 -0.7308 0.2645 -0.9248 0.4273 -7.1741
  
06/25/2014 04:50 PM (3):Percolator: XCorr SpScore Delta Cn From Second PSM Binomial Score Isolation Interference [%] MH+ [Da] Delta Mass [Da] Delta Mass [ppm] Absolute Delta Mass [Da] Absolute Delta Mass [ppm] Peptide Length Is z=1 Is z=2 Is z=3 Is z=4 Is z=5 Is z>5 # Missed Cleavages Log Peptides Matched Log Total Intensity Fraction Matched Intensity [%] Fragment Coverage Series A, B, C [%] Fragment Coverage Series X, Y, Z [%] Log Matched Fragment Series Intensities A, B, C Log Matched Fragment Series Intensities X, Y, Z Longest Sequence Series A, B, C Longest Sequence Series X, Y, Z IQR Fragment Delta Mass [Da] IQR Fragment Delta Mass [ppm] Mean Fragment Delta Mass [Da] Mean Fragment Delta Mass [ppm] Mean Absolute Fragment Delta Mass [Da] Mean Absolute Fragment Delta Mass [ppm] m0
  
06/25/2014 04:50 PM (3):Percolator: # first line contains normalized weights, second line the raw weights
  
06/25/2014 04:50 PM (3):Percolator: Obtained weights (only showing weights of first cross validation set)
  
06/25/2014 04:50 PM (3):Percolator: Iteration 10 : After the iteration step, 572 target PSMs with q<0.01 were estimated by cross validation
  
06/25/2014 04:50 PM (3):Percolator: Iteration 9 : After the iteration step, 572 target PSMs with q<0.01 were estimated by cross validation
  
06/25/2014 04:50 PM (3):Percolator: Iteration 8 : After the iteration step, 571 target PSMs with q<0.01 were estimated by cross validation
  
06/25/2014 04:50 PM (3):Percolator: Iteration 7 : After the iteration step, 571 target PSMs with q<0.01 were estimated by cross validation
  
06/25/2014 04:50 PM (3):Percolator: Iteration 6 : After the iteration step, 570 target PSMs with q<0.01 were estimated by cross validation
  
06/25/2014 04:50 PM (3):Percolator: Iteration 5 : After the iteration step, 567 target PSMs with q<0.01 were estimated by cross validation
  
06/25/2014 04:50 PM (3):Percolator: Iteration 4 : After the iteration step, 563 target PSMs with q<0.01 were estimated by cross validation
  
06/25/2014 04:50 PM (3):Percolator: Iteration 3 : After the iteration step, 553 target PSMs with q<0.01 were estimated by cross validation
  
06/25/2014 04:50 PM (3):Percolator: Iteration 2 : After the iteration step, 533 target PSMs with q<0.01 were estimated by cross validation
  
06/25/2014 04:50 PM (3):Percolator: Iteration 1 : After the iteration step, 509 target PSMs with q<0.01 were estimated by cross validation
  
06/25/2014 04:50 PM (3):Percolator: ---Training with Cpos selected by cross validation, Cneg selected by cross validation, fdr=0.01
  
06/25/2014 04:50 PM (3):Percolator: Reading in data and feature calculation took 6.037 cpu seconds or 6 seconds wall time
  
06/25/2014 04:50 PM (3):Percolator: Estimating 308 over q=0.01 in initial direction
  
06/25/2014 04:50 PM (3):Percolator: Selected feature number 1 as initial search direction, could separate 177 positives in that direction
  
06/25/2014 04:50 PM (3):Percolator: Selected feature number 27 as initial search direction, could separate 238 positives in that direction
  
06/25/2014 04:50 PM (3):Percolator: Selected feature number 27 as initial search direction, could separate 216 positives in that direction
  
06/25/2014 04:50 PM (3):Percolator: selecting cneg by cross validation
  
06/25/2014 04:50 PM (3):Percolator: selecting cpos by cross validation
  
06/25/2014 04:50 PM (3):Percolator: Train/test set contains 3858 positives and 4059 negatives, size ratio=0.95048 and pi0=1
  
06/25/2014 04:50 PM (3):Percolator: 31e77142-29e9-402c-9ec2-8468a9513af0 e39a792e-622c-452d-b49b-59809cad79d0 Delta Cn From Second PSM Binomial Score b8754504-e95e-476b-b9a4-454d4bb53aeb 1d91a87b-953a-4887-9f22-f75a497a3538 Delta Mass [Da] Delta Mass [ppm] Absolute Delta Mass [Da] Absolute Delta Mass [ppm] Peptide Length Is z=1 Is z=2 Is z=3 Is z=4 Is z=5 Is z>5 041eb6d5-e486-44a0-9bc1-19e25811c686 Log Peptides Matched Log Total Intensity Fraction Matched Intensity [%] Fragment Coverage Series A, B, C [%] Fragment Coverage Series X, Y, Z [%] Log Matched Fragment Series Intensities A, B, C Log Matched Fragment Series Intensities X, Y, Z Longest Sequence Series A, B, C Longest Sequence Series X, Y, Z IQR Fragment Delta Mass [Da] IQR Fragment Delta Mass [ppm] Mean Fragment Delta Mass [Da] Mean Fragment Delta Mass [ppm] Mean Absolute Fragment Delta Mass [Da] Mean Absolute Fragment Delta Mass [ppm]
  
06/25/2014 04:50 PM (3):Percolator: Features:
  
06/25/2014 04:50 PM (3):Percolator: enzyme=Trypsin
  
06/25/2014 04:50 PM (3):Percolator: Hyperparameters fdr=0.01, Cpos=0, Cneg=0, maxNiter=10
  
06/25/2014 04:50 PM (3):Percolator: Started Wed Jun 25 16:50:36 2014
  
06/25/2014 04:50 PM (3):Percolator: C:\Program Files\Thermo\Discoverer 1.4\Tools\Percolator\percolator.exe -X C:\ProgramData\Thermo\Discoverer 1.4\Scratch\21614ab5-8172-4c35-919d-b42fc7c256ff\output.xml -Z C:\ProgramData\Thermo\Discoverer 1.4\Scratch\21614ab5-8172-4c35-919d-b42fc7c256ff\input.xml
  
06/25/2014 04:50 PM (3):Percolator: Issued command:
  
06/25/2014 04:50 PM (3):Percolator: Department of Genome Sciences at the University of Washington.
  
06/25/2014 04:50 PM (3):Percolator: Written by Lukas K+�ll (lukall@u.washington.edu) in the
  
06/25/2014 04:50 PM (3):Percolator: Copyright (c) 2006-9 University of Washington. All rights reserved.
  
06/25/2014 04:50 PM (3):Percolator: Percolator version 2.04, Build Date Feb 1 2012 03:35:34
  
06/25/2014 04:50 PM (3):Percolator: Starting Percolator
  
06/25/2014 04:50 PM (3):Percolator: The input file contains 3858 peptides, 4059 decoy peptides and 33 features.
  
06/25/2014 04:50 PM (3):Percolator: Creating input file for SEQUEST (4) took 26.6 s.
  
06/25/2014 04:50 PM (3):Percolator: Start calculating features for peptides of SEQUEST (4)
  
06/25/2014 04:50 PM (2):Mascot: Total search time was 1 min 42 s.
  
06/25/2014 04:50 PM (3):Percolator: Performing percolator for Mascot (2) took 38.8 s.
  
06/25/2014 04:50 PM (2):Mascot: Search completed
  
06/25/2014 04:50 PM (2):Mascot: 147 protein(s) + 25 decoy proteins scored and inserted into result file in 0.6 s.
  
06/25/2014 04:50 PM (2):Mascot: 147 protein(s) scored
  
06/25/2014 04:50 PM (2):Mascot: Search result finalization started.
  
06/25/2014 04:50 PM (3):Percolator: Start reading Percolator results
  
06/25/2014 04:50 PM (3):Percolator: Processing took 8.627 cpu seconds or 9 seconds wall time
  
06/25/2014 04:50 PM (3):Percolator: Calibrating statistics - calculating Posterior error probabilities (PEPs)
  
06/25/2014 04:50 PM (3):Percolator: PSMId score q-value posterior\_error\_prob peptide proteinIds
  
06/25/2014 04:50 PM (3):Percolator: New pi\_0 estimate on merged list gives 287 peptides over q=0.0100
  
06/25/2014 04:50 PM (3):Percolator: Calibrating statistics - calculating q values
  
06/25/2014 04:50 PM (3):Percolator: Selecting pi\_0=0.8555
  
06/25/2014 04:50 PM (3):Percolator: Tossing out "redundant" PSMs keeping only the best scoring PSM for each unique peptide.
  
06/25/2014 04:49 PM (3):Percolator: Merging results from 3 datasets
  
06/25/2014 04:49 PM (3):Percolator: Found 436 target PSMs scoring over 1.0000% FDR level on testset
  
06/25/2014 04:49 PM (3):Percolator: 0.0797 1.9754 0.0009 -0.0187 -0.0021 74.9282 -0.1213 218.0007 -0.0732 0.0864 0.0000 4.8539 3.0551 -4.5020 -13.3275 -15.3606 -3.0574 0.0000 -4.4814 0.0401 -0.0127 0.0379 0.5654 1.4797 0.4515 0.8459 1.8520 -0.0094 -9.9586 0.0027 9.8455 -0.0013 -3.4260
  
06/25/2014 04:49 PM (3):Percolator: 0.829 0.6081 0.0273 -0.5526 -2.3082 1.2509 -0.6760 2.2014 -0.2111 0.8680 0.0000 2.2263 1.5139 -1.7277 -3.4404 -1.8390 -3.2747 0.0000 -3.5398 0.6546 -0.2440 0.7917 3.4726 4.6715 0.8279 1.8631 0.4418 -3.3408 -1.3368 0.4899 1.1090 -0.2826 -10.5602
  
06/25/2014 04:49 PM (3):Percolator: IonScore Delta Cn From Second PSM Binomial Score Isolation Interference [%] MH+ [Da] Delta Mass [Da] Delta Mass [ppm] Absolute Delta Mass [Da] Absolute Delta Mass [ppm] Peptide Length Is z=1 Is z=2 Is z=3 Is z=4 Is z=5 Is z>5 # Missed Cleavages Log Peptides Matched Log Total Intensity Fraction Matched Intensity [%] Fragment Coverage Series A, B, C [%] Fragment Coverage Series X, Y, Z [%] Log Matched Fragment Series Intensities A, B, C Log Matched Fragment Series Intensities X, Y, Z Longest Sequence Series A, B, C Longest Sequence Series X, Y, Z IQR Fragment Delta Mass [Da] IQR Fragment Delta Mass [ppm] Mean Fragment Delta Mass [Da] Mean Fragment Delta Mass [ppm] Mean Absolute Fragment Delta Mass [Da] Mean Absolute Fragment Delta Mass [ppm] m0
  
06/25/2014 04:49 PM (3):Percolator: # first line contains normalized weights, second line the raw weights
  
06/25/2014 04:49 PM (3):Percolator: Obtained weights (only showing weights of first cross validation set)
  
06/25/2014 04:49 PM (3):Percolator: Iteration 10 : After the iteration step, 559 target PSMs with q<0.01 were estimated by cross validation
  
06/25/2014 04:49 PM (3):Percolator: Iteration 9 : After the iteration step, 558 target PSMs with q<0.01 were estimated by cross validation
  
06/25/2014 04:49 PM (3):Percolator: Iteration 8 : After the iteration step, 558 target PSMs with q<0.01 were estimated by cross validation
  
06/25/2014 04:49 PM (3):Percolator: Iteration 7 : After the iteration step, 557 target PSMs with q<0.01 were estimated by cross validation
  
06/25/2014 04:49 PM (3):Percolator: Iteration 6 : After the iteration step, 554 target PSMs with q<0.01 were estimated by cross validation
  
06/25/2014 04:49 PM (3):Percolator: Iteration 5 : After the iteration step, 551 target PSMs with q<0.01 were estimated by cross validation
  
06/25/2014 04:49 PM (3):Percolator: Iteration 4 : After the iteration step, 542 target PSMs with q<0.01 were estimated by cross validation
  
06/25/2014 04:49 PM (3):Percolator: Iteration 3 : After the iteration step, 538 target PSMs with q<0.01 were estimated by cross validation
  
06/25/2014 04:49 PM (3):Percolator: Iteration 2 : After the iteration step, 523 target PSMs with q<0.01 were estimated by cross validation
  
06/25/2014 04:49 PM (3):Percolator: Iteration 1 : After the iteration step, 493 target PSMs with q<0.01 were estimated by cross validation
  
06/25/2014 04:49 PM (3):Percolator: ---Training with Cpos selected by cross validation, Cneg selected by cross validation, fdr=0.01
  
06/25/2014 04:49 PM (3):Percolator: Reading in data and feature calculation took 4.789 cpu seconds or 5 seconds wall time
  
06/25/2014 04:49 PM (3):Percolator: Estimating 315 over q=0.01 in initial direction
  
06/25/2014 04:49 PM (3):Percolator: Selected feature number 1 as initial search direction, could separate 236 positives in that direction
  
06/25/2014 04:49 PM (3):Percolator: Selected feature number 1 as initial search direction, could separate 191 positives in that direction
  
06/25/2014 04:49 PM (3):Percolator: Selected feature number 1 as initial search direction, could separate 187 positives in that direction
  
06/25/2014 04:49 PM (3):Percolator: selecting cneg by cross validation
  
06/25/2014 04:49 PM (3):Percolator: selecting cpos by cross validation
  
06/25/2014 04:49 PM (3):Percolator: Train/test set contains 3249 positives and 3214 negatives, size ratio=1.01089 and pi0=1
  
06/25/2014 04:49 PM (3):Percolator: e6e22773-e9a6-4a26-9694-1ca77a797099 Delta Cn From Second PSM Binomial Score b8754504-e95e-476b-b9a4-454d4bb53aeb 1d91a87b-953a-4887-9f22-f75a497a3538 Delta Mass [Da] Delta Mass [ppm] Absolute Delta Mass [Da] Absolute Delta Mass [ppm] Peptide Length Is z=1 Is z=2 Is z=3 Is z=4 Is z=5 Is z>5 041eb6d5-e486-44a0-9bc1-19e25811c686 Log Peptides Matched Log Total Intensity Fraction Matched Intensity [%] Fragment Coverage Series A, B, C [%] Fragment Coverage Series X, Y, Z [%] Log Matched Fragment Series Intensities A, B, C Log Matched Fragment Series Intensities X, Y, Z Longest Sequence Series A, B, C Longest Sequence Series X, Y, Z IQR Fragment Delta Mass [Da] IQR Fragment Delta Mass [ppm] Mean Fragment Delta Mass [Da] Mean Fragment Delta Mass [ppm] Mean Absolute Fragment Delta Mass [Da] Mean Absolute Fragment Delta Mass [ppm]
  
06/25/2014 04:49 PM (3):Percolator: Features:
  
06/25/2014 04:49 PM (3):Percolator: enzyme=Trypsin
  
06/25/2014 04:49 PM (3):Percolator: Hyperparameters fdr=0.01, Cpos=0, Cneg=0, maxNiter=10
  
06/25/2014 04:49 PM (3):Percolator: Started Wed Jun 25 16:49:52 2014
  
06/25/2014 04:49 PM (3):Percolator: C:\Program Files\Thermo\Discoverer 1.4\Tools\Percolator\percolator.exe -X C:\ProgramData\Thermo\Discoverer 1.4\Scratch\ddaf81f5-7329-4554-bdbd-2631349ba792\output.xml -Z C:\ProgramData\Thermo\Discoverer 1.4\Scratch\ddaf81f5-7329-4554-bdbd-2631349ba792\input.xml
  
06/25/2014 04:49 PM (3):Percolator: Issued command:
  
06/25/2014 04:49 PM (3):Percolator: Department of Genome Sciences at the University of Washington.
  
06/25/2014 04:49 PM (3):Percolator: Written by Lukas K+�ll (lukall@u.washington.edu) in the
  
06/25/2014 04:49 PM (3):Percolator: Copyright (c) 2006-9 University of Washington. All rights reserved.
  
06/25/2014 04:49 PM (3):Percolator: Percolator version 2.04, Build Date Feb 1 2012 03:35:34
  
06/25/2014 04:49 PM (3):Percolator: Starting Percolator
  
06/25/2014 04:49 PM (3):Percolator: The input file contains 3249 peptides, 3214 decoy peptides and 32 features.
  
06/25/2014 04:49 PM (3):Percolator: Creating input file for Mascot (2) took 23.9 s.
  
06/25/2014 04:49 PM (3):Percolator: Start calculating features for peptides of Mascot (2)
  
06/25/2014 04:49 PM (2):Mascot: Used mascot server http://fenn.bham.ac.uk/mascot/ with Mascot version 2.4.1
  
06/25/2014 04:49 PM (2):Mascot: Sending 2702 peptide hits (12052 peptides) to result file
  
06/25/2014 04:49 PM (2):Mascot: Sending 2599 decoy peptide hits (11399 peptides) to result file
  
06/25/2014 04:49 PM (2):Mascot: Reading decoy results
  
06/25/2014 04:49 PM (2):Mascot: Start translating results
  
06/25/2014 04:49 PM (2):Mascot: Start mapping modifications
  
06/25/2014 04:49 PM (2):Mascot: Received 166 proteins from Mascot server
  
06/25/2014 04:49 PM (2):Mascot: Start mapping 166 proteins
  
06/25/2014 04:49 PM (2):Mascot: Start parsing results
  
06/25/2014 04:49 PM (2):Mascot: Received Mascot result file (filename=../data/20140625/F004278.dat)
  
06/25/2014 04:49 PM (2):Mascot: Mascot Server completed
  
06/25/2014 04:48 PM (2):Mascot: Mascot result on server (filename=../data/20140625/F004278.dat)
  
06/25/2014 04:47 PM (2):Mascot: Start searching 4318 spectra
  
06/25/2014 04:47 PM (4):SEQUEST: Sending 318 decoy peptide hits (1421 peptides) to result file
  
06/25/2014 04:47 PM (4):SEQUEST: Starting SEQUEST decoy search
  
06/25/2014 04:47 PM (4):SEQUEST: Sending 318 peptide hits (1435 peptides) to result file
  
06/25/2014 04:47 PM (4):SEQUEST: Starting SEQUEST (search spectra 4000 - 4318)
  
06/25/2014 04:47 PM (4):SEQUEST: Sending 1000 decoy peptide hits (3060 peptides) to result file
  
06/25/2014 04:47 PM (4):SEQUEST: Starting SEQUEST decoy search
  
06/25/2014 04:47 PM (4):SEQUEST: Sending 1000 peptide hits (3095 peptides) to result file
  
06/25/2014 04:47 PM (4):SEQUEST: Starting SEQUEST (search spectra 3000 - 4000)
  
06/25/2014 04:47 PM (4):SEQUEST: Sending 1000 decoy peptide hits (5538 peptides) to result file
  
06/25/2014 04:47 PM (4):SEQUEST: Starting SEQUEST decoy search
  
06/25/2014 04:47 PM (4):SEQUEST: Sending 1000 peptide hits (5499 peptides) to result file
  
06/25/2014 04:47 PM (4):SEQUEST: Starting SEQUEST (search spectra 2000 - 3000)
  
06/25/2014 04:47 PM (4):SEQUEST: Sending 1000 decoy peptide hits (8694 peptides) to result file
  
06/25/2014 04:46 PM (4):SEQUEST: Starting SEQUEST decoy search
  
06/25/2014 04:46 PM (4):SEQUEST: Sending 1000 peptide hits (8717 peptides) to result file
  
06/25/2014 04:46 PM (4):SEQUEST: Starting SEQUEST (search spectra 1000 - 2000)
  
06/25/2014 04:46 PM (4):SEQUEST: Sending 1000 decoy peptide hits (9345 peptides) to result file
  
06/25/2014 04:46 PM (4):SEQUEST: Starting SEQUEST decoy search
  
06/25/2014 04:46 PM (4):SEQUEST: Sending 1000 peptide hits (9347 peptides) to result file
  
06/25/2014 04:46 PM (4):SEQUEST: Starting SEQUEST (search spectra 0 - 1000)
  
06/25/2014 04:46 PM (4):SEQUEST: There is already an adequate decoy FASTA index.
  
06/25/2014 04:46 PM (4):SEQUEST: Looking for existing decoy FASTA index.
  
06/25/2014 04:46 PM (4):SEQUEST: There is already an adequate target FASTA index.
  
06/25/2014 04:46 PM (4):SEQUEST: Looking for existing target FASTA index.
  
06/25/2014 04:46 PM (2):Mascot: Use mascot server http://fenn.bham.ac.uk/mascot/ with Mascot version 2.4.1
  
06/25/2014 04:45 PM (1):Spectrum Selector: Reading from File 1 of 1:E:\Jos\Methanol\_70\_1.raw (7744 spectra total)
  
  
  
Top
