# Supplementary material for: Top-Down and Bottom-Up Identification of Proteins by Liquid Extraction Surface Analysis Mass Spectrometry of Healthy and Diseased Human Liver Tissue
Source: J Am Soc Mass Spectrom. 2014 Sep 3;25(11):1953–61. doi: 10.1007/s13361-014-0967-z (PMC4197381; doi:10.1007/s13361-014-0967-z)
Supplement: Supplementary file 9 — (ZIP 1716 kb) [file 13361_2014_967_MOESM9_ESM.zip › index.html]

Annotated spectra


## Annotated spectra of Methanol\_70\_2\_MC3.msf

go to Peptides  
go to Search Summary  

### Peptides

|  |  |  |  |  |  |  |  |  |  |  |  |  |  |  |  |  |  |  |  |  |  |  |  |  |  |  |  |  |  |  |  |  |
| --- | --- | --- | --- | --- | --- | --- | --- | --- | --- | --- | --- | --- | --- | --- | --- | --- | --- | --- | --- | --- | --- | --- | --- | --- | --- | --- | --- | --- | --- | --- | --- | --- |
|  | | | | | | | | | | | | | | | | | | | | | | | | | | | | | | | | |
| Confidence Sequence Activation Type Modifications IonScore XCorr ΔScore Rank Search Engine Charge Precursor m/z [Da] ΔM [ppm] First Scan Last Scan Annotated Spectrum Peak List  | | | | | | | | | | | | | | | | | | | | | | | | | | | | | | | | |
|  | | | | | | | | | | | | | | | | | | | | | | | | | | | | | | | | |
|  | **O43708 - Maleylacetoacetate isomerase OS=Homo sapiens GN=GSTZ1 PE=1 SV=3 - [MAAI\_HUMAN]** | | | | | | | | | | | | | | | | | | | | | | | | | | | | | | |  |
|  | | | | | | | | | | | | | | | | | | | | | | | | | | | | | | | | |
|  | High |  | DFQALNPMK |  | CID |  |  |  | 22.88 |  |  |  | 1.00 |  | 1 |  | Mascot (2) |  | 2 |  | 532.26837 |  | 5.00 |  | 1753 |  | 1753 |  | Image |  | Peak List |  |
|  | | | | | | | | | | | | | | | | | | | | | | | | | | | | | | | | |
|  | **P00338 - L-lactate dehydrogenase A chain OS=Homo sapiens GN=LDHA PE=1 SV=2 - [LDHA\_HUMAN]** | | | | | | | | | | | | | | | | | | | | | | | | | | | | | | |  |
|  | | | | | | | | | | | | | | | | | | | | | | | | | | | | | | | | |
|  | High |  | ITVVGVGAVGMACAISILMK |  | CID |  |  |  | 27.87 |  |  |  | 1.00 |  | 1 |  | Mascot (2) |  | 3 |  | 645.03259 |  | 7.65 |  | 4434 |  | 4434 |  | Image |  | Peak List |  |
|  | | | | | | | | | | | | | | | | | | | | | | | | | | | | | | | | |
|  | High |  | ITVVGVGAVGMACAISILMK |  | CID |  |  |  |  |  | 3.02 |  | 1.00 |  | 1 |  | SEQUEST (4) |  | 3 |  | 645.03259 |  | 7.65 |  | 4434 |  | 4434 |  | Image |  | Peak List |  |
|  | | | | | | | | | | | | | | | | | | | | | | | | | | | | | | | | |
|  | **P00441 - Superoxide dismutase [Cu-Zn] OS=Homo sapiens GN=SOD1 PE=1 SV=2 - [SODC\_HUMAN]** | | | | | | | | | | | | | | | | | | | | | | | | | | | | | | |  |
|  | | | | | | | | | | | | | | | | | | | | | | | | | | | | | | | | |
|  | High |  | DGVADVSIEDSVISLSGDHCIIGR |  | CID |  |  |  | 31.92 |  |  |  | 1.00 |  | 1 |  | Mascot (2) |  | 3 |  | 819.73944 |  | 4.50 |  | 3052 |  | 3052 |  | Image |  | Peak List |  |
|  | | | | | | | | | | | | | | | | | | | | | | | | | | | | | | | | |
|  | **P00738 - Haptoglobin OS=Homo sapiens GN=HP PE=1 SV=1 - [HPT\_HUMAN]** | | | | | | | | | | | | | | | | | | | | | | | | | | | | | | |  |
|  | | | | | | | | | | | | | | | | | | | | | | | | | | | | | | | | |
|  | High |  | VGYVSGWGR |  | CID |  |  |  | 34.50 |  |  |  | 1.00 |  | 1 |  | Mascot (2) |  | 2 |  | 490.75244 |  | 2.78 |  | 1199 |  | 1199 |  | Image |  | Peak List |  |
|  | | | | | | | | | | | | | | | | | | | | | | | | | | | | | | | | |
|  | High |  | VGYVSGWGR |  | CID |  |  |  |  |  | 2.37 |  | 1.00 |  | 1 |  | SEQUEST (4) |  | 2 |  | 490.75244 |  | 2.78 |  | 1199 |  | 1199 |  | Image |  | Peak List |  |
|  | | | | | | | | | | | | | | | | | | | | | | | | | | | | | | | | |
|  | **P01009 - Alpha-1-antitrypsin OS=Homo sapiens GN=SERPINA1 PE=1 SV=3 - [A1AT\_HUMAN]** | | | | | | | | | | | | | | | | | | | | | | | | | | | | | | |  |
|  | | | | | | | | | | | | | | | | | | | | | | | | | | | | | | | | |
|  | High |  | DTEEEDFHVDQVTTVK |  | CID |  |  |  | 63.94 |  |  |  | 1.00 |  | 1 |  | Mascot (2) |  | 2 |  | 946.43536 |  | 4.11 |  | 1287 |  | 1287 |  | Image |  | Peak List |  |
|  | | | | | | | | | | | | | | | | | | | | | | | | | | | | | | | | |
|  | High |  | DTEEEDFHVDQVTTVK |  | CID |  |  |  |  |  | 5.01 |  | 1.00 |  | 1 |  | SEQUEST (4) |  | 2 |  | 946.43536 |  | 4.11 |  | 1287 |  | 1287 |  | Image |  | Peak List |  |
|  | | | | | | | | | | | | | | | | | | | | | | | | | | | | | | | | |
|  | **P01859 - Ig gamma-2 chain C region OS=Homo sapiens GN=IGHG2 PE=1 SV=2 - [IGHG2\_HUMAN]** | | | | | | | | | | | | | | | | | | | | | | | | | | | | | | |  |
|  | | | | | | | | | | | | | | | | | | | | | | | | | | | | | | | | |
|  | High |  | DTLMISR |  | CID |  |  |  | 36.15 |  |  |  | 1.00 |  | 1 |  | Mascot (2) |  | 2 |  | 418.22180 |  | 2.47 |  | 1112 |  | 1112 |  | Image |  | Peak List |  |
|  | | | | | | | | | | | | | | | | | | | | | | | | | | | | | | | | |
|  | **P02656 - Apolipoprotein C-III OS=Homo sapiens GN=APOC3 PE=1 SV=1 - [APOC3\_HUMAN]** | | | | | | | | | | | | | | | | | | | | | | | | | | | | | | |  |
|  | | | | | | | | | | | | | | | | | | | | | | | | | | | | | | | | |
|  | High |  | DALSSVQESQVAQQAR |  | CID |  |  |  | 48.56 |  |  |  | 1.00 |  | 1 |  | Mascot (2) |  | 2 |  | 858.93170 |  | 2.87 |  | 1204 |  | 1204 |  | Image |  | Peak List |  |
|  | | | | | | | | | | | | | | | | | | | | | | | | | | | | | | | | |
|  | High |  | DALSSVQESQVAQQAR |  | CID |  |  |  |  |  | 3.74 |  | 1.00 |  | 1 |  | SEQUEST (4) |  | 2 |  | 858.93170 |  | 2.87 |  | 1204 |  | 1204 |  | Image |  | Peak List |  |
|  | | | | | | | | | | | | | | | | | | | | | | | | | | | | | | | | |
|  | **P02788 - Lactotransferrin OS=Homo sapiens GN=LTF PE=1 SV=6 - [TRFL\_HUMAN]** | | | | | | | | | | | | | | | | | | | | | | | | | | | | | | |  |
|  | | | | | | | | | | | | | | | | | | | | | | | | | | | | | | | | |
|  | High |  | DGAGDVAFIR |  | CID |  |  |  | 37.35 |  |  |  | 1.00 |  | 1 |  | Mascot (2) |  | 2 |  | 510.75964 |  | 1.04 |  | 1579 |  | 1579 |  | Image |  | Peak List |  |
|  | | | | | | | | | | | | | | | | | | | | | | | | | | | | | | | | |
|  | High |  | DGAGDVAFIR |  | CID |  |  |  |  |  | 2.91 |  | 1.00 |  | 1 |  | SEQUEST (4) |  | 2 |  | 510.75964 |  | 1.04 |  | 1579 |  | 1579 |  | Image |  | Peak List |  |
|  | | | | | | | | | | | | | | | | | | | | | | | | | | | | | | | | |
|  | **P07900 - Heat shock protein HSP 90-alpha OS=Homo sapiens GN=HSP90AA1 PE=1 SV=5 - [HS90A\_HUMAN]** | | | | | | | | | | | | | | | | | | | | | | | | | | | | | | |  |
|  | | | | | | | | | | | | | | | | | | | | | | | | | | | | | | | | |
|  | High |  | DLVILLYETALLSSGFSLEDPQTHANR |  | CID |  |  |  | 42.22 |  |  |  | 1.00 |  | 1 |  | Mascot (2) |  | 3 |  | 1001.52875 |  | 8.20 |  | 4582 |  | 4582 |  | Image |  | Peak List |  |
|  | | | | | | | | | | | | | | | | | | | | | | | | | | | | | | | | |
|  | High |  | DLVILLYETALLSSGFSLEDPQTHANR |  | CID |  |  |  |  |  | 1.98 |  | 1.00 |  | 1 |  | SEQUEST (4) |  | 3 |  | 1001.52875 |  | 8.20 |  | 4582 |  | 4582 |  | Image |  | Peak List |  |
|  | | | | | | | | | | | | | | | | | | | | | | | | | | | | | | | | |
|  | **P0CG48 - Polyubiquitin-C OS=Homo sapiens GN=UBC PE=1 SV=3 - [UBC\_HUMAN]** | | | | | | | | | | | | | | | | | | | | | | | | | | | | | | |  |
|  | | | | | | | | | | | | | | | | | | | | | | | | | | | | | | | | |
|  | High |  | TITLEVEPSDTIENVK |  | CID |  |  |  | 50.04 |  |  |  | 1.00 |  | 1 |  | Mascot (2) |  | 2 |  | 894.46906 |  | 1.93 |  | 1909 |  | 1909 |  | Image |  | Peak List |  |
|  | | | | | | | | | | | | | | | | | | | | | | | | | | | | | | | | |
|  | High |  | TITLEVEPSDTIENVK |  | CID |  |  |  |  |  | 4.50 |  | 1.00 |  | 1 |  | SEQUEST (4) |  | 2 |  | 894.46906 |  | 1.93 |  | 1909 |  | 1909 |  | Image |  | Peak List |  |
|  | | | | | | | | | | | | | | | | | | | | | | | | | | | | | | | | |
|  | **P10599 - Thioredoxin OS=Homo sapiens GN=TXN PE=1 SV=3 - [THIO\_HUMAN]** | | | | | | | | | | | | | | | | | | | | | | | | | | | | | | |  |
|  | | | | | | | | | | | | | | | | | | | | | | | | | | | | | | | | |
|  | High |  | EKLEATINELV |  | CID |  |  |  | 38.58 |  |  |  | 1.00 |  | 1 |  | Mascot (2) |  | 2 |  | 629.84924 |  | 1.78 |  | 2135 |  | 2135 |  | Image |  | Peak List |  |
|  | | | | | | | | | | | | | | | | | | | | | | | | | | | | | | | | |
|  | High |  | EKLEATINELV |  | CID |  |  |  |  |  | 3.37 |  | 1.00 |  | 1 |  | SEQUEST (4) |  | 2 |  | 629.84924 |  | 1.78 |  | 2135 |  | 2135 |  | Image |  | Peak List |  |
|  | | | | | | | | | | | | | | | | | | | | | | | | | | | | | | | | |
|  | **P14174 - Macrophage migration inhibitory factor OS=Homo sapiens GN=MIF PE=1 SV=4 - [MIF\_HUMAN]** | | | | | | | | | | | | | | | | | | | | | | | | | | | | | | |  |
|  | | | | | | | | | | | | | | | | | | | | | | | | | | | | | | | | |
|  | High |  | PMFIVNTNVPR |  | CID |  |  |  | 56.25 |  |  |  | 1.00 |  | 1 |  | Mascot (2) |  | 2 |  | 644.34766 |  | 0.13 |  | 1838 |  | 1838 |  | Image |  | Peak List |  |
|  | | | | | | | | | | | | | | | | | | | | | | | | | | | | | | | | |
|  | **P14625 - Endoplasmin OS=Homo sapiens GN=HSP90B1 PE=1 SV=1 - [ENPL\_HUMAN]** | | | | | | | | | | | | | | | | | | | | | | | | | | | | | | |  |
|  | | | | | | | | | | | | | | | | | | | | | | | | | | | | | | | | |
|  | High |  | GTTITLVLKEEASDYLELDTIK |  | CID |  |  |  | 21.36 |  |  |  | 1.00 |  | 1 |  | Mascot (2) |  | 3 |  | 818.11078 |  | 4.40 |  | 2952 |  | 2952 |  | Image |  | Peak List |  |
|  | | | | | | | | | | | | | | | | | | | | | | | | | | | | | | | | |
|  | High |  | GTTITLVLKEEASDYLELDTIK |  | CID |  |  |  |  |  | 4.08 |  | 1.00 |  | 1 |  | SEQUEST (4) |  | 3 |  | 818.11053 |  | 4.11 |  | 2956 |  | 2956 |  | Image |  | Peak List |  |
|  | | | | | | | | | | | | | | | | | | | | | | | | | | | | | | | | |
|  | **P18859 - ATP synthase-coupling factor 6, mitochondrial OS=Homo sapiens GN=ATP5J PE=1 SV=1 - [ATP5J\_HUMAN]** | | | | | | | | | | | | | | | | | | | | | | | | | | | | | | |  |
|  | | | | | | | | | | | | | | | | | | | | | | | | | | | | | | | | |
|  | High |  | QTSGGPVDASSEYQQELER |  | CID |  |  |  | 31.96 |  |  |  | 1.00 |  | 1 |  | Mascot (2) |  | 2 |  | 1040.97839 |  | 3.68 |  | 1268 |  | 1268 |  | Image |  | Peak List |  |
|  | | | | | | | | | | | | | | | | | | | | | | | | | | | | | | | | |
|  | High |  | QTSGGPVDASSEYQQELER |  | CID |  |  |  |  |  | 2.61 |  | 1.00 |  | 1 |  | SEQUEST (4) |  | 2 |  | 1040.97839 |  | 3.68 |  | 1268 |  | 1268 |  | Image |  | Peak List |  |
|  | | | | | | | | | | | | | | | | | | | | | | | | | | | | | | | | |
|  | **P20962 - Parathymosin OS=Homo sapiens GN=PTMS PE=1 SV=2 - [PTMS\_HUMAN]** | | | | | | | | | | | | | | | | | | | | | | | | | | | | | | |  |
|  | | | | | | | | | | | | | | | | | | | | | | | | | | | | | | | | |
|  | High |  | SVEAAAELSAK |  | CID |  |  |  | 41.74 |  |  |  | 1.00 |  | 1 |  | Mascot (2) |  | 2 |  | 538.28607 |  | 1.70 |  | 908 |  | 908 |  | Image |  | Peak List |  |
|  | | | | | | | | | | | | | | | | | | | | | | | | | | | | | | | | |
|  | High |  | SVEAAAELSAK |  | CID |  |  |  |  |  | 2.55 |  | 1.00 |  | 1 |  | SEQUEST (4) |  | 2 |  | 538.28607 |  | 1.70 |  | 908 |  | 908 |  | Image |  | Peak List |  |
|  | | | | | | | | | | | | | | | | | | | | | | | | | | | | | | | | |
|  | **P23141 - Liver carboxylesterase 1 OS=Homo sapiens GN=CES1 PE=1 SV=2 - [EST1\_HUMAN]** | | | | | | | | | | | | | | | | | | | | | | | | | | | | | | |  |
|  | | | | | | | | | | | | | | | | | | | | | | | | | | | | | | | | |
|  | High |  | DLFLDLIADVMFGVPSVIVAR |  | CID |  |  |  | 39.92 |  |  |  | 1.00 |  | 1 |  | Mascot (2) |  | 3 |  | 764.09399 |  | 6.87 |  | 6352 |  | 6352 |  | Image |  | Peak List |  |
|  | | | | | | | | | | | | | | | | | | | | | | | | | | | | | | | | |
|  | High |  | DLFLDLIADVMFGVPSVIVAR |  | CID |  |  |  |  |  | 3.49 |  | 1.00 |  | 1 |  | SEQUEST (4) |  | 3 |  | 764.09326 |  | 5.91 |  | 6346 |  | 6346 |  | Image |  | Peak List |  |
|  | | | | | | | | | | | | | | | | | | | | | | | | | | | | | | | | |
|  | **P25325 - 3-mercaptopyruvate sulfurtransferase OS=Homo sapiens GN=MPST PE=1 SV=3 - [THTM\_HUMAN]** | | | | | | | | | | | | | | | | | | | | | | | | | | | | | | |  |
|  | | | | | | | | | | | | | | | | | | | | | | | | | | | | | | | | |
|  | High |  | ALVSAQWVAEALR |  | CID |  |  |  | 87.85 |  |  |  | 1.00 |  | 1 |  | Mascot (2) |  | 2 |  | 707.39862 |  | 3.55 |  | 2717 |  | 2717 |  | Image |  | Peak List |  |
|  | | | | | | | | | | | | | | | | | | | | | | | | | | | | | | | | |
|  | High |  | ALVSAQWVAEALR |  | CID |  |  |  |  |  | 4.36 |  | 1.00 |  | 1 |  | SEQUEST (4) |  | 2 |  | 707.39862 |  | 3.55 |  | 2717 |  | 2717 |  | Image |  | Peak List |  |
|  | | | | | | | | | | | | | | | | | | | | | | | | | | | | | | | | |
|  | **P30039 - Phenazine biosynthesis-like domain-containing protein OS=Homo sapiens GN=PBLD PE=1 SV=2 - [PBLD\_HUMAN]** | | | | | | | | | | | | | | | | | | | | | | | | | | | | | | |  |
|  | | | | | | | | | | | | | | | | | | | | | | | | | | | | | | | | |
|  | High |  | LPIFIADAFTAR |  | CID |  |  |  | 39.73 |  |  |  | 1.00 |  | 1 |  | Mascot (2) |  | 2 |  | 667.87976 |  | 4.10 |  | 3048 |  | 3048 |  | Image |  | Peak List |  |
|  | | | | | | | | | | | | | | | | | | | | | | | | | | | | | | | | |
|  | High |  | LPIFIADAFTAR |  | CID |  |  |  |  |  | 3.05 |  | 1.00 |  | 1 |  | SEQUEST (4) |  | 2 |  | 667.87976 |  | 4.10 |  | 3048 |  | 3048 |  | Image |  | Peak List |  |
|  | | | | | | | | | | | | | | | | | | | | | | | | | | | | | | | | |
|  | **P30046 - D-dopachrome decarboxylase OS=Homo sapiens GN=DDT PE=1 SV=3 - [DOPD\_HUMAN]** | | | | | | | | | | | | | | | | | | | | | | | | | | | | | | |  |
|  | | | | | | | | | | | | | | | | | | | | | | | | | | | | | | | | |
|  | High |  | SHSAHFFEFLTK |  | CID |  |  |  | 22.30 |  |  |  | 1.00 |  | 1 |  | Mascot (2) |  | 3 |  | 484.24304 |  | 2.17 |  | 1418 |  | 1418 |  | Image |  | Peak List |  |
|  | | | | | | | | | | | | | | | | | | | | | | | | | | | | | | | | |
|  | High |  | SHSAHFFEFLTK |  | CID |  |  |  |  |  | 2.24 |  | 1.00 |  | 1 |  | SEQUEST (4) |  | 3 |  | 484.24304 |  | 2.17 |  | 1418 |  | 1418 |  | Image |  | Peak List |  |
|  | | | | | | | | | | | | | | | | | | | | | | | | | | | | | | | | |
|  | **P30084 - Enoyl-CoA hydratase, mitochondrial OS=Homo sapiens GN=ECHS1 PE=1 SV=4 - [ECHM\_HUMAN]** | | | | | | | | | | | | | | | | | | | | | | | | | | | | | | |  |
|  | | | | | | | | | | | | | | | | | | | | | | | | | | | | | | | | |
|  | High |  | ESVNAAFEMTLTEGSK |  | CID |  |  |  | 35.47 |  |  |  | 1.00 |  | 1 |  | Mascot (2) |  | 2 |  | 857.40717 |  | 4.07 |  | 2067 |  | 2067 |  | Image |  | Peak List |  |
|  | | | | | | | | | | | | | | | | | | | | | | | | | | | | | | | | |
|  | High |  | ESVNAAFEMTLTEGSK |  | CID |  |  |  |  |  | 2.46 |  | 1.00 |  | 1 |  | SEQUEST (4) |  | 2 |  | 857.40717 |  | 4.07 |  | 2067 |  | 2067 |  | Image |  | Peak List |  |
|  | | | | | | | | | | | | | | | | | | | | | | | | | | | | | | | | |
|  | **P31327 - Carbamoyl-phosphate synthase [ammonia], mitochondrial OS=Homo sapiens GN=CPS1 PE=1 SV=2 - [CPSM\_HUMAN]** | | | | | | | | | | | | | | | | | | | | | | | | | | | | | | |  |
|  | | | | | | | | | | | | | | | | | | | | | | | | | | | | | | | | |
|  | High |  | VLILGSGGLSIGQAGEFDYSGSQAVK |  | CID |  |  |  | 30.05 |  |  |  | 1.00 |  | 1 |  | Mascot (2) |  | 3 |  | 851.78143 |  | 3.96 |  | 2799 |  | 2799 |  | Image |  | Peak List |  |
|  | | | | | | | | | | | | | | | | | | | | | | | | | | | | | | | | |
|  | High |  | VLILGSGGLSIGQAGEFDYSGSQAVK |  | CID |  |  |  |  |  | 4.17 |  | 1.00 |  | 1 |  | SEQUEST (4) |  | 3 |  | 851.78143 |  | 3.96 |  | 2799 |  | 2799 |  | Image |  | Peak List |  |
|  | | | | | | | | | | | | | | | | | | | | | | | | | | | | | | | | |
|  | **P52758 - Ribonuclease UK114 OS=Homo sapiens GN=HRSP12 PE=1 SV=1 - [UK114\_HUMAN]** | | | | | | | | | | | | | | | | | | | | | | | | | | | | | | |  |
|  | | | | | | | | | | | | | | | | | | | | | | | | | | | | | | | | |
|  | High |  | TTVLLADINDFNTVNEIYK |  | CID |  |  |  | 51.25 |  |  |  | 1.00 |  | 1 |  | Mascot (2) |  | 2 |  | 1092.07043 |  | 4.80 |  | 3018 |  | 3018 |  | Image |  | Peak List |  |
|  | | | | | | | | | | | | | | | | | | | | | | | | | | | | | | | | |
|  | High |  | TTVLLADINDFNTVNEIYK |  | CID |  |  |  |  |  | 5.21 |  | 1.00 |  | 1 |  | SEQUEST (4) |  | 2 |  | 1092.07043 |  | 4.80 |  | 3018 |  | 3018 |  | Image |  | Peak List |  |
|  | | | | | | | | | | | | | | | | | | | | | | | | | | | | | | | | |
|  | **P61457 - Pterin-4-alpha-carbinolamine dehydratase OS=Homo sapiens GN=PCBD1 PE=1 SV=2 - [PHS\_HUMAN]** | | | | | | | | | | | | | | | | | | | | | | | | | | | | | | |  |
|  | | | | | | | | | | | | | | | | | | | | | | | | | | | | | | | | |
|  | High |  | AFGFMTR |  | CID |  |  |  | 32.79 |  |  |  | 1.00 |  | 1 |  | Mascot (2) |  | 2 |  | 415.20532 |  | 0.96 |  | 1570 |  | 1570 |  | Image |  | Peak List |  |
|  | | | | | | | | | | | | | | | | | | | | | | | | | | | | | | | | |
|  | **P62158 - Calmodulin OS=Homo sapiens GN=CALM1 PE=1 SV=2 - [CALM\_HUMAN]** | | | | | | | | | | | | | | | | | | | | | | | | | | | | | | |  |
|  | | | | | | | | | | | | | | | | | | | | | | | | | | | | | | | | |
|  | High |  | VFDKDGNGYISAAELR |  | CID |  |  |  | 16.37 |  |  |  | 1.00 |  | 1 |  | Mascot (2) |  | 3 |  | 585.63049 |  | 3.45 |  | 1373 |  | 1373 |  | Image |  | Peak List |  |
|  | | | | | | | | | | | | | | | | | | | | | | | | | | | | | | | | |
|  | High |  | VFDKDGNGYISAAELR |  | CID |  |  |  |  |  | 2.65 |  | 1.00 |  | 1 |  | SEQUEST (4) |  | 3 |  | 585.62939 |  | 1.57 |  | 1388 |  | 1388 |  | Image |  | Peak List |  |
|  | | | | | | | | | | | | | | | | | | | | | | | | | | | | | | | | |
|  | **Q03154 - Aminoacylase-1 OS=Homo sapiens GN=ACY1 PE=1 SV=1 - [ACY1\_HUMAN]** | | | | | | | | | | | | | | | | | | | | | | | | | | | | | | |  |
|  | | | | | | | | | | | | | | | | | | | | | | | | | | | | | | | | |
|  | High |  | VVNSILAFR |  | CID |  |  |  | 43.28 |  |  |  | 1.00 |  | 1 |  | Mascot (2) |  | 2 |  | 509.80646 |  | 1.18 |  | 1809 |  | 1809 |  | Image |  | Peak List |  |
|  | | | | | | | | | | | | | | | | | | | | | | | | | | | | | | | | |
|  | High |  | VVNSILAFR |  | CID |  |  |  |  |  | 2.28 |  | 1.00 |  | 1 |  | SEQUEST (4) |  | 2 |  | 509.80646 |  | 1.18 |  | 1809 |  | 1809 |  | Image |  | Peak List |  |
|  | | | | | | | | | | | | | | | | | | | | | | | | | | | | | | | | |
|  | **Q93088 - Betaine--homocysteine S-methyltransferase 1 OS=Homo sapiens GN=BHMT PE=1 SV=2 - [BHMT1\_HUMAN]** | | | | | | | | | | | | | | | | | | | | | | | | | | | | | | |  |
|  | | | | | | | | | | | | | | | | | | | | | | | | | | | | | | | | |
|  | High |  | ISGQEVNEAACDIAR |  | CID |  |  |  | 40.37 |  |  |  | 1.00 |  | 1 |  | Mascot (2) |  | 2 |  | 788.37744 |  | 2.78 |  | 1352 |  | 1352 |  | Image |  | Peak List |  |
|  | | | | | | | | | | | | | | | | | | | | | | | | | | | | | | | | |
|  | High |  | ISGQEVNEAACDIAR |  | CID |  |  |  |  |  | 3.12 |  | 1.00 |  | 1 |  | SEQUEST (4) |  | 2 |  | 788.37744 |  | 2.78 |  | 1352 |  | 1352 |  | Image |  | Peak List |  |
|  | | | | | | | | | | | | | | | | | | | | | | | | | | | | | | | | |
|  | **Q96IU4 - Alpha/beta hydrolase domain-containing protein 14B OS=Homo sapiens GN=ABHD14B PE=1 SV=1 - [ABHEB\_HUMAN]** | | | | | | | | | | | | | | | | | | | | | | | | | | | | | | |  |
|  | | | | | | | | | | | | | | | | | | | | | | | | | | | | | | | | |
|  | High |  | TPALIVYGDQDPMGQTSFEHLK |  | CID |  |  |  | 51.42 |  |  |  | 1.00 |  | 1 |  | Mascot (2) |  | 3 |  | 816.40356 |  | 2.00 |  | 2099 |  | 2099 |  | Image |  | Peak List |  |
|  | | | | | | | | | | | | | | | | | | | | | | | | | | | | | | | | |
|  | High |  | TPALIVYGDQDPMGQTSFEHLK |  | CID |  |  |  |  |  | 4.40 |  | 1.00 |  | 1 |  | SEQUEST (4) |  | 3 |  | 816.40356 |  | 2.00 |  | 2099 |  | 2099 |  | Image |  | Peak List |  |
|  | | | | | | | | | | | | | | | | | | | | | | | | | | | | | | | | |
|  | **Q99497 - Protein DJ-1 OS=Homo sapiens GN=PARK7 PE=1 SV=2 - [PARK7\_HUMAN]** | | | | | | | | | | | | | | | | | | | | | | | | | | | | | | |  |
|  | | | | | | | | | | | | | | | | | | | | | | | | | | | | | | | | |
|  | High |  | GPGTSFEFALAIVEALNGK |  | CID |  |  |  | 61.52 |  |  |  | 1.00 |  | 1 |  | Mascot (2) |  | 2 |  | 961.01605 |  | 9.46 |  | 4316 |  | 4316 |  | Image |  | Peak List |  |
|  | | | | | | | | | | | | | | | | | | | | | | | | | | | | | | | | |
|  | High |  | GPGTSFEFALAIVEALNGK |  | CID |  |  |  |  |  | 5.01 |  | 1.00 |  | 1 |  | SEQUEST (4) |  | 2 |  | 961.01563 |  | 9.01 |  | 4303 |  | 4303 |  | Image |  | Peak List |  |
|  | | | | | | | | | | | | | | | | | | | | | | | | | | | | | | | | |
|  | **Q9BSH5 - Haloacid dehalogenase-like hydrolase domain-containing protein 3 OS=Homo sapiens GN=HDHD3 PE=1 SV=1 - [HDHD3\_HUMAN]** | | | | | | | | | | | | | | | | | | | | | | | | | | | | | | |  |
|  | | | | | | | | | | | | | | | | | | | | | | | | | | | | | | | | |
|  | High |  | AHGLEVEPSALEQGFR |  | CID |  |  |  | 27.05 |  |  |  | 1.00 |  | 1 |  | Mascot (2) |  | 3 |  | 580.62909 |  | 0.86 |  | 1612 |  | 1612 |  | Image |  | Peak List |  |
|  | | | | | | | | | | | | | | | | | | | | | | | | | | | | | | | | |
|  | High |  | AHGLEVEPSALEQGFR |  | CID |  |  |  |  |  | 3.71 |  | 1.00 |  | 1 |  | SEQUEST (4) |  | 3 |  | 580.62909 |  | 0.86 |  | 1612 |  | 1612 |  | Image |  | Peak List |  |
|  | | | | | | | | | | | | | | | | | | | | | | | | | | | | | | | | |
|  | **A2A299 - Protein-glutamine gamma-glutamyltransferase 2 (Fragment) OS=Homo sapiens GN=TGM2 PE=4 SV=1 - [A2A299\_HUMAN]** | | | | | | | | | | | | | | | | | | | | | | | | | | | | | | |  |
|  | | | | | | | | | | | | | | | | | | | | | | | | | | | | | | | | |
|  | High |  | NIPWNFGQFEDGILDICLILLDVNPK |  | CID |  |  |  |  |  | 2.52 |  | 1.00 |  | 1 |  | SEQUEST (4) |  | 3 |  | 996.19037 |  | 6.06 |  | 6084 |  | 6084 |  | Image |  | Peak List |  |
|  | | | | | | | | | | | | | | | | | | | | | | | | | | | | | | | | |
|  | **E9PN89 - Heat shock cognate 71 kDa protein (Fragment) OS=Homo sapiens GN=HSPA8 PE=3 SV=1 - [E9PN89\_HUMAN]** | | | | | | | | | | | | | | | | | | | | | | | | | | | | | | |  |
|  | | | | | | | | | | | | | | | | | | | | | | | | | | | | | | | | |
|  | High |  | NVLIFDLGGGTFDVSILTIEDGIFEVK |  | CID |  |  |  |  |  | 1.65 |  | 1.00 |  | 1 |  | SEQUEST (4) |  | 3 |  | 971.19049 |  | 7.85 |  | 5025 |  | 5025 |  | Image |  | Peak List |  |
|  | | | | | | | | | | | | | | | | | | | | | | | | | | | | | | | | |
|  | **H0YNJ5 - Solute carrier family 12 member 6 (Fragment) OS=Homo sapiens GN=SLC12A6 PE=4 SV=1 - [H0YNJ5\_HUMAN]** | | | | | | | | | | | | | | | | | | | | | | | | | | | | | | |  |
|  | | | | | | | | | | | | | | | | | | | | | | | | | | | | | | | | |
|  | High |  | MANYTNLTQGAKEHEEAENITEGK |  | CID |  |  |  |  |  | 1.64 |  | 1.00 |  | 1 |  | SEQUEST (4) |  | 5 |  | 536.45776 |  | 8.72 |  | 1592 |  | 1592 |  | Image |  | Peak List |  |
|  | | | | | | | | | | | | | | | | | | | | | | | | | | | | | | | | |
|  | **H7BZ45 - Catechol O-methyltransferase (Fragment) OS=Homo sapiens GN=COMT PE=4 SV=1 - [H7BZ45\_HUMAN]** | | | | | | | | | | | | | | | | | | | | | | | | | | | | | | |  |
|  | | | | | | | | | | | | | | | | | | | | | | | | | | | | | | | | |
|  | High |  | YDVDTLDMVFLDHWK |  | CID |  |  |  |  |  | 3.23 |  | 1.00 |  | 1 |  | SEQUEST (4) |  | 3 |  | 632.96948 |  | 5.35 |  | 3149 |  | 3149 |  | Image |  | Peak List |  |
|  | | | | | | | | | | | | | | | | | | | | | | | | | | | | | | | | |
|  | **O43598-2 - Isoform 2 of Deoxyribonucleoside 5'-monophosphate N-glycosidase OS=Homo sapiens GN=RCL - [RCL\_HUMAN]** | | | | | | | | | | | | | | | | | | | | | | | | | | | | | | |  |
|  | | | | | | | | | | | | | | | | | | | | | | | | | | | | | | | | |
|  | High |  | FGTVLTEHVAAAELGAR |  | CID |  |  |  |  |  | 2.61 |  | 1.00 |  | 1 |  | SEQUEST (4) |  | 3 |  | 581.31256 |  | -0.07 |  | 1628 |  | 1628 |  | Image |  | Peak List |  |
|  | | | | | | | | | | | | | | | | | | | | | | | | | | | | | | | | |
|  | **P06744 - Glucose-6-phosphate isomerase OS=Homo sapiens GN=GPI PE=1 SV=4 - [G6PI\_HUMAN]** | | | | | | | | | | | | | | | | | | | | | | | | | | | | | | |  |
|  | | | | | | | | | | | | | | | | | | | | | | | | | | | | | | | | |
|  | High |  | IFVQGIIWDINSFDQWGVELGK |  | CID |  |  |  |  |  | 1.86 |  | 1.00 |  | 1 |  | SEQUEST (4) |  | 3 |  | 855.45166 |  | 8.55 |  | 4338 |  | 4338 |  | Image |  | Peak List |  |
|  | | | | | | | | | | | | | | | | | | | | | | | | | | | | | | | | |
|  | **P21549 - Serine--pyruvate aminotransferase OS=Homo sapiens GN=AGXT PE=1 SV=1 - [SPYA\_HUMAN]** | | | | | | | | | | | | | | | | | | | | | | | | | | | | | | |  |
|  | | | | | | | | | | | | | | | | | | | | | | | | | | | | | | | | |
|  | High |  | DIVSYVIDHFDIEIMGGLGPSTGK |  | CID |  |  |  |  |  | 2.16 |  | 1.00 |  | 1 |  | SEQUEST (4) |  | 3 |  | 855.10138 |  | 5.69 |  | 4547 |  | 4547 |  | Image |  | Peak List |  |
|  | | | | | | | | | | | | | | | | | | | | | | | | | | | | | | | | |
|  | **P54819-4 - Isoform 4 of Adenylate kinase 2, mitochondrial OS=Homo sapiens GN=AK2 - [KAD2\_HUMAN]** | | | | | | | | | | | | | | | | | | | | | | | | | | | | | | |  |
|  | | | | | | | | | | | | | | | | | | | | | | | | | | | | | | | | |
|  | High |  | LVSDEMVVELIEK |  | CID |  |  |  |  |  | 2.51 |  | 1.00 |  | 1 |  | SEQUEST (4) |  | 2 |  | 752.40582 |  | 4.53 |  | 2842 |  | 2842 |  | Image |  | Peak List |  |
|  | | | | | | | | | | | | | | | | | | | | | | | | | | | | | | | | |
|  | **P54868-2 - Isoform 2 of Hydroxymethylglutaryl-CoA synthase, mitochondrial OS=Homo sapiens GN=HMGCS2 - [HMCS2\_HUMAN]** | | | | | | | | | | | | | | | | | | | | | | | | | | | | | | |  |
|  | | | | | | | | | | | | | | | | | | | | | | | | | | | | | | | | |
|  | High |  | LEVGTETIIDK |  | CID |  |  |  |  |  | 1.79 |  | 1.00 |  | 1 |  | SEQUEST (4) |  | 2 |  | 609.33612 |  | 2.09 |  | 1469 |  | 1469 |  | Image |  | Peak List |  |
|  | | | | | | | | | | | | | | | | | | | | | | | | | | | | | | | | |
|  | **Q06520 - Bile salt sulfotransferase OS=Homo sapiens GN=SULT2A1 PE=1 SV=3 - [ST2A1\_HUMAN]** | | | | | | | | | | | | | | | | | | | | | | | | | | | | | | |  |
|  | | | | | | | | | | | | | | | | | | | | | | | | | | | | | | | | |
|  | High |  | VRDEFVIRDEDVIILTYPK |  | CID |  |  |  |  |  | 3.32 |  | 1.00 |  | 1 |  | SEQUEST (4) |  | 3 |  | 774.08978 |  | -0.02 |  | 2176 |  | 2176 |  | Image |  | Peak List |  |
|  | | | | | | | | | | | | | | | | | | | | | | | | | | | | | | | | |
|  | **Q08257-2 - Isoform 2 of Quinone oxidoreductase OS=Homo sapiens GN=CRYZ - [QOR\_HUMAN]** | | | | | | | | | | | | | | | | | | | | | | | | | | | | | | |  |
|  | | | | | | | | | | | | | | | | | | | | | | | | | | | | | | | | |
|  | High |  | GIDIIIEMLANVNLSK |  | CID |  |  |  |  |  | 2.93 |  | 1.00 |  | 1 |  | SEQUEST (4) |  | 2 |  | 871.99567 |  | 6.81 |  | 4469 |  | 4469 |  | Image |  | Peak List |  |
|  | | | | | | | | | | | | | | | | | | | | | | | | | | | | | | | | |
|  | **F5H4S7 - Succinyl-CoA ligase [GDP-forming] subunit beta, mitochondrial OS=Homo sapiens GN=SUCLG2 PE=4 SV=1 - [F5H4S7\_HUMAN]** | | | | | | | | | | | | | | | | | | | | | | | | | | | | | | |  |
|  | | | | | | | | | | | | | | | | | | | | | | | | | | | | | | | | |
|  | High |  | DPNVVGQLAK |  | CID |  |  |  |  |  | 2.46 |  | 1.00 |  | 1 |  | SEQUEST (4) |  | 2 |  | 520.79083 |  | 0.81 |  | 1163 |  | 1163 |  | Image |  | Peak List |  |
|  | | | | | | | | | | | | | | | | | | | | | | | | | | | | | | | | |

  
Top
  

### Search Summary

Workflow created with Discoverer version: 1.4.0.288 (DBVersion:79)
  
  
================================================================================
  
  
Search name: Methanol\_70\_2\_MC3
  
Search description: -
  
Search date: 06/25/2014 16:31:29
  
  
================================================================================
  
  
The pipeline tree:
  
------------------
  
  
    |-(0) Spectrum Files
  
        |-(1) Spectrum Selector
  
            |-(2) Mascot
  
                |-(3) Percolator
  
            |-(4) SEQUEST
  
                |-(3) Percolator
  
  
================================================================================
  
  
Search name: Methanol\_70\_2\_MC3
  
Search description: -
  
Search date: 06/25/2014 16:31:29
  
  
================================================================================
  
  
The pipeline tree:
  
------------------
  
  
    |-(0) Spectrum Files
  
        |-(1) Spectrum Selector
  
            |-(2) Mascot
  
                |-(3) Percolator
  
            |-(4) SEQUEST
  
                |-(3) Percolator
  
  
------------------------------------------------------------------------------
  
Processing node 0: Spectrum Files
  
------------------------------------------------------------------------------
  
  
Input Data:
  
-----------------------------
  
File Name(s): E:\Jos\Methanol\_70\_2.raw
  
  
------------------------------------------------------------------------------
  
Processing node 1: Spectrum Selector
  
------------------------------------------------------------------------------
  
  
1. General Settings:
  
-----------------------------
  
Precursor Selection: Use MS1 Precursor
  
Use New Precursor Reevaluation: True
  
  
2. Spectrum Properties Filter:
  
-----------------------------
  
Lower RT Limit: 0
  
Upper RT Limit: 0
  
First Scan: 0
  
Last Scan: 0
  
Lowest Charge State: 0
  
Highest Charge State: 0
  
Min. Precursor Mass: 350 Da
  
Max. Precursor Mass: 5000 Da
  
Total Intensity Threshold: 0
  
Minimum Peak Count: 1
  
  
3. Scan Event Filters:
  
-----------------------------
  
MS Order: Is MS2
  
Activation Type: Is CID
  
Min. Collision Energy: 0
  
Max. Collision Energy: 1000
  
Scan Type: Is Full
  
Ionization Source: Is Nanospray
  
  
4. Peak Filters:
  
-----------------------------
  
S/N Threshold (FT-only): 1.5
  
  
5. Replacements for Unrecognized Properties:
  
-----------------------------
  
Unrecognized Charge Replacements: Automatic
  
Unrecognized Mass Analyzer Replacements: ITMS
  
Unrecognized MS Order Replacements: MS2
  
Unrecognized Activation Type Replacements: CID
  
Unrecognized Polarity Replacements: +
  
  
6. Just for Testing:
  
-----------------------------
  
Precursor Clipping Range Before: 2.5 Da
  
Precursor Clipping Range After: 5.5 Da
  
  
------------------------------------------------------------------------------
  
Processing node 2: Mascot
  
------------------------------------------------------------------------------
  
  
1. Input Data:
  
-----------------------------
  
Protein Database: SwissProt
  
Enzyme Name: Trypsin
  
Maximum Missed Cleavage Sites: 3
  
Instrument: Default
  
Taxonomy: . . . . . . . . . . . . . . . . Homo sapiens (human)
  
  
1.1 Peptide Scoring Options:
  
-----------------------------
  
Peptide Cut Off Score: 10
  
Peptide Without Protein Cut Off Score: 5
  
  
1.2 Protein Scoring Options:
  
-----------------------------
  
Use MudPIT Scoring: Automatic
  
Protein Relevance Threshold: 20
  
Protein Relevance Factor: 1
  
  
2. Tolerances:
  
-----------------------------
  
Precursor Mass Tolerance: 10 ppm
  
Fragment Mass Tolerance: 0.8 Da
  
Use Average Precursor Mass: False
  
  
4. Dynamic Modifications:
  
-----------------------------
  
1. Dynamic Modification: Oxidation (M)
  
  
------------------------------------------------------------------------------
  
Processing node 3: Percolator
  
------------------------------------------------------------------------------
  
  
1. Input Data:
  
-----------------------------
  
Maximum Delta Cn: 0.05
  
  
2. Decoy Database Search:
  
-----------------------------
  
Target FDR (Strict): 0.01
  
Target FDR (Relaxed): 0.05
  
Validation based on: q-Value
  
  
------------------------------------------------------------------------------
  
Processing node 4: SEQUEST
  
------------------------------------------------------------------------------
  
  
1. Input Data:
  
-----------------------------
  
Protein Database: HUMAN\_swiss\_Jos.fasta
  
Enzyme Name: Trypsin (Full)
  
Maximum Missed Cleavage Sites: 3
  
  
1.1 Peptide Scoring Options:
  
-----------------------------
  
Maximum Peptides Considered: 500
  
Maximum Peptides Output: 10
  
Calculate Probability Scores: False
  
Absolute XCorr Threshold: 0.4
  
Fragment Ion Cutoff Percentage: 0.1
  
Peptide Without Protein XCorr Threshold: 1.5
  
  
1.2 Protein Scoring Options:
  
-----------------------------
  
Maximum Protein References Per Peptide: 100
  
Protein Relevance Threshold: 1.5
  
Peptide Relevance Factor: 0.4
  
  
2. Tolerances:
  
-----------------------------
  
Precursor Mass Tolerance: 10 ppm
  
Fragment Mass Tolerance: 0.8 Da
  
Use Average Precursor Mass: False
  
Use Average Fragment Masses: False
  
  
3. Ion Series:
  
-----------------------------
  
Use Neutral Loss a Ions: True
  
Use Neutral Loss b Ions: True
  
Use Neutral Loss y Ions: True
  
Weight of a Ions: 0
  
Weight of b Ions: 1
  
Weight of c Ions: 0
  
Weight of x Ions: 0
  
Weight of y Ions: 1
  
Weight of z Ions: 0
  
  
4. Dynamic Modifications:
  
-----------------------------
  
Max. Modifications Per Peptide: 4
  
1. Dynamic Modification: Oxidation / +15.995 Da (M)
  
  
================================================================================
  
  
Processing details:
  
  
06/25/2014 04:55 PM (4):SEQUEST: Total search time was 1 min 16 s.
  
06/25/2014 04:55 PM (3):Percolator: Performing percolator for SEQUEST (4) took 38.1 s.
  
06/25/2014 04:55 PM (4):SEQUEST: Search completed
  
06/25/2014 04:55 PM (4):SEQUEST: 5520 protein(s) + 5125 decoy proteins scored and inserted into result file in 3.4 s.
  
06/25/2014 04:55 PM (4):SEQUEST: 5520 protein(s) scored
  
06/25/2014 04:55 PM (4):SEQUEST: Search result finalization started.
  
06/25/2014 04:55 PM (3):Percolator: Start reading Percolator results
  
06/25/2014 04:55 PM (3):Percolator: Processing took 5.616 cpu seconds or 5 seconds wall time
  
06/25/2014 04:55 PM (3):Percolator: Calibrating statistics - calculating Posterior error probabilities (PEPs)
  
06/25/2014 04:55 PM (3):Percolator: PSMId score q-value posterior\_error\_prob peptide proteinIds
  
06/25/2014 04:55 PM (3):Percolator: New pi\_0 estimate on merged list gives 267 peptides over q=0.0100
  
06/25/2014 04:55 PM (3):Percolator: Calibrating statistics - calculating q values
  
06/25/2014 04:55 PM (3):Percolator: Selecting pi\_0=0.8969
  
06/25/2014 04:55 PM (3):Percolator: Tossing out "redundant" PSMs keeping only the best scoring PSM for each unique peptide.
  
06/25/2014 04:55 PM (3):Percolator: Merging results from 3 datasets
  
06/25/2014 04:55 PM (3):Percolator: Found 364 target PSMs scoring over 1.0000% FDR level on testset
  
06/25/2014 04:55 PM (3):Percolator: 3.7790 0.0026 5.5802 0.0544 0.0088 -0.0007 -66.1761 0.1679 -3.7720 -0.0415 0.0854 0.0000 -5.4084 1.6460 4.4044 3.4752 8.5339 -2.3155 0.0000 -0.8409 0.0820 -0.0365 0.0614 -1.4206 0.4479 0.5922 0.5532 8.1301 0.0016 -39.1677 -0.0016 -21.5507 -0.0067 -10.3504
  
06/25/2014 04:55 PM (3):Percolator: 2.55 0.5575 1.5666 1.4531 0.2452 -0.6251 -0.9782 0.9443 -0.0334 -0.1191 0.7269 0.0000 -2.6379 0.7948 1.6280 0.8666 0.8516 -2.4678 0.0000 -0.4317 0.9862 -0.6056 1.0836 -3.9578 1.2558 0.9577 1.0604 1.7053 0.5455 -4.2410 -0.3001 -2.0125 -1.3609 -12.6974
  
06/25/2014 04:55 PM (3):Percolator: XCorr SpScore Delta Cn From Second PSM Binomial Score Isolation Interference [%] MH+ [Da] Delta Mass [Da] Delta Mass [ppm] Absolute Delta Mass [Da] Absolute Delta Mass [ppm] Peptide Length Is z=1 Is z=2 Is z=3 Is z=4 Is z=5 Is z>5 # Missed Cleavages Log Peptides Matched Log Total Intensity Fraction Matched Intensity [%] Fragment Coverage Series A, B, C [%] Fragment Coverage Series X, Y, Z [%] Log Matched Fragment Series Intensities A, B, C Log Matched Fragment Series Intensities X, Y, Z Longest Sequence Series A, B, C Longest Sequence Series X, Y, Z IQR Fragment Delta Mass [Da] IQR Fragment Delta Mass [ppm] Mean Fragment Delta Mass [Da] Mean Fragment Delta Mass [ppm] Mean Absolute Fragment Delta Mass [Da] Mean Absolute Fragment Delta Mass [ppm] m0
  
06/25/2014 04:55 PM (3):Percolator: # first line contains normalized weights, second line the raw weights
  
06/25/2014 04:55 PM (3):Percolator: Obtained weights (only showing weights of first cross validation set)
  
06/25/2014 04:55 PM (3):Percolator: Iteration 10 : After the iteration step, 506 target PSMs with q<0.01 were estimated by cross validation
  
06/25/2014 04:55 PM (3):Percolator: Iteration 9 : After the iteration step, 506 target PSMs with q<0.01 were estimated by cross validation
  
06/25/2014 04:55 PM (3):Percolator: Iteration 8 : After the iteration step, 505 target PSMs with q<0.01 were estimated by cross validation
  
06/25/2014 04:55 PM (3):Percolator: Iteration 7 : After the iteration step, 503 target PSMs with q<0.01 were estimated by cross validation
  
06/25/2014 04:55 PM (3):Percolator: Iteration 6 : After the iteration step, 502 target PSMs with q<0.01 were estimated by cross validation
  
06/25/2014 04:55 PM (3):Percolator: Iteration 5 : After the iteration step, 501 target PSMs with q<0.01 were estimated by cross validation
  
06/25/2014 04:55 PM (3):Percolator: Iteration 4 : After the iteration step, 498 target PSMs with q<0.01 were estimated by cross validation
  
06/25/2014 04:55 PM (3):Percolator: Iteration 3 : After the iteration step, 491 target PSMs with q<0.01 were estimated by cross validation
  
06/25/2014 04:55 PM (3):Percolator: Iteration 2 : After the iteration step, 476 target PSMs with q<0.01 were estimated by cross validation
  
06/25/2014 04:55 PM (3):Percolator: Iteration 1 : After the iteration step, 430 target PSMs with q<0.01 were estimated by cross validation
  
06/25/2014 04:55 PM (3):Percolator: ---Training with Cpos selected by cross validation, Cneg selected by cross validation, fdr=0.01
  
06/25/2014 04:55 PM (3):Percolator: Reading in data and feature calculation took 5.818 cpu seconds or 6 seconds wall time
  
06/25/2014 04:55 PM (3):Percolator: Estimating 250 over q=0.01 in initial direction
  
06/25/2014 04:55 PM (3):Percolator: Selected feature number 1 as initial search direction, could separate 162 positives in that direction
  
06/25/2014 04:55 PM (3):Percolator: Selected feature number 1 as initial search direction, could separate 151 positives in that direction
  
06/25/2014 04:55 PM (3):Percolator: Selected feature number 1 as initial search direction, could separate 185 positives in that direction
  
06/25/2014 04:55 PM (3):Percolator: selecting cneg by cross validation
  
06/25/2014 04:55 PM (3):Percolator: selecting cpos by cross validation
  
06/25/2014 04:55 PM (3):Percolator: Train/test set contains 3723 positives and 3932 negatives, size ratio=0.946846 and pi0=1
  
06/25/2014 04:55 PM (3):Percolator: 31e77142-29e9-402c-9ec2-8468a9513af0 e39a792e-622c-452d-b49b-59809cad79d0 Delta Cn From Second PSM Binomial Score b8754504-e95e-476b-b9a4-454d4bb53aeb 1d91a87b-953a-4887-9f22-f75a497a3538 Delta Mass [Da] Delta Mass [ppm] Absolute Delta Mass [Da] Absolute Delta Mass [ppm] Peptide Length Is z=1 Is z=2 Is z=3 Is z=4 Is z=5 Is z>5 041eb6d5-e486-44a0-9bc1-19e25811c686 Log Peptides Matched Log Total Intensity Fraction Matched Intensity [%] Fragment Coverage Series A, B, C [%] Fragment Coverage Series X, Y, Z [%] Log Matched Fragment Series Intensities A, B, C Log Matched Fragment Series Intensities X, Y, Z Longest Sequence Series A, B, C Longest Sequence Series X, Y, Z IQR Fragment Delta Mass [Da] IQR Fragment Delta Mass [ppm] Mean Fragment Delta Mass [Da] Mean Fragment Delta Mass [ppm] Mean Absolute Fragment Delta Mass [Da] Mean Absolute Fragment Delta Mass [ppm]
  
06/25/2014 04:55 PM (3):Percolator: Features:
  
06/25/2014 04:55 PM (3):Percolator: enzyme=Trypsin
  
06/25/2014 04:55 PM (3):Percolator: Hyperparameters fdr=0.01, Cpos=0, Cneg=0, maxNiter=10
  
06/25/2014 04:55 PM (3):Percolator: Started Wed Jun 25 16:55:14 2014
  
06/25/2014 04:55 PM (3):Percolator: C:\Program Files\Thermo\Discoverer 1.4\Tools\Percolator\percolator.exe -X C:\ProgramData\Thermo\Discoverer 1.4\Scratch\2714f219-1d6c-4f4e-8460-e085ef457781\output.xml -Z C:\ProgramData\Thermo\Discoverer 1.4\Scratch\2714f219-1d6c-4f4e-8460-e085ef457781\input.xml
  
06/25/2014 04:55 PM (3):Percolator: Issued command:
  
06/25/2014 04:55 PM (3):Percolator: Department of Genome Sciences at the University of Washington.
  
06/25/2014 04:55 PM (3):Percolator: Written by Lukas K+�ll (lukall@u.washington.edu) in the
  
06/25/2014 04:55 PM (3):Percolator: Copyright (c) 2006-9 University of Washington. All rights reserved.
  
06/25/2014 04:55 PM (3):Percolator: Percolator version 2.04, Build Date Feb 1 2012 03:35:34
  
06/25/2014 04:55 PM (3):Percolator: Starting Percolator
  
06/25/2014 04:55 PM (3):Percolator: The input file contains 3723 peptides, 3932 decoy peptides and 33 features.
  
06/25/2014 04:55 PM (3):Percolator: Creating input file for SEQUEST (4) took 24.8 s.
  
06/25/2014 04:54 PM (3):Percolator: Start calculating features for peptides of SEQUEST (4)
  
06/25/2014 04:54 PM (2):Mascot: Total search time was 1 min 31 s.
  
06/25/2014 04:54 PM (3):Percolator: Performing percolator for Mascot (2) took 33.2 s.
  
06/25/2014 04:54 PM (2):Mascot: Search completed
  
06/25/2014 04:54 PM (2):Mascot: 142 protein(s) + 24 decoy proteins scored and inserted into result file in 0.6 s.
  
06/25/2014 04:54 PM (2):Mascot: 142 protein(s) scored
  
06/25/2014 04:54 PM (2):Mascot: Search result finalization started.
  
06/25/2014 04:54 PM (3):Percolator: Start reading Percolator results
  
06/25/2014 04:54 PM (3):Percolator: Processing took 4.789 cpu seconds or 5 seconds wall time
  
06/25/2014 04:54 PM (3):Percolator: Calibrating statistics - calculating Posterior error probabilities (PEPs)
  
06/25/2014 04:54 PM (3):Percolator: PSMId score q-value posterior\_error\_prob peptide proteinIds
  
06/25/2014 04:54 PM (3):Percolator: New pi\_0 estimate on merged list gives 274 peptides over q=0.0100
  
06/25/2014 04:54 PM (3):Percolator: Calibrating statistics - calculating q values
  
06/25/2014 04:54 PM (3):Percolator: Selecting pi\_0=0.8862
  
06/25/2014 04:54 PM (3):Percolator: Tossing out "redundant" PSMs keeping only the best scoring PSM for each unique peptide.
  
06/25/2014 04:54 PM (3):Percolator: Merging results from 3 datasets
  
06/25/2014 04:54 PM (3):Percolator: Found 393 target PSMs scoring over 1.0000% FDR level on testset
  
06/25/2014 04:54 PM (3):Percolator: 0.0429 0.7718 0.0101 0.0080 -0.0011 24.0565 -0.0746 30.9755 -0.0946 0.0404 0.0000 -0.4774 1.0130 -0.5505 -0.7993 -1.0068 -1.3889 0.0000 -1.2355 0.0153 -0.0118 -0.0198 0.0289 0.3038 0.1168 0.5390 -2.9347 -0.0011 0.8152 -0.0004 -0.8569 -0.0007 2.8437
  
06/25/2014 04:54 PM (3):Percolator: 0.439 0.2399 0.3026 0.2388 -1.1272 0.3951 -0.4160 0.3112 -0.2747 0.3953 0.0000 -0.2240 0.4995 -0.2103 -0.2031 -0.0969 -1.4922 0.0000 -0.9468 0.2416 -0.2286 -0.4175 0.1714 0.9351 0.2063 1.1524 -0.7043 -0.3958 0.1113 -0.0845 -0.0965 -0.1556 -4.4834
  
06/25/2014 04:54 PM (3):Percolator: IonScore Delta Cn From Second PSM Binomial Score Isolation Interference [%] MH+ [Da] Delta Mass [Da] Delta Mass [ppm] Absolute Delta Mass [Da] Absolute Delta Mass [ppm] Peptide Length Is z=1 Is z=2 Is z=3 Is z=4 Is z=5 Is z>5 # Missed Cleavages Log Peptides Matched Log Total Intensity Fraction Matched Intensity [%] Fragment Coverage Series A, B, C [%] Fragment Coverage Series X, Y, Z [%] Log Matched Fragment Series Intensities A, B, C Log Matched Fragment Series Intensities X, Y, Z Longest Sequence Series A, B, C Longest Sequence Series X, Y, Z IQR Fragment Delta Mass [Da] IQR Fragment Delta Mass [ppm] Mean Fragment Delta Mass [Da] Mean Fragment Delta Mass [ppm] Mean Absolute Fragment Delta Mass [Da] Mean Absolute Fragment Delta Mass [ppm] m0
  
06/25/2014 04:54 PM (3):Percolator: # first line contains normalized weights, second line the raw weights
  
06/25/2014 04:54 PM (3):Percolator: Obtained weights (only showing weights of first cross validation set)
  
06/25/2014 04:54 PM (3):Percolator: Iteration 10 : After the iteration step, 501 target PSMs with q<0.01 were estimated by cross validation
  
06/25/2014 04:54 PM (3):Percolator: Iteration 9 : After the iteration step, 499 target PSMs with q<0.01 were estimated by cross validation
  
06/25/2014 04:54 PM (3):Percolator: Iteration 8 : After the iteration step, 501 target PSMs with q<0.01 were estimated by cross validation
  
06/25/2014 04:54 PM (3):Percolator: Iteration 7 : After the iteration step, 502 target PSMs with q<0.01 were estimated by cross validation
  
06/25/2014 04:54 PM (3):Percolator: Iteration 6 : After the iteration step, 498 target PSMs with q<0.01 were estimated by cross validation
  
06/25/2014 04:54 PM (3):Percolator: Iteration 5 : After the iteration step, 493 target PSMs with q<0.01 were estimated by cross validation
  
06/25/2014 04:54 PM (3):Percolator: Iteration 4 : After the iteration step, 491 target PSMs with q<0.01 were estimated by cross validation
  
06/25/2014 04:54 PM (3):Percolator: Iteration 3 : After the iteration step, 485 target PSMs with q<0.01 were estimated by cross validation
  
06/25/2014 04:54 PM (3):Percolator: Iteration 2 : After the iteration step, 479 target PSMs with q<0.01 were estimated by cross validation
  
06/25/2014 04:54 PM (3):Percolator: Iteration 1 : After the iteration step, 448 target PSMs with q<0.01 were estimated by cross validation
  
06/25/2014 04:54 PM (3):Percolator: ---Training with Cpos selected by cross validation, Cneg selected by cross validation, fdr=0.01
  
06/25/2014 04:54 PM (3):Percolator: Reading in data and feature calculation took 4.524 cpu seconds or 4 seconds wall time
  
06/25/2014 04:54 PM (3):Percolator: Estimating 305 over q=0.01 in initial direction
  
06/25/2014 04:54 PM (3):Percolator: Selected feature number 1 as initial search direction, could separate 189 positives in that direction
  
06/25/2014 04:54 PM (3):Percolator: Selected feature number 1 as initial search direction, could separate 195 positives in that direction
  
06/25/2014 04:54 PM (3):Percolator: Selected feature number 1 as initial search direction, could separate 226 positives in that direction
  
06/25/2014 04:54 PM (3):Percolator: selecting cneg by cross validation
  
06/25/2014 04:54 PM (3):Percolator: selecting cpos by cross validation
  
06/25/2014 04:54 PM (3):Percolator: Train/test set contains 3098 positives and 2999 negatives, size ratio=1.03301 and pi0=1
  
06/25/2014 04:54 PM (3):Percolator: e6e22773-e9a6-4a26-9694-1ca77a797099 Delta Cn From Second PSM Binomial Score b8754504-e95e-476b-b9a4-454d4bb53aeb 1d91a87b-953a-4887-9f22-f75a497a3538 Delta Mass [Da] Delta Mass [ppm] Absolute Delta Mass [Da] Absolute Delta Mass [ppm] Peptide Length Is z=1 Is z=2 Is z=3 Is z=4 Is z=5 Is z>5 041eb6d5-e486-44a0-9bc1-19e25811c686 Log Peptides Matched Log Total Intensity Fraction Matched Intensity [%] Fragment Coverage Series A, B, C [%] Fragment Coverage Series X, Y, Z [%] Log Matched Fragment Series Intensities A, B, C Log Matched Fragment Series Intensities X, Y, Z Longest Sequence Series A, B, C Longest Sequence Series X, Y, Z IQR Fragment Delta Mass [Da] IQR Fragment Delta Mass [ppm] Mean Fragment Delta Mass [Da] Mean Fragment Delta Mass [ppm] Mean Absolute Fragment Delta Mass [Da] Mean Absolute Fragment Delta Mass [ppm]
  
06/25/2014 04:54 PM (3):Percolator: Features:
  
06/25/2014 04:54 PM (3):Percolator: enzyme=Trypsin
  
06/25/2014 04:54 PM (3):Percolator: Hyperparameters fdr=0.01, Cpos=0, Cneg=0, maxNiter=10
  
06/25/2014 04:54 PM (3):Percolator: Started Wed Jun 25 16:54:37 2014
  
06/25/2014 04:54 PM (3):Percolator: C:\Program Files\Thermo\Discoverer 1.4\Tools\Percolator\percolator.exe -X C:\ProgramData\Thermo\Discoverer 1.4\Scratch\b8a1cd13-5e43-47a0-9505-14ad9f2df312\output.xml -Z C:\ProgramData\Thermo\Discoverer 1.4\Scratch\b8a1cd13-5e43-47a0-9505-14ad9f2df312\input.xml
  
06/25/2014 04:54 PM (3):Percolator: Issued command:
  
06/25/2014 04:54 PM (3):Percolator: Department of Genome Sciences at the University of Washington.
  
06/25/2014 04:54 PM (3):Percolator: Written by Lukas K+�ll (lukall@u.washington.edu) in the
  
06/25/2014 04:54 PM (3):Percolator: Copyright (c) 2006-9 University of Washington. All rights reserved.
  
06/25/2014 04:54 PM (3):Percolator: Percolator version 2.04, Build Date Feb 1 2012 03:35:34
  
06/25/2014 04:54 PM (3):Percolator: Starting Percolator
  
06/25/2014 04:54 PM (3):Percolator: The input file contains 3098 peptides, 2999 decoy peptides and 32 features.
  
06/25/2014 04:54 PM (3):Percolator: Creating input file for Mascot (2) took 22.3 s.
  
06/25/2014 04:54 PM (3):Percolator: Start calculating features for peptides of Mascot (2)
  
06/25/2014 04:54 PM (2):Mascot: Used mascot server http://fenn.bham.ac.uk/mascot/ with Mascot version 2.4.1
  
06/25/2014 04:54 PM (2):Mascot: Sending 2638 peptide hits (11693 peptides) to result file
  
06/25/2014 04:54 PM (2):Mascot: Sending 2474 decoy peptide hits (10832 peptides) to result file
  
06/25/2014 04:54 PM (2):Mascot: Reading decoy results
  
06/25/2014 04:54 PM (2):Mascot: Start translating results
  
06/25/2014 04:54 PM (2):Mascot: Start mapping modifications
  
06/25/2014 04:54 PM (2):Mascot: Received 160 proteins from Mascot server
  
06/25/2014 04:54 PM (2):Mascot: Start mapping 160 proteins
  
06/25/2014 04:54 PM (2):Mascot: Start parsing results
  
06/25/2014 04:54 PM (2):Mascot: Received Mascot result file (filename=../data/20140625/F004279.dat)
  
06/25/2014 04:54 PM (2):Mascot: Mascot Server completed
  
06/25/2014 04:53 PM (2):Mascot: Mascot result on server (filename=../data/20140625/F004279.dat)
  
06/25/2014 04:52 PM (2):Mascot: Start searching 4211 spectra
  
06/25/2014 04:52 PM (4):SEQUEST: Sending 211 decoy peptide hits (999 peptides) to result file
  
06/25/2014 04:52 PM (4):SEQUEST: Starting SEQUEST decoy search
  
06/25/2014 04:52 PM (4):SEQUEST: Sending 211 peptide hits (985 peptides) to result file
  
06/25/2014 04:52 PM (4):SEQUEST: Starting SEQUEST (search spectra 4000 - 4211)
  
06/25/2014 04:52 PM (4):SEQUEST: Sending 1000 decoy peptide hits (2741 peptides) to result file
  
06/25/2014 04:52 PM (4):SEQUEST: Starting SEQUEST decoy search
  
06/25/2014 04:52 PM (4):SEQUEST: Sending 1000 peptide hits (2723 peptides) to result file
  
06/25/2014 04:52 PM (4):SEQUEST: Starting SEQUEST (search spectra 3000 - 4000)
  
06/25/2014 04:52 PM (4):SEQUEST: Sending 1000 decoy peptide hits (5010 peptides) to result file
  
06/25/2014 04:52 PM (4):SEQUEST: Starting SEQUEST decoy search
  
06/25/2014 04:52 PM (4):SEQUEST: Sending 1000 peptide hits (4977 peptides) to result file
  
06/25/2014 04:52 PM (4):SEQUEST: Starting SEQUEST (search spectra 2000 - 3000)
  
06/25/2014 04:52 PM (4):SEQUEST: Sending 1000 decoy peptide hits (8876 peptides) to result file
  
06/25/2014 04:51 PM (4):SEQUEST: Starting SEQUEST decoy search
  
06/25/2014 04:51 PM (4):SEQUEST: Sending 1000 peptide hits (8852 peptides) to result file
  
06/25/2014 04:51 PM (4):SEQUEST: Starting SEQUEST (search spectra 1000 - 2000)
  
06/25/2014 04:51 PM (4):SEQUEST: Sending 1000 decoy peptide hits (9289 peptides) to result file
  
06/25/2014 04:51 PM (4):SEQUEST: Starting SEQUEST decoy search
  
06/25/2014 04:51 PM (4):SEQUEST: Sending 1000 peptide hits (9275 peptides) to result file
  
06/25/2014 04:51 PM (4):SEQUEST: Starting SEQUEST (search spectra 0 - 1000)
  
06/25/2014 04:51 PM (4):SEQUEST: There is already an adequate decoy FASTA index.
  
06/25/2014 04:51 PM (4):SEQUEST: Looking for existing decoy FASTA index.
  
06/25/2014 04:51 PM (4):SEQUEST: There is already an adequate target FASTA index.
  
06/25/2014 04:51 PM (4):SEQUEST: Looking for existing target FASTA index.
  
06/25/2014 04:51 PM (2):Mascot: Use mascot server http://fenn.bham.ac.uk/mascot/ with Mascot version 2.4.1
  
06/25/2014 04:50 PM (1):Spectrum Selector: Reading from File 1 of 1:E:\Jos\Methanol\_70\_2.raw (7682 spectra total)
  
  
  
Top
