# Supplementary material for: Top-Down and Bottom-Up Identification of Proteins by Liquid Extraction Surface Analysis Mass Spectrometry of Healthy and Diseased Human Liver Tissue
Source: J Am Soc Mass Spectrom. 2014 Sep 3;25(11):1953–61. doi: 10.1007/s13361-014-0967-z (PMC4197381; doi:10.1007/s13361-014-0967-z)
Supplement: Supplementary file 2 — (ZIP 20872 kb) [file 13361_2014_967_MOESM2_ESM.zip › Supplementary file 4/Water_2_MC3.zip!/index.html]

Annotated spectra


## Annotated spectra of Water\_2\_MC3.msf

go to Peptides  
go to Search Summary  

### Peptides

|  |  |  |  |  |  |  |  |  |  |  |  |  |  |  |  |  |  |  |  |  |  |  |  |  |  |  |  |  |  |  |  |  |
| --- | --- | --- | --- | --- | --- | --- | --- | --- | --- | --- | --- | --- | --- | --- | --- | --- | --- | --- | --- | --- | --- | --- | --- | --- | --- | --- | --- | --- | --- | --- | --- | --- |
|  | | | | | | | | | | | | | | | | | | | | | | | | | | | | | | | | |
| Confidence Sequence Activation Type Modifications IonScore XCorr ΔScore Rank Search Engine Charge Precursor m/z [Da] ΔM [ppm] First Scan Last Scan Annotated Spectrum Peak List  | | | | | | | | | | | | | | | | | | | | | | | | | | | | | | | | |  |  |  |  |  |  |  |  |  |  |  |  |  |  |  |  |  |  |  |  |  |  |  |  |  |  |  |  |  |  |  |  |
|  | | | | | | | | | | | | | | | | | | | | | | | | | | | | | | | | |
|  | **O14773 - Tripeptidyl-peptidase 1 OS=Homo sapiens GN=TPP1 PE=1 SV=2 - [TPP1\_HUMAN]** | | | | | | | | | | | | | | | | | | | | | | | | | | | | | | |  |
|  | | | | | | | | | | | | | | | | | | | | | | | | | | | | | | | | |
|  | High |  | GLTLLFASGDSGAGCWSVSGR |  | CID |  |  |  | 61.49 |  |  |  | 1.00 |  | 1 |  | Mascot (2) |  | 2 |  | 1020.99969 |  | 5.51 |  | 2923 |  | 2923 |  | Image |  | Peak List |  |
|  | | | | | | | | | | | | | | | | | | | | | | | | | | | | | | | | |
|  | High |  | GLTLLFASGDSGAGCWSVSGR |  | CID |  |  |  |  |  | 5.46 |  | 1.00 |  | 1 |  | SEQUEST (4) |  | 2 |  | 1020.99969 |  | 5.51 |  | 2923 |  | 2923 |  | Image |  | Peak List |  |
|  | | | | | | | | | | | | | | | | | | | | | | | | | | | | | | | | |
|  | **O43598 - 2'-deoxynucleoside 5'-phosphate N-hydrolase 1 OS=Homo sapiens GN=DNPH1 PE=1 SV=1 - [DNPH1\_HUMAN]** | | | | | | | | | | | | | | | | | | | | | | | | | | | | | | |  |
|  | | | | | | | | | | | | | | | | | | | | | | | | | | | | | | | | |
|  | High |  | FQVWDYEEGEVEALLDR |  | CID |  |  |  | 69.65 |  |  |  | 1.00 |  | 1 |  | Mascot (2) |  | 2 |  | 1049.49670 |  | 4.60 |  | 3433 |  | 3433 |  | Image |  | Peak List |  |
|  | | | | | | | | | | | | | | | | | | | | | | | | | | | | | | | | |
|  | High |  | FQVWDYEEGEVEALLDR |  | CID |  |  |  |  |  | 5.42 |  | 1.00 |  | 1 |  | SEQUEST (4) |  | 2 |  | 1049.49670 |  | 4.60 |  | 3433 |  | 3433 |  | Image |  | Peak List |  |
|  | | | | | | | | | | | | | | | | | | | | | | | | | | | | | | | | |
|  | **O60763 - General vesicular transport factor p115 OS=Homo sapiens GN=USO1 PE=1 SV=2 - [USO1\_HUMAN]** | | | | | | | | | | | | | | | | | | | | | | | | | | | | | | |  |
|  | | | | | | | | | | | | | | | | | | | | | | | | | | | | | | | | |
|  | High |  | QQENVTLLLSLLEEFDFHVR |  | CID |  |  |  | 24.45 |  |  |  | 1.00 |  | 1 |  | Mascot (2) |  | 3 |  | 810.76666 |  | 7.81 |  | 4000 |  | 4000 |  | Image |  | Peak List |  |
|  | | | | | | | | | | | | | | | | | | | | | | | | | | | | | | | | |
|  | High |  | QQENVTLLLSLLEEFDFHVR |  | CID |  |  |  |  |  | 3.13 |  | 1.00 |  | 1 |  | SEQUEST (4) |  | 3 |  | 810.76721 |  | 8.49 |  | 3990 |  | 3990 |  | Image |  | Peak List |  |
|  | | | | | | | | | | | | | | | | | | | | | | | | | | | | | | | | |
|  | **O75964 - ATP synthase subunit g, mitochondrial OS=Homo sapiens GN=ATP5L PE=1 SV=3 - [ATP5L\_HUMAN]** | | | | | | | | | | | | | | | | | | | | | | | | | | | | | | |  |
|  | | | | | | | | | | | | | | | | | | | | | | | | | | | | | | | | |
|  | High |  | EAVLNGLVATEVLMWFYVGEIIGK |  | CID |  |  |  | 20.47 |  |  |  | 1.00 |  | 1 |  | Mascot (2) |  | 3 |  | 884.48267 |  | 6.78 |  | 5615 |  | 5615 |  | Image |  | Peak List |  |
|  | | | | | | | | | | | | | | | | | | | | | | | | | | | | | | | | |
|  | High |  | EAVLNGLVATEVLMWFYVGEIIGK |  | CID |  |  |  |  |  | 3.21 |  | 1.00 |  | 1 |  | SEQUEST (4) |  | 3 |  | 884.48090 |  | 4.78 |  | 5596 |  | 5596 |  | Image |  | Peak List |  |
|  | | | | | | | | | | | | | | | | | | | | | | | | | | | | | | | | |
|  | **O95479 - GDH/6PGL endoplasmic bifunctional protein OS=Homo sapiens GN=H6PD PE=1 SV=2 - [G6PE\_HUMAN]** | | | | | | | | | | | | | | | | | | | | | | | | | | | | | | |  |
|  | | | | | | | | | | | | | | | | | | | | | | | | | | | | | | | | |
|  | High |  | LCAEEDQGAQIYAR |  | CID |  |  |  | 33.08 |  |  |  | 1.00 |  | 1 |  | Mascot (2) |  | 2 |  | 783.86835 |  | 4.89 |  | 1032 |  | 1032 |  | Image |  | Peak List |  |
|  | | | | | | | | | | | | | | | | | | | | | | | | | | | | | | | | |
|  | High |  | LCAEEDQGAQIYAR |  | CID |  |  |  |  |  | 2.83 |  | 1.00 |  | 1 |  | SEQUEST (4) |  | 2 |  | 783.86835 |  | 4.89 |  | 1032 |  | 1032 |  | Image |  | Peak List |  |
|  | | | | | | | | | | | | | | | | | | | | | | | | | | | | | | | | |
|  | **O95571 - Persulfide dioxygenase ETHE1, mitochondrial OS=Homo sapiens GN=ETHE1 PE=1 SV=2 - [ETHE1\_HUMAN]** | | | | | | | | | | | | | | | | | | | | | | | | | | | | | | |  |
|  | | | | | | | | | | | | | | | | | | | | | | | | | | | | | | | | |
|  | High |  | LSGAQADLHIEDGDSIR |  | CID |  |  |  | 23.53 |  |  |  | 1.00 |  | 1 |  | Mascot (2) |  | 3 |  | 599.63397 |  | 5.53 |  | 1112 |  | 1112 |  | Image |  | Peak List |  |
|  | | | | | | | | | | | | | | | | | | | | | | | | | | | | | | | | |
|  | High |  | LSGAQADLHIEDGDSIR |  | CID |  |  |  |  |  | 1.54 |  | 1.00 |  | 1 |  | SEQUEST (4) |  | 3 |  | 599.63397 |  | 5.53 |  | 1112 |  | 1112 |  | Image |  | Peak List |  |
|  | | | | | | | | | | | | | | | | | | | | | | | | | | | | | | | | |
|  | **P00915 - Carbonic anhydrase 1 OS=Homo sapiens GN=CA1 PE=1 SV=2 - [CAH1\_HUMAN]** | | | | | | | | | | | | | | | | | | | | | | | | | | | | | | |  |
|  | | | | | | | | | | | | | | | | | | | | | | | | | | | | | | | | |
|  | High |  | ESISVSSEQLAQFR |  | CID |  |  |  | 44.06 |  |  |  | 1.00 |  | 1 |  | Mascot (2) |  | 2 |  | 790.90143 |  | 2.55 |  | 1734 |  | 1734 |  | Image |  | Peak List |  |
|  | | | | | | | | | | | | | | | | | | | | | | | | | | | | | | | | |
|  | High |  | ESISVSSEQLAQFR |  | CID |  |  |  |  |  | 3.73 |  | 1.00 |  | 1 |  | SEQUEST (4) |  | 2 |  | 790.90143 |  | 2.55 |  | 1734 |  | 1734 |  | Image |  | Peak List |  |
|  | | | | | | | | | | | | | | | | | | | | | | | | | | | | | | | | |
|  | **P01023 - Alpha-2-macroglobulin OS=Homo sapiens GN=A2M PE=1 SV=3 - [A2MG\_HUMAN]** | | | | | | | | | | | | | | | | | | | | | | | | | | | | | | |  |
|  | | | | | | | | | | | | | | | | | | | | | | | | | | | | | | | | |
|  | High |  | GGVEDEVTLSAYITIALLEIPLTVTHPVVR |  | CID |  |  |  | 31.47 |  |  |  | 1.00 |  | 1 |  | Mascot (2) |  | 3 |  | 1069.27039 |  | 7.67 |  | 5109 |  | 5109 |  | Image |  | Peak List |  |
|  | | | | | | | | | | | | | | | | | | | | | | | | | | | | | | | | |
|  | High |  | GGVEDEVTLSAYITIALLEIPLTVTHPVVR |  | CID |  |  |  |  |  | 2.11 |  | 1.00 |  | 1 |  | SEQUEST (4) |  | 3 |  | 1069.27039 |  | 7.67 |  | 5109 |  | 5109 |  | Image |  | Peak List |  |
|  | | | | | | | | | | | | | | | | | | | | | | | | | | | | | | | | |
|  | **P02763 - Alpha-1-acid glycoprotein 1 OS=Homo sapiens GN=ORM1 PE=1 SV=1 - [A1AG1\_HUMAN]** | | | | | | | | | | | | | | | | | | | | | | | | | | | | | | |  |
|  | | | | | | | | | | | | | | | | | | | | | | | | | | | | | | | | |
|  | High |  | TYMLAFDVNDEKNWGLSVYADKPETTK |  | CID |  |  |  | 52.90 |  |  |  | 1.00 |  | 1 |  | Mascot (2) |  | 3 |  | 1045.84277 |  | 5.01 |  | 2338 |  | 2338 |  | Image |  | Peak List |  |
|  | | | | | | | | | | | | | | | | | | | | | | | | | | | | | | | | |
|  | High |  | TYMLAFDVNDEKNWGLSVYADKPETTK |  | CID |  |  |  |  |  | 5.20 |  | 1.00 |  | 1 |  | SEQUEST (4) |  | 3 |  | 1045.84277 |  | 5.01 |  | 2338 |  | 2338 |  | Image |  | Peak List |  |
|  | | | | | | | | | | | | | | | | | | | | | | | | | | | | | | | | |
|  | **P02795 - Metallothionein-2 OS=Homo sapiens GN=MT2A PE=1 SV=1 - [MT2\_HUMAN]** | | | | | | | | | | | | | | | | | | | | | | | | | | | | | | |  |
|  | | | | | | | | | | | | | | | | | | | | | | | | | | | | | | | | |
|  | High |  | MDPNCSCAAGDSCTCAGSCK |  | CID |  |  |  | 34.43 |  |  |  | 1.00 |  | 1 |  | Mascot (2) |  | 2 |  | 962.33215 |  | 5.25 |  | 1017 |  | 1017 |  | Image |  | Peak List |  |
|  | | | | | | | | | | | | | | | | | | | | | | | | | | | | | | | | |
|  | High |  | MDPNCSCAAGDSCTCAGSCK |  | CID |  |  |  |  |  | 2.61 |  | 1.00 |  | 1 |  | SEQUEST (4) |  | 2 |  | 962.33215 |  | 5.25 |  | 1017 |  | 1017 |  | Image |  | Peak List |  |
|  | | | | | | | | | | | | | | | | | | | | | | | | | | | | | | | | |
|  | **P05166 - Propionyl-CoA carboxylase beta chain, mitochondrial OS=Homo sapiens GN=PCCB PE=1 SV=3 - [PCCB\_HUMAN]** | | | | | | | | | | | | | | | | | | | | | | | | | | | | | | |  |
|  | | | | | | | | | | | | | | | | | | | | | | | | | | | | | | | | |
|  | High |  | LVYVFSQDFTVFGGSLSGAHAQK |  | CID |  |  |  | 22.45 |  |  |  | 1.00 |  | 1 |  | Mascot (2) |  | 3 |  | 820.08990 |  | 6.07 |  | 2623 |  | 2623 |  | Image |  | Peak List |  |
|  | | | | | | | | | | | | | | | | | | | | | | | | | | | | | | | | |
|  | High |  | LVYVFSQDFTVFGGSLSGAHAQK |  | CID |  |  |  |  |  | 1.94 |  | 1.00 |  | 1 |  | SEQUEST (4) |  | 3 |  | 820.08990 |  | 6.07 |  | 2623 |  | 2623 |  | Image |  | Peak List |  |
|  | | | | | | | | | | | | | | | | | | | | | | | | | | | | | | | | |
|  | **P05388 - 60S acidic ribosomal protein P0 OS=Homo sapiens GN=RPLP0 PE=1 SV=1 - [RLA0\_HUMAN]** | | | | | | | | | | | | | | | | | | | | | | | | | | | | | | |  |
|  | | | | | | | | | | | | | | | | | | | | | | | | | | | | | | | | |
|  | High |  | VLALSVETDYTFPLAEK |  | CID |  |  |  | 56.26 |  |  |  | 1.00 |  | 1 |  | Mascot (2) |  | 2 |  | 948.50818 |  | 4.71 |  | 2744 |  | 2744 |  | Image |  | Peak List |  |
|  | | | | | | | | | | | | | | | | | | | | | | | | | | | | | | | | |
|  | High |  | VLALSVETDYTFPLAEK |  | CID |  |  |  |  |  | 4.23 |  | 1.00 |  | 1 |  | SEQUEST (4) |  | 2 |  | 948.50818 |  | 4.71 |  | 2744 |  | 2744 |  | Image |  | Peak List |  |
|  | | | | | | | | | | | | | | | | | | | | | | | | | | | | | | | | |
|  | **P07339 - Cathepsin D OS=Homo sapiens GN=CTSD PE=1 SV=1 - [CATD\_HUMAN]** | | | | | | | | | | | | | | | | | | | | | | | | | | | | | | |  |
|  | | | | | | | | | | | | | | | | | | | | | | | | | | | | | | | | |
|  | High |  | LVDQNIFSFYLSR |  | CID |  |  |  | 52.68 |  |  |  | 1.00 |  | 1 |  | Mascot (2) |  | 2 |  | 801.42474 |  | 6.20 |  | 3014 |  | 3014 |  | Image |  | Peak List |  |
|  | | | | | | | | | | | | | | | | | | | | | | | | | | | | | | | | |
|  | High |  | LVDQNIFSFYLSR |  | CID |  |  |  |  |  | 3.24 |  | 1.00 |  | 1 |  | SEQUEST (4) |  | 2 |  | 801.42474 |  | 6.20 |  | 3014 |  | 3014 |  | Image |  | Peak List |  |
|  | | | | | | | | | | | | | | | | | | | | | | | | | | | | | | | | |
|  | **P07737 - Profilin-1 OS=Homo sapiens GN=PFN1 PE=1 SV=2 - [PROF1\_HUMAN]** | | | | | | | | | | | | | | | | | | | | | | | | | | | | | | |  |
|  | | | | | | | | | | | | | | | | | | | | | | | | | | | | | | | | |
|  | High |  | DSLLQDGEFSMDLR |  | CID |  |  |  | 60.28 |  |  |  | 1.00 |  | 1 |  | Mascot (2) |  | 2 |  | 813.38220 |  | 5.83 |  | 2693 |  | 2693 |  | Image |  | Peak List |  |
|  | | | | | | | | | | | | | | | | | | | | | | | | | | | | | | | | |
|  | High |  | DSLLQDGEFSMDLR |  | CID |  |  |  |  |  | 3.87 |  | 1.00 |  | 1 |  | SEQUEST (4) |  | 2 |  | 813.38220 |  | 5.83 |  | 2693 |  | 2693 |  | Image |  | Peak List |  |
|  | | | | | | | | | | | | | | | | | | | | | | | | | | | | | | | | |
|  | **P08133 - Annexin A6 OS=Homo sapiens GN=ANXA6 PE=1 SV=3 - [ANXA6\_HUMAN]** | | | | | | | | | | | | | | | | | | | | | | | | | | | | | | |  |
|  | | | | | | | | | | | | | | | | | | | | | | | | | | | | | | | | |
|  | High |  | MLVVLLQGTREEDDVVSEDLVQQDVQDLYEAGELK |  | CID |  |  |  | 26.79 |  |  |  | 1.00 |  | 1 |  | Mascot (2) |  | 4 |  | 994.75800 |  | 8.96 |  | 3422 |  | 3422 |  | Image |  | Peak List |  |
|  | | | | | | | | | | | | | | | | | | | | | | | | | | | | | | | | |
|  | High |  | MLVVLLQGTREEDDVVSEDLVQQDVQDLYEAGELK |  | CID |  |  |  |  |  | 5.53 |  | 1.00 |  | 1 |  | SEQUEST (4) |  | 4 |  | 994.75800 |  | 8.96 |  | 3422 |  | 3422 |  | Image |  | Peak List |  |
|  | | | | | | | | | | | | | | | | | | | | | | | | | | | | | | | | |
|  | **P10620 - Microsomal glutathione S-transferase 1 OS=Homo sapiens GN=MGST1 PE=1 SV=1 - [MGST1\_HUMAN]** | | | | | | | | | | | | | | | | | | | | | | | | | | | | | | |  |
|  | | | | | | | | | | | | | | | | | | | | | | | | | | | | | | | | |
|  | High |  | VDLTQVMDDEVFMAFASYATIILSK |  | CID |  |  |  | 41.58 |  |  |  | 1.00 |  | 1 |  | Mascot (2) |  | 3 |  | 936.47461 |  | 7.46 |  | 5863 |  | 5863 |  | Image |  | Peak List |  |
|  | | | | | | | | | | | | | | | | | | | | | | | | | | | | | | | | |
|  | **P10768 - S-formylglutathione hydrolase OS=Homo sapiens GN=ESD PE=1 SV=2 - [ESTD\_HUMAN]** | | | | | | | | | | | | | | | | | | | | | | | | | | | | | | |  |
|  | | | | | | | | | | | | | | | | | | | | | | | | | | | | | | | | |
|  | High |  | SGYHQSASEHGLVVIAPDTSPR |  | CID |  |  |  | 24.62 |  |  |  | 1.00 |  | 1 |  | Mascot (2) |  | 3 |  | 770.05359 |  | 6.27 |  | 966 |  | 966 |  | Image |  | Peak List |  |
|  | | | | | | | | | | | | | | | | | | | | | | | | | | | | | | | | |
|  | High |  | SGYHQSASEHGLVVIAPDTSPR |  | CID |  |  |  |  |  | 3.08 |  | 1.00 |  | 1 |  | SEQUEST (4) |  | 3 |  | 770.05359 |  | 6.27 |  | 966 |  | 966 |  | Image |  | Peak List |  |
|  | | | | | | | | | | | | | | | | | | | | | | | | | | | | | | | | |
|  | **P12694 - 2-oxoisovalerate dehydrogenase subunit alpha, mitochondrial OS=Homo sapiens GN=BCKDHA PE=1 SV=2 - [ODBA\_HUMAN]** | | | | | | | | | | | | | | | | | | | | | | | | | | | | | | |  |
|  | | | | | | | | | | | | | | | | | | | | | | | | | | | | | | | | |
|  | High |  | ISFYMTNYGEEGTHVGSAAALDNTDLVFGQYR |  | CID |  |  |  | 37.16 |  |  |  | 1.00 |  | 1 |  | Mascot (2) |  | 3 |  | 1176.22107 |  | 7.53 |  | 2649 |  | 2649 |  | Image |  | Peak List |  |
|  | | | | | | | | | | | | | | | | | | | | | | | | | | | | | | | | |
|  | High |  | ISFYMTNYGEEGTHVGSAAALDNTDLVFGQYR |  | CID |  |  |  |  |  | 4.25 |  | 1.00 |  | 1 |  | SEQUEST (4) |  | 3 |  | 1176.21826 |  | 5.14 |  | 2626 |  | 2626 |  | Image |  | Peak List |  |
|  | | | | | | | | | | | | | | | | | | | | | | | | | | | | | | | | |
|  | **P13693 - Translationally-controlled tumor protein OS=Homo sapiens GN=TPT1 PE=1 SV=1 - [TCTP\_HUMAN]** | | | | | | | | | | | | | | | | | | | | | | | | | | | | | | |  |
|  | | | | | | | | | | | | | | | | | | | | | | | | | | | | | | | | |
|  | High |  | EIADGLCLEVEGK |  | CID |  |  |  | 29.21 |  |  |  | 1.00 |  | 1 |  | Mascot (2) |  | 2 |  | 688.34497 |  | 3.80 |  | 1995 |  | 1995 |  | Image |  | Peak List |  |
|  | | | | | | | | | | | | | | | | | | | | | | | | | | | | | | | | |
|  | High |  | EIADGLCLEVEGK |  | CID |  |  |  |  |  | 2.52 |  | 1.00 |  | 1 |  | SEQUEST (4) |  | 2 |  | 688.34497 |  | 3.80 |  | 1995 |  | 1995 |  | Image |  | Peak List |  |
|  | | | | | | | | | | | | | | | | | | | | | | | | | | | | | | | | |
|  | **P14618 - Pyruvate kinase PKM OS=Homo sapiens GN=PKM PE=1 SV=4 - [KPYM\_HUMAN]** | | | | | | | | | | | | | | | | | | | | | | | | | | | | | | |  |
|  | | | | | | | | | | | | | | | | | | | | | | | | | | | | | | | | |
|  | High |  | FGVEQDVDMVFASFIR |  | CID |  |  |  | 30.68 |  |  |  | 1.00 |  | 1 |  | Mascot (2) |  | 2 |  | 930.46045 |  | 7.46 |  | 3887 |  | 3887 |  | Image |  | Peak List |  |
|  | | | | | | | | | | | | | | | | | | | | | | | | | | | | | | | | |
|  | High |  | FGVEQDVDMVFASFIR |  | CID |  |  |  |  |  | 3.68 |  | 1.00 |  | 1 |  | SEQUEST (4) |  | 2 |  | 930.46045 |  | 7.46 |  | 3887 |  | 3887 |  | Image |  | Peak List |  |
|  | | | | | | | | | | | | | | | | | | | | | | | | | | | | | | | | |
|  | **P15428 - 15-hydroxyprostaglandin dehydrogenase [NAD(+)] OS=Homo sapiens GN=HPGD PE=1 SV=1 - [PGDH\_HUMAN]** | | | | | | | | | | | | | | | | | | | | | | | | | | | | | | |  |
|  | | | | | | | | | | | | | | | | | | | | | | | | | | | | | | | | |
|  | High |  | TLFIQCDVADQQQLR |  | CID |  |  |  | 48.57 |  |  |  | 1.00 |  | 1 |  | Mascot (2) |  | 2 |  | 889.45355 |  | 5.40 |  | 2046 |  | 2046 |  | Image |  | Peak List |  |
|  | | | | | | | | | | | | | | | | | | | | | | | | | | | | | | | | |
|  | High |  | TLFIQCDVADQQQLR |  | CID |  |  |  |  |  | 4.31 |  | 1.00 |  | 1 |  | SEQUEST (4) |  | 2 |  | 889.45355 |  | 5.40 |  | 2046 |  | 2046 |  | Image |  | Peak List |  |
|  | | | | | | | | | | | | | | | | | | | | | | | | | | | | | | | | |
|  | **P16435 - NADPH--cytochrome P450 reductase OS=Homo sapiens GN=POR PE=1 SV=2 - [NCPR\_HUMAN]** | | | | | | | | | | | | | | | | | | | | | | | | | | | | | | |  |
|  | | | | | | | | | | | | | | | | | | | | | | | | | | | | | | | | |
|  | High |  | HLMHLELDISDSK |  | CID |  |  |  | 21.35 |  |  |  | 1.00 |  | 1 |  | Mascot (2) |  | 3 |  | 513.26294 |  | 4.09 |  | 1250 |  | 1250 |  | Image |  | Peak List |  |
|  | | | | | | | | | | | | | | | | | | | | | | | | | | | | | | | | |
|  | High |  | HLMHLELDISDSK |  | CID |  |  |  |  |  | 2.73 |  | 1.00 |  | 1 |  | SEQUEST (4) |  | 3 |  | 513.26294 |  | 4.09 |  | 1250 |  | 1250 |  | Image |  | Peak List |  |
|  | | | | | | | | | | | | | | | | | | | | | | | | | | | | | | | | |
|  | **P17987 - T-complex protein 1 subunit alpha OS=Homo sapiens GN=TCP1 PE=1 SV=1 - [TCPA\_HUMAN]** | | | | | | | | | | | | | | | | | | | | | | | | | | | | | | |  |
|  | | | | | | | | | | | | | | | | | | | | | | | | | | | | | | | | |
|  | High |  | IIGINGDFFANMVVDAVLAIK |  | CID |  |  |  | 43.99 |  |  |  | 1.00 |  | 1 |  | Mascot (2) |  | 3 |  | 740.74670 |  | 7.09 |  | 4907 |  | 4907 |  | Image |  | Peak List |  |
|  | | | | | | | | | | | | | | | | | | | | | | | | | | | | | | | | |
|  | High |  | IIGINGDFFANMVVDAVLAIK |  | CID |  |  |  |  |  | 4.88 |  | 1.00 |  | 1 |  | SEQUEST (4) |  | 3 |  | 740.74670 |  | 7.09 |  | 4907 |  | 4907 |  | Image |  | Peak List |  |
|  | | | | | | | | | | | | | | | | | | | | | | | | | | | | | | | | |
|  | **P20618 - Proteasome subunit beta type-1 OS=Homo sapiens GN=PSMB1 PE=1 SV=2 - [PSB1\_HUMAN]** | | | | | | | | | | | | | | | | | | | | | | | | | | | | | | |  |
|  | | | | | | | | | | | | | | | | | | | | | | | | | | | | | | | | |
|  | High |  | FSPYVFNGGTILAIAGEDFAIVASDTR |  | CID |  |  |  | 32.29 |  |  |  | 1.00 |  | 1 |  | Mascot (2) |  | 3 |  | 944.48627 |  | 6.74 |  | 4457 |  | 4457 |  | Image |  | Peak List |  |
|  | | | | | | | | | | | | | | | | | | | | | | | | | | | | | | | | |
|  | High |  | FSPYVFNGGTILAIAGEDFAIVASDTR |  | CID |  |  |  |  |  | 4.51 |  | 1.00 |  | 1 |  | SEQUEST (4) |  | 3 |  | 944.48627 |  | 6.74 |  | 4457 |  | 4457 |  | Image |  | Peak List |  |
|  | | | | | | | | | | | | | | | | | | | | | | | | | | | | | | | | |
|  | **P21695 - Glycerol-3-phosphate dehydrogenase [NAD(+)], cytoplasmic OS=Homo sapiens GN=GPD1 PE=1 SV=4 - [GPDA\_HUMAN]** | | | | | | | | | | | | | | | | | | | | | | | | | | | | | | |  |
|  | | | | | | | | | | | | | | | | | | | | | | | | | | | | | | | | |
|  | High |  | NVVAVGAGFCDGLGFGDNTK |  | CID |  |  |  | 66.68 |  |  |  | 1.00 |  | 1 |  | Mascot (2) |  | 2 |  | 970.96771 |  | 5.64 |  | 2552 |  | 2552 |  | Image |  | Peak List |  |
|  | | | | | | | | | | | | | | | | | | | | | | | | | | | | | | | | |
|  | High |  | NVVAVGAGFCDGLGFGDNTK |  | CID |  |  |  |  |  | 5.13 |  | 1.00 |  | 1 |  | SEQUEST (4) |  | 2 |  | 970.96771 |  | 5.64 |  | 2552 |  | 2552 |  | Image |  | Peak List |  |
|  | | | | | | | | | | | | | | | | | | | | | | | | | | | | | | | | |
|  | **P23396 - 40S ribosomal protein S3 OS=Homo sapiens GN=RPS3 PE=1 SV=2 - [RS3\_HUMAN]** | | | | | | | | | | | | | | | | | | | | | | | | | | | | | | |  |
|  | | | | | | | | | | | | | | | | | | | | | | | | | | | | | | | | |
|  | High |  | ELAEDGYSGVEVR |  | CID |  |  |  | 26.73 |  |  |  | 1.00 |  | 1 |  | Mascot (2) |  | 2 |  | 712.34216 |  | 4.93 |  | 1137 |  | 1137 |  | Image |  | Peak List |  |
|  | | | | | | | | | | | | | | | | | | | | | | | | | | | | | | | | |
|  | High |  | ELAEDGYSGVEVR |  | CID |  |  |  |  |  | 2.38 |  | 1.00 |  | 1 |  | SEQUEST (4) |  | 2 |  | 712.34216 |  | 4.93 |  | 1137 |  | 1137 |  | Image |  | Peak List |  |
|  | | | | | | | | | | | | | | | | | | | | | | | | | | | | | | | | |
|  | **P25325 - 3-mercaptopyruvate sulfurtransferase OS=Homo sapiens GN=MPST PE=1 SV=3 - [THTM\_HUMAN]** | | | | | | | | | | | | | | | | | | | | | | | | | | | | | | |  |
|  | | | | | | | | | | | | | | | | | | | | | | | | | | | | | | | | |
|  | High |  | LGVGAATHVVIYDASDQGLYSAPR |  | CID |  |  |  | 35.18 |  |  |  | 1.00 |  | 1 |  | Mascot (2) |  | 3 |  | 820.75818 |  | 3.30 |  | 1774 |  | 1774 |  | Image |  | Peak List |  |
|  | | | | | | | | | | | | | | | | | | | | | | | | | | | | | | | | |
|  | High |  | LGVGAATHVVIYDASDQGLYSAPR |  | CID |  |  |  |  |  | 4.11 |  | 1.00 |  | 1 |  | SEQUEST (4) |  | 3 |  | 820.75818 |  | 3.30 |  | 1774 |  | 1774 |  | Image |  | Peak List |  |
|  | | | | | | | | | | | | | | | | | | | | | | | | | | | | | | | | |
|  | **P28070 - Proteasome subunit beta type-4 OS=Homo sapiens GN=PSMB4 PE=1 SV=4 - [PSB4\_HUMAN]** | | | | | | | | | | | | | | | | | | | | | | | | | | | | | | |  |
|  | | | | | | | | | | | | | | | | | | | | | | | | | | | | | | | | |
|  | High |  | FEGGVVIAADMLGSYGSLAR |  | CID |  |  |  | 70.95 |  |  |  | 1.00 |  | 1 |  | Mascot (2) |  | 2 |  | 1007.01697 |  | 7.73 |  | 3161 |  | 3161 |  | Image |  | Peak List |  |
|  | | | | | | | | | | | | | | | | | | | | | | | | | | | | | | | | |
|  | High |  | FEGGVVIAADMLGSYGSLAR |  | CID |  |  |  |  |  | 5.32 |  | 1.00 |  | 1 |  | SEQUEST (4) |  | 2 |  | 1007.01697 |  | 7.73 |  | 3161 |  | 3161 |  | Image |  | Peak List |  |
|  | | | | | | | | | | | | | | | | | | | | | | | | | | | | | | | | |
|  | **P30042 - ES1 protein homolog, mitochondrial OS=Homo sapiens GN=C21orf33 PE=1 SV=3 - [ES1\_HUMAN]** | | | | | | | | | | | | | | | | | | | | | | | | | | | | | | |  |
|  | | | | | | | | | | | | | | | | | | | | | | | | | | | | | | | | |
|  | High |  | GVEVTVGHEQEEGGKWPYAGTAEAIK |  | CID |  |  |  | 22.54 |  |  |  | 1.00 |  | 1 |  | Mascot (2) |  | 3 |  | 914.78851 |  | 5.07 |  | 1239 |  | 1239 |  | Image |  | Peak List |  |
|  | | | | | | | | | | | | | | | | | | | | | | | | | | | | | | | | |
|  | High |  | GVEVTVGHEQEEGGKWPYAGTAEAIK |  | CID |  |  |  |  |  | 2.17 |  | 1.00 |  | 1 |  | SEQUEST (4) |  | 3 |  | 914.78851 |  | 5.07 |  | 1239 |  | 1239 |  | Image |  | Peak List |  |
|  | | | | | | | | | | | | | | | | | | | | | | | | | | | | | | | | |
|  | **P30043 - Flavin reductase (NADPH) OS=Homo sapiens GN=BLVRB PE=1 SV=3 - [BLVRB\_HUMAN]** | | | | | | | | | | | | | | | | | | | | | | | | | | | | | | |  |
|  | | | | | | | | | | | | | | | | | | | | | | | | | | | | | | | | |
|  | High |  | IAIFGATGQTGLTTLAQAVQAGYEVTVLVR |  | CID |  |  |  | 20.20 |  |  |  | 1.00 |  | 1 |  | Mascot (2) |  | 3 |  | 1016.90338 |  | 7.43 |  | 4216 |  | 4216 |  | Image |  | Peak List |  |
|  | | | | | | | | | | | | | | | | | | | | | | | | | | | | | | | | |
|  | High |  | IAIFGATGQTGLTTLAQAVQAGYEVTVLVR |  | CID |  |  |  |  |  | 3.02 |  | 1.00 |  | 1 |  | SEQUEST (4) |  | 3 |  | 1016.90411 |  | 8.15 |  | 4286 |  | 4286 |  | Image |  | Peak List |  |
|  | | | | | | | | | | | | | | | | | | | | | | | | | | | | | | | | |
|  | **P30048 - Thioredoxin-dependent peroxide reductase, mitochondrial OS=Homo sapiens GN=PRDX3 PE=1 SV=3 - [PRDX3\_HUMAN]** | | | | | | | | | | | | | | | | | | | | | | | | | | | | | | |  |
|  | | | | | | | | | | | | | | | | | | | | | | | | | | | | | | | | |
|  | High |  | ANEFHDVNCEVVAVSVDSHFSHLAWINTPR |  | CID |  |  |  | 41.10 |  |  |  | 1.00 |  | 1 |  | Mascot (2) |  | 3 |  | 1131.87866 |  | 4.23 |  | 2437 |  | 2437 |  | Image |  | Peak List |  |
|  | | | | | | | | | | | | | | | | | | | | | | | | | | | | | | | | |
|  | High |  | ANEFHDVNCEVVAVSVDSHFSHLAWINTPR |  | CID |  |  |  |  |  | 5.58 |  | 1.00 |  | 1 |  | SEQUEST (4) |  | 4 |  | 849.16125 |  | 4.75 |  | 2413 |  | 2413 |  | Image |  | Peak List |  |
|  | | | | | | | | | | | | | | | | | | | | | | | | | | | | | | | | |
|  | **P30050 - 60S ribosomal protein L12 OS=Homo sapiens GN=RPL12 PE=1 SV=1 - [RL12\_HUMAN]** | | | | | | | | | | | | | | | | | | | | | | | | | | | | | | |  |
|  | | | | | | | | | | | | | | | | | | | | | | | | | | | | | | | | |
|  | High |  | EILGTAQSVGCNVDGR |  | CID |  |  |  | 41.17 |  |  |  | 1.00 |  | 1 |  | Mascot (2) |  | 2 |  | 809.89990 |  | 4.38 |  | 1324 |  | 1324 |  | Image |  | Peak List |  |
|  | | | | | | | | | | | | | | | | | | | | | | | | | | | | | | | | |
|  | High |  | EILGTAQSVGCNVDGR |  | CID |  |  |  |  |  | 2.65 |  | 1.00 |  | 1 |  | SEQUEST (4) |  | 2 |  | 809.89990 |  | 4.38 |  | 1324 |  | 1324 |  | Image |  | Peak List |  |
|  | | | | | | | | | | | | | | | | | | | | | | | | | | | | | | | | |
|  | **P30101 - Protein disulfide-isomerase A3 OS=Homo sapiens GN=PDIA3 PE=1 SV=4 - [PDIA3\_HUMAN]** | | | | | | | | | | | | | | | | | | | | | | | | | | | | | | |  |
|  | | | | | | | | | | | | | | | | | | | | | | | | | | | | | | | | |
|  | High |  | DLLIAYYDVDYEK |  | CID |  |  |  | 73.81 |  |  |  | 1.00 |  | 1 |  | Mascot (2) |  | 2 |  | 810.40070 |  | 6.26 |  | 2696 |  | 2696 |  | Image |  | Peak List |  |
|  | | | | | | | | | | | | | | | | | | | | | | | | | | | | | | | | |
|  | High |  | DLLIAYYDVDYEK |  | CID |  |  |  |  |  | 4.60 |  | 1.00 |  | 1 |  | SEQUEST (4) |  | 2 |  | 810.40070 |  | 6.26 |  | 2696 |  | 2696 |  | Image |  | Peak List |  |
|  | | | | | | | | | | | | | | | | | | | | | | | | | | | | | | | | |
|  | **P32119 - Peroxiredoxin-2 OS=Homo sapiens GN=PRDX2 PE=1 SV=5 - [PRDX2\_HUMAN]** | | | | | | | | | | | | | | | | | | | | | | | | | | | | | | |  |
|  | | | | | | | | | | | | | | | | | | | | | | | | | | | | | | | | |
|  | High |  | LGCEVLGVSVDSQFTHLAWINTPR |  | CID |  |  |  | 12.85 |  |  |  | 1.00 |  | 1 |  | Mascot (2) |  | 3 |  | 881.45880 |  | 8.40 |  | 3096 |  | 3096 |  | Image |  | Peak List |  |
|  | | | | | | | | | | | | | | | | | | | | | | | | | | | | | | | | |
|  | High |  | LGCEVLGVSVDSQFTHLAWINTPR |  | CID |  |  |  |  |  | 2.89 |  | 1.00 |  | 1 |  | SEQUEST (4) |  | 3 |  | 881.45880 |  | 8.40 |  | 3096 |  | 3096 |  | Image |  | Peak List |  |
|  | | | | | | | | | | | | | | | | | | | | | | | | | | | | | | | | |
|  | **P32754 - 4-hydroxyphenylpyruvate dioxygenase OS=Homo sapiens GN=HPD PE=1 SV=2 - [HPPD\_HUMAN]** | | | | | | | | | | | | | | | | | | | | | | | | | | | | | | |  |
|  | | | | | | | | | | | | | | | | | | | | | | | | | | | | | | | | |
|  | High |  | DIAFEVEDCDYIVQK |  | CID |  |  |  | 100.01 |  |  |  | 1.00 |  | 1 |  | Mascot (2) |  | 2 |  | 893.91888 |  | 5.61 |  | 2646 |  | 2646 |  | Image |  | Peak List |  |
|  | | | | | | | | | | | | | | | | | | | | | | | | | | | | | | | | |
|  | High |  | DIAFEVEDCDYIVQK |  | CID |  |  |  |  |  | 5.07 |  | 1.00 |  | 1 |  | SEQUEST (4) |  | 2 |  | 893.91888 |  | 5.61 |  | 2646 |  | 2646 |  | Image |  | Peak List |  |
|  | | | | | | | | | | | | | | | | | | | | | | | | | | | | | | | | |
|  | **P34913 - Bifunctional epoxide hydrolase 2 OS=Homo sapiens GN=EPHX2 PE=1 SV=2 - [HYES\_HUMAN]** | | | | | | | | | | | | | | | | | | | | | | | | | | | | | | |  |
|  | | | | | | | | | | | | | | | | | | | | | | | | | | | | | | | | |
|  | High |  | MHFDFLIESCQVGMVKPEPQIYK |  | CID |  |  |  | 23.96 |  |  |  | 1.00 |  | 1 |  | Mascot (2) |  | 3 |  | 913.78772 |  | 5.17 |  | 2496 |  | 2496 |  | Image |  | Peak List |  |
|  | | | | | | | | | | | | | | | | | | | | | | | | | | | | | | | | |
|  | High |  | MHFDFLIESCQVGMVKPEPQIYK |  | CID |  |  |  |  |  | 3.58 |  | 1.00 |  | 1 |  | SEQUEST (4) |  | 3 |  | 913.78772 |  | 5.17 |  | 2496 |  | 2496 |  | Image |  | Peak List |  |
|  | | | | | | | | | | | | | | | | | | | | | | | | | | | | | | | | |
|  | **P38117 - Electron transfer flavoprotein subunit beta OS=Homo sapiens GN=ETFB PE=1 SV=3 - [ETFB\_HUMAN]** | | | | | | | | | | | | | | | | | | | | | | | | | | | | | | |  |
|  | | | | | | | | | | | | | | | | | | | | | | | | | | | | | | | | |
|  | High |  | HSMNPFCEIAVEEAVR |  | CID |  |  |  | 44.89 |  |  |  | 1.00 |  | 1 |  | Mascot (2) |  | 3 |  | 611.28943 |  | 3.86 |  | 2024 |  | 2024 |  | Image |  | Peak List |  |
|  | | | | | | | | | | | | | | | | | | | | | | | | | | | | | | | | |
|  | High |  | HSMNPFCEIAVEEAVR |  | CID |  |  |  |  |  | 3.49 |  | 1.00 |  | 1 |  | SEQUEST (4) |  | 3 |  | 611.28943 |  | 3.86 |  | 2024 |  | 2024 |  | Image |  | Peak List |  |
|  | | | | | | | | | | | | | | | | | | | | | | | | | | | | | | | | |
|  | **P40763 - Signal transducer and activator of transcription 3 OS=Homo sapiens GN=STAT3 PE=1 SV=2 - [STAT3\_HUMAN]** | | | | | | | | | | | | | | | | | | | | | | | | | | | | | | |  |
|  | | | | | | | | | | | | | | | | | | | | | | | | | | | | | | | | |
|  | High |  | VMNMEESNNGSLSAEFK |  | CID |  |  |  | 43.00 |  |  |  | 1.00 |  | 1 |  | Mascot (2) |  | 2 |  | 943.92169 |  | 5.36 |  | 1461 |  | 1461 |  | Image |  | Peak List |  |
|  | | | | | | | | | | | | | | | | | | | | | | | | | | | | | | | | |
|  | High |  | VMNMEESNNGSLSAEFK |  | CID |  |  |  |  |  | 2.67 |  | 1.00 |  | 1 |  | SEQUEST (4) |  | 2 |  | 943.92169 |  | 5.36 |  | 1461 |  | 1461 |  | Image |  | Peak List |  |
|  | | | | | | | | | | | | | | | | | | | | | | | | | | | | | | | | |
|  | **P40926 - Malate dehydrogenase, mitochondrial OS=Homo sapiens GN=MDH2 PE=1 SV=3 - [MDHM\_HUMAN]** | | | | | | | | | | | | | | | | | | | | | | | | | | | | | | |  |
|  | | | | | | | | | | | | | | | | | | | | | | | | | | | | | | | | |
|  | High |  | AGAGSATLSMAYAGAR |  | CID |  |  |  | 55.68 |  |  |  | 1.00 |  | 1 |  | Mascot (2) |  | 2 |  | 727.86078 |  | 5.88 |  | 1173 |  | 1173 |  | Image |  | Peak List |  |
|  | | | | | | | | | | | | | | | | | | | | | | | | | | | | | | | | |
|  | High |  | AGAGSATLSMAYAGAR |  | CID |  |  |  |  |  | 3.45 |  | 1.00 |  | 1 |  | SEQUEST (4) |  | 2 |  | 727.86078 |  | 5.88 |  | 1173 |  | 1173 |  | Image |  | Peak List |  |
|  | | | | | | | | | | | | | | | | | | | | | | | | | | | | | | | | |
|  | **P42126 - Enoyl-CoA delta isomerase 1, mitochondrial OS=Homo sapiens GN=ECI1 PE=1 SV=1 - [ECI1\_HUMAN]** | | | | | | | | | | | | | | | | | | | | | | | | | | | | | | |  |
|  | | | | | | | | | | | | | | | | | | | | | | | | | | | | | | | | |
|  | High |  | FKNPPVNSLSLEFLTELVISLEK |  | CID |  |  |  | 24.52 |  |  |  | 1.00 |  | 1 |  | Mascot (2) |  | 3 |  | 873.15918 |  | 5.40 |  | 4115 |  | 4115 |  | Image |  | Peak List |  |
|  | | | | | | | | | | | | | | | | | | | | | | | | | | | | | | | | |
|  | High |  | FKNPPVNSLSLEFLTELVISLEK |  | CID |  |  |  |  |  | 3.99 |  | 1.00 |  | 1 |  | SEQUEST (4) |  | 3 |  | 873.15918 |  | 5.40 |  | 4115 |  | 4115 |  | Image |  | Peak List |  |
|  | | | | | | | | | | | | | | | | | | | | | | | | | | | | | | | | |
|  | **P42357 - Histidine ammonia-lyase OS=Homo sapiens GN=HAL PE=1 SV=1 - [HUTH\_HUMAN]** | | | | | | | | | | | | | | | | | | | | | | | | | | | | | | |  |
|  | | | | | | | | | | | | | | | | | | | | | | | | | | | | | | | | |
|  | High |  | ALCHPSSVDSLSTSAATEDHVSMGGWAAR |  | CID |  |  |  | 20.38 |  |  |  | 1.00 |  | 1 |  | Mascot (2) |  | 4 |  | 736.59241 |  | 3.97 |  | 1473 |  | 1473 |  | Image |  | Peak List |  |
|  | | | | | | | | | | | | | | | | | | | | | | | | | | | | | | | | |
|  | High |  | ALCHPSSVDSLSTSAATEDHVSMGGWAAR |  | CID |  |  |  |  |  | 2.05 |  | 1.00 |  | 1 |  | SEQUEST (4) |  | 4 |  | 736.59241 |  | 3.97 |  | 1473 |  | 1473 |  | Image |  | Peak List |  |
|  | | | | | | | | | | | | | | | | | | | | | | | | | | | | | | | | |
|  | **P46783 - 40S ribosomal protein S10 OS=Homo sapiens GN=RPS10 PE=1 SV=1 - [RS10\_HUMAN]** | | | | | | | | | | | | | | | | | | | | | | | | | | | | | | |  |
|  | | | | | | | | | | | | | | | | | | | | | | | | | | | | | | | | |
|  | High |  | AEAGAGSATEFQFR |  | CID |  |  |  | 38.51 |  |  |  | 1.00 |  | 1 |  | Mascot (2) |  | 2 |  | 721.34369 |  | 6.51 |  | 1261 |  | 1261 |  | Image |  | Peak List |  |
|  | | | | | | | | | | | | | | | | | | | | | | | | | | | | | | | | |
|  | High |  | AEAGAGSATEFQFR |  | CID |  |  |  |  |  | 2.97 |  | 1.00 |  | 1 |  | SEQUEST (4) |  | 2 |  | 721.34314 |  | 5.75 |  | 1257 |  | 1257 |  | Image |  | Peak List |  |
|  | | | | | | | | | | | | | | | | | | | | | | | | | | | | | | | | |
|  | **P48643 - T-complex protein 1 subunit epsilon OS=Homo sapiens GN=CCT5 PE=1 SV=1 - [TCPE\_HUMAN]** | | | | | | | | | | | | | | | | | | | | | | | | | | | | | | |  |
|  | | | | | | | | | | | | | | | | | | | | | | | | | | | | | | | | |
|  | High |  | QMAEIAVNAVLTVADMER |  | CID |  |  |  | 23.63 |  |  |  | 1.00 |  | 1 |  | Mascot (2) |  | 3 |  | 654.33698 |  | 6.73 |  | 3339 |  | 3339 |  | Image |  | Peak List |  |
|  | | | | | | | | | | | | | | | | | | | | | | | | | | | | | | | | |
|  | High |  | QMAEIAVNAVLTVADMER |  | CID |  |  |  |  |  | 3.22 |  | 1.00 |  | 1 |  | SEQUEST (4) |  | 3 |  | 654.33618 |  | 5.51 |  | 3344 |  | 3344 |  | Image |  | Peak List |  |
|  | | | | | | | | | | | | | | | | | | | | | | | | | | | | | | | | |
|  | **P49773 - Histidine triad nucleotide-binding protein 1 OS=Homo sapiens GN=HINT1 PE=1 SV=2 - [HINT1\_HUMAN]** | | | | | | | | | | | | | | | | | | | | | | | | | | | | | | |  |
|  | | | | | | | | | | | | | | | | | | | | | | | | | | | | | | | | |
|  | High |  | KHISQISVAEDDDESLLGHLMIVGK |  | CID |  |  |  | 42.52 |  |  |  | 1.00 |  | 1 |  | Mascot (2) |  | 3 |  | 912.14362 |  | 2.98 |  | 1976 |  | 1976 |  | Image |  | Peak List |  |
|  | | | | | | | | | | | | | | | | | | | | | | | | | | | | | | | | |
|  | High |  | KHISQISVAEDDDESLLGHLMIVGK |  | CID |  |  |  |  |  | 4.02 |  | 1.00 |  | 1 |  | SEQUEST (4) |  | 3 |  | 912.14362 |  | 2.98 |  | 1976 |  | 1976 |  | Image |  | Peak List |  |
|  | | | | | | | | | | | | | | | | | | | | | | | | | | | | | | | | |
|  | **P49914 - 5-formyltetrahydrofolate cyclo-ligase OS=Homo sapiens GN=MTHFS PE=1 SV=2 - [MTHFS\_HUMAN]** | | | | | | | | | | | | | | | | | | | | | | | | | | | | | | |  |
|  | | | | | | | | | | | | | | | | | | | | | | | | | | | | | | | | |
|  | High |  | ISIFLSMQDEIETEEIIKDIFQR |  | CID |  |  |  | 31.56 |  |  |  | 1.00 |  | 1 |  | Mascot (2) |  | 3 |  | 933.15515 |  | 6.41 |  | 4039 |  | 4039 |  | Image |  | Peak List |  |
|  | | | | | | | | | | | | | | | | | | | | | | | | | | | | | | | | |
|  | High |  | ISIFLSMQDEIETEEIIKDIFQR |  | CID |  |  |  |  |  | 2.64 |  | 1.00 |  | 1 |  | SEQUEST (4) |  | 3 |  | 933.15515 |  | 6.41 |  | 4039 |  | 4039 |  | Image |  | Peak List |  |
|  | | | | | | | | | | | | | | | | | | | | | | | | | | | | | | | | |
|  | **P50053 - Ketohexokinase OS=Homo sapiens GN=KHK PE=1 SV=1 - [KHK\_HUMAN]** | | | | | | | | | | | | | | | | | | | | | | | | | | | | | | |  |
|  | | | | | | | | | | | | | | | | | | | | | | | | | | | | | | | | |
|  | High |  | VSVEVEKPREELFQLFGYGDVVFVSK |  | CID |  |  |  | 36.36 |  |  |  | 1.00 |  | 1 |  | Mascot (2) |  | 3 |  | 1000.86884 |  | 6.70 |  | 2863 |  | 2863 |  | Image |  | Peak List |  |
|  | | | | | | | | | | | | | | | | | | | | | | | | | | | | | | | | |
|  | High |  | VSVEVEKPREELFQLFGYGDVVFVSK |  | CID |  |  |  |  |  | 4.76 |  | 1.00 |  | 1 |  | SEQUEST (4) |  | 3 |  | 1000.86884 |  | 6.70 |  | 2863 |  | 2863 |  | Image |  | Peak List |  |
|  | | | | | | | | | | | | | | | | | | | | | | | | | | | | | | | | |
|  | **P50135 - Histamine N-methyltransferase OS=Homo sapiens GN=HNMT PE=1 SV=1 - [HNMT\_HUMAN]** | | | | | | | | | | | | | | | | | | | | | | | | | | | | | | |  |
|  | | | | | | | | | | | | | | | | | | | | | | | | | | | | | | | | |
|  | High |  | ILSIGGGAGEIDLQILSK |  | CID |  |  |  | 69.42 |  |  |  | 1.00 |  | 1 |  | Mascot (2) |  | 2 |  | 892.51770 |  | 6.52 |  | 2864 |  | 2864 |  | Image |  | Peak List |  |
|  | | | | | | | | | | | | | | | | | | | | | | | | | | | | | | | | |
|  | High |  | ILSIGGGAGEIDLQILSK |  | CID |  |  |  |  |  | 4.30 |  | 1.00 |  | 1 |  | SEQUEST (4) |  | 2 |  | 892.51715 |  | 5.90 |  | 2768 |  | 2768 |  | Image |  | Peak List |  |
|  | | | | | | | | | | | | | | | | | | | | | | | | | | | | | | | | |
|  | **P50225 - Sulfotransferase 1A1 OS=Homo sapiens GN=SULT1A1 PE=1 SV=3 - [ST1A1\_HUMAN]** | | | | | | | | | | | | | | | | | | | | | | | | | | | | | | |  |
|  | | | | | | | | | | | | | | | | | | | | | | | | | | | | | | | | |
|  | High |  | FMVGEVSYGSWYQHVQEWWELSR |  | CID |  |  |  | 44.52 |  |  |  | 1.00 |  | 1 |  | Mascot (2) |  | 3 |  | 968.45190 |  | 5.67 |  | 3301 |  | 3301 |  | Image |  | Peak List |  |
|  | | | | | | | | | | | | | | | | | | | | | | | | | | | | | | | | |
|  | High |  | FMVGEVSYGSWYQHVQEWWELSR |  | CID |  |  |  |  |  | 4.22 |  | 1.00 |  | 1 |  | SEQUEST (4) |  | 3 |  | 968.45190 |  | 5.67 |  | 3301 |  | 3301 |  | Image |  | Peak List |  |
|  | | | | | | | | | | | | | | | | | | | | | | | | | | | | | | | | |
|  | **P50395 - Rab GDP dissociation inhibitor beta OS=Homo sapiens GN=GDI2 PE=1 SV=2 - [GDIB\_HUMAN]** | | | | | | | | | | | | | | | | | | | | | | | | | | | | | | |  |
|  | | | | | | | | | | | | | | | | | | | | | | | | | | | | | | | | |
|  | High |  | YIAIVSTTVETK |  | CID |  |  |  | 28.80 |  |  |  | 1.00 |  | 1 |  | Mascot (2) |  | 2 |  | 662.87506 |  | 5.24 |  | 1381 |  | 1381 |  | Image |  | Peak List |  |
|  | | | | | | | | | | | | | | | | | | | | | | | | | | | | | | | | |
|  | High |  | YIAIVSTTVETK |  | CID |  |  |  |  |  | 2.72 |  | 1.00 |  | 1 |  | SEQUEST (4) |  | 2 |  | 662.87506 |  | 5.24 |  | 1381 |  | 1381 |  | Image |  | Peak List |  |
|  | | | | | | | | | | | | | | | | | | | | | | | | | | | | | | | | |
|  | **P50440 - Glycine amidinotransferase, mitochondrial OS=Homo sapiens GN=GATM PE=1 SV=1 - [GATM\_HUMAN]** | | | | | | | | | | | | | | | | | | | | | | | | | | | | | | |  |
|  | | | | | | | | | | | | | | | | | | | | | | | | | | | | | | | | |
|  | High |  | DILIVVGNEIIEAPMAWR |  | CID |  |  |  | 48.21 |  |  |  | 1.00 |  | 1 |  | Mascot (2) |  | 3 |  | 680.37610 |  | 6.97 |  | 3818 |  | 3818 |  | Image |  | Peak List |  |
|  | | | | | | | | | | | | | | | | | | | | | | | | | | | | | | | | |
|  | High |  | DILIVVGNEIIEAPMAWR |  | CID |  |  |  |  |  | 4.99 |  | 1.00 |  | 1 |  | SEQUEST (4) |  | 3 |  | 680.37610 |  | 6.97 |  | 3818 |  | 3818 |  | Image |  | Peak List |  |
|  | | | | | | | | | | | | | | | | | | | | | | | | | | | | | | | | |
|  | **P51570 - Galactokinase OS=Homo sapiens GN=GALK1 PE=1 SV=1 - [GALK1\_HUMAN]** | | | | | | | | | | | | | | | | | | | | | | | | | | | | | | |  |
|  | | | | | | | | | | | | | | | | | | | | | | | | | | | | | | | | |
|  | High |  | HIQEHYGGTATFYLSQAADGAK |  | CID |  |  |  | 27.15 |  |  |  | 1.00 |  | 1 |  | Mascot (2) |  | 3 |  | 789.04913 |  | 5.08 |  | 1191 |  | 1191 |  | Image |  | Peak List |  |
|  | | | | | | | | | | | | | | | | | | | | | | | | | | | | | | | | |
|  | High |  | HIQEHYGGTATFYLSQAADGAK |  | CID |  |  |  |  |  | 2.15 |  | 1.00 |  | 1 |  | SEQUEST (4) |  | 3 |  | 789.04913 |  | 5.08 |  | 1191 |  | 1191 |  | Image |  | Peak List |  |
|  | | | | | | | | | | | | | | | | | | | | | | | | | | | | | | | | |
|  | **P51687 - Sulfite oxidase, mitochondrial OS=Homo sapiens GN=SUOX PE=1 SV=2 - [SUOX\_HUMAN]** | | | | | | | | | | | | | | | | | | | | | | | | | | | | | | |  |
|  | | | | | | | | | | | | | | | | | | | | | | | | | | | | | | | | |
|  | High |  | DGETVESGEVTIK |  | CID |  |  |  | 26.16 |  |  |  | 1.00 |  | 1 |  | Mascot (2) |  | 2 |  | 682.33698 |  | 5.77 |  | 1043 |  | 1043 |  | Image |  | Peak List |  |
|  | | | | | | | | | | | | | | | | | | | | | | | | | | | | | | | | |
|  | High |  | DGETVESGEVTIK |  | CID |  |  |  |  |  | 1.70 |  | 1.00 |  | 1 |  | SEQUEST (4) |  | 2 |  | 682.33698 |  | 5.77 |  | 1043 |  | 1043 |  | Image |  | Peak List |  |
|  | | | | | | | | | | | | | | | | | | | | | | | | | | | | | | | | |
|  | **P52209 - 6-phosphogluconate dehydrogenase, decarboxylating OS=Homo sapiens GN=PGD PE=1 SV=3 - [6PGD\_HUMAN]** | | | | | | | | | | | | | | | | | | | | | | | | | | | | | | |  |
|  | | | | | | | | | | | | | | | | | | | | | | | | | | | | | | | | |
|  | High |  | GILFVGSGVSGGEEGAR |  | CID |  |  |  | 46.88 |  |  |  | 1.00 |  | 1 |  | Mascot (2) |  | 2 |  | 796.40906 |  | 2.06 |  | 1802 |  | 1802 |  | Image |  | Peak List |  |
|  | | | | | | | | | | | | | | | | | | | | | | | | | | | | | | | | |
|  | High |  | GILFVGSGVSGGEEGAR |  | CID |  |  |  |  |  | 3.47 |  | 1.00 |  | 1 |  | SEQUEST (4) |  | 2 |  | 796.40906 |  | 2.06 |  | 1802 |  | 1802 |  | Image |  | Peak List |  |
|  | | | | | | | | | | | | | | | | | | | | | | | | | | | | | | | | |
|  | **P54819 - Adenylate kinase 2, mitochondrial OS=Homo sapiens GN=AK2 PE=1 SV=2 - [KAD2\_HUMAN]** | | | | | | | | | | | | | | | | | | | | | | | | | | | | | | |  |
|  | | | | | | | | | | | | | | | | | | | | | | | | | | | | | | | | |
|  | High |  | LVSDEMVVELIEK |  | CID |  |  |  | 72.70 |  |  |  | 1.00 |  | 1 |  | Mascot (2) |  | 2 |  | 752.40637 |  | 5.26 |  | 2715 |  | 2715 |  | Image |  | Peak List |  |
|  | | | | | | | | | | | | | | | | | | | | | | | | | | | | | | | | |
|  | High |  | LVSDEMVVELIEK |  | CID |  |  |  |  |  | 3.43 |  | 1.00 |  | 1 |  | SEQUEST (4) |  | 2 |  | 752.40637 |  | 5.26 |  | 2715 |  | 2715 |  | Image |  | Peak List |  |
|  | | | | | | | | | | | | | | | | | | | | | | | | | | | | | | | | |
|  | **P55786 - Puromycin-sensitive aminopeptidase OS=Homo sapiens GN=NPEPPS PE=1 SV=2 - [PSA\_HUMAN]** | | | | | | | | | | | | | | | | | | | | | | | | | | | | | | |  |
|  | | | | | | | | | | | | | | | | | | | | | | | | | | | | | | | | |
|  | High |  | IDLIAIADFAAGAMENWGLVTYR |  | CID |  |  |  | 37.78 |  |  |  | 1.00 |  | 1 |  | Mascot (2) |  | 3 |  | 837.43744 |  | 9.09 |  | 5237 |  | 5237 |  | Image |  | Peak List |  |
|  | | | | | | | | | | | | | | | | | | | | | | | | | | | | | | | | |
|  | High |  | IDLIAIADFAAGAMENWGLVTYR |  | CID |  |  |  |  |  | 4.71 |  | 1.00 |  | 1 |  | SEQUEST (4) |  | 3 |  | 837.43744 |  | 9.09 |  | 5237 |  | 5237 |  | Image |  | Peak List |  |
|  | | | | | | | | | | | | | | | | | | | | | | | | | | | | | | | | |
|  | **P61513 - 60S ribosomal protein L37a OS=Homo sapiens GN=RPL37A PE=1 SV=2 - [RL37A\_HUMAN]** | | | | | | | | | | | | | | | | | | | | | | | | | | | | | | |  |
|  | | | | | | | | | | | | | | | | | | | | | | | | | | | | | | | | |
|  | High |  | TVAGGAWTYNTTSAVTVK |  | CID |  |  |  | 47.23 |  |  |  | 1.00 |  | 1 |  | Mascot (2) |  | 2 |  | 913.97345 |  | 6.16 |  | 1447 |  | 1447 |  | Image |  | Peak List |  |
|  | | | | | | | | | | | | | | | | | | | | | | | | | | | | | | | | |
|  | High |  | TVAGGAWTYNTTSAVTVK |  | CID |  |  |  |  |  | 3.16 |  | 1.00 |  | 1 |  | SEQUEST (4) |  | 2 |  | 913.97345 |  | 6.16 |  | 1447 |  | 1447 |  | Image |  | Peak List |  |
|  | | | | | | | | | | | | | | | | | | | | | | | | | | | | | | | | |
|  | **P61604 - 10 kDa heat shock protein, mitochondrial OS=Homo sapiens GN=HSPE1 PE=1 SV=2 - [CH10\_HUMAN]** | | | | | | | | | | | | | | | | | | | | | | | | | | | | | | |  |
|  | | | | | | | | | | | | | | | | | | | | | | | | | | | | | | | | |
|  | High |  | SQGKVLQATVVAVGSGSKGK |  | CID |  |  |  | 30.74 |  |  |  | 1.00 |  | 1 |  | Mascot (2) |  | 3 |  | 634.36932 |  | 6.24 |  | 914 |  | 914 |  | Image |  | Peak List |  |
|  | | | | | | | | | | | | | | | | | | | | | | | | | | | | | | | | |
|  | High |  | SQGKVLQATVVAVGSGSKGK |  | CID |  |  |  |  |  | 2.58 |  | 1.00 |  | 1 |  | SEQUEST (4) |  | 3 |  | 634.36932 |  | 6.24 |  | 914 |  | 914 |  | Image |  | Peak List |  |
|  | | | | | | | | | | | | | | | | | | | | | | | | | | | | | | | | |
|  | **P62244 - 40S ribosomal protein S15a OS=Homo sapiens GN=RPS15A PE=1 SV=2 - [RS15A\_HUMAN]** | | | | | | | | | | | | | | | | | | | | | | | | | | | | | | |  |
|  | | | | | | | | | | | | | | | | | | | | | | | | | | | | | | | | |
|  | High |  | QFGFIVLTTSAGIMDHEEAR |  | CID |  |  |  | 45.51 |  |  |  | 1.00 |  | 1 |  | Mascot (2) |  | 3 |  | 741.37268 |  | 5.56 |  | 2565 |  | 2565 |  | Image |  | Peak List |  |
|  | | | | | | | | | | | | | | | | | | | | | | | | | | | | | | | | |
|  | High |  | QFGFIVLTTSAGIMDHEEAR |  | CID |  |  |  |  |  | 3.37 |  | 1.00 |  | 1 |  | SEQUEST (4) |  | 3 |  | 741.37268 |  | 5.56 |  | 2565 |  | 2565 |  | Image |  | Peak List |  |
|  | | | | | | | | | | | | | | | | | | | | | | | | | | | | | | | | |
|  | **P62701 - 40S ribosomal protein S4, X isoform OS=Homo sapiens GN=RPS4X PE=1 SV=2 - [RS4X\_HUMAN]** | | | | | | | | | | | | | | | | | | | | | | | | | | | | | | |  |
|  | | | | | | | | | | | | | | | | | | | | | | | | | | | | | | | | |
|  | High |  | VNDTIQIDLETGK |  | CID |  |  |  | 41.56 |  |  |  | 1.00 |  | 1 |  | Mascot (2) |  | 2 |  | 723.38049 |  | 3.75 |  | 1565 |  | 1565 |  | Image |  | Peak List |  |
|  | | | | | | | | | | | | | | | | | | | | | | | | | | | | | | | | |
|  | High |  | VNDTIQIDLETGK |  | CID |  |  |  |  |  | 3.28 |  | 1.00 |  | 1 |  | SEQUEST (4) |  | 2 |  | 723.38049 |  | 3.75 |  | 1565 |  | 1565 |  | Image |  | Peak List |  |
|  | | | | | | | | | | | | | | | | | | | | | | | | | | | | | | | | |
|  | **P62820 - Ras-related protein Rab-1A OS=Homo sapiens GN=RAB1A PE=1 SV=3 - [RAB1A\_HUMAN]** | | | | | | | | | | | | | | | | | | | | | | | | | | | | | | |  |
|  | | | | | | | | | | | | | | | | | | | | | | | | | | | | | | | | |
|  | High |  | GAHGIIVVYDVTDQESFNNVK |  | CID |  |  |  | 22.61 |  |  |  | 1.00 |  | 1 |  | Mascot (2) |  | 3 |  | 769.05621 |  | 3.51 |  | 1806 |  | 1806 |  | Image |  | Peak List |  |
|  | | | | | | | | | | | | | | | | | | | | | | | | | | | | | | | | |
|  | High |  | GAHGIIVVYDVTDQESFNNVK |  | CID |  |  |  |  |  | 3.16 |  | 1.00 |  | 1 |  | SEQUEST (4) |  | 3 |  | 769.05621 |  | 3.51 |  | 1806 |  | 1806 |  | Image |  | Peak List |  |
|  | | | | | | | | | | | | | | | | | | | | | | | | | | | | | | | | |
|  | **P62937 - Peptidyl-prolyl cis-trans isomerase A OS=Homo sapiens GN=PPIA PE=1 SV=2 - [PPIA\_HUMAN]** | | | | | | | | | | | | | | | | | | | | | | | | | | | | | | |  |
|  | | | | | | | | | | | | | | | | | | | | | | | | | | | | | | | | |
|  | High |  | VNPTVFFDIAVDGEPLGR |  | CID |  |  |  | 21.76 |  |  |  | 1.00 |  | 1 |  | Mascot (2) |  | 2 |  | 973.51129 |  | 6.88 |  | 3331 |  | 3331 |  | Image |  | Peak List |  |
|  | | | | | | | | | | | | | | | | | | | | | | | | | | | | | | | | |
|  | **Q06323 - Proteasome activator complex subunit 1 OS=Homo sapiens GN=PSME1 PE=1 SV=1 - [PSME1\_HUMAN]** | | | | | | | | | | | | | | | | | | | | | | | | | | | | | | |  |
|  | | | | | | | | | | | | | | | | | | | | | | | | | | | | | | | | |
|  | High |  | IEDGNNFGVAVQEK |  | CID |  |  |  | 28.06 |  |  |  | 1.00 |  | 1 |  | Mascot (2) |  | 2 |  | 760.37732 |  | 5.64 |  | 1128 |  | 1128 |  | Image |  | Peak List |  |
|  | | | | | | | | | | | | | | | | | | | | | | | | | | | | | | | | |
|  | High |  | IEDGNNFGVAVQEK |  | CID |  |  |  |  |  | 2.40 |  | 1.00 |  | 1 |  | SEQUEST (4) |  | 2 |  | 760.37732 |  | 5.64 |  | 1128 |  | 1128 |  | Image |  | Peak List |  |
|  | | | | | | | | | | | | | | | | | | | | | | | | | | | | | | | | |
|  | **Q06830 - Peroxiredoxin-1 OS=Homo sapiens GN=PRDX1 PE=1 SV=1 - [PRDX1\_HUMAN]** | | | | | | | | | | | | | | | | | | | | | | | | | | | | | | |  |
|  | | | | | | | | | | | | | | | | | | | | | | | | | | | | | | | | |
|  | High |  | LNCQVIGASVDSHFCHLAWVNTPK |  | CID |  |  |  | 29.68 |  |  |  | 1.00 |  | 1 |  | Mascot (2) |  | 4 |  | 660.57935 |  | 3.68 |  | 2273 |  | 2273 |  | Image |  | Peak List |  |
|  | | | | | | | | | | | | | | | | | | | | | | | | | | | | | | | | |
|  | High |  | LNCQVIGASVDSHFCHLAWVNTPK |  | CID |  |  |  |  |  | 3.05 |  | 1.00 |  | 1 |  | SEQUEST (4) |  | 4 |  | 660.57935 |  | 3.68 |  | 2273 |  | 2273 |  | Image |  | Peak List |  |
|  | | | | | | | | | | | | | | | | | | | | | | | | | | | | | | | | |
|  | **Q14558 - Phosphoribosyl pyrophosphate synthase-associated protein 1 OS=Homo sapiens GN=PRPSAP1 PE=1 SV=2 - [KPRA\_HUMAN]** | | | | | | | | | | | | | | | | | | | | | | | | | | | | | | |  |
|  | | | | | | | | | | | | | | | | | | | | | | | | | | | | | | | | |
|  | High |  | IAIIVDDIIDDVESFVAAAEILK |  | CID |  |  |  | 36.47 |  |  |  | 1.00 |  | 1 |  | Mascot (2) |  | 3 |  | 824.79364 |  | 7.23 |  | 5912 |  | 5912 |  | Image |  | Peak List |  |
|  | | | | | | | | | | | | | | | | | | | | | | | | | | | | | | | | |
|  | High |  | IAIIVDDIIDDVESFVAAAEILK |  | CID |  |  |  |  |  | 4.47 |  | 1.00 |  | 1 |  | SEQUEST (4) |  | 3 |  | 824.79358 |  | 7.16 |  | 5940 |  | 5940 |  | Image |  | Peak List |  |
|  | | | | | | | | | | | | | | | | | | | | | | | | | | | | | | | | |
|  | **Q15631 - Translin OS=Homo sapiens GN=TSN PE=1 SV=1 - [TSN\_HUMAN]** | | | | | | | | | | | | | | | | | | | | | | | | | | | | | | |  |
|  | | | | | | | | | | | | | | | | | | | | | | | | | | | | | | | | |
|  | High |  | GFHLDVEDYLSGVLILASELSR |  | CID |  |  |  | 22.74 |  |  |  | 1.00 |  | 1 |  | Mascot (2) |  | 3 |  | 811.76819 |  | 9.82 |  | 4672 |  | 4672 |  | Image |  | Peak List |  |
|  | | | | | | | | | | | | | | | | | | | | | | | | | | | | | | | | |
|  | High |  | GFHLDVEDYLSGVLILASELSR |  | CID |  |  |  |  |  | 2.87 |  | 1.00 |  | 1 |  | SEQUEST (4) |  | 3 |  | 811.76660 |  | 7.86 |  | 4706 |  | 4706 |  | Image |  | Peak List |  |
|  | | | | | | | | | | | | | | | | | | | | | | | | | | | | | | | | |
|  | **Q16762 - Thiosulfate sulfurtransferase OS=Homo sapiens GN=TST PE=1 SV=4 - [THTR\_HUMAN]** | | | | | | | | | | | | | | | | | | | | | | | | | | | | | | |  |
|  | | | | | | | | | | | | | | | | | | | | | | | | | | | | | | | | |
|  | High |  | LGISNHTHVVVYDGEHLGSFYAPR |  | CID |  |  |  | 27.63 |  |  |  | 1.00 |  | 1 |  | Mascot (2) |  | 3 |  | 890.11847 |  | 5.29 |  | 1371 |  | 1371 |  | Image |  | Peak List |  |
|  | | | | | | | | | | | | | | | | | | | | | | | | | | | | | | | | |
|  | High |  | LGISNHTHVVVYDGEHLGSFYAPR |  | CID |  |  |  |  |  | 4.66 |  | 1.00 |  | 1 |  | SEQUEST (4) |  | 3 |  | 890.11847 |  | 5.29 |  | 1371 |  | 1371 |  | Image |  | Peak List |  |
|  | | | | | | | | | | | | | | | | | | | | | | | | | | | | | | | | |
|  | **Q6NVY1 - 3-hydroxyisobutyryl-CoA hydrolase, mitochondrial OS=Homo sapiens GN=HIBCH PE=1 SV=2 - [HIBCH\_HUMAN]** | | | | | | | | | | | | | | | | | | | | | | | | | | | | | | |  |
|  | | | | | | | | | | | | | | | | | | | | | | | | | | | | | | | | |
|  | High |  | TLQEVLTMEYR |  | CID |  |  |  | 39.99 |  |  |  | 1.00 |  | 1 |  | Mascot (2) |  | 2 |  | 691.85492 |  | 2.95 |  | 2002 |  | 2002 |  | Image |  | Peak List |  |
|  | | | | | | | | | | | | | | | | | | | | | | | | | | | | | | | | |
|  | High |  | TLQEVLTMEYR |  | CID |  |  |  |  |  | 2.57 |  | 1.00 |  | 1 |  | SEQUEST (4) |  | 2 |  | 691.85492 |  | 2.95 |  | 2002 |  | 2002 |  | Image |  | Peak List |  |
|  | | | | | | | | | | | | | | | | | | | | | | | | | | | | | | | | |
|  | **Q6P2I3 - Fumarylacetoacetate hydrolase domain-containing protein 2B OS=Homo sapiens GN=FAHD2B PE=2 SV=1 - [FAH2B\_HUMAN]** | | | | | | | | | | | | | | | | | | | | | | | | | | | | | | |  |
|  | | | | | | | | | | | | | | | | | | | | | | | | | | | | | | | | |
|  | High |  | TMTQFLEQGEATLSVAR |  | CID |  |  |  | 26.82 |  |  |  | 1.00 |  | 1 |  | Mascot (2) |  | 3 |  | 627.98669 |  | 4.22 |  | 2504 |  | 2504 |  | Image |  | Peak List |  |
|  | | | | | | | | | | | | | | | | | | | | | | | | | | | | | | | | |
|  | High |  | TMTQFLEQGEATLSVAR |  | CID |  |  |  |  |  | 2.23 |  | 1.00 |  | 1 |  | SEQUEST (4) |  | 3 |  | 627.98669 |  | 4.22 |  | 2504 |  | 2504 |  | Image |  | Peak List |  |
|  | | | | | | | | | | | | | | | | | | | | | | | | | | | | | | | | |
|  | **Q6P587 - Acylpyruvase FAHD1, mitochondrial OS=Homo sapiens GN=FAHD1 PE=1 SV=2 - [FAHD1\_HUMAN]** | | | | | | | | | | | | | | | | | | | | | | | | | | | | | | |  |
|  | | | | | | | | | | | | | | | | | | | | | | | | | | | | | | | | |
|  | High |  | NLHHELELGVVMGK |  | CID |  |  |  | 28.41 |  |  |  | 1.00 |  | 1 |  | Mascot (2) |  | 3 |  | 525.95117 |  | 4.89 |  | 1146 |  | 1146 |  | Image |  | Peak List |  |
|  | | | | | | | | | | | | | | | | | | | | | | | | | | | | | | | | |
|  | High |  | NLHHELELGVVMGK |  | CID |  |  |  |  |  | 2.63 |  | 1.00 |  | 1 |  | SEQUEST (4) |  | 3 |  | 525.95117 |  | 4.89 |  | 1146 |  | 1146 |  | Image |  | Peak List |  |
|  | | | | | | | | | | | | | | | | | | | | | | | | | | | | | | | | |
|  | **Q6PKH6 - Dehydrogenase/reductase SDR family member 4-like 2 OS=Homo sapiens GN=DHRS4L2 PE=2 SV=1 - [DR4L2\_HUMAN]** | | | | | | | | | | | | | | | | | | | | | | | | | | | | | | |  |
|  | | | | | | | | | | | | | | | | | | | | | | | | | | | | | | | | |
|  | High |  | VALVTASTDGIGFAIAR |  | CID |  |  |  | 50.57 |  |  |  | 1.00 |  | 1 |  | Mascot (2) |  | 2 |  | 831.46906 |  | 5.20 |  | 2400 |  | 2400 |  | Image |  | Peak List |  |
|  | | | | | | | | | | | | | | | | | | | | | | | | | | | | | | | | |
|  | High |  | VALVTASTDGIGFAIAR |  | CID |  |  |  |  |  | 4.78 |  | 1.00 |  | 1 |  | SEQUEST (4) |  | 2 |  | 831.46906 |  | 5.20 |  | 2400 |  | 2400 |  | Image |  | Peak List |  |
|  | | | | | | | | | | | | | | | | | | | | | | | | | | | | | | | | |
|  | **Q86TX2 - Acyl-coenzyme A thioesterase 1 OS=Homo sapiens GN=ACOT1 PE=1 SV=1 - [ACOT1\_HUMAN]** | | | | | | | | | | | | | | | | | | | | | | | | | | | | | | |  |
|  | | | | | | | | | | | | | | | | | | | | | | | | | | | | | | | | |
|  | High |  | GFAVMALAYYNYEDLPK |  | CID |  |  |  | 29.73 |  |  |  | 1.00 |  | 1 |  | Mascot (2) |  | 3 |  | 655.65698 |  | 5.12 |  | 3050 |  | 3050 |  | Image |  | Peak List |  |
|  | | | | | | | | | | | | | | | | | | | | | | | | | | | | | | | | |
|  | High |  | GFAVMALAYYNYEDLPK |  | CID |  |  |  |  |  | 2.60 |  | 1.00 |  | 1 |  | SEQUEST (4) |  | 3 |  | 655.65698 |  | 5.12 |  | 3050 |  | 3050 |  | Image |  | Peak List |  |
|  | | | | | | | | | | | | | | | | | | | | | | | | | | | | | | | | |
|  | **Q8N0X4 - Citrate lyase subunit beta-like protein, mitochondrial OS=Homo sapiens GN=CLYBL PE=2 SV=2 - [CLYBL\_HUMAN]** | | | | | | | | | | | | | | | | | | | | | | | | | | | | | | |  |
|  | | | | | | | | | | | | | | | | | | | | | | | | | | | | | | | | |
|  | High |  | VGPQVGLFLDAVVFGGEDFR |  | CID |  |  |  | 31.32 |  |  |  | 1.00 |  | 1 |  | Mascot (2) |  | 3 |  | 708.04193 |  | 6.76 |  | 3954 |  | 3954 |  | Image |  | Peak List |  |
|  | | | | | | | | | | | | | | | | | | | | | | | | | | | | | | | | |
|  | High |  | VGPQVGLFLDAVVFGGEDFR |  | CID |  |  |  |  |  | 4.30 |  | 1.00 |  | 1 |  | SEQUEST (4) |  | 3 |  | 708.04193 |  | 6.76 |  | 3954 |  | 3954 |  | Image |  | Peak List |  |
|  | | | | | | | | | | | | | | | | | | | | | | | | | | | | | | | | |
|  | **Q8WUM4 - Programmed cell death 6-interacting protein OS=Homo sapiens GN=PDCD6IP PE=1 SV=1 - [PDC6I\_HUMAN]** | | | | | | | | | | | | | | | | | | | | | | | | | | | | | | |  |
|  | | | | | | | | | | | | | | | | | | | | | | | | | | | | | | | | |
|  | High |  | EPTVDISPDTVGTLSLIMLAQAQEVFFLK |  | CID |  |  |  | 33.31 |  |  |  | 1.00 |  | 1 |  | Mascot (2) |  | 3 |  | 1054.90002 |  | 6.66 |  | 5276 |  | 5276 |  | Image |  | Peak List |  |
|  | | | | | | | | | | | | | | | | | | | | | | | | | | | | | | | | |
|  | High |  | EPTVDISPDTVGTLSLIMLAQAQEVFFLK |  | CID |  |  |  |  |  | 3.83 |  | 1.00 |  | 1 |  | SEQUEST (4) |  | 3 |  | 1054.90002 |  | 6.66 |  | 5276 |  | 5276 |  | Image |  | Peak List |  |
|  | | | | | | | | | | | | | | | | | | | | | | | | | | | | | | | | |
|  | **Q96EK6 - Glucosamine 6-phosphate N-acetyltransferase OS=Homo sapiens GN=GNPNAT1 PE=1 SV=1 - [GNA1\_HUMAN]** | | | | | | | | | | | | | | | | | | | | | | | | | | | | | | |  |
|  | | | | | | | | | | | | | | | | | | | | | | | | | | | | | | | | |
|  | High |  | SGDYYVTVVEDVTLGQIVATATLIIEHK |  | CID |  |  |  | 32.85 |  |  |  | 1.00 |  | 1 |  | Mascot (2) |  | 4 |  | 759.40997 |  | 6.47 |  | 4832 |  | 4832 |  | Image |  | Peak List |  |
|  | | | | | | | | | | | | | | | | | | | | | | | | | | | | | | | | |
|  | High |  | SGDYYVTVVEDVTLGQIVATATLIIEHK |  | CID |  |  |  |  |  | 2.30 |  | 1.00 |  | 1 |  | SEQUEST (4) |  | 4 |  | 759.40997 |  | 6.47 |  | 4832 |  | 4832 |  | Image |  | Peak List |  |
|  | | | | | | | | | | | | | | | | | | | | | | | | | | | | | | | | |
|  | **Q96HY7 - Probable 2-oxoglutarate dehydrogenase E1 component DHKTD1, mitochondrial OS=Homo sapiens GN=DHTKD1 PE=1 SV=2 - [DHTK1\_HUMAN]** | | | | | | | | | | | | | | | | | | | | | | | | | | | | | | |  |
|  | | | | | | | | | | | | | | | | | | | | | | | | | | | | | | | | |
|  | High |  | IGGSVHLIVNNQLGYTTPAER |  | CID |  |  |  | 34.43 |  |  |  | 1.00 |  | 1 |  | Mascot (2) |  | 3 |  | 747.07001 |  | 5.55 |  | 1466 |  | 1466 |  | Image |  | Peak List |  |
|  | | | | | | | | | | | | | | | | | | | | | | | | | | | | | | | | |
|  | High |  | IGGSVHLIVNNQLGYTTPAER |  | CID |  |  |  |  |  | 2.55 |  | 1.00 |  | 1 |  | SEQUEST (4) |  | 3 |  | 747.06885 |  | 4.00 |  | 1589 |  | 1589 |  | Image |  | Peak List |  |
|  | | | | | | | | | | | | | | | | | | | | | | | | | | | | | | | | |
|  | **Q96I99 - Succinyl-CoA ligase [GDP-forming] subunit beta, mitochondrial OS=Homo sapiens GN=SUCLG2 PE=1 SV=2 - [SUCB2\_HUMAN]** | | | | | | | | | | | | | | | | | | | | | | | | | | | | | | |  |
|  | | | | | | | | | | | | | | | | | | | | | | | | | | | | | | | | |
|  | High |  | VEAILVNIFGGIVNCAIIANGITK |  | CID |  |  |  | 26.81 |  |  |  | 1.00 |  | 1 |  | Mascot (2) |  | 3 |  | 814.80402 |  | 7.57 |  | 4748 |  | 4748 |  | Image |  | Peak List |  |
|  | | | | | | | | | | | | | | | | | | | | | | | | | | | | | | | | |
|  | High |  | VEAILVNIFGGIVNCAIIANGITK |  | CID |  |  |  |  |  | 4.14 |  | 1.00 |  | 1 |  | SEQUEST (4) |  | 3 |  | 814.80402 |  | 7.57 |  | 4748 |  | 4748 |  | Image |  | Peak List |  |
|  | | | | | | | | | | | | | | | | | | | | | | | | | | | | | | | | |
|  | **Q96IU4 - Alpha/beta hydrolase domain-containing protein 14B OS=Homo sapiens GN=ABHD14B PE=1 SV=1 - [ABHEB\_HUMAN]** | | | | | | | | | | | | | | | | | | | | | | | | | | | | | | |  |
|  | | | | | | | | | | | | | | | | | | | | | | | | | | | | | | | | |
|  | High |  | EGTIQVQGQALFFR |  | CID |  |  |  | 40.64 |  |  |  | 1.00 |  | 1 |  | Mascot (2) |  | 2 |  | 797.42535 |  | 3.13 |  | 2134 |  | 2134 |  | Image |  | Peak List |  |
|  | | | | | | | | | | | | | | | | | | | | | | | | | | | | | | | | |
|  | High |  | EGTIQVQGQALFFR |  | CID |  |  |  |  |  | 3.66 |  | 1.00 |  | 1 |  | SEQUEST (4) |  | 2 |  | 797.42535 |  | 3.13 |  | 2134 |  | 2134 |  | Image |  | Peak List |  |
|  | | | | | | | | | | | | | | | | | | | | | | | | | | | | | | | | |
|  | **Q96KP4 - Cytosolic non-specific dipeptidase OS=Homo sapiens GN=CNDP2 PE=1 SV=2 - [CNDP2\_HUMAN]** | | | | | | | | | | | | | | | | | | | | | | | | | | | | | | |  |
|  | | | | | | | | | | | | | | | | | | | | | | | | | | | | | | | | |
|  | High |  | LYDDIDFDIEEFAK |  | CID |  |  |  | 46.35 |  |  |  | 1.00 |  | 1 |  | Mascot (2) |  | 2 |  | 866.90747 |  | 7.13 |  | 3066 |  | 3066 |  | Image |  | Peak List |  |
|  | | | | | | | | | | | | | | | | | | | | | | | | | | | | | | | | |
|  | High |  | LYDDIDFDIEEFAK |  | CID |  |  |  |  |  | 3.96 |  | 1.00 |  | 1 |  | SEQUEST (4) |  | 2 |  | 866.90747 |  | 7.13 |  | 3066 |  | 3066 |  | Image |  | Peak List |  |
|  | | | | | | | | | | | | | | | | | | | | | | | | | | | | | | | | |
|  | **Q96N76 - Urocanate hydratase OS=Homo sapiens GN=UROC1 PE=1 SV=1 - [HUTU\_HUMAN]** | | | | | | | | | | | | | | | | | | | | | | | | | | | | | | |  |
|  | | | | | | | | | | | | | | | | | | | | | | | | | | | | | | | | |
|  | High |  | GATWVALHNGGGVGWGEVINGGFGLVLDGTPEAEGR |  | CID |  |  |  | 29.29 |  |  |  | 1.00 |  | 1 |  | Mascot (2) |  | 3 |  | 1183.92871 |  | 5.75 |  | 3203 |  | 3203 |  | Image |  | Peak List |  |
|  | | | | | | | | | | | | | | | | | | | | | | | | | | | | | | | | |
|  | High |  | GATWVALHNGGGVGWGEVINGGFGLVLDGTPEAEGR |  | CID |  |  |  |  |  | 3.25 |  | 1.00 |  | 1 |  | SEQUEST (4) |  | 3 |  | 1183.92871 |  | 5.75 |  | 3203 |  | 3203 |  | Image |  | Peak List |  |
|  | | | | | | | | | | | | | | | | | | | | | | | | | | | | | | | | |
|  | **Q96PE7 - Methylmalonyl-CoA epimerase, mitochondrial OS=Homo sapiens GN=MCEE PE=1 SV=1 - [MCEE\_HUMAN]** | | | | | | | | | | | | | | | | | | | | | | | | | | | | | | |  |
|  | | | | | | | | | | | | | | | | | | | | | | | | | | | | | | | | |
|  | High |  | AGGMHHICIEVDNINAAVMDLK |  | CID |  |  |  | 42.88 |  |  |  | 1.00 |  | 1 |  | Mascot (2) |  | 3 |  | 784.38519 |  | 4.43 |  | 2032 |  | 2032 |  | Image |  | Peak List |  |
|  | | | | | | | | | | | | | | | | | | | | | | | | | | | | | | | | |
|  | High |  | AGGMHHICIEVDNINAAVMDLK |  | CID |  |  |  |  |  | 3.71 |  | 1.00 |  | 1 |  | SEQUEST (4) |  | 3 |  | 784.38519 |  | 4.43 |  | 2032 |  | 2032 |  | Image |  | Peak List |  |
|  | | | | | | | | | | | | | | | | | | | | | | | | | | | | | | | | |
|  | **Q9BSH4 - Translational activator of cytochrome c oxidase 1 OS=Homo sapiens GN=TACO1 PE=1 SV=1 - [TACO1\_HUMAN]** | | | | | | | | | | | | | | | | | | | | | | | | | | | | | | |  |
|  | | | | | | | | | | | | | | | | | | | | | | | | | | | | | | | | |
|  | High |  | GVIVVEVEDR |  | CID |  |  |  | 32.43 |  |  |  | 1.00 |  | 1 |  | Mascot (2) |  | 2 |  | 557.81171 |  | 5.21 |  | 1290 |  | 1290 |  | Image |  | Peak List |  |
|  | | | | | | | | | | | | | | | | | | | | | | | | | | | | | | | | |
|  | High |  | GVIVVEVEDR |  | CID |  |  |  |  |  | 2.37 |  | 1.00 |  | 1 |  | SEQUEST (4) |  | 2 |  | 557.81171 |  | 5.21 |  | 1290 |  | 1290 |  | Image |  | Peak List |  |
|  | | | | | | | | | | | | | | | | | | | | | | | | | | | | | | | | |
|  | **Q9BWD1 - Acetyl-CoA acetyltransferase, cytosolic OS=Homo sapiens GN=ACAT2 PE=1 SV=2 - [THIC\_HUMAN]** | | | | | | | | | | | | | | | | | | | | | | | | | | | | | | |  |
|  | | | | | | | | | | | | | | | | | | | | | | | | | | | | | | | | |
|  | High |  | AGWSLEDVDIFEINEAFAAVSAAIVK |  | CID |  |  |  | 61.62 |  |  |  | 1.00 |  | 1 |  | Mascot (2) |  | 3 |  | 922.47998 |  | 7.95 |  | 4883 |  | 4883 |  | Image |  | Peak List |  |
|  | | | | | | | | | | | | | | | | | | | | | | | | | | | | | | | | |
|  | High |  | AGWSLEDVDIFEINEAFAAVSAAIVK |  | CID |  |  |  |  |  | 5.49 |  | 1.00 |  | 1 |  | SEQUEST (4) |  | 3 |  | 922.47998 |  | 7.95 |  | 4883 |  | 4883 |  | Image |  | Peak List |  |
|  | | | | | | | | | | | | | | | | | | | | | | | | | | | | | | | | |
|  | **Q9BY49 - Peroxisomal trans-2-enoyl-CoA reductase OS=Homo sapiens GN=PECR PE=1 SV=2 - [PECR\_HUMAN]** | | | | | | | | | | | | | | | | | | | | | | | | | | | | | | |  |
|  | | | | | | | | | | | | | | | | | | | | | | | | | | | | | | | | |
|  | High |  | ELLELGSNVVIASR |  | CID |  |  |  | 25.43 |  |  |  | 1.00 |  | 1 |  | Mascot (2) |  | 2 |  | 750.42804 |  | 3.96 |  | 2190 |  | 2190 |  | Image |  | Peak List |  |
|  | | | | | | | | | | | | | | | | | | | | | | | | | | | | | | | | |
|  | High |  | ELLELGSNVVIASR |  | CID |  |  |  |  |  | 3.05 |  | 1.00 |  | 1 |  | SEQUEST (4) |  | 2 |  | 750.42804 |  | 3.96 |  | 2190 |  | 2190 |  | Image |  | Peak List |  |
|  | | | | | | | | | | | | | | | | | | | | | | | | | | | | | | | | |
|  | **Q9H2M3 - S-methylmethionine--homocysteine S-methyltransferase BHMT2 OS=Homo sapiens GN=BHMT2 PE=1 SV=1 - [BHMT2\_HUMAN]** | | | | | | | | | | | | | | | | | | | | | | | | | | | | | | |  |
|  | | | | | | | | | | | | | | | | | | | | | | | | | | | | | | | | |
|  | High |  | NVDFLIAEYFEHVEEAVWAVEVLK |  | CID |  |  |  | 37.20 |  |  |  | 1.00 |  | 1 |  | Mascot (2) |  | 3 |  | 950.49219 |  | 7.81 |  | 4573 |  | 4573 |  | Image |  | Peak List |  |
|  | | | | | | | | | | | | | | | | | | | | | | | | | | | | | | | | |
|  | High |  | NVDFLIAEYFEHVEEAVWAVEVLK |  | CID |  |  |  |  |  | 4.05 |  | 1.00 |  | 1 |  | SEQUEST (4) |  | 3 |  | 950.49219 |  | 7.81 |  | 4573 |  | 4573 |  | Image |  | Peak List |  |
|  | | | | | | | | | | | | | | | | | | | | | | | | | | | | | | | | |
|  | **Q9P2E9 - Ribosome-binding protein 1 OS=Homo sapiens GN=RRBP1 PE=1 SV=4 - [RRBP1\_HUMAN]** | | | | | | | | | | | | | | | | | | | | | | | | | | | | | | |  |
|  | | | | | | | | | | | | | | | | | | | | | | | | | | | | | | | | |
|  | High |  | LTAEFEEAQTSACR |  | CID |  |  |  | 38.79 |  |  |  | 1.00 |  | 1 |  | Mascot (2) |  | 2 |  | 778.36096 |  | 5.71 |  | 1155 |  | 1155 |  | Image |  | Peak List |  |
|  | | | | | | | | | | | | | | | | | | | | | | | | | | | | | | | | |
|  | High |  | LTAEFEEAQTSACR |  | CID |  |  |  |  |  | 2.58 |  | 1.00 |  | 1 |  | SEQUEST (4) |  | 2 |  | 778.36096 |  | 5.71 |  | 1155 |  | 1155 |  | Image |  | Peak List |  |
|  | | | | | | | | | | | | | | | | | | | | | | | | | | | | | | | | |
|  | **Q9UJM8 - Hydroxyacid oxidase 1 OS=Homo sapiens GN=HAO1 PE=1 SV=1 - [HAOX1\_HUMAN]** | | | | | | | | | | | | | | | | | | | | | | | | | | | | | | |  |
|  | | | | | | | | | | | | | | | | | | | | | | | | | | | | | | | | |
|  | High |  | VEVFLDGGVR |  | CID |  |  |  | 29.97 |  |  |  | 1.00 |  | 1 |  | Mascot (2) |  | 2 |  | 545.80048 |  | 4.10 |  | 1621 |  | 1621 |  | Image |  | Peak List |  |
|  | | | | | | | | | | | | | | | | | | | | | | | | | | | | | | | | |
|  | High |  | VEVFLDGGVR |  | CID |  |  |  |  |  | 2.78 |  | 1.00 |  | 1 |  | SEQUEST (4) |  | 2 |  | 545.80048 |  | 4.10 |  | 1621 |  | 1621 |  | Image |  | Peak List |  |
|  | | | | | | | | | | | | | | | | | | | | | | | | | | | | | | | | |
|  | **Q9UL46 - Proteasome activator complex subunit 2 OS=Homo sapiens GN=PSME2 PE=1 SV=4 - [PSME2\_HUMAN]** | | | | | | | | | | | | | | | | | | | | | | | | | | | | | | |  |
|  | | | | | | | | | | | | | | | | | | | | | | | | | | | | | | | | |
|  | High |  | IEDGNDFGVAIQEK |  | CID |  |  |  | 32.95 |  |  |  | 1.00 |  | 1 |  | Mascot (2) |  | 2 |  | 767.87683 |  | 5.16 |  | 1416 |  | 1416 |  | Image |  | Peak List |  |
|  | | | | | | | | | | | | | | | | | | | | | | | | | | | | | | | | |
|  | High |  | IEDGNDFGVAIQEK |  | CID |  |  |  |  |  | 2.99 |  | 1.00 |  | 1 |  | SEQUEST (4) |  | 2 |  | 767.87744 |  | 5.96 |  | 1410 |  | 1410 |  | Image |  | Peak List |  |
|  | | | | | | | | | | | | | | | | | | | | | | | | | | | | | | | | |
|  | **Q9Y281 - Cofilin-2 OS=Homo sapiens GN=CFL2 PE=1 SV=1 - [COF2\_HUMAN]** | | | | | | | | | | | | | | | | | | | | | | | | | | | | | | |  |
|  | | | | | | | | | | | | | | | | | | | | | | | | | | | | | | | | |
|  | High |  | LGGNVVVSLEGKPL |  | CID |  |  |  | 48.73 |  |  |  | 1.00 |  | 1 |  | Mascot (2) |  | 2 |  | 691.40802 |  | 2.71 |  | 1886 |  | 1886 |  | Image |  | Peak List |  |
|  | | | | | | | | | | | | | | | | | | | | | | | | | | | | | | | | |
|  | High |  | LGGNVVVSLEGKPL |  | CID |  |  |  |  |  | 3.00 |  | 1.00 |  | 1 |  | SEQUEST (4) |  | 2 |  | 691.40851 |  | 3.41 |  | 1903 |  | 1903 |  | Image |  | Peak List |  |
|  | | | | | | | | | | | | | | | | | | | | | | | | | | | | | | | | |
|  | **Q9Y2Q3 - Glutathione S-transferase kappa 1 OS=Homo sapiens GN=GSTK1 PE=1 SV=3 - [GSTK1\_HUMAN]** | | | | | | | | | | | | | | | | | | | | | | | | | | | | | | |  |
|  | | | | | | | | | | | | | | | | | | | | | | | | | | | | | | | | |
|  | High |  | YGAFGLPITVAHVDGQTHMLFGSDR |  | CID |  |  |  | 27.68 |  |  |  | 1.00 |  | 1 |  | Mascot (2) |  | 3 |  | 897.11646 |  | 5.79 |  | 2406 |  | 2406 |  | Image |  | Peak List |  |
|  | | | | | | | | | | | | | | | | | | | | | | | | | | | | | | | | |
|  | High |  | YGAFGLPITVAHVDGQTHMLFGSDR |  | CID |  |  |  |  |  | 2.80 |  | 1.00 |  | 1 |  | SEQUEST (4) |  | 3 |  | 897.11646 |  | 5.79 |  | 2406 |  | 2406 |  | Image |  | Peak List |  |
|  | | | | | | | | | | | | | | | | | | | | | | | | | | | | | | | | |
|  | **Q9Y2S2 - Lambda-crystallin homolog OS=Homo sapiens GN=CRYL1 PE=1 SV=3 - [CRYL1\_HUMAN]** | | | | | | | | | | | | | | | | | | | | | | | | | | | | | | |  |
|  | | | | | | | | | | | | | | | | | | | | | | | | | | | | | | | | |
|  | High |  | SWAMLFASGGFQVK |  | CID |  |  |  | 53.69 |  |  |  | 1.00 |  | 1 |  | Mascot (2) |  | 2 |  | 764.88922 |  | 6.14 |  | 2914 |  | 2914 |  | Image |  | Peak List |  |
|  | | | | | | | | | | | | | | | | | | | | | | | | | | | | | | | | |
|  | High |  | SWAMLFASGGFQVK |  | CID |  |  |  |  |  | 4.40 |  | 1.00 |  | 1 |  | SEQUEST (4) |  | 2 |  | 764.88922 |  | 6.14 |  | 2914 |  | 2914 |  | Image |  | Peak List |  |
|  | | | | | | | | | | | | | | | | | | | | | | | | | | | | | | | | |
|  | **Q9Y490 - Talin-1 OS=Homo sapiens GN=TLN1 PE=1 SV=3 - [TLN1\_HUMAN]** | | | | | | | | | | | | | | | | | | | | | | | | | | | | | | |  |
|  | | | | | | | | | | | | | | | | | | | | | | | | | | | | | | | | |
|  | High |  | AAAFEEQENETVVVK |  | CID |  |  |  | 31.99 |  |  |  | 1.00 |  | 1 |  | Mascot (2) |  | 2 |  | 832.41766 |  | 6.38 |  | 1110 |  | 1110 |  | Image |  | Peak List |  |
|  | | | | | | | | | | | | | | | | | | | | | | | | | | | | | | | | |
|  | High |  | AAAFEEQENETVVVK |  | CID |  |  |  |  |  | 2.53 |  | 1.00 |  | 1 |  | SEQUEST (4) |  | 2 |  | 832.41766 |  | 6.38 |  | 1110 |  | 1110 |  | Image |  | Peak List |  |
|  | | | | | | | | | | | | | | | | | | | | | | | | | | | | | | | | |
|  | **A2RUS2-2 - Isoform 2 of DENN domain-containing protein 3 OS=Homo sapiens GN=DENND3 - [DEND3\_HUMAN]** | | | | | | | | | | | | | | | | | | | | | | | | | | | | | | |  |
|  | | | | | | | | | | | | | | | | | | | | | | | | | | | | | | | | |
|  | High |  | EADGLVLINIDHGSITYSK |  | CID |  |  |  |  |  | 1.29 |  | 1.00 |  | 1 |  | SEQUEST (4) |  | 3 |  | 682.35638 |  | -0.22 |  | 4864 |  | 4864 |  | Image |  | Peak List |  |
|  | | | | | | | | | | | | | | | | | | | | | | | | | | | | | | | | |
|  | **A2VEC9 - SCO-spondin OS=Homo sapiens GN=SSPO PE=2 SV=1 - [SSPO\_HUMAN]** | | | | | | | | | | | | | | | | | | | | | | | | | | | | | | |  |
|  | | | | | | | | | | | | | | | | | | | | | | | | | | | | | | | | |
|  | High |  | SCSSLAPGDTTCPGPHSQTR |  | CID |  |  |  |  |  | 1.78 |  | 1.00 |  | 1 |  | SEQUEST (4) |  | 2 |  | 1001.44269 |  | 1.36 |  | 1811 |  | 1811 |  | Image |  | Peak List |  |
|  | | | | | | | | | | | | | | | | | | | | | | | | | | | | | | | | |
|  | **A6NJH9 - Eukaryotic translation initiation factor 1A, Y-chromosomal OS=Homo sapiens GN=EIF1AY PE=4 SV=1 - [A6NJH9\_HUMAN]** | | | | | | | | | | | | | | | | | | | | | | | | | | | | | | |  |
|  | | | | | | | | | | | | | | | | | | | | | | | | | | | | | | | | |
|  | High |  | ELVFKEDGQEYAQVIK |  | CID |  |  |  |  |  | 1.76 |  | 1.00 |  | 1 |  | SEQUEST (4) |  | 3 |  | 632.66541 |  | 3.51 |  | 1548 |  | 1548 |  | Image |  | Peak List |  |
|  | | | | | | | | | | | | | | | | | | | | | | | | | | | | | | | | |
|  | **A8MT82 - Putative centaurin-gamma-like family member 11P OS=Homo sapiens GN=CTGLF11P PE=5 SV=2 - [CTLFB\_HUMAN]** | | | | | | | | | | | | | | | | | | | | | | | | | | | | | | |  |
|  | | | | | | | | | | | | | | | | | | | | | | | | | | | | | | | | |
|  | High |  | VmSSIGNDLANSIWEGSSQGQTKPSVK |  | CID |  | M2(Oxidation) |  |  |  | 2.00 |  | 1.00 |  | 1 |  | SEQUEST (4) |  | 4 |  | 709.85638 |  | 8.96 |  | 5936 |  | 5936 |  | Image |  | Peak List |  |
|  | | | | | | | | | | | | | | | | | | | | | | | | | | | | | | | | |
|  | **A8MU58 - Aminoacyl tRNA synthase complex-interacting multifunctional protein 2 OS=Homo sapiens GN=AIMP2 PE=4 SV=2 - [A8MU58\_HUMAN]** | | | | | | | | | | | | | | | | | | | | | | | | | | | | | | |  |
|  | | | | | | | | | | | | | | | | | | | | | | | | | | | | | | | | |
|  | High |  | HNAVNATLIDSWVDIAIFQLK |  | CID |  |  |  |  |  | 2.40 |  | 1.00 |  | 1 |  | SEQUEST (4) |  | 3 |  | 790.10083 |  | 9.24 |  | 3705 |  | 3705 |  | Image |  | Peak List |  |
|  | | | | | | | | | | | | | | | | | | | | | | | | | | | | | | | | |
|  | **B0QYA8 - Eukaryotic translation initiation factor 3 subunit D (Fragment) OS=Homo sapiens GN=EIF3D PE=4 SV=1 - [B0QYA8\_HUMAN]** | | | | | | | | | | | | | | | | | | | | | | | | | | | | | | |  |
|  | | | | | | | | | | | | | | | | | | | | | | | | | | | | | | | | |
|  | High |  | YSSQFGGGSQYAYFHEEDESSFQLVDTAR |  | CID |  |  |  |  |  | 2.21 |  | 1.00 |  | 1 |  | SEQUEST (4) |  | 3 |  | 1102.48230 |  | 0.86 |  | 2099 |  | 2099 |  | Image |  | Peak List |  |
|  | | | | | | | | | | | | | | | | | | | | | | | | | | | | | | | | |
|  | **B4DDQ6 - Protein phosphatase 2A activator, regulatory subunit 4 OS=Homo sapiens GN=PPP2R4 PE=2 SV=1 - [B4DDQ6\_HUMAN]** | | | | | | | | | | | | | | | | | | | | | | | | | | | | | | |  |
|  | | | | | | | | | | | | | | | | | | | | | | | | | | | | | | | | |
|  | High |  | CGTWGSCFLLPHCFALNLRPAPLTLGASESPIWGMGSQR |  | CID |  |  |  |  |  | 2.20 |  | 1.00 |  | 1 |  | SEQUEST (4) |  | 4 |  | 1044.25854 |  | -3.85 |  | 3809 |  | 3809 |  | Image |  | Peak List |  |
|  | | | | | | | | | | | | | | | | | | | | | | | | | | | | | | | | |
|  | **B4DEV8 - Proteasome subunit alpha type OS=Homo sapiens GN=PSMA1 PE=2 SV=1 - [B4DEV8\_HUMAN]** | | | | | | | | | | | | | | | | | | | | | | | | | | | | | | |  |
|  | | | | | | | | | | | | | | | | | | | | | | | | | | | | | | | | |
|  | High |  | ILHVDNHIGISIAGLTADAR |  | CID |  |  |  |  |  | 3.19 |  | 1.00 |  | 1 |  | SEQUEST (4) |  | 3 |  | 696.05359 |  | 2.76 |  | 1890 |  | 1890 |  | Image |  | Peak List |  |
|  | | | | | | | | | | | | | | | | | | | | | | | | | | | | | | | | |
|  | **B5MD47 - Septin-2 (Fragment) OS=Homo sapiens GN=SEPT2 PE=4 SV=1 - [B5MD47\_HUMAN]** | | | | | | | | | | | | | | | | | | | | | | | | | | | | | | |  |
|  | | | | | | | | | | | | | | | | | | | | | | | | | | | | | | | | |
|  | High |  | TVQIEASTVEIEER |  | CID |  |  |  |  |  | 1.81 |  | 1.00 |  | 1 |  | SEQUEST (4) |  | 2 |  | 802.41748 |  | 6.39 |  | 1376 |  | 1376 |  | Image |  | Peak List |  |
|  | | | | | | | | | | | | | | | | | | | | | | | | | | | | | | | | |
|  | **B7Z6A8 - Abhydrolase domain-containing protein 10, mitochondrial OS=Homo sapiens GN=ABHD10 PE=2 SV=1 - [B7Z6A8\_HUMAN]** | | | | | | | | | | | | | | | | | | | | | | | | | | | | | | |  |
|  | | | | | | | | | | | | | | | | | | | | | | | | | | | | | | | | |
|  | High |  | VVALIGVATAADTLVTK |  | CID |  |  |  |  |  | 2.43 |  | 1.00 |  | 1 |  | SEQUEST (4) |  | 3 |  | 548.00012 |  | 4.37 |  | 2704 |  | 2704 |  | Image |  | Peak List |  |
|  | | | | | | | | | | | | | | | | | | | | | | | | | | | | | | | | |
|  | **C9J9K3 - 40S ribosomal protein SA (Fragment) OS=Homo sapiens GN=RPSA PE=3 SV=1 - [C9J9K3\_HUMAN]** | | | | | | | | | | | | | | | | | | | | | | | | | | | | | | |  |
|  | | | | | | | | | | | | | | | | | | | | | | | | | | | | | | | | |
|  | High |  | FLAAGTHLGGTNLDFQMEQYIYK |  | CID |  |  |  |  |  | 3.13 |  | 1.00 |  | 1 |  | SEQUEST (4) |  | 3 |  | 873.10114 |  | 5.05 |  | 2395 |  | 2395 |  | Image |  | Peak List |  |
|  | | | | | | | | | | | | | | | | | | | | | | | | | | | | | | | | |
|  | **C9JLH5 - Elongator complex protein 6 homolog (Fragment) OS=Homo sapiens GN=C3orf75 PE=4 SV=1 - [C9JLH5\_HUMAN]** | | | | | | | | | | | | | | | | | | | | | | | | | | | | | | |  |
|  | | | | | | | | | | | | | | | | | | | | | | | | | | | | | | | | |
|  | High |  | EPHPLQFLREANAGNLKPLFEFVREALKPVDSGEAR |  | CID |  |  |  |  |  | 2.54 |  | 1.00 |  | 1 |  | SEQUEST (4) |  | 3 |  | 1359.06738 |  | 8.88 |  | 4254 |  | 4254 |  | Image |  | Peak List |  |
|  | | | | | | | | | | | | | | | | | | | | | | | | | | | | | | | | |
|  | **D3DQ64 - Adenylate kinase isoenzyme 4, mitochondrial OS=Homo sapiens GN=AK4 PE=3 SV=1 - [D3DQ64\_HUMAN]** | | | | | | | | | | | | | | | | | | | | | | | | | | | | | | |  |
|  | | | | | | | | | | | | | | | | | | | | | | | | | | | | | | | | |
|  | High |  | TLGQAEALDKICEVDLVISLNIPFETLK |  | CID |  |  |  |  |  | 2.25 |  | 1.00 |  | 1 |  | SEQUEST (4) |  | 3 |  | 1024.89404 |  | 4.46 |  | 4064 |  | 4064 |  | Image |  | Peak List |  |
|  | | | | | | | | | | | | | | | | | | | | | | | | | | | | | | | | |
|  | **D6R9R1 - Methylcrotonoyl-CoA carboxylase beta chain, mitochondrial OS=Homo sapiens GN=MCCC2 PE=4 SV=2 - [D6R9R1\_HUMAN]** | | | | | | | | | | | | | | | | | | | | | | | | | | | | | | |  |
|  | | | | | | | | | | | | | | | | | | | | | | | | | | | | | | | | |
|  | High |  | VSGVECMIIANDATVK |  | CID |  |  |  |  |  | 3.35 |  | 1.00 |  | 1 |  | SEQUEST (4) |  | 2 |  | 825.41846 |  | 3.54 |  | 1994 |  | 1994 |  | Image |  | Peak List |  |
|  | | | | | | | | | | | | | | | | | | | | | | | | | | | | | | | | |
|  | **D6RD47 - 40S ribosomal protein S23 OS=Homo sapiens GN=RPS23 PE=3 SV=1 - [D6RD47\_HUMAN]** | | | | | | | | | | | | | | | | | | | | | | | | | | | | | | |  |
|  | | | | | | | | | | | | | | | | | | | | | | | | | | | | | | | | |
|  | High |  | ITAFVPNDGCLNFIEENDEVLVAGFGR |  | CID |  |  |  |  |  | 2.38 |  | 1.00 |  | 1 |  | SEQUEST (4) |  | 3 |  | 980.48151 |  | 1.85 |  | 3913 |  | 3913 |  | Image |  | Peak List |  |
|  | | | | | | | | | | | | | | | | | | | | | | | | | | | | | | | | |
|  | **E9PBI1 - Alcohol dehydrogenase 6 OS=Homo sapiens GN=ADH6 PE=3 SV=1 - [E9PBI1\_HUMAN]** | | | | | | | | | | | | | | | | | | | | | | | | | | | | | | |  |
|  | | | | | | | | | | | | | | | | | | | | | | | | | | | | | | | | |
|  | High |  | SIYHFGNTSTFCEYTVIK |  | CID |  |  |  |  |  | 2.05 |  | 1.00 |  | 1 |  | SEQUEST (4) |  | 3 |  | 704.00519 |  | 2.82 |  | 1910 |  | 1910 |  | Image |  | Peak List |  |
|  | | | | | | | | | | | | | | | | | | | | | | | | | | | | | | | | |
|  | **E9PBV1 - NACHT and WD repeat domain-containing protein 1 OS=Homo sapiens GN=NWD1 PE=4 SV=1 - [E9PBV1\_HUMAN]** | | | | | | | | | | | | | | | | | | | | | | | | | | | | | | |  |
|  | | | | | | | | | | | | | | | | | | | | | | | | | | | | | | | | |
|  | High |  | LEQTHGQLLVAHVLGYIVSSR |  | CID |  |  |  |  |  | 1.70 |  | 1.00 |  | 1 |  | SEQUEST (4) |  | 3 |  | 774.09558 |  | -2.19 |  | 5730 |  | 5730 |  | Image |  | Peak List |  |
|  | | | | | | | | | | | | | | | | | | | | | | | | | | | | | | | | |
|  | **F6SBX2 - Isoleucine--tRNA ligase, mitochondrial OS=Homo sapiens GN=IARS2 PE=3 SV=1 - [F6SBX2\_HUMAN]** | | | | | | | | | | | | | | | | | | | | | | | | | | | | | | |  |
|  | | | | | | | | | | | | | | | | | | | | | | | | | | | | | | | | |
|  | High |  | AVLEEGTDVVIK |  | CID |  |  |  |  |  | 2.49 |  | 1.00 |  | 1 |  | SEQUEST (4) |  | 2 |  | 636.85901 |  | 4.80 |  | 1342 |  | 1342 |  | Image |  | Peak List |  |
|  | | | | | | | | | | | | | | | | | | | | | | | | | | | | | | | | |
|  | **F8VYZ3 - Serine dehydratase-like (Fragment) OS=Homo sapiens GN=SDSL PE=4 SV=1 - [F8VYZ3\_HUMAN]** | | | | | | | | | | | | | | | | | | | | | | | | | | | | | | |  |
|  | | | | | | | | | | | | | | | | | | | | | | | | | | | | | | | | |
|  | High |  | HLVCSSGGNAGIAAAYAAR |  | CID |  |  |  |  |  | 2.10 |  | 1.00 |  | 1 |  | SEQUEST (4) |  | 3 |  | 596.96704 |  | 3.08 |  | 1019 |  | 1019 |  | Image |  | Peak List |  |
|  | | | | | | | | | | | | | | | | | | | | | | | | | | | | | | | | |
|  | **F8W1D4 - Phenylalanine-4-hydroxylase (Fragment) OS=Homo sapiens GN=PAH PE=4 SV=1 - [F8W1D4\_HUMAN]** | | | | | | | | | | | | | | | | | | | | | | | | | | | | | | |  |
|  | | | | | | | | | | | | | | | | | | | | | | | | | | | | | | | | |
|  | High |  | LSDFGQETSYIEDNCNQNGAISLIFSLK |  | CID |  |  |  |  |  | 2.21 |  | 1.00 |  | 1 |  | SEQUEST (4) |  | 3 |  | 1036.17065 |  | 9.63 |  | 3641 |  | 3641 |  | Image |  | Peak List |  |
|  | | | | | | | | | | | | | | | | | | | | | | | | | | | | | | | | |
|  | **F8W7D6 - Molybdopterin adenylyltransferase OS=Homo sapiens GN=GPHN PE=4 SV=1 - [F8W7D6\_HUMAN]** | | | | | | | | | | | | | | | | | | | | | | | | | | | | | | |  |
|  | | | | | | | | | | | | | | | | | | | | | | | | | | | | | | | | |
|  | High |  | CILTWHHQEPLPWAQSTGNQMSSR |  | CID |  |  |  |  |  | 2.02 |  | 1.00 |  | 1 |  | SEQUEST (4) |  | 5 |  | 562.27142 |  | 4.93 |  | 1383 |  | 1383 |  | Image |  | Peak List |  |
|  | | | | | | | | | | | | | | | | | | | | | | | | | | | | | | | | |
|  | **H0Y4W2 - Transformation/transcription domain-associated protein (Fragment) OS=Homo sapiens GN=TRRAP PE=4 SV=1 - [H0Y4W2\_HUMAN]** | | | | | | | | | | | | | | | | | | | | | | | | | | | | | | |  |
|  | | | | | | | | | | | | | | | | | | | | | | | | | | | | | | | | |
|  | High |  | YSQQmVKGMLQLLSNCPAETAHLRK |  | CID |  | M5(Oxidation) |  |  |  | 2.22 |  | 0.05 |  | 1 |  | SEQUEST (4) |  | 3 |  | 954.82684 |  | 8.23 |  | 6328 |  | 6328 |  | Image |  | Peak List |  |
|  | | | | | | | | | | | | | | | | | | | | | | | | | | | | | | | | |
|  | **H0YF40 - Aldehyde dehydrogenase family 8 member A1 (Fragment) OS=Homo sapiens GN=ALDH8A1 PE=4 SV=1 - [H0YF40\_HUMAN]** | | | | | | | | | | | | | | | | | | | | | | | | | | | | | | |  |
|  | | | | | | | | | | | | | | | | | | | | | | | | | | | | | | | | |
|  | High |  | YGLAATVWSSNVGR |  | CID |  |  |  |  |  | 2.33 |  | 1.00 |  | 1 |  | SEQUEST (4) |  | 2 |  | 740.88464 |  | 5.17 |  | 1819 |  | 1819 |  | Image |  | Peak List |  |
|  | | | | | | | | | | | | | | | | | | | | | | | | | | | | | | | | |
|  | **H0YLS6 - Proteasome subunit alpha type OS=Homo sapiens GN=PSMA4 PE=3 SV=1 - [H0YLS6\_HUMAN]** | | | | | | | | | | | | | | | | | | | | | | | | | | | | | | |  |
|  | | | | | | | | | | | | | | | | | | | | | | | | | | | | | | | | |
|  | High |  | LYQVEYAMEAIGHAGTCLGILANDGVLLAAER |  | CID |  |  |  |  |  | 2.86 |  | 1.00 |  | 1 |  | SEQUEST (4) |  | 3 |  | 1121.24438 |  | 7.89 |  | 4185 |  | 4185 |  | Image |  | Peak List |  |
|  | | | | | | | | | | | | | | | | | | | | | | | | | | | | | | | | |
|  | **H3BP04 - Cytochrome b-c1 complex subunit 2, mitochondrial (Fragment) OS=Homo sapiens GN=UQCRC2 PE=4 SV=1 - [H3BP04\_HUMAN]** | | | | | | | | | | | | | | | | | | | | | | | | | | | | | | |  |
|  | | | | | | | | | | | | | | | | | | | | | | | | | | | | | | | | |
|  | High |  | GDVDILMEFLLNVTTAPEFR |  | CID |  |  |  |  |  | 1.98 |  | 1.00 |  | 1 |  | SEQUEST (4) |  | 3 |  | 760.73035 |  | 8.03 |  | 5214 |  | 5214 |  | Image |  | Peak List |  |
|  | | | | | | | | | | | | | | | | | | | | | | | | | | | | | | | | |
|  | **H7C0A4 - Vigilin (Fragment) OS=Homo sapiens GN=HDLBP PE=4 SV=1 - [H7C0A4\_HUMAN]** | | | | | | | | | | | | | | | | | | | | | | | | | | | | | | |  |
|  | | | | | | | | | | | | | | | | | | | | | | | | | | | | | | | | |
|  | High |  | VHIEFTEGEDKITLEGPTEDVNVAQEQIEGMVK |  | CID |  |  |  |  |  | 2.19 |  | 1.00 |  | 1 |  | SEQUEST (4) |  | 4 |  | 921.95898 |  | 5.16 |  | 2507 |  | 2507 |  | Image |  | Peak List |  |
|  | | | | | | | | | | | | | | | | | | | | | | | | | | | | | | | | |
|  | **H7C402 - Proteasome subunit alpha type-2 (Fragment) OS=Homo sapiens GN=PSMA2 PE=4 SV=1 - [H7C402\_HUMAN]** | | | | | | | | | | | | | | | | | | | | | | | | | | | | | | |  |
|  | | | | | | | | | | | | | | | | | | | | | | | | | | | | | | | | |
|  | High |  | LVQIEYALAAVAGGAPSVGIK |  | CID |  |  |  |  |  | 2.18 |  | 1.00 |  | 1 |  | SEQUEST (4) |  | 3 |  | 676.39233 |  | 4.33 |  | 3115 |  | 3115 |  | Image |  | Peak List |  |
|  | | | | | | | | | | | | | | | | | | | | | | | | | | | | | | | | |
|  | **H7C4C8 - T-complex protein 1 subunit theta (Fragment) OS=Homo sapiens GN=CCT8 PE=3 SV=1 - [H7C4C8\_HUMAN]** | | | | | | | | | | | | | | | | | | | | | | | | | | | | | | |  |
|  | | | | | | | | | | | | | | | | | | | | | | | | | | | | | | | | |
|  | High |  | LTPPVLEEMGHCDSVYLSEVGDTQVVVFK |  | CID |  |  |  |  |  | 3.13 |  | 1.00 |  | 1 |  | SEQUEST (4) |  | 3 |  | 1064.53198 |  | 5.43 |  | 3039 |  | 3039 |  | Image |  | Peak List |  |
|  | | | | | | | | | | | | | | | | | | | | | | | | | | | | | | | | |
|  | **I3L0K7 - Heat shock protein 75 kDa, mitochondrial OS=Homo sapiens GN=TRAP1 PE=4 SV=1 - [I3L0K7\_HUMAN]** | | | | | | | | | | | | | | | | | | | | | | | | | | | | | | |  |
|  | | | | | | | | | | | | | | | | | | | | | | | | | | | | | | | | |
|  | High |  | DTEVLFCFEQFDELTLLHLR |  | CID |  |  |  |  |  | 2.17 |  | 1.00 |  | 1 |  | SEQUEST (4) |  | 3 |  | 823.41541 |  | 6.05 |  | 4112 |  | 4112 |  | Image |  | Peak List |  |
|  | | | | | | | | | | | | | | | | | | | | | | | | | | | | | | | | |
|  | **O75356 - Ectonucleoside triphosphate diphosphohydrolase 5 OS=Homo sapiens GN=ENTPD5 PE=1 SV=1 - [ENTP5\_HUMAN]** | | | | | | | | | | | | | | | | | | | | | | | | | | | | | | |  |
|  | | | | | | | | | | | | | | | | | | | | | | | | | | | | | | | | |
|  | High |  | VNNIETGWALGATFHLLQSLGISH |  | CID |  |  |  |  |  | 1.94 |  | 1.00 |  | 1 |  | SEQUEST (4) |  | 3 |  | 860.12415 |  | 6.41 |  | 3923 |  | 3923 |  | Image |  | Peak List |  |
|  | | | | | | | | | | | | | | | | | | | | | | | | | | | | | | | | |
|  | **P07108 - Acyl-CoA-binding protein OS=Homo sapiens GN=DBI PE=1 SV=2 - [ACBP\_HUMAN]** | | | | | | | | | | | | | | | | | | | | | | | | | | | | | | |  |
|  | | | | | | | | | | | | | | | | | | | | | | | | | | | | | | | | |
|  | High |  | TKPSDEEMLFIYGHYK |  | CID |  |  |  |  |  | 2.15 |  | 1.00 |  | 1 |  | SEQUEST (4) |  | 3 |  | 653.32086 |  | 5.89 |  | 1474 |  | 1474 |  | Image |  | Peak List |  |
|  | | | | | | | | | | | | | | | | | | | | | | | | | | | | | | | | |
|  | **P0C876 - Uncharacterized protein FLJ90687 OS=Homo sapiens PE=1 SV=1 - [YB053\_HUMAN]** | | | | | | | | | | | | | | | | | | | | | | | | | | | | | | |  |
|  | | | | | | | | | | | | | | | | | | | | | | | | | | | | | | | | |
|  | High |  | EGGGPDQPAGSPAPLRPPLPR |  | CID |  |  |  |  |  | 1.52 |  | 1.00 |  | 1 |  | SEQUEST (4) |  | 3 |  | 689.37054 |  | 9.29 |  | 7079 |  | 7079 |  | Image |  | Peak List |  |
|  | | | | | | | | | | | | | | | | | | | | | | | | | | | | | | | | |
|  | **P11509 - Cytochrome P450 2A6 OS=Homo sapiens GN=CYP2A6 PE=1 SV=3 - [CP2A6\_HUMAN]** | | | | | | | | | | | | | | | | | | | | | | | | | | | | | | |  |
|  | | | | | | | | | | | | | | | | | | | | | | | | | | | | | | | | |
|  | High |  | NLVMTTLNLFIGGTETVSTTLR |  | CID |  |  |  |  |  | 3.97 |  | 1.00 |  | 1 |  | SEQUEST (4) |  | 3 |  | 794.43616 |  | 8.12 |  | 4232 |  | 4232 |  | Image |  | Peak List |  |
|  | | | | | | | | | | | | | | | | | | | | | | | | | | | | | | | | |
|  | **P13798 - Acylamino-acid-releasing enzyme OS=Homo sapiens GN=APEH PE=1 SV=4 - [ACPH\_HUMAN]** | | | | | | | | | | | | | | | | | | | | | | | | | | | | | | |  |
|  | | | | | | | | | | | | | | | | | | | | | | | | | | | | | | | | |
|  | High |  | STHALSEVEVESDSFMNAVLWLR |  | CID |  |  |  |  |  | 2.72 |  | 1.00 |  | 1 |  | SEQUEST (4) |  | 3 |  | 874.10065 |  | 6.14 |  | 3325 |  | 3325 |  | Image |  | Peak List |  |
|  | | | | | | | | | | | | | | | | | | | | | | | | | | | | | | | | |
|  | **B4E3C2 - 60S ribosomal protein L17 OS=Homo sapiens GN=RPL17 PE=2 SV=1 - [B4E3C2\_HUMAN]** | | | | | | | | | | | | | | | | | | | | | | | | | | | | | | |  |
|  | | | | | | | | | | | | | | | | | | | | | | | | | | | | | | | | |
|  | High |  | GLDVDSLVIEHIQVNK |  | CID |  |  |  |  |  | 2.37 |  | 1.00 |  | 1 |  | SEQUEST (4) |  | 3 |  | 593.66217 |  | 4.04 |  | 2197 |  | 2197 |  | Image |  | Peak List |  |
|  | | | | | | | | | | | | | | | | | | | | | | | | | | | | | | | | |
|  | **A6NKK6 - UDP-glucuronosyltransferase 1-6 OS=Homo sapiens GN=UGT1A6 PE=3 SV=1 - [A6NKK6\_HUMAN]** | | | | | | | | | | | | | | | | | | | | | | | | | | | | | | |  |
|  | | | | | | | | | | | | | | | | | | | | | | | | | | | | | | | | |
|  | High |  | DRPVEPLDLAVFWVEFVMR |  | CID |  |  |  |  |  | 2.27 |  | 1.00 |  | 1 |  | SEQUEST (4) |  | 3 |  | 773.40906 |  | 5.31 |  | 5125 |  | 5125 |  | Image |  | Peak List |  |
|  | | | | | | | | | | | | | | | | | | | | | | | | | | | | | | | | |
|  | **P20749 - B-cell lymphoma 3 protein OS=Homo sapiens GN=BCL3 PE=1 SV=2 - [BCL3\_HUMAN]** | | | | | | | | | | | | | | | | | | | | | | | | | | | | | | |  |
|  | | | | | | | | | | | | | | | | | | | | | | | | | | | | | | | | |
|  | High |  | DPPGFPMAPPNFFLPSPSPPAFLPFAGVLR |  | CID |  |  |  |  |  | 1.90 |  | 1.00 |  | 1 |  | SEQUEST (4) |  | 3 |  | 1060.88232 |  | -1.80 |  | 3475 |  | 3475 |  | Image |  | Peak List |  |
|  | | | | | | | | | | | | | | | | | | | | | | | | | | | | | | | | |
|  | **P24298 - Alanine aminotransferase 1 OS=Homo sapiens GN=GPT PE=1 SV=3 - [ALAT1\_HUMAN]** | | | | | | | | | | | | | | | | | | | | | | | | | | | | | | |  |
|  | | | | | | | | | | | | | | | | | | | | | | | | | | | | | | | | |
|  | High |  | TGVLIPIPQYPLYSATLAELGAVQVDYYLDEER |  | CID |  |  |  |  |  | 2.02 |  | 1.00 |  | 1 |  | SEQUEST (4) |  | 3 |  | 1232.98499 |  | 9.62 |  | 4467 |  | 4467 |  | Image |  | Peak List |  |
|  | | | | | | | | | | | | | | | | | | | | | | | | | | | | | | | | |
|  | **P26641 - Elongation factor 1-gamma OS=Homo sapiens GN=EEF1G PE=1 SV=3 - [EF1G\_HUMAN]** | | | | | | | | | | | | | | | | | | | | | | | | | | | | | | |  |
|  | | | | | | | | | | | | | | | | | | | | | | | | | | | | | | | | |
|  | High |  | VPAFEGDDGFCVFESNAIAYYVSNEELR |  | CID |  |  |  |  |  | 3.22 |  | 1.00 |  | 1 |  | SEQUEST (4) |  | 3 |  | 1047.81934 |  | 9.14 |  | 3790 |  | 3790 |  | Image |  | Peak List |  |
|  | | | | | | | | | | | | | | | | | | | | | | | | | | | | | | | | |
|  | **P49721 - Proteasome subunit beta type-2 OS=Homo sapiens GN=PSMB2 PE=1 SV=1 - [PSB2\_HUMAN]** | | | | | | | | | | | | | | | | | | | | | | | | | | | | | | |  |
|  | | | | | | | | | | | | | | | | | | | | | | | | | | | | | | | | |
|  | High |  | ILLLCVGEAGDTVQFAEYIQK |  | CID |  |  |  |  |  | 4.00 |  | 1.00 |  | 1 |  | SEQUEST (4) |  | 3 |  | 770.74451 |  | 6.00 |  | 3296 |  | 3296 |  | Image |  | Peak List |  |
|  | | | | | | | | | | | | | | | | | | | | | | | | | | | | | | | | |
|  | **P51580 - Thiopurine S-methyltransferase OS=Homo sapiens GN=TPMT PE=1 SV=1 - [TPMT\_HUMAN]** | | | | | | | | | | | | | | | | | | | | | | | | | | | | | | |  |
|  | | | | | | | | | | | | | | | | | | | | | | | | | | | | | | | | |
|  | High |  | GHSVVGVEISELGIQEFFTEQNLSYSEEPITEIPGTK |  | CID |  |  |  |  |  | 3.39 |  | 1.00 |  | 1 |  | SEQUEST (4) |  | 3 |  | 1355.35059 |  | 8.56 |  | 3330 |  | 3330 |  | Image |  | Peak List |  |
|  | | | | | | | | | | | | | | | | | | | | | | | | | | | | | | | | |
|  | **P62906 - 60S ribosomal protein L10a OS=Homo sapiens GN=RPL10A PE=1 SV=2 - [RL10A\_HUMAN]** | | | | | | | | | | | | | | | | | | | | | | | | | | | | | | |  |
|  | | | | | | | | | | | | | | | | | | | | | | | | | | | | | | | | |
|  | High |  | MTDDELVYNIHLAVNFLVSLLK |  | CID |  |  |  |  |  | 2.15 |  | 1.00 |  | 1 |  | SEQUEST (4) |  | 3 |  | 849.79724 |  | 9.55 |  | 5470 |  | 5470 |  | Image |  | Peak List |  |
|  | | | | | | | | | | | | | | | | | | | | | | | | | | | | | | | | |
|  | **P78371-2 - Isoform 2 of T-complex protein 1 subunit beta OS=Homo sapiens GN=CCT2 - [TCPB\_HUMAN]** | | | | | | | | | | | | | | | | | | | | | | | | | | | | | | |  |
|  | | | | | | | | | | | | | | | | | | | | | | | | | | | | | | | | |
|  | High |  | EGTIGDMAILGITESFQVK |  | CID |  |  |  |  |  | 3.12 |  | 1.00 |  | 1 |  | SEQUEST (4) |  | 2 |  | 1005.02484 |  | 8.13 |  | 3449 |  | 3449 |  | Image |  | Peak List |  |
|  | | | | | | | | | | | | | | | | | | | | | | | | | | | | | | | | |
|  | **Q00610-2 - Isoform 2 of Clathrin heavy chain 1 OS=Homo sapiens GN=CLTC - [CLH1\_HUMAN]** | | | | | | | | | | | | | | | | | | | | | | | | | | | | | | |  |
|  | | | | | | | | | | | | | | | | | | | | | | | | | | | | | | | | |
|  | High |  | LDNYDAPDIANIAISNELFEEAFAIFR |  | CID |  |  |  |  |  | 3.49 |  | 1.00 |  | 1 |  | SEQUEST (4) |  | 3 |  | 1024.51477 |  | 9.75 |  | 4930 |  | 4930 |  | Image |  | Peak List |  |
|  | | | | | | | | | | | | | | | | | | | | | | | | | | | | | | | | |
|  | **Q05707-3 - Isoform 3 of Collagen alpha-1(XIV) chain OS=Homo sapiens GN=COL14A1 - [COEA1\_HUMAN]** | | | | | | | | | | | | | | | | | | | | | | | | | | | | | | |  |
|  | | | | | | | | | | | | | | | | | | | | | | | | | | | | | | | | |
|  | High |  | IVYKPVSVPGPTLETFVGADINTILITNLLSGmDYNVK |  | CID |  | M33(Oxidation) |  |  |  | 1.62 |  | 1.00 |  | 1 |  | SEQUEST (4) |  | 5 |  | 822.44336 |  | -1.25 |  | 3233 |  | 3233 |  | Image |  | Peak List |  |
|  | | | | | | | | | | | | | | | | | | | | | | | | | | | | | | | | |
|  | **Q49AI2 - BPHL protein OS=Homo sapiens GN=BPHL PE=2 SV=1 - [Q49AI2\_HUMAN]** | | | | | | | | | | | | | | | | | | | | | | | | | | | | | | |  |
|  | | | | | | | | | | | | | | | | | | | | | | | | | | | | | | | | |
|  | High |  | MVIWGANAYVTDEDSMIYEGIR |  | CID |  |  |  |  |  | 3.05 |  | 1.00 |  | 1 |  | SEQUEST (4) |  | 2 |  | 1267.10010 |  | 7.51 |  | 3088 |  | 3088 |  | Image |  | Peak List |  |
|  | | | | | | | | | | | | | | | | | | | | | | | | | | | | | | | | |
|  | **Q5VZC3 - Fructose-1,6-bisphosphatase 1 (Fragment) OS=Homo sapiens GN=FBP1 PE=2 SV=1 - [Q5VZC3\_HUMAN]** | | | | | | | | | | | | | | | | | | | | | | | | | | | | | | |  |
|  | | | | | | | | | | | | | | | | | | | | | | | | | | | | | | | | |
|  | High |  | SSFATCVLVSEEDKHAIIVEPEKR |  | CID |  |  |  |  |  | 3.66 |  | 1.00 |  | 1 |  | SEQUEST (4) |  | 3 |  | 896.46643 |  | 5.10 |  | 1458 |  | 1458 |  | Image |  | Peak List |  |
|  | | | | | | | | | | | | | | | | | | | | | | | | | | | | | | | | |
|  | **Q6IB77 - Glycine N-acyltransferase OS=Homo sapiens GN=GLYAT PE=1 SV=3 - [GLYAT\_HUMAN]** | | | | | | | | | | | | | | | | | | | | | | | | | | | | | | |  |
|  | | | | | | | | | | | | | | | | | | | | | | | | | | | | | | | | |
|  | High |  | LHGLVTYVIYSHAQK |  | CID |  |  |  |  |  | 2.30 |  | 1.00 |  | 1 |  | SEQUEST (4) |  | 3 |  | 576.98895 |  | 5.27 |  | 1165 |  | 1165 |  | Image |  | Peak List |  |
|  | | | | | | | | | | | | | | | | | | | | | | | | | | | | | | | | |
|  | **Q6NUN0 - Acyl-coenzyme A synthetase ACSM5, mitochondrial OS=Homo sapiens GN=ACSM5 PE=2 SV=2 - [ACSM5\_HUMAN]** | | | | | | | | | | | | | | | | | | | | | | | | | | | | | | |  |
|  | | | | | | | | | | | | | | | | | | | | | | | | | | | | | | | | |
|  | High |  | HQTGVELYEGYGQSETVVICANPK |  | CID |  |  |  |  |  | 3.27 |  | 1.00 |  | 1 |  | SEQUEST (4) |  | 3 |  | 874.76178 |  | 7.72 |  | 1652 |  | 1652 |  | Image |  | Peak List |  |
|  | | | | | | | | | | | | | | | | | | | | | | | | | | | | | | | | |
|  | **Q6ZTQ4-2 - Isoform 2 of Cadherin-related family member 3 OS=Homo sapiens GN=CDHR3 - [CDHR3\_HUMAN]** | | | | | | | | | | | | | | | | | | | | | | | | | | | | | | |  |
|  | | | | | | | | | | | | | | | | | | | | | | | | | | | | | | | | |
|  | High |  | IQITFIVEDVNDNPATCQK |  | CID |  |  |  |  |  | 1.79 |  | 1.00 |  | 1 |  | SEQUEST (4) |  | 3 |  | 716.69647 |  | 4.94 |  | 1558 |  | 1558 |  | Image |  | Peak List |  |
|  | | | | | | | | | | | | | | | | | | | | | | | | | | | | | | | | |
|  | **Q86WI1 - Fibrocystin-L OS=Homo sapiens GN=PKHD1L1 PE=2 SV=2 - [PKHL1\_HUMAN]** | | | | | | | | | | | | | | | | | | | | | | | | | | | | | | |  |
|  | | | | | | | | | | | | | | | | | | | | | | | | | | | | | | | | |
|  | High |  | IDGEPCPGQFNPVEK |  | CID |  |  |  |  |  | 1.04 |  | 1.00 |  | 1 |  | SEQUEST (4) |  | 3 |  | 543.92615 |  | 3.72 |  | 1142 |  | 1142 |  | Image |  | Peak List |  |
|  | | | | | | | | | | | | | | | | | | | | | | | | | | | | | | | | |
|  | **Q8NFA0 - Ubiquitin carboxyl-terminal hydrolase 32 OS=Homo sapiens GN=USP32 PE=1 SV=1 - [UBP32\_HUMAN]** | | | | | | | | | | | | | | | | | | | | | | | | | | | | | | |  |
|  | | | | | | | | | | | | | | | | | | | | | | | | | | | | | | | | |
|  | High |  | ELHPDEIDTDSAYILFYEQQGIDYAQFLPKTDGK |  | CID |  |  |  |  |  | 1.67 |  | 1.00 |  | 1 |  | SEQUEST (4) |  | 4 |  | 990.73285 |  | 5.87 |  | 1984 |  | 1984 |  | Image |  | Peak List |  |
|  | | | | | | | | | | | | | | | | | | | | | | | | | | | | | | | | |
|  | **Q8WXR4-6 - Isoform 6 of Myosin-IIIb OS=Homo sapiens GN=MYO3B - [MYO3B\_HUMAN]** | | | | | | | | | | | | | | | | | | | | | | | | | | | | | | |  |
|  | | | | | | | | | | | | | | | | | | | | | | | | | | | | | | | | |
|  | High |  | RNESAAHNQAGDTSNQSSGPHSPVAAGTR |  | CID |  |  |  |  |  | 1.92 |  | 1.00 |  | 1 |  | SEQUEST (4) |  | 3 |  | 968.79163 |  | 9.45 |  | 1778 |  | 1778 |  | Image |  | Peak List |  |
|  | | | | | | | | | | | | | | | | | | | | | | | | | | | | | | | | |
|  | **Q92786 - Prospero homeobox protein 1 OS=Homo sapiens GN=PROX1 PE=1 SV=2 - [PROX1\_HUMAN]** | | | | | | | | | | | | | | | | | | | | | | | | | | | | | | |  |
|  | | | | | | | | | | | | | | | | | | | | | | | | | | | | | | | | |
|  | High |  | HLAETLKQELNTAMSQVVDTVVK |  | CID |  |  |  |  |  | 2.18 |  | 1.00 |  | 1 |  | SEQUEST (4) |  | 3 |  | 852.11914 |  | -4.60 |  | 5773 |  | 5773 |  | Image |  | Peak List |  |
|  | | | | | | | | | | | | | | | | | | | | | | | | | | | | | | | | |
|  | **Q96AB3-3 - Isoform 3 of Isochorismatase domain-containing protein 2, mitochondrial OS=Homo sapiens GN=ISOC2 - [ISOC2\_HUMAN]** | | | | | | | | | | | | | | | | | | | | | | | | | | | | | | |  |
|  | | | | | | | | | | | | | | | | | | | | | | | | | | | | | | | | |
|  | High |  | GLQVHVVVDACSSR |  | CID |  |  |  |  |  | 2.73 |  | 1.00 |  | 1 |  | SEQUEST (4) |  | 3 |  | 490.59152 |  | 4.78 |  | 1210 |  | 1210 |  | Image |  | Peak List |  |
|  | | | | | | | | | | | | | | | | | | | | | | | | | | | | | | | | |
|  | **Q96CN7 - Isochorismatase domain-containing protein 1 OS=Homo sapiens GN=ISOC1 PE=1 SV=3 - [ISOC1\_HUMAN]** | | | | | | | | | | | | | | | | | | | | | | | | | | | | | | |  |
|  | | | | | | | | | | | | | | | | | | | | | | | | | | | | | | | | |
|  | High |  | TGIIVTTSEAVLLQLVADKDHPK |  | CID |  |  |  |  |  | 3.45 |  | 1.00 |  | 1 |  | SEQUEST (4) |  | 4 |  | 612.85260 |  | 7.20 |  | 2658 |  | 2658 |  | Image |  | Peak List |  |
|  | | | | | | | | | | | | | | | | | | | | | | | | | | | | | | | | |
|  | **Q96D53 - Uncharacterized aarF domain-containing protein kinase 4 OS=Homo sapiens GN=ADCK4 PE=1 SV=2 - [ADCK4\_HUMAN]** | | | | | | | | | | | | | | | | | | | | | | | | | | | | | | |  |
|  | | | | | | | | | | | | | | | | | | | | | | | | | | | | | | | | |
|  | High |  | VGQMLSIQDNSFISPQLQHIFER |  | CID |  |  |  |  |  | 2.24 |  | 1.00 |  | 1 |  | SEQUEST (4) |  | 5 |  | 538.28223 |  | 7.78 |  | 1480 |  | 1480 |  | Image |  | Peak List |  |
|  | | | | | | | | | | | | | | | | | | | | | | | | | | | | | | | | |
|  | **Q99460-2 - Isoform 2 of 26S proteasome non-ATPase regulatory subunit 1 OS=Homo sapiens GN=PSMD1 - [PSMD1\_HUMAN]** | | | | | | | | | | | | | | | | | | | | | | | | | | | | | | |  |
|  | | | | | | | | | | | | | | | | | | | | | | | | | | | | | | | | |
|  | High |  | HGGSLGLGLAAMGTAR |  | CID |  |  |  |  |  | 1.45 |  | 1.00 |  | 1 |  | SEQUEST (4) |  | 3 |  | 490.26312 |  | 3.96 |  | 1326 |  | 1326 |  | Image |  | Peak List |  |
|  | | | | | | | | | | | | | | | | | | | | | | | | | | | | | | | | |
|  | **Q99757 - Thioredoxin, mitochondrial OS=Homo sapiens GN=TXN2 PE=1 SV=2 - [THIOM\_HUMAN]** | | | | | | | | | | | | | | | | | | | | | | | | | | | | | | |  |
|  | | | | | | | | | | | | | | | | | | | | | | | | | | | | | | | | |
|  | High |  | VDIDDHTDLAIEYEVSAVPTVLAMK |  | CID |  |  |  |  |  | 1.91 |  | 1.00 |  | 1 |  | SEQUEST (4) |  | 3 |  | 915.46698 |  | 5.96 |  | 3102 |  | 3102 |  | Image |  | Peak List |  |
|  | | | | | | | | | | | | | | | | | | | | | | | | | | | | | | | | |
|  | **Q9H3U1-2 - Isoform 2 of Protein unc-45 homolog A OS=Homo sapiens GN=UNC45A - [UN45A\_HUMAN]** | | | | | | | | | | | | | | | | | | | | | | | | | | | | | | |  |
|  | | | | | | | | | | | | | | | | | | | | | | | | | | | | | | | | |
|  | High |  | AAAGGLAMLTSMRPTLCSR |  | CID |  |  |  |  |  | 1.11 |  | 1.00 |  | 1 |  | SEQUEST (4) |  | 3 |  | 636.32861 |  | 2.72 |  | 1276 |  | 1276 |  | Image |  | Peak List |  |
|  | | | | | | | | | | | | | | | | | | | | | | | | | | | | | | | | |
|  | **A8MXW0 - Rho GDP-dissociation inhibitor 1 OS=Homo sapiens GN=ARHGDIA PE=2 SV=2 - [A8MXW0\_HUMAN]** | | | | | | | | | | | | | | | | | | | | | | | | | | | | | | |  |
|  | | | | | | | | | | | | | | | | | | | | | | | | | | | | | | | | |
|  | High |  | FTDDDKTDHLSWEWNLTIK |  | CID |  |  |  |  |  | 2.91 |  | 1.00 |  | 1 |  | SEQUEST (4) |  | 3 |  | 788.71136 |  | 2.19 |  | 2127 |  | 2127 |  | Image |  | Peak List |  |
|  | | | | | | | | | | | | | | | | | | | | | | | | | | | | | | | | |
|  | **B4DLJ0 - Aldehyde dehydrogenase X, mitochondrial OS=Homo sapiens GN=ALDH1B1 PE=2 SV=1 - [B4DLJ0\_HUMAN]** | | | | | | | | | | | | | | | | | | | | | | | | | | | | | | |  |
|  | | | | | | | | | | | | | | | | | | | | | | | | | | | | | | | | |
|  | High |  | GFFIKPTVFGGVQDDMR |  | CID |  |  |  |  |  | 3.84 |  | 1.00 |  | 1 |  | SEQUEST (4) |  | 3 |  | 638.65924 |  | 2.73 |  | 2155 |  | 2155 |  | Image |  | Peak List |  |
|  | | | | | | | | | | | | | | | | | | | | | | | | | | | | | | | | |
|  | **B4DMW1 - Nischarin OS=Homo sapiens GN=NISCH PE=2 SV=1 - [B4DMW1\_HUMAN]** | | | | | | | | | | | | | | | | | | | | | | | | | | | | | | |  |
|  | | | | | | | | | | | | | | | | | | | | | | | | | | | | | | | | |
|  | High |  | VVGSELVDTYTVYIIQVTDGSHEWTVKHR |  | CID |  |  |  |  |  | 1.91 |  | 1.00 |  | 1 |  | SEQUEST (4) |  | 3 |  | 1111.23572 |  | -0.97 |  | 2679 |  | 2679 |  | Image |  | Peak List |  |
|  | | | | | | | | | | | | | | | | | | | | | | | | | | | | | | | | |
|  | **F5GY93 - Cob(I)yrinic acid a,c-diamide adenosyltransferase, mitochondrial (Fragment) OS=Homo sapiens GN=MMAB PE=4 SV=1 - [F5GY93\_HUMAN]** | | | | | | | | | | | | | | | | | | | | | | | | | | | | | | |  |
|  | | | | | | | | | | | | | | | | | | | | | | | | | | | | | | | | |
|  | High |  | DDQVFEAVGTTDELSSAIGFALELVTEK |  | CID |  |  |  |  |  | 3.28 |  | 1.00 |  | 1 |  | SEQUEST (4) |  | 3 |  | 995.50153 |  | 9.25 |  | 5522 |  | 5522 |  | Image |  | Peak List |  |
|  | | | | | | | | | | | | | | | | | | | | | | | | | | | | | | | | |
|  | **F5H0M0 - Acyl-coenzyme A oxidase OS=Homo sapiens GN=ACOX1 PE=3 SV=1 - [F5H0M0\_HUMAN]** | | | | | | | | | | | | | | | | | | | | | | | | | | | | | | |  |
|  | | | | | | | | | | | | | | | | | | | | | | | | | | | | | | | | |
|  | High |  | SDAVALVDAFDFQDVTLGSVLGR |  | CID |  |  |  |  |  | 1.73 |  | 1.00 |  | 1 |  | SEQUEST (4) |  | 3 |  | 799.08221 |  | 7.51 |  | 3970 |  | 3970 |  | Image |  | Peak List |  |
|  | | | | | | | | | | | | | | | | | | | | | | | | | | | | | | | | |
|  | **F5H1F8 - Chloride intracellular channel protein 4 OS=Homo sapiens GN=CLIC4 PE=4 SV=1 - [F5H1F8\_HUMAN]** | | | | | | | | | | | | | | | | | | | | | | | | | | | | | | |  |
|  | | | | | | | | | | | | | | | | | | | | | | | | | | | | | | | | |
|  | High |  | HPESNTAGMDIFAK |  | CID |  |  |  |  |  | 1.82 |  | 1.00 |  | 1 |  | SEQUEST (4) |  | 2 |  | 759.36090 |  | 6.02 |  | 1224 |  | 1224 |  | Image |  | Peak List |  |
|  | | | | | | | | | | | | | | | | | | | | | | | | | | | | | | | | |
|  | **F5H7D7 - Glycogen synthase kinase-3 alpha OS=Homo sapiens GN=GSK3A PE=4 SV=1 - [F5H7D7\_HUMAN]** | | | | | | | | | | | | | | | | | | | | | | | | | | | | | | |  |
|  | | | | | | | | | | | | | | | | | | | | | | | | | | | | | | | | |
|  | High |  | MGGGVGASSSGGGPGGSGGGGSGGPGAGTSFPPPGVK |  | CID |  |  |  |  |  | 2.72 |  | 1.00 |  | 1 |  | SEQUEST (4) |  | 4 |  | 725.83990 |  | 5.11 |  | 3816 |  | 3816 |  | Image |  | Peak List |  |
|  | | | | | | | | | | | | | | | | | | | | | | | | | | | | | | | | |
|  | **H0Y305 - 26S proteasome non-ATPase regulatory subunit 2 OS=Homo sapiens GN=PSMD2 PE=4 SV=1 - [H0Y305\_HUMAN]** | | | | | | | | | | | | | | | | | | | | | | | | | | | | | | |  |
|  | | | | | | | | | | | | | | | | | | | | | | | | | | | | | | | | |
|  | High |  | QLMSQVAVAGLLTVLVSFLDVR |  | CID |  |  |  |  |  | 2.24 |  | 1.00 |  | 1 |  | SEQUEST (4) |  | 3 |  | 787.12524 |  | 8.18 |  | 5814 |  | 5814 |  | Image |  | Peak List |  |
|  | | | | | | | | | | | | | | | | | | | | | | | | | | | | | | | | |
|  | **H0Y3A5 - Peptidyl-prolyl cis-trans isomerase OS=Homo sapiens GN=FKBP5 PE=4 SV=1 - [H0Y3A5\_HUMAN]** | | | | | | | | | | | | | | | | | | | | | | | | | | | | | | |  |
|  | | | | | | | | | | | | | | | | | | | | | | | | | | | | | | | | |
|  | High |  | IPSNATLFFEIELLDFKGEDLFEDGGIIR |  | CID |  |  |  |  |  | 3.06 |  | 1.00 |  | 1 |  | SEQUEST (4) |  | 3 |  | 1100.24365 |  | 8.50 |  | 4024 |  | 4024 |  | Image |  | Peak List |  |
|  | | | | | | | | | | | | | | | | | | | | | | | | | | | | | | | | |

  
Top
  

### Search Summary

Workflow created with Discoverer version: 1.4.0.288 (DBVersion:79)
  
  
================================================================================
  
  
Search name: Water\_2\_MC3
  
Search description: -
  
Search date: 06/25/2014 16:32:24
  
  
================================================================================
  
  
The pipeline tree:
  
------------------
  
  
    |-(0) Spectrum Files
  
        |-(1) Spectrum Selector
  
            |-(2) Mascot
  
                |-(3) Percolator
  
            |-(4) SEQUEST
  
                |-(3) Percolator
  
  
================================================================================
  
  
Search name: Water\_2\_MC3
  
Search description: -
  
Search date: 06/25/2014 16:32:24
  
  
================================================================================
  
  
The pipeline tree:
  
------------------
  
  
    |-(0) Spectrum Files
  
        |-(1) Spectrum Selector
  
            |-(2) Mascot
  
                |-(3) Percolator
  
            |-(4) SEQUEST
  
                |-(3) Percolator
  
  
------------------------------------------------------------------------------
  
Processing node 0: Spectrum Files
  
------------------------------------------------------------------------------
  
  
Input Data:
  
-----------------------------
  
File Name(s): E:\Jos\Water\_2.raw
  
  
------------------------------------------------------------------------------
  
Processing node 1: Spectrum Selector
  
------------------------------------------------------------------------------
  
  
1. General Settings:
  
-----------------------------
  
Precursor Selection: Use MS1 Precursor
  
Use New Precursor Reevaluation: True
  
  
2. Spectrum Properties Filter:
  
-----------------------------
  
Lower RT Limit: 0
  
Upper RT Limit: 0
  
First Scan: 0
  
Last Scan: 0
  
Lowest Charge State: 0
  
Highest Charge State: 0
  
Min. Precursor Mass: 350 Da
  
Max. Precursor Mass: 5000 Da
  
Total Intensity Threshold: 0
  
Minimum Peak Count: 1
  
  
3. Scan Event Filters:
  
-----------------------------
  
MS Order: Is MS2
  
Activation Type: Is CID
  
Min. Collision Energy: 0
  
Max. Collision Energy: 1000
  
Scan Type: Is Full
  
Ionization Source: Is Nanospray
  
  
4. Peak Filters:
  
-----------------------------
  
S/N Threshold (FT-only): 1.5
  
  
5. Replacements for Unrecognized Properties:
  
-----------------------------
  
Unrecognized Charge Replacements: Automatic
  
Unrecognized Mass Analyzer Replacements: ITMS
  
Unrecognized MS Order Replacements: MS2
  
Unrecognized Activation Type Replacements: CID
  
Unrecognized Polarity Replacements: +
  
  
6. Just for Testing:
  
-----------------------------
  
Precursor Clipping Range Before: 2.5 Da
  
Precursor Clipping Range After: 5.5 Da
  
  
------------------------------------------------------------------------------
  
Processing node 2: Mascot
  
------------------------------------------------------------------------------
  
  
1. Input Data:
  
-----------------------------
  
Protein Database: SwissProt
  
Enzyme Name: Trypsin
  
Maximum Missed Cleavage Sites: 3
  
Instrument: Default
  
Taxonomy: . . . . . . . . . . . . . . . . Homo sapiens (human)
  
  
1.1 Peptide Scoring Options:
  
-----------------------------
  
Peptide Cut Off Score: 10
  
Peptide Without Protein Cut Off Score: 5
  
  
1.2 Protein Scoring Options:
  
-----------------------------
  
Use MudPIT Scoring: Automatic
  
Protein Relevance Threshold: 20
  
Protein Relevance Factor: 1
  
  
2. Tolerances:
  
-----------------------------
  
Precursor Mass Tolerance: 10 ppm
  
Fragment Mass Tolerance: 0.8 Da
  
Use Average Precursor Mass: False
  
  
4. Dynamic Modifications:
  
-----------------------------
  
1. Dynamic Modification: Oxidation (M)
  
  
------------------------------------------------------------------------------
  
Processing node 3: Percolator
  
------------------------------------------------------------------------------
  
  
1. Input Data:
  
-----------------------------
  
Maximum Delta Cn: 0.05
  
  
2. Decoy Database Search:
  
-----------------------------
  
Target FDR (Strict): 0.01
  
Target FDR (Relaxed): 0.05
  
Validation based on: q-Value
  
  
------------------------------------------------------------------------------
  
Processing node 4: SEQUEST
  
------------------------------------------------------------------------------
  
  
1. Input Data:
  
-----------------------------
  
Protein Database: HUMAN\_swiss\_Jos.fasta
  
Enzyme Name: Trypsin (Full)
  
Maximum Missed Cleavage Sites: 3
  
  
1.1 Peptide Scoring Options:
  
-----------------------------
  
Maximum Peptides Considered: 500
  
Maximum Peptides Output: 10
  
Calculate Probability Scores: False
  
Absolute XCorr Threshold: 0.4
  
Fragment Ion Cutoff Percentage: 0.1
  
Peptide Without Protein XCorr Threshold: 1.5
  
  
1.2 Protein Scoring Options:
  
-----------------------------
  
Maximum Protein References Per Peptide: 100
  
Protein Relevance Threshold: 1.5
  
Peptide Relevance Factor: 0.4
  
  
2. Tolerances:
  
-----------------------------
  
Precursor Mass Tolerance: 10 ppm
  
Fragment Mass Tolerance: 0.8 Da
  
Use Average Precursor Mass: False
  
Use Average Fragment Masses: False
  
  
3. Ion Series:
  
-----------------------------
  
Use Neutral Loss a Ions: True
  
Use Neutral Loss b Ions: True
  
Use Neutral Loss y Ions: True
  
Weight of a Ions: 0
  
Weight of b Ions: 1
  
Weight of c Ions: 0
  
Weight of x Ions: 0
  
Weight of y Ions: 1
  
Weight of z Ions: 0
  
  
4. Dynamic Modifications:
  
-----------------------------
  
Max. Modifications Per Peptide: 4
  
1. Dynamic Modification: Oxidation / +15.995 Da (M)
  
  
================================================================================
  
  
Processing details:
  
  
06/25/2014 05:11 PM (4):SEQUEST: Total search time was 1 min 39 s.
  
06/25/2014 05:11 PM (3):Percolator: Performing percolator for SEQUEST (4) took 49.8 s.
  
06/25/2014 05:11 PM (4):SEQUEST: Search completed
  
06/25/2014 05:11 PM (4):SEQUEST: 9092 protein(s) + 8208 decoy proteins scored and inserted into result file in 5.4 s.
  
06/25/2014 05:11 PM (4):SEQUEST: 9092 protein(s) scored
  
06/25/2014 05:11 PM (4):SEQUEST: Search result finalization started.
  
06/25/2014 05:11 PM (3):Percolator: Start reading Percolator results
  
06/25/2014 05:11 PM (3):Percolator: Processing took 7.472 cpu seconds or 8 seconds wall time
  
06/25/2014 05:11 PM (3):Percolator: PSMId score q-value posterior\_error\_prob peptide proteinIds
  
06/25/2014 05:11 PM (3):Percolator: Calibrating statistics - calculating Posterior error probabilities (PEPs)
  
06/25/2014 05:11 PM (3):Percolator: New pi\_0 estimate on merged list gives 899 peptides over q=0.0100
  
06/25/2014 05:11 PM (3):Percolator: Calibrating statistics - calculating q values
  
06/25/2014 05:11 PM (3):Percolator: Selecting pi\_0=0.7028
  
06/25/2014 05:11 PM (3):Percolator: Tossing out "redundant" PSMs keeping only the best scoring PSM for each unique peptide.
  
06/25/2014 05:11 PM (3):Percolator: Merging results from 3 datasets
  
06/25/2014 05:11 PM (3):Percolator: Found 1696 target PSMs scoring over 1.0000% FDR level on testset
  
06/25/2014 05:11 PM (3):Percolator: 0.7930 0.0004 1.1222 0.0206 0.0016 -0.0003 -12.6476 0.0765 34.1898 -0.1050 -0.0213 0.0000 -0.9498 0.1247 0.6148 1.8600 2.2699 -0.5322 0.0000 -0.9148 0.0398 -0.0071 -0.0085 -0.2569 -0.0192 0.1044 0.2320 0.8243 -0.0018 -2.1217 -0.0011 -0.4911 -0.0002 1.5770
  
06/25/2014 05:11 PM (3):Percolator: 0.718 0.1169 0.3069 0.7077 0.0404 -0.2690 -0.2085 0.4450 0.3100 -0.2889 -0.1661 0.0000 -0.4096 0.0621 0.2345 0.3035 0.1239 -0.5927 0.0000 -0.3340 0.4192 -0.1092 -0.1381 -0.5202 -0.0391 0.2144 0.6077 0.1589 -0.5265 -0.2141 -0.1760 -0.0400 -0.0412 -2.2632
  
06/25/2014 05:11 PM (3):Percolator: XCorr SpScore Delta Cn From Second PSM Binomial Score Isolation Interference [%] MH+ [Da] Delta Mass [Da] Delta Mass [ppm] Absolute Delta Mass [Da] Absolute Delta Mass [ppm] Peptide Length Is z=1 Is z=2 Is z=3 Is z=4 Is z=5 Is z>5 # Missed Cleavages Log Peptides Matched Log Total Intensity Fraction Matched Intensity [%] Fragment Coverage Series A, B, C [%] Fragment Coverage Series X, Y, Z [%] Log Matched Fragment Series Intensities A, B, C Log Matched Fragment Series Intensities X, Y, Z Longest Sequence Series A, B, C Longest Sequence Series X, Y, Z IQR Fragment Delta Mass [Da] IQR Fragment Delta Mass [ppm] Mean Fragment Delta Mass [Da] Mean Fragment Delta Mass [ppm] Mean Absolute Fragment Delta Mass [Da] Mean Absolute Fragment Delta Mass [ppm] m0
  
06/25/2014 05:11 PM (3):Percolator: # first line contains normalized weights, second line the raw weights
  
06/25/2014 05:11 PM (3):Percolator: Obtained weights (only showing weights of first cross validation set)
  
06/25/2014 05:11 PM (3):Percolator: Iteration 10 : After the iteration step, 1794 target PSMs with q<0.01 were estimated by cross validation
  
06/25/2014 05:11 PM (3):Percolator: Iteration 9 : After the iteration step, 1794 target PSMs with q<0.01 were estimated by cross validation
  
06/25/2014 05:11 PM (3):Percolator: Iteration 8 : After the iteration step, 1793 target PSMs with q<0.01 were estimated by cross validation
  
06/25/2014 05:11 PM (3):Percolator: Iteration 7 : After the iteration step, 1792 target PSMs with q<0.01 were estimated by cross validation
  
06/25/2014 05:11 PM (3):Percolator: Iteration 6 : After the iteration step, 1792 target PSMs with q<0.01 were estimated by cross validation
  
06/25/2014 05:11 PM (3):Percolator: Iteration 5 : After the iteration step, 1787 target PSMs with q<0.01 were estimated by cross validation
  
06/25/2014 05:11 PM (3):Percolator: Iteration 4 : After the iteration step, 1787 target PSMs with q<0.01 were estimated by cross validation
  
06/25/2014 05:11 PM (3):Percolator: Iteration 3 : After the iteration step, 1776 target PSMs with q<0.01 were estimated by cross validation
  
06/25/2014 05:11 PM (3):Percolator: Iteration 2 : After the iteration step, 1757 target PSMs with q<0.01 were estimated by cross validation
  
06/25/2014 05:11 PM (3):Percolator: Iteration 1 : After the iteration step, 1659 target PSMs with q<0.01 were estimated by cross validation
  
06/25/2014 05:11 PM (3):Percolator: ---Training with Cpos selected by cross validation, Cneg selected by cross validation, fdr=0.01
  
06/25/2014 05:11 PM (3):Percolator: Reading in data and feature calculation took 7.675 cpu seconds or 8 seconds wall time
  
06/25/2014 05:11 PM (3):Percolator: Estimating 1142 over q=0.01 in initial direction
  
06/25/2014 05:11 PM (3):Percolator: Selected feature number 1 as initial search direction, could separate 763 positives in that direction
  
06/25/2014 05:11 PM (3):Percolator: Selected feature number 1 as initial search direction, could separate 767 positives in that direction
  
06/25/2014 05:11 PM (3):Percolator: Selected feature number 1 as initial search direction, could separate 754 positives in that direction
  
06/25/2014 05:11 PM (3):Percolator: selecting cneg by cross validation
  
06/25/2014 05:11 PM (3):Percolator: selecting cpos by cross validation
  
06/25/2014 05:11 PM (3):Percolator: Train/test set contains 4685 positives and 5361 negatives, size ratio=0.873904 and pi0=1
  
06/25/2014 05:11 PM (3):Percolator: 31e77142-29e9-402c-9ec2-8468a9513af0 e39a792e-622c-452d-b49b-59809cad79d0 Delta Cn From Second PSM Binomial Score b8754504-e95e-476b-b9a4-454d4bb53aeb 1d91a87b-953a-4887-9f22-f75a497a3538 Delta Mass [Da] Delta Mass [ppm] Absolute Delta Mass [Da] Absolute Delta Mass [ppm] Peptide Length Is z=1 Is z=2 Is z=3 Is z=4 Is z=5 Is z>5 041eb6d5-e486-44a0-9bc1-19e25811c686 Log Peptides Matched Log Total Intensity Fraction Matched Intensity [%] Fragment Coverage Series A, B, C [%] Fragment Coverage Series X, Y, Z [%] Log Matched Fragment Series Intensities A, B, C Log Matched Fragment Series Intensities X, Y, Z Longest Sequence Series A, B, C Longest Sequence Series X, Y, Z IQR Fragment Delta Mass [Da] IQR Fragment Delta Mass [ppm] Mean Fragment Delta Mass [Da] Mean Fragment Delta Mass [ppm] Mean Absolute Fragment Delta Mass [Da] Mean Absolute Fragment Delta Mass [ppm]
  
06/25/2014 05:11 PM (3):Percolator: Features:
  
06/25/2014 05:11 PM (3):Percolator: enzyme=Trypsin
  
06/25/2014 05:11 PM (3):Percolator: Hyperparameters fdr=0.01, Cpos=0, Cneg=0, maxNiter=10
  
06/25/2014 05:11 PM (3):Percolator: Started Wed Jun 25 17:11:14 2014
  
06/25/2014 05:11 PM (3):Percolator: C:\Program Files\Thermo\Discoverer 1.4\Tools\Percolator\percolator.exe -X C:\ProgramData\Thermo\Discoverer 1.4\Scratch\b9f94b98-7087-4213-8d7f-0fe57cab16bf\output.xml -Z C:\ProgramData\Thermo\Discoverer 1.4\Scratch\b9f94b98-7087-4213-8d7f-0fe57cab16bf\input.xml
  
06/25/2014 05:11 PM (3):Percolator: Issued command:
  
06/25/2014 05:11 PM (3):Percolator: Department of Genome Sciences at the University of Washington.
  
06/25/2014 05:11 PM (3):Percolator: Written by Lukas K+�ll (lukall@u.washington.edu) in the
  
06/25/2014 05:11 PM (3):Percolator: Copyright (c) 2006-9 University of Washington. All rights reserved.
  
06/25/2014 05:11 PM (3):Percolator: Percolator version 2.04, Build Date Feb 1 2012 03:35:34
  
06/25/2014 05:11 PM (3):Percolator: Starting Percolator
  
06/25/2014 05:11 PM (3):Percolator: The input file contains 4685 peptides, 5361 decoy peptides and 33 features.
  
06/25/2014 05:11 PM (3):Percolator: Creating input file for SEQUEST (4) took 32.4 s.
  
06/25/2014 05:10 PM (3):Percolator: Start calculating features for peptides of SEQUEST (4)
  
06/25/2014 05:10 PM (2):Mascot: Total search time was 1 min 54 s.
  
06/25/2014 05:10 PM (3):Percolator: Performing percolator for Mascot (2) took 44.2 s.
  
06/25/2014 05:10 PM (2):Mascot: Search completed
  
06/25/2014 05:10 PM (2):Mascot: 323 protein(s) + 17 decoy proteins scored and inserted into result file in 0.6 s.
  
06/25/2014 05:10 PM (2):Mascot: 323 protein(s) scored
  
06/25/2014 05:10 PM (2):Mascot: Search result finalization started.
  
06/25/2014 05:10 PM (3):Percolator: Start reading Percolator results
  
06/25/2014 05:10 PM (3):Percolator: Processing took 6.084 cpu seconds or 6 seconds wall time
  
06/25/2014 05:10 PM (3):Percolator: PSMId score q-value posterior\_error\_prob peptide proteinIds
  
06/25/2014 05:10 PM (3):Percolator: Calibrating statistics - calculating Posterior error probabilities (PEPs)
  
06/25/2014 05:10 PM (3):Percolator: New pi\_0 estimate on merged list gives 865 peptides over q=0.0100
  
06/25/2014 05:10 PM (3):Percolator: Calibrating statistics - calculating q values
  
06/25/2014 05:10 PM (3):Percolator: Selecting pi\_0=0.7152
  
06/25/2014 05:10 PM (3):Percolator: Tossing out "redundant" PSMs keeping only the best scoring PSM for each unique peptide.
  
06/25/2014 05:10 PM (3):Percolator: Merging results from 3 datasets
  
06/25/2014 05:10 PM (3):Percolator: Found 1521 target PSMs scoring over 1.0000% FDR level on testset
  
06/25/2014 05:10 PM (3):Percolator: 0.0189 0.8719 0.0485 0.0229 -0.0047 -42.6809 0.2333 342.9737 -1.0733 0.2285 0.0000 -1.6601 1.0678 -0.0337 0.4815 -2.1761 -2.0070 0.0000 -2.2189 0.0304 -0.0236 -0.0751 -0.6888 1.8593 0.5799 1.0687 0.4686 -0.0031 -17.1877 0.0020 -20.3459 -0.0018 12.1807
  
06/25/2014 05:10 PM (3):Percolator: 0.255 0.2621 1.7493 0.6047 -4.4176 -0.7439 1.3280 3.3226 -2.9139 2.0278 0.0000 -0.6619 0.5295 -0.0137 0.0790 -0.1153 -2.2452 0.0000 -1.1645 0.3760 -0.4056 -1.4356 -2.9387 4.1218 1.2730 3.0969 0.1037 -1.0123 -1.9572 0.3133 -1.9519 -0.3439 -6.3406
  
06/25/2014 05:10 PM (3):Percolator: IonScore Delta Cn From Second PSM Binomial Score Isolation Interference [%] MH+ [Da] Delta Mass [Da] Delta Mass [ppm] Absolute Delta Mass [Da] Absolute Delta Mass [ppm] Peptide Length Is z=1 Is z=2 Is z=3 Is z=4 Is z=5 Is z>5 # Missed Cleavages Log Peptides Matched Log Total Intensity Fraction Matched Intensity [%] Fragment Coverage Series A, B, C [%] Fragment Coverage Series X, Y, Z [%] Log Matched Fragment Series Intensities A, B, C Log Matched Fragment Series Intensities X, Y, Z Longest Sequence Series A, B, C Longest Sequence Series X, Y, Z IQR Fragment Delta Mass [Da] IQR Fragment Delta Mass [ppm] Mean Fragment Delta Mass [Da] Mean Fragment Delta Mass [ppm] Mean Absolute Fragment Delta Mass [Da] Mean Absolute Fragment Delta Mass [ppm] m0
  
06/25/2014 05:10 PM (3):Percolator: # first line contains normalized weights, second line the raw weights
  
06/25/2014 05:10 PM (3):Percolator: Obtained weights (only showing weights of first cross validation set)
  
06/25/2014 05:10 PM (3):Percolator: Iteration 10 : After the iteration step, 1658 target PSMs with q<0.01 were estimated by cross validation
  
06/25/2014 05:10 PM (3):Percolator: Iteration 9 : After the iteration step, 1658 target PSMs with q<0.01 were estimated by cross validation
  
06/25/2014 05:10 PM (3):Percolator: Iteration 8 : After the iteration step, 1658 target PSMs with q<0.01 were estimated by cross validation
  
06/25/2014 05:10 PM (3):Percolator: Iteration 7 : After the iteration step, 1658 target PSMs with q<0.01 were estimated by cross validation
  
06/25/2014 05:10 PM (3):Percolator: Iteration 6 : After the iteration step, 1657 target PSMs with q<0.01 were estimated by cross validation
  
06/25/2014 05:10 PM (3):Percolator: Iteration 5 : After the iteration step, 1656 target PSMs with q<0.01 were estimated by cross validation
  
06/25/2014 05:10 PM (3):Percolator: Iteration 4 : After the iteration step, 1659 target PSMs with q<0.01 were estimated by cross validation
  
06/25/2014 05:10 PM (3):Percolator: Iteration 3 : After the iteration step, 1660 target PSMs with q<0.01 were estimated by cross validation
  
06/25/2014 05:10 PM (3):Percolator: Iteration 2 : After the iteration step, 1636 target PSMs with q<0.01 were estimated by cross validation
  
06/25/2014 05:10 PM (3):Percolator: Iteration 1 : After the iteration step, 1555 target PSMs with q<0.01 were estimated by cross validation
  
06/25/2014 05:10 PM (3):Percolator: ---Training with Cpos selected by cross validation, Cneg selected by cross validation, fdr=0.01
  
06/25/2014 05:10 PM (3):Percolator: Reading in data and feature calculation took 6.084 cpu seconds or 6 seconds wall time
  
06/25/2014 05:10 PM (3):Percolator: Estimating 1048 over q=0.01 in initial direction
  
06/25/2014 05:10 PM (3):Percolator: Selected feature number 1 as initial search direction, could separate 687 positives in that direction
  
06/25/2014 05:10 PM (3):Percolator: Selected feature number 1 as initial search direction, could separate 692 positives in that direction
  
06/25/2014 05:10 PM (3):Percolator: Selected feature number 1 as initial search direction, could separate 717 positives in that direction
  
06/25/2014 05:10 PM (3):Percolator: selecting cneg by cross validation
  
06/25/2014 05:10 PM (3):Percolator: selecting cpos by cross validation
  
06/25/2014 05:10 PM (3):Percolator: Train/test set contains 4217 positives and 3953 negatives, size ratio=1.06678 and pi0=1
  
06/25/2014 05:10 PM (3):Percolator: e6e22773-e9a6-4a26-9694-1ca77a797099 Delta Cn From Second PSM Binomial Score b8754504-e95e-476b-b9a4-454d4bb53aeb 1d91a87b-953a-4887-9f22-f75a497a3538 Delta Mass [Da] Delta Mass [ppm] Absolute Delta Mass [Da] Absolute Delta Mass [ppm] Peptide Length Is z=1 Is z=2 Is z=3 Is z=4 Is z=5 Is z>5 041eb6d5-e486-44a0-9bc1-19e25811c686 Log Peptides Matched Log Total Intensity Fraction Matched Intensity [%] Fragment Coverage Series A, B, C [%] Fragment Coverage Series X, Y, Z [%] Log Matched Fragment Series Intensities A, B, C Log Matched Fragment Series Intensities X, Y, Z Longest Sequence Series A, B, C Longest Sequence Series X, Y, Z IQR Fragment Delta Mass [Da] IQR Fragment Delta Mass [ppm] Mean Fragment Delta Mass [Da] Mean Fragment Delta Mass [ppm] Mean Absolute Fragment Delta Mass [Da] Mean Absolute Fragment Delta Mass [ppm]
  
06/25/2014 05:10 PM (3):Percolator: Features:
  
06/25/2014 05:10 PM (3):Percolator: enzyme=Trypsin
  
06/25/2014 05:10 PM (3):Percolator: Hyperparameters fdr=0.01, Cpos=0, Cneg=0, maxNiter=10
  
06/25/2014 05:10 PM (3):Percolator: Started Wed Jun 25 17:10:26 2014
  
06/25/2014 05:10 PM (3):Percolator: C:\Program Files\Thermo\Discoverer 1.4\Tools\Percolator\percolator.exe -X C:\ProgramData\Thermo\Discoverer 1.4\Scratch\74a0729b-6e0e-4960-8a68-fbba47b6925e\output.xml -Z C:\ProgramData\Thermo\Discoverer 1.4\Scratch\74a0729b-6e0e-4960-8a68-fbba47b6925e\input.xml
  
06/25/2014 05:10 PM (3):Percolator: Issued command:
  
06/25/2014 05:10 PM (3):Percolator: Department of Genome Sciences at the University of Washington.
  
06/25/2014 05:10 PM (3):Percolator: Written by Lukas K+�ll (lukall@u.washington.edu) in the
  
06/25/2014 05:10 PM (3):Percolator: Copyright (c) 2006-9 University of Washington. All rights reserved.
  
06/25/2014 05:10 PM (3):Percolator: Percolator version 2.04, Build Date Feb 1 2012 03:35:34
  
06/25/2014 05:10 PM (3):Percolator: Starting Percolator
  
06/25/2014 05:10 PM (3):Percolator: The input file contains 4217 peptides, 3953 decoy peptides and 32 features.
  
06/25/2014 05:10 PM (3):Percolator: Creating input file for Mascot (2) took 30.3 s.
  
06/25/2014 05:09 PM (3):Percolator: Start calculating features for peptides of Mascot (2)
  
06/25/2014 05:09 PM (2):Mascot: Used mascot server http://fenn.bham.ac.uk/mascot/ with Mascot version 2.4.1
  
06/25/2014 05:09 PM (2):Mascot: Sending 3715 peptide hits (15456 peptides) to result file
  
06/25/2014 05:09 PM (2):Mascot: Sending 3296 decoy peptide hits (13829 peptides) to result file
  
06/25/2014 05:09 PM (2):Mascot: Reading decoy results
  
06/25/2014 05:09 PM (2):Mascot: Start translating results
  
06/25/2014 05:09 PM (2):Mascot: Start mapping modifications
  
06/25/2014 05:09 PM (2):Mascot: Received 330 proteins from Mascot server
  
06/25/2014 05:09 PM (2):Mascot: Start mapping 330 proteins
  
06/25/2014 05:09 PM (2):Mascot: Start parsing results
  
06/25/2014 05:09 PM (2):Mascot: Received Mascot result file (filename=../data/20140625/F004281.dat)
  
06/25/2014 05:09 PM (2):Mascot: Mascot Server completed
  
06/25/2014 05:08 PM (2):Mascot: Mascot result on server (filename=../data/20140625/F004281.dat)
  
06/25/2014 05:08 PM (2):Mascot: Start searching 4593 spectra
  
06/25/2014 05:08 PM (4):SEQUEST: Sending 593 decoy peptide hits (3357 peptides) to result file
  
06/25/2014 05:07 PM (4):SEQUEST: Starting SEQUEST decoy search
  
06/25/2014 05:07 PM (4):SEQUEST: Sending 593 peptide hits (3361 peptides) to result file
  
06/25/2014 05:07 PM (4):SEQUEST: Starting SEQUEST (search spectra 4000 - 4593)
  
06/25/2014 05:07 PM (4):SEQUEST: Sending 1000 decoy peptide hits (6447 peptides) to result file
  
06/25/2014 05:07 PM (4):SEQUEST: Starting SEQUEST decoy search
  
06/25/2014 05:07 PM (4):SEQUEST: Sending 1000 peptide hits (6483 peptides) to result file
  
06/25/2014 05:07 PM (4):SEQUEST: Starting SEQUEST (search spectra 3000 - 4000)
  
06/25/2014 05:07 PM (4):SEQUEST: Sending 1000 decoy peptide hits (8408 peptides) to result file
  
06/25/2014 05:07 PM (4):SEQUEST: Starting SEQUEST decoy search
  
06/25/2014 05:07 PM (4):SEQUEST: Sending 1000 peptide hits (8411 peptides) to result file
  
06/25/2014 05:07 PM (4):SEQUEST: Starting SEQUEST (search spectra 2000 - 3000)
  
06/25/2014 05:06 PM (4):SEQUEST: Sending 1000 decoy peptide hits (9482 peptides) to result file
  
06/25/2014 05:06 PM (4):SEQUEST: Starting SEQUEST decoy search
  
06/25/2014 05:06 PM (4):SEQUEST: Sending 1000 peptide hits (9495 peptides) to result file
  
06/25/2014 05:06 PM (4):SEQUEST: Starting SEQUEST (search spectra 1000 - 2000)
  
06/25/2014 05:06 PM (4):SEQUEST: Sending 1000 decoy peptide hits (9619 peptides) to result file
  
06/25/2014 05:06 PM (4):SEQUEST: Starting SEQUEST decoy search
  
06/25/2014 05:06 PM (4):SEQUEST: Sending 1000 peptide hits (9634 peptides) to result file
  
06/25/2014 05:05 PM (4):SEQUEST: Starting SEQUEST (search spectra 0 - 1000)
  
06/25/2014 05:05 PM (4):SEQUEST: There is already an adequate decoy FASTA index.
  
06/25/2014 05:05 PM (4):SEQUEST: Looking for existing decoy FASTA index.
  
06/25/2014 05:05 PM (4):SEQUEST: There is already an adequate target FASTA index.
  
06/25/2014 05:05 PM (4):SEQUEST: Looking for existing target FASTA index.
  
06/25/2014 05:05 PM (2):Mascot: Use mascot server http://fenn.bham.ac.uk/mascot/ with Mascot version 2.4.1
  
06/25/2014 05:05 PM (1):Spectrum Selector: Reading from File 1 of 1:E:\Jos\Water\_2.raw (7657 spectra total)
  
  
  
Top
